# Perspectives of HPV vaccine decision-making among young adults: A qualitative systematic review and evidence synthesis

**DOI:** 10.1371/journal.pone.0321448

**Published:** 2025-05-05

**Authors:** Namoonga M. Mantina, Jonathan Smith, Flavia Nakayima Miiro, Priscilla Anne Magrath, Deborah Jean McClelland, Leila Barraza, John Ruiz, Purnima Madhivanan

**Affiliations:** 1 Department of Health Promotion Sciences, Mel & Enid Zuckerman College of Public Health, University of Arizona, Tucson, Arizona, United States of America; 2 Department of Epidemiology, Mel & Enid Zuckerman College of Public Health, University of Arizona, Tucson, Arizona, United States of America; 3 Arizona Health Sciences Library, University of Arizona, Tucson, Arizona, United States of America; 4 Department of Public Health Practice, Policy, & Translational Research, Mel & Enid Zuckerman College of Public Health, University of Arizona, Tucson, Arizona, United States of America; 5 Department of Psychology, College of Sciences, University of Arizona, Tucson, Arizona, United States of America; 6 Division of Infectious Diseases, Department of Medicine, College of Medicine, University of Arizona, Tucson, Arizona, United States of America; Centers for Disease Control and Prevention, UNITED STATES OF AMERICA

## Abstract

**Background:**

Despite the demonstrated safety and effectiveness of HPV vaccines in preventing HPV-related cancers, global vaccine coverage remains low. The suboptimal adolescent HPV vaccine coverage rate leaves many young adults at increased risk for developing vaccine preventable HPV-related cancers. This qualitative evidence synthesis (QES) aims to examine the HPV vaccination perspectives of young adults globally and identify the barriers and facilitators to HPV vaccine uptake and decision-making processes.

**Methods:**

A comprehensive search was conducted on October 2023 across seven databases to identify studies that reported on HPV vaccination among young adults aged 18–26 years and used qualitive study methods or analysis techniques.

**Results:**

Forty-two studies were purposively sampled for inclusion, presenting 29 findings across 10 thematic categories. Vaccine eligible young adults believed that they had aged out of eligibility for HPV vaccination. There was also a perspective that condom use, and regular screenings were alternatives to vaccination in preventing HPV infections. Challenges included scheduling appointments, requirements for multiple shots, and vaccine cost. There was also concern for the gendered nature of vaccine promotion. Lastly, despite being at the age to make autonomous decisions, parents were still influential and active in the vaccine decision-making process for their children.

**Conclusion:**

The novelty of this study, as one of the principal QES on catch-up HPV vaccination, presents findings that underscore the complexity of factors across multiple ecological levels which may aid or impede vaccination uptake among young adults and provide important considerations for interventions, programs, and policies aimed at addressing HPV vaccination disparities among young adults.

## Background

Cancer is among the top two leading causes of death across 127 countries globally [1]. The human papillomavirus (HPV) is responsible for approximately 4.5% of all cancer cases worldwide [2] and persistent HPV infection can lead to cancers of the cervix, penis, vulva, vagina, anus, and oropharynx [3]. Cervical cancer is the second most diagnosed cancer among women and second leading cause of death among women worldwide [4]; this has significant societal implications, for example, nearly 210,000 children globally become orphans because their mother died from cervical cancer in 2020 [5].

The first HPV vaccine became publicly available in 2006. The Centers for Disease Control and Prevention (CDC) advocate for the initiation of a two-dose HPV vaccination series in adolescents aged 9–14 and a three-dose regimen for teenagers and young adults between ages 15 and 26 [6]. In December 2022, the World Health Organization (WHO) updated its guidance on the HPV vaccine, introducing a single-dose scheduling option for girls and women aged 9–20 years [7,8]. Despite the demonstrated safety and effectiveness of HPV vaccines in preventing HPV-related cancers, global adolescent vaccine coverage continues to remain low, estimated at only 15% for females and 4% for males [9]. HPV vaccination during early adolescence is critical in reducing the risk of HPV-related cancers. However, not all adolescents receive the vaccine, leading to disparities in vaccination rates across regions [9,10]. Additionally, individuals aged 15–24 account for nearly half of all new sexually transmitted infections (STI) cases annually [11] and it’s estimated that over half of HPV infections that develop into cancer are acquired by the age of 21 [12]. The suboptimal adolescent coverage rate leaves many young adults at increased risk for developing vaccine preventable HPV-related cancers; these young adults remain eligible for the vaccine and have increased autonomy in making healthcare decisions and shaping their health behaviors [13].

Young or emerging adulthood is a distinct developmental phase from adolescence [14] that is characterized by identity exploration, instability, and feeling “in-between”, which result from pivotal life transitions, a focus on “self” and optimism of the possibilities available [15]. Effectively promoting HPV vaccination among young adults requires a deeper comprehension of the unique factors characterizing the vaccination behaviors and influences of this demographic. Interventions to increase vaccination uptake in young adults have had varying effectiveness; prior reviews have reported that majority of interventions designed to increase HPV vaccination in young adults did not result in significant increases in vaccine uptake [16,17]. Interventions also lack participant diversity [18,19] which further exacerbates HPV-related disease inequities and undermines the discovery of how different intervention components may work for diverse populations [19,20].

Although several systematic reviews have evaluated HPV vaccination interventions among young adults, there remains a need for further research in this area to enhance our understanding of HPV vaccine promotion and decision-making for these individuals. One review examining interventions to promote HPV vaccination globally discovered that only 25% of interventions conducted between 2015 and 2020 targeted young adults aged 18–34 years [21]. Furthermore, the broad age range of participants included in reviews presents challenges in understanding the unique factors faced by young adults aged 18–26 years who are eligible for HPV vaccination. For instance, two reviews assessing the effectiveness of HPV interventions targeted individuals 9–26 years [18,22] and another review identifying effective strategies to enhance HPV vaccine uptake encompassed participants aged 11–26 years [19]. Notably, nearly all the systematic review literature on HPV vaccination among young adults have been quantitative syntheses. Qualitative research is suitable for exploring the intricate nuances and complexities that shape vaccination behavior and decision-making processes, subsequently providing a better understanding of how various factors interplay. The only identified qualitative systematic review on HPV vaccination did not have a registered review protocol and exclusively explored adolescent girls aged 9–18 years in high income countries [23].

Given these collective factors—the suboptimal adolescent HPV vaccine coverage rate, limited HPV vaccine interventions targeting young adults, and the wide age range of young adult participants included in HPV vaccine research—there is a crucial opportunity to specifically intervene and promote catch-up HPV vaccination among young adults aged 18–26. To the extent known, there is no published systematic qualitative evidence synthesis of global literature on HPV vaccination among young adults 18–26 years old. The aim of this qualitative evidence synthesis (QES) is to examine the HPV vaccination perspectives of young adults aged 18–26 years and identify barriers and facilitators to HPV vaccine uptake and decision-making processes for this population.

## Methods

This systematic review adheres to the reporting guidelines of the Preferred Reporting Items for Systematic Review and Meta-Analysis (PRISMA) [24] and the QES guidelines detailed by Cochrane [25]. This review was conducted in accordance with the review protocol that was registered with the International Prospective Register of Systematic Reviews (PROSPERO) on April 30, 2023 (PROSPERO registration number CRD42023417052) and published with BMJ Open [26]. Any amendments or deviations from the protocol are documented and reported in this manuscript.

### Review team reflexivity

This systematic review engaged a multidisciplinary team of researchers who represented diverse social identities and research experiences pertinent to the topic. Academic disciplines represented include health promotion sciences, epidemiology, and medical anthropology. Full details of the review team are documented in the protocol paper for this manuscript [26].

### Study eligibility criteria

This review evaluated primary studies that reported on the perspectives, factors and/or decision-making processes of young adults aged 18–26 years. The inclusion and exclusion criteria for this review as aligned with the protocol follow below.

#### Inclusion criteria.

- Reported on HPV vaccination views, perspectives, decision of individuals aged 18–26 years,
- Studies that used qualitative study designs (e.g., ethnography, case studies),
- Studies that used qualitative methods for data collection (e.g., focus groups, interviews, open-ended survey questions),
- Studies that used qualitative data analysis methods (e.g., thematic analysis, grounded theory)
- Mixed methods studies where it is possible to extract data that were collected and analyzed using qualitative methods, and
- We included studies regardless of their linkage to an intervention and irrespective of where vaccination took place or how it was delivered.

#### Exclusion criteria.

- Studies that did not include young adults 18–26 years old and solely presented the perspectives and views of other stakeholders (such as parents, healthcare providers, and policymakers) on the topic of young adults HPV vaccination uptake,
- Articles that were protocols, reviews, conference proceedings and opinion reports,
- Studies that collected data using qualitative methods but did not analyze these data using qualitative analysis methods (e.g., open-ended survey questions where the response data are analyzed using descriptive statistics only),
- Studies that evaluated multiple vaccines, but did not report on HPV separately or it was not possible to extract data specific to HPV,
- Studies where it was not possible to separate and extract data on views of HPV vaccination specific to young adults from views of vaccination of other age groups/demographics (e.g., adolescents under 18 years, studies that included a wide age range of participants, but the qualitative data reported did not explicitly state the age of the participant quoted).

### Information sources and search strategy

We searched the following seven (7) electronic databases to identify studies for inclusion: PubMed, SCOPUS (Elsevier), Embase (Elsevier), the Cochrane Library (Wiley), PsycINFO (EBSCOhost), CINAHL (EBSCOhost) and CABI Global Health (EBSCOhost). No search restrictions were posed on geographic region/country where studies were conducted, nor on language of the manuscript. A comprehensive search strategy was developed and adapted for each database. Key words and Medical Subject Headings (MeSH) terms were combined related to “HPV”, “vaccination”, “young adults” and qualitative methodological filters (e.g., “focus group”, “interview”). Full details of the search terms for each database are details in S1 Appendix.

All databases were searched by NMM with support from DJM beginning in 2006 (the year HPV vaccines were publicly available) up to the date of the search. The initial search was conducted on May 11, 2023. Updates were made to the search terms following peer review feedback on the protocol manuscript and the search was rerun on October 12, 2023. As screening procedures had commenced following the initial search, we followed the procedures outlined by Bramer & Bain [27] to identify the new records retrieved from the updated search (S2 Appendix). Lastly, the reference lists of all eligible studies were also manually searched to identify additional studies for inclusion.

### Selection of studies

The citation manager software EndNote (Clarivate, London, UK) was used to manage and deduplicate all the studies identified through the electronic database search. The deduplicated citation file was then imported into the literature review software DistillerSR (Evidence Partners, Ontario, Canada) for screening in accordance with the review protocol. DistillerSR further identified additional duplicate records which were removed using the software’s functionality.

Studies were screened through a title/abstract review stage and a full text review stage to ascertain inclusion as guided by the eligibility criteria. Each study was independently assessed by two review authors at each screening stage. Any disagreements in evaluation were resolved by discussion and consensus between reviewers. While there were no language restrictions implemented during the database search, there were two non-English full text articles obtained (one Korean and one Spanish). To facilitate the review for inclusion, Google Translate was initially used to aid with reviewing the methods section of the articles. It was determined that both articles did not meet inclusion criteria at full text and were subsequently excluded. After the two stages of screening, 71 articles met the review eligibility criteria for consideration to be included in the review.

### Sampling of studies

In QES, managing the volume of data examined is crucial to uphold analysis quality [28]. Maximizing the heterogeneity of identified themes and concepts to foster a more profound understanding of the research subject is prioritized over exhaustively assessing every study [29]. Consequently, purposively sampling methods were used to aid the management and analysis of the qualitative data to be extracted. In alignment with the approaches taken by other qualitative review authors [30], we purposively sampled a subset of the studies for analysis, using an approach called criterion sampling [31,32], in which studies are sampled based on meeting predetermined criteria. Given the aim of the review was to understand HPV catchup vaccination in individuals aged 18–26, the age criterion employed for sampling inclusion was that studies exclusively recruited participants 18–26 years old. We believed that this sampling criterion would best facilitate obtaining focused and rich information essential to the research question. Forty-five (45) articles were included in the review following purposive sampling.

### Data Extraction

The data extraction process was performed in 2 steps, an adjustment from the original protocol. Step 1 was the extraction of the study characteristics, and step 2 was the extraction of qualitative data. Aligned with the protocol, step 1 of data extraction was performed in DistillerSR, using a data extraction form specifically created for this review. Two reviewers independently extracted data from each study and discrepancies were resolved by a third reviewer. The study characteristics data extracted from eligible studies at step 1 were:

- publication year
- data collection year
- context (study country; rural vs urban)
- participants (number of participants; demographic characteristics, e.g., age, gender, race/ethnicity, sexual orientation)
- study design (objective/research question, sample recruitment, data collection methods, theory frameworks or conceptual models used, analysis methods, funding)

All articles that met review eligibility (n=71) had their data extracted at step 1. This was done to be transparent about the existing literature that was applicable to research question, even though we may not report on all of it for this review.

Step 2 of the data extraction process was conducted in REDCap (Research Electronic Data Capture) [33] as the review team found it was better suited to handle qualitative data. This step was performed only on the included articles that met the criterion sampling (n=45). The information extracted in step 2 were 1) the themes or constructs developed by the primary study authors, including any contextual explanation for how the themes/concepts were defined by the primary authors; and 2) verbatim participant quotes provided. For consistency in how the qualitative data were extracted across all the articles, step 2 data extraction was performed by one review author (NMM).

### Assessment of the methodological limitations of included studies

The methodological limitations of each included study was independently assessed by two review authors using the Critical Appraisal Skills Programme (CASP) quality assessment tool for qualitative studies [34]. An adapted version of the tool was used as guided by other reviews [28,35,36] and was piloted on a subset of studies to assess feasibility and integrity of the assessment. Disagreements were resolved by a third review author.

### Data synthesis

We used Braun and Clark’s methodological process of thematic analysis to analyze and synthesize the qualitative data [37]. Data was analyzed using qualitative analysis software Dedoose version 9.2.005. The following stages of thematic analysis were undertaken:

- *Familiarization*: Three review authors (NMM, JS, FNM) reviewed all the extracted data. Notes were taken on the impression of the data.
- *Generating initial codes*: The aforementioned review authors initially coded data from three different studies that had the largest amount of data extracted, to inductively identify and generate initial codes; codes were also formed from the notes taken during the familiarization stage. After this initial coding, the review authors met to discuss the codes identified, consolidate any duplicate codes, and finalize the codebook. Another round of coding was performed on three additional studies to verify the codebook; no new codes emerged. Each study was then independently coded by two review authors. During the coding process, authors were able to take additional notes in Dedoose pertaining to trends and observations they noticed as they coded. After coding, the two review authors assigned to each study met to discuss any discrepancies in coding and decide the finalized version of the coded data.
- *Generating, reviewing, and finalizing themes*: Once all the data were coded, review author NMM generated the initial themes and subthemes from the codes, and any notes take from the coding process. The initial themes generated formed the basis of the review findings which were subsequently reviewed and revised by the other authors involved in the coding process (JS, FNM).

### Assessment of confidence in synthesized findings

As recommended by the Cochrane handbook on QES, we used the GRADE- CERQual (Grading of Recommendations Assessment, Development and Evaluation - Confidence in the Evidence from Reviews of Qualitative research) [38] to evaluate the confidence in synthesized findings. The CERQual confidence assessment is based on the following four key components:

- *Methodological limitations of included studies*: the extent to which there were concerns about the design or conduct of the primary studies that contributed evidence to an individual review finding [39].
- *Coherence of the review finding*: an assessment of how clear and cogent (i.e., well-supported or compelling) the fit was between the data from the primary studies and a review finding that synthesized those data [40]
- *Adequacy of the data contributing to a review finding*: an overall determination of the degree of richness and quantity of data supporting a review finding [41].
- *Relevance of the included studies to the review question*: the extent to which the body of evidence from the primary studies supporting a review finding was applicable to the context (perspective or population, phenomenon of interest, setting) specified in the review question [42].

To facilitate this process, we used the GRADE-CERQual Interactive Summary of Qualitative Findings [43]. It is noteworthy that the evaluation of methodological limitations of the included studies was not used to exclude studies from the review, but we did consider them in the assessment of confidence for the review findings. After evaluating each of the four components, we made a judgement on the overall confidence in each review finding; all findings started at ‘high confidence’ and then were graded down to ‘medium,’, ‘low’ or ‘very low’ confidence based on any concerns arising from the CERQual components [44].

## Results

### Results of the search and screening

**Fig 1:**
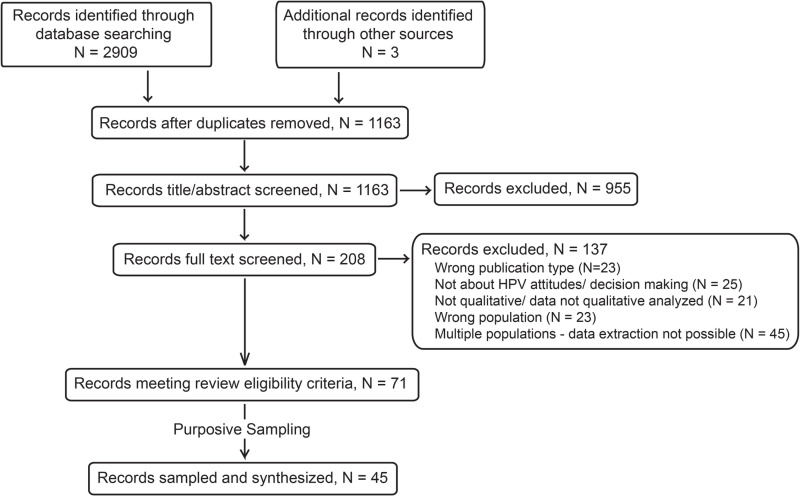
Flow diagram of study selection procedures.

We identified 1163 titles from the search of seven databases and considered 208 articles for full text review. We identified 71 articles representing 68 studies that met the review eligibility criteria. After applying purposive sampling, 45 articles representing 42 studies were included and synthesized in the review. There were 3 pairs of eligible articles that reported results from the same primary study; all three pairs were purposively sampled. The results of the search, screening, and sampling process are detailed in the PRISMA flow diagram (Fig 1).

### Description of eligible studies

This section details the study characteristics for all 68 eligible studies that met review eligibility criteria to help characterize the state of the existing qualitative literature related to HPV vaccination among young adults. Table 1 details the study characteristics of each eligible study.

**Table 1:** Summary of study characteristics of eligible studies.

Author & Year	Study Objective	Country	Study Setting	Design & Data Collection	Theoretical Framework	Eligible Participants	Sample Size	Purposive Sampling
Allen 2009 [45]	“The purpose of this study was to gain a better understanding of men’s knowledge and attitudes toward the HPV vaccine, to inform the development of effective population-based intervention strategies.”	United States	School	Qualitative, Focus Groups	None Indicated	Students 18–22 years of age (n=45)	45	Yes
Al-Naggar 2010 [46]	“The objective of this study is to explore the perceptions and opinions of young women about human papilloma virus (HPV) vaccination and associated barriers”	Malaysia	School	Qualitative, Interviews	None Indicated	female students from different faculties [departments]	30	Yes
Apaydin 2018 [47]	“The aim of this study was to qualitatively identify patient-, provider-, and systems-level barriers and facilitators for HPV vaccination among sexual and gender minority (SGM) people.”	United States	Community	Mixed Methods, Focus Groups and questionnaire	None Indicated	Sexual and gender minority (SGM) people	15	Yes
Basnyat 2018 [48]	“This study examined the means by which young Singaporean women seek and process information about HPV vaccination in their decision to become vaccinated.”	Singapore	Not Specified/ Uncertain	Qualitative, Interviews	Elaboration Likelihood Model	Singaporean females aged 18–26 years, having taken at least one shot of any of the two approved HPV vaccines in Singapore at any time in their lives	26	Yes
Bunton 2013 [49]	“This article reports on the first generation of young Australian women to participate in the mass HPV vaccination programme; what they knew about vaccination, why they participated, and how they reflect upon their experience”	Australia	Not Specified/ Uncertain	Qualitative, Focus Groups	Grounded Theory	“1) Women aged 35 years and older, whose experience solely involved Pap smears 2) Women aged 18 years who had completed the final year of their secondary education in 2008 and been offered the HPV vaccine as part of the school based catch-up programme; and 3) Women aged 20-26 who were eligible for free vaccination under the community-based catchup programme”	46	No
Carnegie 2017 [50]	“To examine cultural barriers and participant solutions regarding acceptance and uptake of the human papillomavirus (HPV) vaccine from the perspective of Black African, White-Caribbean, Arab, Indian, Bangladeshi and Pakistani young people.”	United Kingdom	Not Specified/ Uncertain	Qualitative, focus groups and interviews	Discursive Psychology	Aged 16–26 years	40	No
Chan 2011 [51]	“To identify the perception on human papillomavirus (HPV) vaccination among female nursing students in Hong Kong”	China	School	Qualitative, focus groups and interviews	None Indicated	Local female nursing students were recruited in a university in Hong Kong.	28	Yes
Chen 2021 [52]	“This qualitative research explored Chinese college students’ HPV-related awareness, knowledge, attitudes and beliefs, and their vaccination intention as well as the strategies promoting vaccination in China.”	China	School	Qualitative, Focus Groups	Health Belief Model	An individual who was (1) a student at a university in China; (2) aged 18 and older; and (3) able to read, write, and speak Chinese.	38	Yes
Clevenger 2012 [53]	“The study’s primary objective was to examine access to and knowledge of the HPV vaccine among Latina university students in Denver, Colorado”	United States	School	Qualitative, Interviews	None Indicated	Students between 19 and 26 years of age who self-identified as Latina were recruited to participate in interviews	15	Yes
Cohen 2013 [54]	“To examine differences in knowledge, attitudes, and related practices among adopters and nonadopters of the human papillomavirus (HPV) vaccine”	United States	School	Qualitative, Interviews	Diffusion of Innovation	Participants spoke English, were enrolled as a student from a large state university and were at least 18 years old but not more than 26 years old.	83	Yes
Dai 2020 [55]	“In this piece, [the author] presents a personal intercultural medical story about her HPV vaccine process. She utilizes personal narratives to share with readers her experience with sex education and to convey people’s attitudes toward sexuality and sex education in mainland China.”	United States	Not Specified/ Uncertain	Qualitative, Autoethnography	None Indicated	A woman born and raised in China, had no knowledge of HPV or the HPV vaccine until she came to the United States for graduate school	1	Yes
DeLauer 2020 [56]	“To identify knowledge and beliefs about the human papillomavirus (HPV) among students in a residential academic institution, including perceptions of safety of the HPV vaccine, perceptions of cancer correlation with HPV, and independence/interdependence in health decision-making.”	United States	School	Qualitative, Interviews	None Indicated		52	Unclear/Not Specified
Fields 2022 [57]	“The current study examined individual and structural motivators and barriers to HPV vaccination among medically underserved women utilizing a Planned Parenthood health center in Southeast Pennsylvania”	United States	Community	Qualitative, Interviews	Narrative Engagement Theory	Young adult (18–34) women attending a PPSP health center located in a suburb of Philadelphia, PA, USA, in the summer months between 18 June to 16 July 2015.	24	No
Fontenot 2016 [58]	“The purpose of this study was to elicit YMSM’s beliefs about HPV and the HPV vaccine as well as describe perceived barriers and facilitators of vaccine initiation and completion”	United States	Not Specified/ Uncertain	Qualitative, focus groups and interviews	None Indicated	Young MSM ages 18–26 years who were able to read and understand English	34	Yes
Garcia 2023 [59]	“The objective was to investigate what HPV messages unvaccinated MA patients receive and relay to others at the individual, interpersonal, and community levels; how they ascribe meaning to these messages; and how these messages affected their intentions to vaccinate”	United States	Not Specified/ Uncertain	Qualitative, Interviews	NIHMD Theoretical Framework	Female, aged 18–26 at the time of the interview, self-identify as MA, lived in California for the past 3 years, offered the HPV vaccine by a provider in adulthood (18 years or older), and not yet initiated the HPV vaccine	30	Yes
Gerend 2019 [60]	“The purpose of this study was to identify young sexual minority men’s perspectives on HPV vaccination.”	United States	Community	Qualitative, Interviews	Information, Motivation, and Behavioral Skills Model (IMB)	Eligibility criteria were assigned male sex at birth; male gender identity; ages 18–26 years; self-identify as gay, bisexual, or queer; and currently live in the Chicago metro area.	29	Yes
Glenn 2021 [61]	“The purpose of this study therefore was to explore factors influencing HPV vaccine decision-making and vaccine receipt among college students in the catch-up age range, drawing on perspectives from students, healthcare providers, and staff at a large student health center”	United States	School	Qualitative, focus groups and interviews	Deductive Approach	Students between the ages of 18 and 26 years and receiving care at the student health center	51	No
Gray Brunton 2014 [62]	“Our aim was to explore meanings and/or vaccination experiences of the HPV vaccine amongst young women aged 18–26 years in four European countries with different vaccine implementation programmes: Scotland, Spain, Serbia and Bulgaria”	Multiple Countries (Bulgaria, Scotland, Serbia, Spain)	Not Specified/ Uncertain	Qualitative, Focus Groups	None Indicated	Young women were between 18 and 26 years old.	54	Yes
Head 2012 [63]	“For the present study, we sought to understand young adult women’s perceptions of HPV, cervical cancer, the HPV vaccine, and Pap testing by conducting formative research to support a targeted health communication intervention to increase HPV vaccine uptake and regular Pap testing among young women in rural Appalachian Kentucky, with a special focus on the eight-county KR-ADD”	United States	Not Specified/ Uncertain	Qualitative, Interviews	Integrated Behavioral Model (IBM)	Eligible participants were women between the ages of 18 and 26 years who had received or had decided not to receive the HPV vaccine, and currently or recently resided in one of 29 eastern Kentucky counties or received medical care from the large KR-ADD medical clinic (serving the 29 counties)	19	Yes
Hirth 2018 [64]	“The purpose of this study was to evaluate motivations and barriers among community college students 18?26 years of age.”	United States	School	Qualitative, Interviews	Theory of Planned Behavior	Students 18–26 years old that were currently enrolled in the community college	19	Yes
Hodge 2011 [65]	“This article reports on American Indian(AI) university students’ HPV vaccine readiness and female vaccine decision-making.”	United States	School	Qualitative, Focus Groups	None Indicated	Eligibility criteria included: 1) self-identified as AI; 2) aged 18–26;and 3) currently enrolled college student.	57	Yes
Hodge 2014 [66]	“This paper reports on a preliminary exploration of HPV among an important and often overlooked at risk subgroup, American Indian males, and the unique issues and barriers they experience, as compared to female participants”	United States	School	Qualitative, Focus Groups	None Indicated	Inclusion criteria included age (> 18 years), enrolled college student status, and self-identification as American Indian	57	Yes
Hopfer 2011 [67]	“The goal of this study was to better understand college women’s HPV vaccine attitudes and beliefs with a lens focused on (a) the family, peer, and health care provider messages that college women report receiving about HPV, and (b) how college women interpret, respond to, and incorporate these messages to shape their own HPV vaccine attitudes and decisions”	United States	School	Qualitative, Interviews	Culture-centric Narrative	College women	36	Yes
Hopfer 2017 [68]	“The main purpose of this study was to elicit HPV vaccine decision narratives to understand how cultural values and implicit as well as explicit attitudes shape vaccine decision making among Latina and Vietnamese women attending Planned Parenthood health centers”	United States	Not Specified/ Uncertain	Qualitative, Interviews	Narrative Communication Theory	Women ages 18–26 years, Vietnamese, or Latina	50	No
Jaiswal 2020 [69]	“The objective of this study was to elucidate the nature and depth of (a) HPV and HPV vaccine knowledge and (b) provider communication about HPV vaccine, in a diverse sample of young urban sexual minority men.”	United States	Community	Qualitative, Interviews	None Indicated	“The parent study recruited individuals who, at baseline of Wave 2, were 22-23 years old, were assigned male at birth, had sex with a man in the previous 6 months, reported a negative or unknown HIV serostatus, and lived in the New York City metropolitan region.”	38	Yes
Jin 2023 [70]	“This study examined factors associated with HPV vaccination among college students in the Mid-South of the U.S. using a mixed-methods approach.”	United States	Community	Mixed Methods, interview and questionnaire	None Indicated	“Students who were aged 18 to 26 years and registered for an undergraduate program at the university at the time of the survey. Those eligible for the interviews were the participants who had completed the survey and were not up to date with the vaccination series”	417	Yes
Joseph 2014 [71]	“The purpose of this study was to examine how the knowledge, attitudes, and beliefs related to HPV disease and HPV vaccination affect vaccine uptake among young adult African-American, Haitian, Latina, and White women aged 18.”	United States	Clinical	Mixed Methods, interview and questionnaire	Grounded Theory	Ages 18–22 and self-identified as White, African-American, Latina or Haitian (U.S.-born or immigrants); excluded if they were pregnant or had received HPV vaccination	132	Yes
Kim 2017 [72]	“This study aimed to explore Koran American Female students’ awareness of and attitudes toward HPV vaccination”	United States	School	Qualitative, Focus Groups	Theory of Planned Behavior	Korean undergraduate students or female graduate students aged 18–26 years living in Massachusetts	20	Yes
Koskan 2018 [73]	“This study explores HIV-positive gay and bisexual men’s (GBM) understanding of human papillomavirus (HPV) and the HPV vaccine.”	United States	Community	Qualitative, Interviews	None Indicated	HIV-positive, self-identify as gay or bisexual men [transgender populations excluded] age 18 years and older, English or Spanish fluency, reside in Miami-Dade County).	15	No
Lee 2007 [74]	“To assess the knowledge and beliefs on cervical cancer and HPV infection and to evaluate the acceptability of HPV vaccination among Chinese women”	China	Not Specified/ Uncertain	Qualitative, Focus Groups	None Indicated	Healthy ethnic Hong Kong Chinese women aged 18 years and older	49	No
Lim 2019 [75]	“To explore the facilitators and barriers of HPV vaccination in young females aged 18–26 years in Singapore, and to describe their recommended strategies to improve the uptake of HPV vaccination”	Singapore	School	Qualitative, focus groups and interviews	Deductive Approach	Female students, aged 18–26 years old studying at the National University of Singapore (NUS)	40	Yes
Mancuso 2010 [76]	“learn more about how young women make decisions about HPV vaccination”	Canada	Not Specified/ Uncertain	Qualitative, Interviews	None Indicated	Five young women between the ages of 18 and 26	5	Yes
Martin 2011 [77]	“The aim of this small-scale study is to explore the impact of the introduction of HPV vaccine on attitudes towards HPV, cervical cancer and sexual risk-taking amongst university students aged 20 to 24 years”	United Kingdom	Not Specified/ Uncertain	Qualitative, Focus Groups	None Indicated	Participants were male and female students, aged 20–24 and students at the University of Leeds	34	Yes
McClelland 2006 [78]	“This paper explores knowledge of and attitudes toward sexually transmissible infections, human papillomavirus (HPV) vaccination and vaccine acceptability among young people in Australia.”	Australia	Community	Qualitative, Interviews	None Indicated	The inclusion criterion in relation to participants? Age was determined according to the target population of the current vaccination trials (18?23 years) because men and women often start their sexual lives at this age	14	Yes
McComb 2018 [79]	“The purpose of this study was to explore knowledge, attitudes and barriers regarding the HPV vaccine in this population.”	Canada	Not Specified/ Uncertain	Qualitative, Interviews	Health Belief Model	Eligibility criteria included women who were born in a country other than Canada, without a prior history of vaccination against HPV and who were proficient in English	11	Yes
Mehta 2013 [80]	“The purpose of this study was to determine predictors of HPV vaccine acceptability among college-aged men”	United States	School	Qualitative, Focus Groups	Health Belief Model	Eligibility requirements for enrollment were: English speaking males, 18 years or older, attending the university of interest. The upper age limit for participation was 25 years	50	Yes
Miller-Day 2023 [81]	“The purpose of the current project is to first identify elements of young men’s vaccine decision narratives (phase one) and then translate these into prevention messages for a “Men’s Stories” (MS) narrative-based HPV video intervention (phase two).”	United States	Not Specified/ Uncertain	Qualitative, Interviews	Narrative Engagement Theory	Self-identify as male, age 18–26, English speaking, and self-identify as heterosexual.	15	Yes
Mills 2013 [82]	“To address women’s reasons for declining the HPV vaccine and, among women who initiated the vaccine series, barriers to completion of the 3-dose regimen”	United States	Not Specified/ Uncertain	Qualitative, Interviews	Integrated Behavioral Model (IBM)	Female clinic patients aged 18–26, who had either declined the HPV vaccine or had started the vaccine series but failed to complete doses 2, 3, or both in the required time	17	Yes
Morales-Campos 2021 [83]	To evaluate “(1) What are the gendered beliefs and attitudes around cervical cancer, HPV and HPV vaccination among Mexican and Mexican American men and women living along the Texas-Mexico Border? And (2) What are similarities/differences between mothers and fathers beliefs who have HPV vaccine-eligible daughters?”	United States	Not Specified/ Uncertain	Qualitative, Focus Groups	None Indicated	Being a Hispanic man or woman, aged 18 or older, a parent of a girl aged 11–17 (only for mother and father groups), and residing in Hidalgo or Cameron counties.	71	No
Mortensen 2010 [84]	“Investigate the reasons for young women’s acceptance or rejection of the quadrivalent HPV vaccine after its general availability in Denmark”	Denmark	Not Specified/ Uncertain	Qualitative, focus groups and interviews	None Indicated	Women aged between 16–26 years who had heard of HPV vaccination	435	No
Nadarzynski 2017 [85]	“explore the perceptions of HPV and attitudes towards HPV vaccination to inform the development of future interventions on HPV vaccination for MSM in the United Kingdom.”	United Kingdom	Not Specified/ Uncertain	Qualitative, focus groups and interviews	None Indicated	“English-speaking MSM, between 16 and 40 years old. All self-identified men, who were sexually attracted to or had already had sex with other men, were eligible for inclusion in the study”	32	No
Nkwonta 2020 [86]	“explore 1) international university students knowledge of HPV and its associated sequelae; and 2) international university students attitudes toward and uptake of HPV preventive practices.”	United States	School	Mixed Methods, interview, focus group, and questionnaire	Health Belief Model	1) were 18 years or older; 2) self-reported as an international university student; and 3) had spent at least a semester in the US.	81	No
Pčolkina 2019 [87]	“We explored knowledge, behaviors, and attitudes toward CC prevention strategies in Latvian women”	Latvia	Clinical	Mixed Methods, interview and questionnaire	None Indicated	Latvian women in Riga age 20 and older	158	No
Petrova 2015 [88]	“explore young women’s experiences with risk information about HPV and the HPV vaccine. We explored how women evaluated the quantity and quality of HPV-related information and information sources they consulted, and how this information affected their decisions about the HPV vaccine”	Multiple Countries (Bulgaria, Scotland, Serbia, Spain)	School	Qualitative, Focus Groups	None Indicated	Women between 18 and 26 years old	54	Yes
Pierre Joseph 2014 [89]	“To examine the attitudes toward human papillomavirus (HPV) vaccination among young men from African American, Haitian, Caucasian, and Latino backgrounds”	United States	Community	Qualitative, Interviews	Health Belief Model	89 men (31 black, 26 Haitian, 18 Latinos and 14 Caucasian), average age 19	89	Yes
Pierre-Victor 2017 [90]	“This study investigated the role of healthcare providers’ Recommendation style in Haitian parents’ and female patients’ HPV vaccine decision-making”	United States	School	Qualitative, Interviews	None Indicated	Self-identifying as Haitian, female, and being between 17 and 26 years of age.	30	No
Power 2009 [91]	“explore how lesbian and bisexual women perceive their level of risk for HPV, their willingness to accept the HPV vaccine, the reasons why they do or do not feel at risk and how they manage their sexual health in relation to their identity as a non-heterosexual woman”	Australia	School	Mixed Methods, interview and questionnaire	Symbolic Interactionist Theory	Lesbian and bisexual women and a comparison group of heterosexual women	352	No
Pratt 2019 [92]	“This study sought to understand the views of Somali young adults regarding HPV immunization.”	Somalia	Clinical	Qualitative, Focus Groups	None Indicated	Somali and between 18 and 26 years of age	34	Yes
Prokopovich 2023 [93]	“explore how one culturally and linguistically diverse (CALD) community engages with school and HPV vaccination”	Australia	Not Specified/ Uncertain	Qualitative, Focus Groups	None Indicated	“(I) parents or grandparents of a child/grandchild aged 7-24 years old (those who have consented or will be approached to consent for school vaccination); and (ii) young adults aged 18?24 years old who had been exposed to their local school vaccination programme.”	31	No
Rahim 2023 [94]	“The objective of this study was to assess vaccination rates in both populations at our own institution as well as understand AYA and caregiver’s beliefs regarding HPV vaccination and vaccine hesitancy, which may contribute to the lower HPV vaccination rates in these high-risk patient populations.”	United States	Clinical	Mixed Methods, Retrospective chart review and interview	None Indicated	Caregivers of patients 9–21 years of age and patients who were 18–21 years old who presented to either the comprehensive SCD clinic or the oncology survivorship clinic from March 2021 to July 2021	69	No
Reiter 2014 [95]	“The current study collected data from four key stakeholder groups from Appalachian communities to examine their acceptability of HPV vaccine for males and potential barriers to vaccinating males against HPV in their communities.”	United States	Not Specified/ Uncertain	Qualitative, focus groups and interviews	None Indicated	(A) parents with adolescent sons ages 9–17 years, (b) young adult men ages 18–26 years (c) health care providers, and (d) community leaders.	102	No
Ross 2010 [96]	“We undertook a qualitative study among first year university students as a first step towards developing a model that explains pathways to vaccine acceptance or rejection amongst older teenagers.”	United Kingdom	School	Qualitative, Focus Groups	Multiple Theories (Health Belief model, Social Cognitive Model)	“female first year undergraduates who resided permanently in the UK and were therefore eligible for HPV vaccination before they came to University. Undergraduates studying nursing and medicine were not eligible for the study.”	0	Yes
Salad 2015 [97]	“aims to explore the perceptions of Somali women living in the Netherlands regarding measures to prevent cervical cancer”	Netherlands	Not Specified/ Uncertain	Qualitative, focus groups and interviews	Multiple Theories (Health Belief model, intersectionality framework)	“being female and of Somali origin, living in the Netherlands, aged between 18 and 65, and having a migration date from the first or second wave of migration”	20	No
Schmidt-Grimminger 2013 [98]	“We assessed HPV knowledge, attitudes, and beliefs towards HPV and HPV vaccination during a community-based participatory research project among tribal youth, young adults, parents, and health professionals”	United States	Not Specified/ Uncertain	Mixed Methods, focus group and questionnaire	CBPR Framework	Young adult women ages 19–26 (n=22).	73	No
Schwendener 2022 [99]	“We aimed to provide a detailed characterisation of human papillomavirus (HPV) vaccine awareness, knowledge and information sources in the HPV vaccine decision-making process of youth, both male and female, in Switzerland.”	Switzerland	Not Specified/ Uncertain	Mixed Methods, interview and questionnaire	None Indicated	Participants were 15–26 years of age, male and female	997	Unclear/Not Specified
Shetty 2021 [100]	“The purpose of this exploratory study is to evaluate undergraduate student understanding towards barriers and facilitators of HPV vaccination and identify potential health education opportunities for HPV vaccination.”	India	School	Qualitative, Focus Groups	None Indicated	“students aged 18–26 years enrolled in medical, dental, nursing, and MLT degree courses at KSHEMA who previously participated in a previously published quantitative study”	20	Yes
Siu 2013 [101]	“This paper investigates, using a qualitative approach, barriers to receiving Human Papillomavirus (HPV) vaccine among female undergraduate students in a Hong Kong university.”	China	School	Qualitative, Interviews	None Indicated	(1) female undergraduate students, excluding medical and health sciences students, (2) no experience of sexual intercourse, (3) have not yet received an HPV vaccination and (4) Hong Kong Chinese by ethnicity	35	Yes
Stephens 2014 [102]	“assess current and preferred social networks that influence human papillomavirus(HPV) vaccine decision-making in a sample of Hispanic college women.”	United States	School	Qualitative, Interviews	Social Network Theory	Criteria included self-identifying as Hispanic, female, and between 18 and 24 years of age	41	Yes
Stephens 2016 [103]	“To identify factors influencing human papillomavirus (HPV) vaccination up taking decision making among vaccinated and non-vaccinated Hispanic college women.”	United States	School	Qualitative, Interviews	None Indicated	Hispanic undergraduate women aged 18–24	49	Yes
Sullivan-Blum 2022 [104]	“This study aimed to determine the attitudes, beliefs and barriers of primary care patients on prep towards the HPV vaccine.”	United States	Clinical	Qualitative, Interviews	None Indicated	Status as a prep patient in the two family medicine clinics	16	No
Teitelman 2018 [105]	“the purpose of this article is to identify salient beliefs about HPV vaccine completion among young adult women who live in economically disadvantaged urban communities and describe the integration of those beliefs into the development of an mhealth application using theory-based research and user-centered design to promote vaccine completion.”	United States	Community	Mixed Methods, Survey/Questionnaire	Integrated Behavioral Model (IBM)	“women between 18 and 26 years of age who received the first dose of the HPV vaccine at the current clinic visit, had ever had vaginal sex, and owned a smartphone.”	35	Yes
Thompson 2017 [106]	“The purpose of this study was to understand how relationship status and vaccination status impact risk perceptions and perceived need for the HPV vaccine among young adult women.”	United States	School	Mixed Methods, interview and questionnaire	None Indicated	“Women were eligible for the study if they met the following criteria: 1) university student, 2) between 18 and 26 years of age, 3) has not received any doses of the HPV vaccine or has received the first dose of the HPV vaccination series in the last 6 months, 4) speaks English, and 5) provides informed consent.”	50	Yes
Thompson 2018 [107]	“The purpose of this study was to elicit the information needs, motivations, and behavioral skills related to HPV vaccine decision-making among young adult women by comparing these factors between unvaccinated and recently vaccinated women.”	United States	School	Mixed Methods, interview and questionnaire	Information, Motivation, and Behavioral Skills Model (IMB)	(1) 18–26 years of age, (2) college student, (3) had not received the HPV vaccine or received the first dose of the HPV vaccine series in the last 6 months, and (4) English speaking	50	Yes
Tu 2013 [108]	“To enhance understanding of young women’s experiences of human papillomavirus-related cervical cancer prevention in Taiwan”	Taiwan	School	Qualitative, Interviews	None Indicated	“The college women participants met the criteria below: (1) above 20 years old with sexual experience and never been married and/or pregnant, (2) heard about HPV and (3) were willing to share their HPV-related cervical cancer prevention experiences”	24	Yes
Waters 2022 [109]	“We sought to describe participants’ experiences with the HPV vaccine”	United States	Clinical	Mixed Methods, interview and questionnaire	None Indicated	“Eligible participants were either an HPV vaccine eligible cancer survivor (18–26 years) or a caregiver of a younger HPV vaccine eligible cancer survivor (9-17 years). Eligible survivors had completed treatment and received care during 2013-2018. Eligible caregivers were at least 18 years of age and the caregiver of a survivor under 18 years of age who had completed treatment at PCH between 2013 and 2018”	20	No
Wheldon 2017 [110]	“The purpose of this study was to (1) describe salient beliefs related to HPV vaccination among young MSM; (2) determine factors that underlie these beliefs; and (3) describe a model for HPV vaccine decision-making.”	United States	Community	Qualitative, Interviews	Integrative Model of Behavioral Prediction (IM)	MSM between the ages of 18 and 26 years who may or may not identify as gay or bisexual	22	Yes
Wong 2008 [111]	“To investigate the acceptability of the HPV vaccine among a multiethnic sample of young women in Malaysia”	Malaysia	Not Specified/ Uncertain	Qualitative, Focus Groups	None Indicated	Young unmarried Malaysian women aged between 13 and 27 years.	40	No
Wong 2009 [112]	“This qualitative study used focus group discussions (FGDs) to evaluate information needed in order to make informed human papillomavirus (HPV) vaccination decision, opinion on the most acceptable public education messages, and channel of delivery in a multiethnic, multicultural and multireligion country.”	Malaysia	Community	Qualitative, Focus Groups	Grounded Theory	Mothers of eligible vaccinees, young women eligible for the vaccine aged between 18 and 26 years old, and men (single men and fathers of eligible vaccinees).	114	No
Wyndham-West 2016 [113]	“In this article I explore the limitation of the public health account of decision-making, grounded in individual cost/benefit rationality and explore the ways in which for these students vaccination decisions were shaped by social context and identity issues”	Canada	School	Qualitative, Interviews	None Indicated	Ontario-based women university students who were up to 26 years of age.	24	Yes
Yarmohammadi 2022 [114]	“this study aimed to determine strategies for improving HPV vaccination among young adults”	Iran	Community	Qualitative, Interviews	None Indicated	(1) Tehran native young adult participant, (2) 18–26 years old, and (3) having information about HPV (both young adult participants and health professionals)	30	No
Young 2018 [115]	“The objective of this study is to determine the knowledge, understanding and concerns that young women have about HPV when attending colposcopy and whether their information needs are met.”	United Kingdom	Clinical	Qualitative, Interviews	None indicated	“all English-speaking women born after 1/9/1990 with abnormal cervical cytology and who had been referred to colposcopy at a regional colposcopy clinic were eligible to take part”	15	Yes

#### Study design and data collection.

Most studies (n=56) were qualitative, utilizing focus groups and/or interview data collection methods; one study was an autoethnography. Twelve (12) studies were mixed methods design, utilizing questionnaires in combination with interviews and/or focus groups; one study utilized retrospective chart review and interviews. Data collection was conducted between 2006 and 2021.

#### Theoretical frameworks & Conceptual models.

Nineteen (19) theoretical frameworks and conceptual models were utilized across the eligible articles. The Health Belief Model was the most common theory cited (7 studies). Thirty-eight (38) articles did not cite use of a theory or model.

#### Study setting and country.

Twenty-six (26) studies cited the study was conducted in a school setting, 12 in a community setting and 7 in a clinical setting; 23 studies did not specify a study setting. Across the eligible articles, 19 countries spanning 5 continents were represented; according to the World Bank classification of economies [116], 13 were high income countries (Australia, Canada, Bulgaria, Denmark, Latvia, Netherlands, Scotland, Singapore, Spain, Switzerland, Taiwan, United Kingdom, United States); 5 were middle income countries (China, India, Iran, Malaysia, Serbia); and 1 was a low-income country (Somalia). Most articles presented studies conducted in North America (41 studies), majority of which were in the United States (38 studies); 12 studies were conducted in Asia, 10 in Europe, 4 in Oceania, and only one 1 study was conducted in Africa. One study spanned multiple countries in Europe (Bulgaria, Scotland, Serbia, and Spain).

#### Study participants.

There were a total of 4788 participants across the eligible studies. Fifty-five (55) studies sampled women/females, 30 sampled men/males, and 7 sampled sexual and gender minority (SGM) individuals. College students were represented in 16 studies, parents were sampled in 4 studies, healthcare professionals were included in 4 studies, caregivers sampled in 2 studies, and community leaders were included in 1 study.

Race and ethnicity categories were not uniformly reported across articles. We present them below as they were reported in the manuscripts, acknowledging that some labels may seem duplicitous/refer to the same classification of race/ethnicity: 21 studies included White/Caucasian individuals; 4 studies included Non-Hispanic White individuals; 1 study included Non-Hispanic/Latinx individuals; 2 studies included Hispanic White individuals; 20 studies included Hispanic/Latinx individuals; 1 study included Mexican American individuals; 20 studies included Black/African American individuals; 12 studies included Asian individuals; 1 study included Asian American individuals; 3 studies included Native American/American Indian individuals; 5 studies included multiracial/multiethnic individuals; and 12 studies cited individuals with other/unknown race and ethnicity.

#### Study publication.

Study manuscripts were published between 2006 and 2023

Table 2 provides a summarized comparison of the study characteristics between the 68 studies that met review eligibility and the 42 studies that were included in the review after purposive sampling.

**Table 2:** Study Characteristics Comparison Between Eligible and Included Studies.

	Eligible Studies	Included Studies
**# Studies (articles)**	68 studies (71 articles)	42 studies (45 articles)
**# Participants**	4788	1790
**Study Design**	Qualitative: 56	Qualitative: 37
	Mixed Methods: 12	Mixed Methods: 5
**Data Collection**	Between 2006 and 2021	Between 2007 and 2021
**Study Setting**	Clinical: 7	Clinical: 3
	Community: 12	Community: 8
	School: 26	School: 21
	Not specified: 23	Not specified: 10
**Continent & Country**	5 continents & 19 countries	5 continents & 14 countries
	Africa (1): Somalia (1)	Africa (1): Somalia (1)
	Asia (12): China (4), India (1), Iran (1), Malaysia (3), Singapore (2), Taiwan (1)	Asia (8): China (3), India (1), Malaysia (1), Singapore (2), Taiwan (1)
	Europe (10): Denmark (1), Latvia (1), Netherlands (1), Switzerland (1), United Kingdom (5); Multiple countries (1)	Europe (4): United Kingdom (3); Multiple countries (1)
	North America (41): Canada (3), United States (38)	North America (28): Canada (3), United States (25)
	Oceania(4): Australia (4)	Oceania(1): Australia (1)
**Economy Classification**	High income: 13 countries	High income: 9 countries
	Middle-income: 5 countries	Middle-income: 4 countries
	Low-income: 1 country	Low-income: 1 country
**Study Participants: groups**	Women: 55	Women: 34
	Men: 30	Men: 17
	SGM: 7	SGM: 3
	College students: 16	College students: 14
	Parents: 4	
	Healthcare professionals: 4	
	Caregivers: 2	
	Community leaders: 1	
**Study Participants: Race and Ethnicity (as reported from the articles; not mutually exclusive)**	White/Caucasian: 21	White/Caucasian: 15
	Non-Hispanic White: 4	Non-Hispanic White: 1
	Non-Hispanic/Latinx: 1	Non-Hispanic/Latinx: 1
	Hispanic White: 2	Hispanic White: 1
	Hispanic/Latinx: 20	Hispanic/Latinx: 15
	Mexican American: 1	Mexican American: 1
	Black/African American: 20	Black/African American: 16
	Asian: 12	Asian: 8
	Asian American: 1	Asian American: 1
	Native American: 3	Native American: 2
	Multiracial/Multiethnic: 5	Multiracial/Multiethnic: 3
	Other/Unknown: 12	Other/Unknown: 8
**Article Publication**	Between 2006 and 2023	Between 2006 and 2023

### Methodological quality of included studies

Across the 42 studies that were purposively sampled and included in the review, the majority (35 studies represented by 37 articles) had an overall assessment of minor to moderate quality (Table 3) The most common issue across the studies was poor or no reporting of researcher reflexivity (36 studies). It is worth noting that the requirement for reflexivity in reporting guidelines for qualitive research may have been implemented after some of the studies were published. Additionally, three studies did not provide details on data analysis procedures and failed to take ethical issues into consideration (no mention of informed consent or IRB approval) [46,55,76]. Of the 42 included studies, only two studies had severe concerns on methodological quality [46,55].

**Table 3:** Methodological limitations of included studies.

Study ID	Was there a clear statement of the aims of the research?	Are the setting(s) and context described adequately?	Is the sampling strategy described, and is this appropriate?	Is the sampling strategy described, and is this appropriate?	Is the data analysis described, and is this appropriate?	Is there evidence of reflexivity?	Have ethical issues been taken into consideration?	Are the claims made/findings supported by sufficient evidence?	Overall assessment
Allen 2009	Yes	Yes	Can’t Tell	Yes	Yes	No	Yes	Yes	Moderate
Al-Naggar 2010	Yes	Yes	Yes	Yes	No	No	No	Yes	Severe
Apaydin 2018	Yes	Yes	Yes	Yes	Yes	No	Yes	Yes	Minor to moderate
Basnyat 2018	Yes	Yes	Yes	Yes	Yes	No	Yes	Yes	Minor to moderate
Chan 2011	Yes	Yes	Yes	Yes	Yes	Can’t Tell	Yes	Yes	Minor to moderate
Chen 2021	Yes	Yes	Yes	Yes	Yes	No	Yes	Yes	Minor to moderate
Clevenger 2012	Yes	Yes	Yes	Yes	Yes	No	Yes	Yes	Minor to moderate
Cohen 2013	Yes	Yes	Yes	Yes	Yes	Can’t Tell	Yes	Yes	Minor to moderate
Dai 2020	No	Yes	No	No	No	Yes	No	Yes	Severe
Fontenot 2016	Yes	Yes	Yes	Yes	Yes	No	Yes	Yes	Minor to moderate
Garcia 2023	Yes	Yes	Yes	Yes	Yes	No	Yes	Yes	Minor to moderate
Gerend 2019	Yes	Yes	Yes	Yes	Yes	No	Yes	Yes	Minor to moderate
Gray Brunton 2014	Yes	Yes	Yes	Yes	Yes	No	Yes	Yes	Minor to moderate
Head 2012	Yes	Yes	Yes	Yes	Yes	No	Yes	Yes	Minor to moderate
Hirth 2018	Yes	Yes	Yes	Yes	Yes	No	Yes	Yes	Minor to moderate
Hodge 2011	Yes	Yes	Yes	Yes	Yes	Yes	Yes	Yes	Minor
Hodge 2014	Yes	Yes	Yes	Yes	Yes	No	Yes	Yes	Minor to moderate
Hopfer 2011	Yes	Yes	Yes	Yes	Yes	Yes	Yes	Yes	Minor
Jaiswal 2020	Yes	Yes	Yes	Yes	Yes	No	Yes	Yes	Minor to moderate
Jin 2023	Yes	Yes	Yes	Yes	Yes	No	Yes	Yes	Minor to moderate
Joseph 2014	Yes	Yes	Yes	Yes	Yes	No	Yes	Yes	Minor to moderate
Kim 2017	Yes	Yes	Yes	Yes	Yes	No	Yes	Yes	Minor to moderate
Lim 2019	Yes	Yes	Yes	Yes	Yes	Yes	Yes	Yes	Minor
Mancuso 2010	Yes	Yes	Yes	Yes	No	No	No	Yes	Moderate to severe
Martin 2011	Yes	Yes	Yes	Yes	Yes	No	Yes	Yes	Minor to moderate
McClelland 2006	Yes	Yes	Yes	Yes	Yes	No	Yes	Yes	Minor to moderate
McComb 2018	Yes	Yes	Yes	Yes	Yes	No	Yes	Yes	Minor to moderate
Mehta 2013	Yes	Yes	Yes	Yes	Yes	No	Yes	Yes	Minor to moderate
Miller-Day 2023	Yes	Yes	Yes	Yes	Yes	No	Yes	Yes	Minor to moderate
Mills 2013	Yes	Yes	Yes	Yes	Yes	No	Yes	Yes	Minor to moderate
Petrova 2015	Yes	Yes	Yes	Yes	Yes	No	Yes	Yes	Minor to moderate
Pierre Joseph 2014	Yes	Yes	Yes	Yes	Yes	No	Yes	Yes	Minor to moderate
Pratt 2019	Yes	Yes	Yes	Yes	Yes	No	Yes	Yes	Minor to moderate
Ross 2010	Yes	Yes	Yes	Yes	Yes	No	Yes	Yes	Minor to moderate
Shetty 2021	Yes	Yes	Yes	Yes	Yes	No	Yes	Yes	Minor to moderate
Siu 2013	Yes	Yes	Yes	Yes	Yes	No	Yes	Yes	Minor to moderate
Stephens 2014	Yes	Yes	Yes	Yes	Yes	No	Yes	Yes	Minor to moderate
Stephens 2016	Yes	Yes	Yes	Yes	Yes	Yes	Yes	Yes	Minor
Teitelman 2018	Yes	Yes	Yes	Yes	Yes	No	Yes	Yes	Minor to moderate
Thompson 2017	Yes	Yes	Yes	Yes	Yes	No	Yes	Yes	Minor to moderate
Thompson 2018	Yes	Yes	Yes	Yes	Yes	No	Yes	Yes	Minor to moderate
Tu 2013	Yes	Yes	Yes	Yes	Yes	No	Yes	Yes	Minor to moderate
Wheldon 2017	Yes	Yes	Yes	Yes	Yes	No	Yes	Yes	Minor to moderate
Wyndham-West 2016	Yes	Yes	Yes	Yes	Yes	No	Yes	Yes	Minor to moderate
Young 2018	Yes	Yes	Yes	Yes	Yes	No	Yes	Yes	Minor to moderate

### Confidence in review findings

Our QES yielded 29 review findings. We graded 11 findings as high confidence, 17 findings as moderate confidence and 1 finding as low confidence (Table 4). The GRADE CERQual evidence profile detailing our evaluation of each component for each finding and associated explanations are in S3 Appendix.

**Table 4:** Summary of qualitative finding.

Summarized review finding	GRADE-CERQual Assessment of confidence	Explanation of GRADE-CERQual Assessment	References
**THEME 1: Individual Factors**			
[Finding 1] Missed opportunity: Some young adults believed they missed the window for vaccination because they were too old to benefit from it	Moderate confidence	5 studies reflecting minor concerns regarding methodological limitations, and minor concerns regarding adequacy.	Kim 2017; Jaiswal et al. 2020; Tu & Wang 2013; Wheldon et al. 2017; Hodge et al. 2011;
[Finding 2] Risk perception: Some young adults believed they were not at risk for HPV-related illnesses or that they still had time to decide on vaccination.	Moderate confidence	7 studies reflecting minor concerns regarding methodological limitations, minor concerns regarding coherence, and minor concerns regarding adequacy.	Hodge 2014; Hopfer & Clippard 2011; Allen et al. 2009; Lim & Lim 2019; Miller-Day et al. 2023; Wheldon et al. 2017; Siu 2013;
[Finding 3] Proactive measure: Young adults viewed vaccination as a proactive measure to safeguard their health in the present and prevent future regret if they didn’t get vaccinated.	High confidence	13 studies reflecting minor concerns regarding methodological limitations.	Hopfer & Clippard 2011; Apaydin et al. 2018; Chen et al. 2021; Gerend et al. 2019; Hirth et al. 2018; Joseph et al. 2014; Pierre Joseph et al. 2014; Shetty et al. 2021; Thompson et al. 2017; Wheldon et al. 2017; Wyndham-West 2016; McClelland & Liamputtong 2006; Stephens et al. 2016;
[Finding 4] Low-stakes preventive measure: Several individuals mention the simplicity of the decision to get vaccinated, seeing it as a low-risk preventive measure with potentially significant benefits	Low confidence	5 studies reflecting minor concerns regarding methodological limitations, and moderate concerns regarding adequacy.	Kim 2017; Hirth et al. 2018; Lim & Lim 2019; Pierre Joseph et al. 2014; Cohen & Head 2013; McClelland & Liamputtong 2006;
**THEME 2: Alternatives to vaccination**			
[Finding 5] Reliance on pap smears: Young adult women believed that regular Pap testing negated the need for HPV vaccination.	Moderate confidence	4 studies reflecting moderate concerns regarding methodological limitations, and minor concerns regarding adequacy.	Mancuso & Polzer 2010; Petrova et al. 2015; Tu & Wang 2013; Hodge et al. 2011;
[Finding 6] Condom use and other precautions: Young adults expressed precautions such as condom use, abstinence, and decisions on sexual partners were sufficient to protect against HPV, rendering the vaccine unnecessary.	Moderate confidence	6 studies reflecting minor concerns regarding methodological limitations, and minor concerns regarding adequacy.	Hopfer & Clippard 2011; Miller-Day et al. 2023; Pierre Joseph et al. 2014; Thompson et al. 2017; Tu & Wang 2013; Cohen & Head 2013;
**THEME 3: Knowledge and information**			
[Finding 7] Many young adults expressed having insufficient information on HPV or the vaccine before deciding to get vaccinated. There was an expressed desire more information about HPV and the HPV vaccine.	High confidence	13 studies reflecting minor concerns regarding methodological limitations.	Hodge 2014; Kim 2017; Fontenot et al. 2016; Garcia et al. 2023; Gray Brunton et al. 2014; Hirth et al. 2018; Joseph et al. 2014; Miller-Day et al. 2023; Pierre Joseph et al. 2014; Cohen & Head 2013; Head & Cohen 2012; Hodge et al. 2011; McClelland & Liamputtong 2006;
**THEME 4: Sex and romantic relationships**			
[Finding 8] Sexual activity and behavior: Some individuals believe that vaccination against HPV is only necessary if one plans to engage in sexual activity or has many sexual partners.	High confidence	12 studies reflecting minor concerns regarding methodological limitations.	Hopfer & Clippard 2011; Garcia et al. 2023; Joseph et al. 2014; Lim & Lim 2019; Miller-Day et al. 2023; Petrova et al. 2015; Thompson et al. 2017; Wheldon et al. 2017; Cohen & Head 2013; Head & Cohen 2012; Siu 2013; Stephens et al. 2016;
[Finding 9] Relationship status and sexual history influenced risk perception among young adults.	High confidence	7 studies reflecting minor concerns regarding methodological limitations.	Hopfer & Clippard 2011; Miller-Day et al. 2023; Pierre Joseph et al. 2014; Thompson et al. 2017; Thompson et al. 2018; McClelland & Liamputtong 2006; Mehta et al. 2013;
[Finding 10] Parental beliefs about sex and the HPV vaccine influenced young adults’ vaccine attitudes and decisions.	Moderate confidence	6 studies reflecting minor concerns regarding methodological limitations, minor concerns regarding coherence, minor concerns regarding adequacy.	Hopfer & Clippard 2011; Fontenot et al. 2016; McComb et al. 2018; Shetty et al. 2021; Hodge et al. 2011; Siu 2013;
**THEME 5: Parents and peers**			
[Finding 11] Mothers more than fathers played an active role in HPV vaccination process among young adults.	High confidence	12 studies reflecting minor concerns regarding methodological limitations.	Hopfer & Clippard 2011; Kim 2017; Garcia et al. 2023; Gerend et al. 2019; Hirth et al. 2018; Lim & Lim 2019; Miller-Day et al. 2023; Basnyat & Lim 2018; Cohen & Head 2013; McClelland & Liamputtong 2006; Ross et al. 2010; Stephens & Thomas 2014;
[Finding 12] Decision-making power: There was a mix of deferred or shared the decision to get vaccinated between young adults and parents.	Moderate confidence	5 studies reflecting minor concerns regarding methodological limitations, minor concerns regarding coherence, and minor concerns regarding adequacy.	Mills et al. 2013; Hirth et al. 2018; Miller-Day et al. 2023; Cohen & Head 2013; Stephens et al. 2016;
[Finding 13] Peer influence: Friends and acquaintances played a significant role in shaping vaccination decisions.	Moderate confidence	Minor concerns regarding methodological limitations, No/Very minor concerns regarding coherence, Minor concerns regarding adequacy, and No/Very minor concerns regarding relevance	Hopfer & Clippard 2011; Thompson et al. 2018; Wheldon et al. 2017; Basnyat & Lim 2018; Cohen & Head 2013; Head & Cohen 2012; Ross et al. 2010; Siu 2013;
[Finding 14] Health history of family and friends was a motivating factor in the decision to get vaccinated.	Moderate confidence	5 studies reflecting minor concerns regarding methodological limitations and minor concerns regarding adequacy.	Chen et al. 2021; Pierre Joseph et al. 2014; Wyndham-West 2016; Head & Cohen 2012; Stephens et al. 2016;
**THEME 6: Physicians**			
[Finding 15] Doctor recommendation: Physician recommendations (positive or negative) were influential in vaccination uptake.	High confidence	18 studies reflecting minor concerns regarding methodological limitations.	Hopfer & Clippard 2011; Apaydin et al. 2018; Gerend et al. 2019; Hirth et al. 2018; Jaiswal et al. 2020; Joseph et al. 2014; McComb et al. 2018; Miller-Day et al. 2023; Pierre Joseph et al. 2014; Thompson et al. 2017; Thompson et al. 2018; Wheldon et al. 2017; Wyndham-West 2016; Chan et al. 2011; Clevenger et al. 2012; Cohen & Head 2013; Ross et al. 2010; Stephens & Thomas 2014;
[Finding 16] Communication gap with doctors: Young adults desired better communication and more informed conversations with their doctors.	High confidence	9 studies reflecting minor concerns regarding methodological limitations.	Mills et al. 2013; Apaydin et al. 2018; Fontenot et al. 2016; Garcia et al. 2023; Jaiswal et al. 2020; Petrova et al. 2015; Pierre Joseph et al. 2014; Wheldon et al. 2017; Siu 2013;
**THEME 7: Logistics**			
[Finding 17] Appointments: Scheduling and appointment challenges impacted young adults’ ability to get vaccinated.	Moderate confidence	10 studies reflecting minor concerns regarding methodological limitations and minor concerns regarding adequacy.	Mills et al. 2013; Apaydin et al. 2018; Fontenot et al. 2016; Hirth et al. 2018; Jaiswal et al. 2020; Pierre Joseph et al. 2014; Teitelman et al. 2018; Head & Cohen 2012; Stephens et al. 2016; Stephens & Thomas 2014;
[Finding 18] Multiple shots: Some young adults found the process of going back multiple times for the three doses burdensome, leading to forgetfulness or avoidance.	Moderate confidence	8 studies reflecting minor concerns regarding methodological limitations and minor concerns regarding adequacy.	Hopfer & Clippard 2011; Apaydin et al. 2018; Jaiswal et al. 2020; Lim & Lim 2019; Miller-Day et al. 2023; Teitelman et al. 2018; Head & Cohen 2012; Ross et al. 2010;
[Finding 19] Accessibility: Transportation and access challenges hindered HPV vaccination uptake.	Moderate confidence	8 studies reflecting moderate concerns regarding methodological limitations and minor concerns regarding adequacy.	Dai 2020; Hopfer & Clippard 2011; Mills et al. 2013; Hirth et al. 2018; Lim & Lim 2019; Wheldon et al. 2017; Hodge et al. 2011; Ross et al. 2010;
[Finding 20] Reminders: Reminders were cited as helpful in ensuring adherence to the vaccination schedule.	Moderate confidence	5 studies reflecting minor concerns regarding methodological limitations ad minor concerns regarding adequacy.	Hopfer & Clippard 2011; Apaydin et al. 2018; Hirth et al. 2018; Miller-Day et al. 2023; Ross et al. 2010;
[Finding 21] Mandates: Young adults had mixed views on mandates for HPV vaccination.	Moderate confidence	7 studies reflecting minor concerns regarding methodological limitations and minor concerns regarding adequacy.	Hirth et al. 2018; Joseph et al. 2014; Lim & Lim 2019; Miller-Day et al. 2023; Pierre Joseph et al. 2014; Pratt et al. 2019; Shetty et al. 2021;
**THEME 8: The Vaccine**			
[Finding 22] Vaccine cost: Young adults expressed concerns about paying for the HPV vaccine, citing high out-of-pocket costs and need for government subsidies.	High confidence	18 studies reflecting minor concerns regarding methodological limitations.	Hopfer & Clippard 2011; Chen et al. 2021; Gray Brunton et al. 2014; Hirth et al. 2018; Lim & Lim 2019; Miller-Day et al. 2023; Pierre Joseph et al. 2014; Shetty et al. 2021; Teitelman et al. 2018; Tu & Wang 2013; Wheldon et al. 2017; Chan et al. 2011; Clevenger et al. 2012; Cohen & Head 2013; Head & Cohen 2012; Hodge et al. 2011; Ross et al. 2010; Siu 2013;
[Finding 23] Side effects: Young adults expressed concerned about the side effects of the HPV vaccine.	High confidence	12 studies reflecting minor concerns regarding methodological limitations.	Kim 2017; Mancuso & Polzer 2010; Garcia et al. 2023; Hirth et al. 2018; Jaiswal et al. 2020; Joseph et al. 2014; Petrova et al. 2015; Chan et al. 2011; Cohen & Head 2013; Hodge et al. 2011; Siu 2013; Stephens et al. 2016;
[Finding 24] Understanding of vaccine effectiveness: Young adults questioned the effectiveness of the HPV vaccine given the limited strains it covers and lack of 100% protection.	Moderate confidence	9 studies reflecting minor concerns regarding methodological limitations, minor concerns regarding coherence, and minor concerns regarding adequacy.	Mancuso & Polzer 2010; Gray Brunton et al. 2014; Petrova et al. 2015; Thompson et al. 2017; Wheldon et al. 2017; Cohen & Head 2013; Hodge et al. 2011; Ross et al. 2010; Young et al. 2018;
[Finding 25] Development of the vaccine: Concerns were raised about the vaccine’s novelty, hastened development, and lack of long-term studies.	High confidence	11 studies reflecting minor concerns regarding methodological limitations.	Hodge 2014; Hopfer & Clippard 2011; Gray Brunton et al. 2014; Hirth et al. 2018; Lim & Lim 2019; Petrova et al. 2015; Tu & Wang 2013; Wyndham-West 2016; Chan et al. 2011; Cohen & Head 2013; Young et al. 2018;
[Finding 26] Pharmaceutical companies and business motive: Young adults expressed skepticism towards pharmaceutical companies and their motives, suggesting that public panic and greed may influence vaccination campaigns.	Moderate confidence	5 studies reflecting minor concerns regarding methodological limitations and Minor concerns regarding adequacy.	Mancuso & Polzer 2010; Gray Brunton et al. 2014; Petrova et al. 2015; Chan et al. 2011; Siu 2013;
**Theme 9: Gendered perceptions and bias**			
[Finding 27] Gendered perceptions of HPV risk: Many participants exhibit misconceptions about HPV being primarily a women’s disease.	Moderate confidence	7 studies reflecting moderate concerns regarding methodological limitations and minor concerns regarding adequacy.	Hodge 2014; Kim 2017; Allen et al. 2009; Fontenot et al. 2016; Gerend et al. 2019; Jaiswal et al. 2020; Mehta et al. 2013;
[Finding 28] Gender biased promotion: The gendered marketing and promotion of HPV vaccines led to perceived unfairness in who gets access to vaccine and who is responsible for getting the vaccinated in the context of sexual health.	High confidence	8 studies reflecting minor concerns regarding methodological limitations.	Hodge 2014; Apaydin et al. 2018; Fontenot et al. 2016; Gray Brunton et al. 2014; Jaiswal et al. 2020; Martin et al. 2011; Miller-Day et al. 2023; Wyndham-West 2016;
**Theme 10: Government and policy**			
[Finding 29] The government’s involvement and role (or lack thereof) in HPV vaccination promotion were influential in vaccine decision making.	Moderate confidence	5 studies reflecting minor concerns regarding methodological limitations, and minor concerns regarding adequacy.	Chen et al. 2021; Petrova et al. 2015; Basnyat & Lim 2018; Hodge et al. 2011; Ross et al. 2010;

### Synthesis of findings

This section details the review findings from the QES. The review findings are categorized into 10 thematic categories that emerged from the thematic data analysis process to reflect the barriers and facilitators to HPV vaccine uptake and decision-making processes among young adults. While the individual findings are presented as distinct elements with a specific theme, we acknowledge that findings may also fit or overlap with other thematic categories. The thematic categories include:

- Individual factors: influence of personal attitudes or beliefs on decision-making
- Alternatives to vaccination: behaviors/practices believed to be alternatives to the HPV vaccine
- Knowledge and information: knowledge and information about HPV/HPV vaccine
- Sex and romantic relationships: influence of sexual status and relations on decision-making
- Parents and peers: role of parents/peers in decision-making
- Physicians: role of physicians in decision-making
- Logistics: factors associated with the process of getting vaccinated
- The vaccine: components about the HPV vaccine influential in decision-making
- Gendered perceptions and bias: influence of gendered views on promotion and uptake
- Government and policy: role of government in decision-making

Theme 1: Individual factors

**Finding 1. Missed opportunity: Some young adults believed they missed the window for vaccination because they were too old to benefit from it.** Studies reported that some young adults believed they were too old to receive the HPV vaccine. This was due to lack of clarity about the vaccine’s efficacy past certain ages [65,72,108,110]: *“I heard that the HPV vaccination works on girls prior to age 15. It is too late for me to beneﬁt from the vaccination because I am too old and have been sexually active”* [108]. Some participants also believed the vaccine could cause adverse reactions when administered after certain ages [69] or associated the vaccine as one typically administered in childhood and subsequently no longer applicable when older:

“When I came to college, people were saying, “Oh, I got the HPV vaccine recently,” or whatever. I, like, “You’re 20-something years old. What do you mean you just got it?” I just thought it was one of those things that you just get when you’re young, like measles and like smallpox and all those vaccines.” [72]

**Finding 2. Low risk perception: Some young adults believed they were not at risk for HPV-related illnesses or that they still had time to decide on vaccination.** Personal risk perception played an influential role in vaccine decision-making, with some participants feeling they were low risk for HPV-related diseases to justify vaccination [45,66,75,81,110]: *“I think more the reason I just didn’t get [vaccinated] was just because - yeah, I guess you’re kind of right, since I was thinking, oh, it’s not that big of a deal for me. Maybe this isn’t a vaccine I 100 percent need to get right now”* [81]. Some young adults did not perceive the urgency to need to get vaccinated or believed it would never be too late for them to get vaccinated[67,101]: *“Since you can get vaccinated up to age 26, I have 5 more years to think about it”* [67].

**Finding 3. Proactive measure: Many young adults viewed vaccination as a proactive measure to safeguard their health in the present and prevent future regret if they didn’t get vaccinated**. Many young adults expressed a desire to protect themselves from cervical cancer [64,67,71,89,100,103] other diseases associated with HPV [52,71,89,106,110]: *“When I heard what the shot can help prevent- cervical cancer - I wanted to get it”* [67]. Some individuals recalled learning about HPV and the vaccine during specific life events, such as pregnancy/health diagnoses which served as the cue to action to get vaccinated [47,113]: *“I kind of panicked a little bit when I was diagnosed with HIV, but then I’m like, OK, I’m getting everything. So it’s like -- I got it [HPV vaccine]*” [47]. Participants who had experienced HPV-related symptoms expressed an increased interest in vaccination [60,78]: *“I started developing symptoms (warts), I kind of had a suspicion that’s what it was. I thought I wouldn’t be really suited for the vaccine, but then my doctor said that it might actually help”* [60].

Concerns about long-term health and future relationships drove some individuals to prioritize HPV prevention and avoid future regret [64,106]:

“…I didn’t want to eventually be married and have that, you know, worry in my mind about HPV.” [106]

‘‘I think it would be a good thing to do. Instead of sitting there and be like ‘‘ok, I’m not gonna do it, then then all of a sudden you go to the doctor and they say you have cancer, you gonna think back like, ‘‘Oh I should’ve continued the shots instead of doing all that.“ [64]

**Finding 4. Low-stakes preventive measure: Several individuals mention the simplicity of the decision to get vaccinated, seeing it as a low-risk preventive measure with potentially significant benefits** [54,64,72,75,78,89]

“Well, it doesn’t take much to vaccinate people, so I guess it is helpful. Just go and get a shot and you are all set.” [89]

“There is no harm in getting the vaccine. It’s a preventive measure. It’s like one of those there’s nothing to lose type of thing.” [72]

Theme 2: Alternatives to vaccination

**Finding 5. Reliance on pap smears: Young adult women believed that regular Pap testing negated the need for HPV vaccination.** Some young adult women believe regular cervical cancer screenings were sufficient to monitor their sexual health, with many questioning the necessity for vaccination if they still had to continue getting screened [65,76,88,108]: *“Cervical cancer is treat-able if caught early, so there would be no need to get a vaccine if you are doing an annual Pap smear”* (US) [65]. In one study of participants from multiple countries, female participants also express costed savings from not getting vaccinated and just getting screened:

“Screening is something that might be...even more effective, then if you keep track you’re going to avoid it and all that. Had I known it earlier, we would have saved a lot of money from vaccines [laughter]. If I’m going to have my check, what’s the use of it?” [88]

**Finding 6. Condom use and other precautions: Young adults expressed precautions such as condom use, abstinence, and decisions on sexual partners were sufficient to protect against HPV, rendering the vaccine unnecessary.** Some participants believed that making selective decisions about who to have sexual relations with and using barrier methods are effective in preventing HPV infection, leading to a perception that vaccination is unnecessary [67,108]:

“My mother said to just not be stupid about sex … I’m not an idiot about who I sleep with. I really feel like HPV only affects people who don’t make smart decisions. If there are condoms out there today, use a condom. … I feel like there are other ways to prevent HPV… less serious ways than getting the vaccine.” [67]

Condom use was highlighted as a primary method of protection against HPV [67,81,89,108] as exemplified by statements such as “*I feel like condoms will protect me”* [67] and “*I typically wear condoms so I’m not particularly worried about it*” [89]. Some individuals also expressed confidence in abstinence as a means of HPV prevention, questioning the need for vaccination [80,106]: *“I am abstinent, why should I get it?”* [80]

Theme 3: Knowledge and Information

**Finding 7. Many young adults expressed having insufficient information on HPV or the vaccine before deciding to get vaccinated. There was an expressed desire more information about HPV and the HPV vaccine.** Young adults expressed concerns about not knowing enough about HPV or the vaccine [58,59,65,71,72,81,96]. Some participants demonstrated a lack of information on HPV transmission [54,62,63], how the vaccine works [54,60,78], and the association between HPV and cervical cancer [64].

If I have more information about the vaccine...I would probably get it …. As the old adage says, it is better to look before you leap. If I have more information about the vaccine, I’m willing to get it, but I don’t know what it is very well.” [72]

Some young adults admitted that they had never heard of HPV before vaccination or receiving the HPV vaccine without understanding what HPV is or how it is transmitted and others [54,58]: “*I have no idea what it is honestly, I got the Gardasil shot...... my mom wanted me to... but I don’t know anything about it*” [54].

Participants expressed a desire for more information and awareness of HPV and the vaccine [65,72], which ranged from general knowledge about the benefits of the vaccine [58,59] to more scientific/research- related information on the vaccine [66,89]: “*It’d be very unlikely (to accept the vaccine) because I’d have to-like I said earlier-I’d have to go on the Internet and just see what has other researchers come up with and other studies and other experiments on it-what would all that calculate to*” [89].

Theme 4: Sex and romantic relationships

**Finding 8. Sexual activity and behavior: Some individuals believe that vaccination against HPV is only necessary if one plans to engage in sexual activity or has many sexual partners.** Some young adults expressed a lack of perceived need for vaccination because they were not currently sexually active and therefore did not need the vaccine [54,59,63,64,71,75,101,103,106]: *“I have no sexual experience, so I do not think I am at risk of getting cervical cancer. Cervical cancer is caused by sexual intercourse. I think those who plan to have sex may ﬁnd the vaccine more useful. There is no need for me to get vaccinated if I do not have sex or get married in the near future.”* [101]

Additionally, young adults also believed that people who had a lot of sex partners should get vaccinated [67,81,88,106,110]: *“So, it will make sense to do it if you are going to be out here just engaging in a lot of sex with a lot of people, it makes sense to protect your-self”* [81].

**Finding 9. Relationship status influenced risk perception and subsequent vaccination behavior among young adults.** Many young adults in committed or monogamous relationships often perceived themselves to be safer and perceived being at lower risk for HPV infection, thereby negating the need for HPV vaccination[67,78,80,81,89,106]: *“I feel like if I were in - I don’t know like single or perhaps in like a non-exclusive relationship, it’s something that I would think about a little bit more. But as - yeah, I’m just like - it - it hasn’t really felt as relevant for me, but I deﬁnitely think that there’s like a lot of beneﬁts to it”* [106]. Conversely, a few young adults expressed interest in vaccination or that they did get vaccinated because of the additional safety and protection it offered even if they were in a relationship [106,107]: “*Yes, only because like we were sexually active, and I was like any kind of like extra protection I was game for. He -he had been with other people before, and I’m like you can’t really tell when somebody has HPV; he might not even know”* (P19, single and dating, vaccinated) [106]. Changes in relationship status also appeared influential. Some participants indicated they would consider getting vaccinated if they became single [81] while others expressed that they got vaccinated after their relationship status changed: *“I wasn’t going to get it, but I broke up with my boyfriend. That’s when I had my first shot”* [67]*.*

**Finding 10. Parental beliefs about sex and the HPV vaccine influenced young adults’ vaccine attitudes and decisions.** Participants reported feeling influenced by their parents’ beliefs regarding vaccination and sexual behavior. Parental beliefs included perceptions that getting the HPV vaccine advocates having sex [58,65,67,79,100,101]; monogamous relationships negate the need for vaccination [67]; or attitudes about morality and protecting one’s virginity till marriage [101]. In these cases, young adults were deterred from getting seeking vaccination.

“My mother was very anxious after learning that I wanted to have the cervical cancer vaccine. She said only girls who want to do something bad [have sex] want to have such vaccination, and there is no need for me to do so. My mother said it is important for good girls to protect their virginity until they get married. The reaction of my mother was so strong that it made me dare not mention it again.” [101]

Theme 5: Parents and peers

**Finding 11. Mothers more than fathers played an active role in HPV vaccination process among young adults.** Many participants cited their mothers as significant sources of influence in getting the vaccine, through providing information and advice, conducting research before vaccination, and advocating for their children to receive it [48,54,59,60,64,67,72,75,102]. Mothers also facilitated important cues to action such as scheduling vaccination appointments, demonstrating their active involvement in the vaccination process [67,81,96]: *“Just every time I go in for a vaccine, my mom set the appointments. I just go and get the shot; get a nice little bandage and leave”* [81]. Some participants expressed that their mothers played a crucial role in advocating for HPV vaccination in situations where other family members such as fathers were more reluctant and hesitant about the necessity of vaccination for their child [54,78]: *“And my dad was just kind of furious [about the cost and need if she wasn’t sexually active] and then... My mom was like, yeah... we need to get these and it was all okay”* [54].

**Finding 12. Decision-making power: There was a mix of deferred or shared the decision to get vaccinated between young adults and parents.** Some participants deferred vaccination decisions to their parents, particularly their mothers, trusting their understanding of healthcare information and recommendations [54,64,81]: *“Anything else again any new vaccines or any diseases going around I actually leave that with my mom. Cause she will better understand what people are saying than what I would.“* (Male 18, non-initiator) [64]. In other instances, some young adults engaged in discussion and shared decision-making with their parents when considering vaccination [82,103]: *“I’ve discussed it with my parents but we all agreed we wanted to wait a couple more years to see what the side effects were”* [103].

**Finding 13. Peer influence: Friends and acquaintances played a significant role in shaping vaccination decisions.** Young adults who believed their peers were vaccinated or had friend groups of people who were vaccinated expressed favorable vaccine attitudes and willingness to get vaccinated [54,63,67,96,107]: *“My mom didn’t want me to get it done but I thought everyone else is getting it done so I might as well”* [96]. Friends were also seen as a trust source of vaccine information which influenced uptake, and, in some instances, friends would decide to get vaccinated together [48,54]: *“One day I was just talking in the group chat, and I [asked] my friend “Oh, who hasn’t gotten cervical cancer jab? I’m thinking of getting it.” One of my friends said that she hasn’t gotten the jab as well, and then she also expressed her interest of getting it. So I said, “Why not we just go together?” She did all the research about the vaccine and then I just followed along”* [48].

Conversely, some young adults described instances where negative reactions or perceived negative perceptions from their friends deterred them from getting vaccinated [101,110]: *“Some of the friends that I do have in particular would probably think that I’m just being extremely promiscuous sleeping around with guys like every night.”* [110]

**Finding 14. Health history of family and friends was a motivating factor in the decision to get vaccinated.** Concerns about cancer given family history or observing others battled HPV-related illnesses emerged as a motivator for vaccination among some participants [52,63,89,103,113]:

“Having seen my friend going through having HPV I think it’s a pretty terrible thing to go through and it affects who she chooses as a future partner, she’s a single woman, right. So it’s going to affect how her relationships end up unravelling and that sort of thing and whom she meets. I mean a world without HPV would be nice. But I guess my own concern about my sexual health outweighed any of the potential problems that might come with it.” [113]

Theme 6: Physicians

**Finding 15. Doctor recommendation: Physician recommendations (positive or negative) were influential in vaccination uptake.** Many young adults cited their doctor’s positive recommendation as a significant factor in their decision to get vaccinated, viewing it as essential for their health and trusting their expertise [47,51,54,60,64,67,69,71,81,89,96,106,113]: *“It was the doctor’s recommendation. I honestly wouldn’t have thought about it had he not recommended it*” [60]. Participants expressed trust and confidence in their doctors’ recommendations, seeing them as experts whose advice should be followed for health-related matters [48,71,89,102,107,110].

Conversely, some individuals reported their doctor either advising against vaccination [53,54,69], or never mentioned or offered the vaccine [67,69,71,79]:

“I didn’t get it [the HPV vaccine]... because my doctor told me it wasn’t necessary if I wasn’t sexually active or anything.” [54]

“I talked to my doctor, and he honestly told me it was basically that...if people have not been getting the shots and not been getting cancer for a long time and it doesn’t run in my family, I don’t have a high probability of having it.” [53]

**Finding 16. Communication gap with doctors: Young adults desired better communication and more informed conversations with their doctors.** Some participants felt that doctors were reluctant to provide detailed information unless prompted, leading to uncertainty and frustration about where to find trustworthy information [69,82,88,101]:

I : So when you did bring [HPV] up to your doctor, did she talk to you at all about the virus?

P: No, not at all...She was like “I guess you’re sexually active, like you can get it if you want it.”...I think this was before I was sexually active, because this was like my annual physical freshman year of college, and I brought it up and I’m like “This is something I read I needed,” and she didn’t talk about side effects, she didn’t talk about what can come with it, it was like me advocating for myself and she was like “You can get it if you want but like you’re gonna have to pay out of pocket.” [69]

Participants expressed a need for open, non-judgmental communication with healthcare providers, where they could ask questions, receive thorough explanations and comprehensive information about the vaccine, be treated respectfully and not feel rushed [47,58,59,89]:

“The first time I was offered the HPV vaccine, I just said no, because I didn’t understand what the doctor said. Some of the terms I didn’t understand, and she just spoke brief about it. I was shy at the time, too shy to ask any questions. I saw her [provider] in a hurry, and I was like, “Oh yeah, I understand,” but in reality, I didn’t. I just said that because she seemed in a hurry.” [59]

Participants also stressed the importance of providers creating a safe and non-judgmental environment particularly given stigma and discomfort around discussing sexual health [58,89]: *“There is still some taboo feeling with discussing sex with anybody so I think it’s really important that the doctor listen and be understanding of any responses that the patient might give.”* [89]. This was particularly important for LGBTQ+ participants who highlighted the importance of LGBTQ+ Inclusive healthcare and preferences of receiving care from LGBTQ+ friendly healthcare providers [47,110]: *“A gay provider would be more into or up-to-date with newer things that are coming out. Especially like with the threats that are more for the gay lifestyle. Because I really don’t think that my health provider would know about HPV”* [110].

Theme 7: Logistics

**Finding 17. Appointments: Scheduling and appointment challenges impacted young adults’ ability to get vaccinated.** Participants described difficulties in completing the vaccine series due to busy schedules and other responsibilities which made it a challenge finding time to go get vaccinated [47,63,64,69,82,102,103,105]. Young adults expressed interest in combining HPV vaccination with other medical visits such as annual physicals to streamline the process and make it more convenient [58,69,89,105]: *“It would be hard for me to schedule an appointment to complete the HPV vaccine series. It would be easy to get the HPV vaccine if offered during my clinic visits”* [105]. For some participants, HPV vaccination was simply not a priority to make an effort for [69,103]: *“I never got around to it and to be honest I don’t think it’s a big deal to change my schedule for’‘* [103].

**Finding 18. Multiple shots: Some young adults found the process of going back multiple times for the three doses burdensome, leading to forgetfulness or avoidance.** Challenges with appointments were compounded by the need for multiple visits to complete the HPV vaccine series which young adults found inconvenient [47 67,69,75,81,105]:

‘‘It’s so troublesome…it’s not like maybe 3 times a week you can get it over and done with. It’s over more than a year, it’s really long…” [75]

“I would have gotten it… it’s just that getting three shots across nine months is a hassle, and I’ve been moving around a lot in the last couple of years” [67]

Some participants were unclear about the number of doses required for the HPV vaccine and what to do if they missed doses, leading to missed opportunities for completion [63,96]: *“didn’t even know there were three shots involved with it until I came in”* [63].

**Finding 19. Accessibility: Transportation and access challenges hindered HPV vaccination uptake.** Participants cited lack of transportation or long distances as barriers to HPV vaccination [55,64,65,67,82]. Alternative locations such as pharmacies, emergency rooms or vaccine drives in atypical settings were also discussed as preferred venues to receive the HPV vaccine and increase accessibility to get vaccinated [64,82,96,110]. For some participants on college campuses, research authors noted there seemed to be an unawareness of the vaccine availability at the local campus clinic [64,75].

**Finding 20. Reminders: Reminders were cited as helpful in ensuring adherence to the vaccination schedule.** Whether from providers or parents, or personal calendar systems to track information, young adults relied on reminders to aid them remember appointments and get vaccinated [47,64,96]. Several individuals mention that their mothers were responsible for scheduling their vaccine appointments, indicating a dependency on family members for healthcare management [67,81,96]: *“It was up to my mom to book the appointment -again”* [96].

**Finding 21. Mandates: Young adults had mixed views on mandates for HPV vaccination.** Some participants opposed mandates for HPV vaccination [71,92]. Other participants expressed compliance with mandates as part of requirements for school enrollment [64,81,100], support of public safety efforts [89], or trust in the government making vaccination compulsory [75].

Theme 8: The vaccine

**Finding 22. Vaccine cost: Young adults expressed concerns about paying for the HPV vaccine, citing high out-of-pocket costs and need for government subsidies.** Participants expressed difficulties in paying the cost for the vaccine series and subsequently opting out of vaccination [51,52,62,64,67,75,81,96,101,105,108]. Young adults also highlighted the impact of insurance coverage on their ability to afford the vaccine and decision to get vaccinated [53,63,65,89,108,110].

“”The vaccine is too expensive. It costs almost my whole monthly wages from my part-time job. If I really have the need, I would not mind spending my wages on the vaccination. However, I am still unmarried so I do not think I have the need at this moment. Spending such a large amount of money on a vaccine that is not useful for me at this moment is not worthwhile.” [101]

I would have received the HPV vaccination if my health insurance covered it at that time or if the government paid for the HPV vaccination.” [108]

Parental willingness or refusal to pay was also influential in the decision to get vaccinated [54,67,75,100,101]:

“My mother said there is no use for me to get vaccinated except if I want to do something inappropriate [having sex]. As my mother discourages me to get vaccinated, I will not have it because she will not pay for it or sponsor me. I just have one part-time job but the wages are insufﬁcient to pay for the vaccination.” [101]

Don’t know [how much I would be willing to pay for the vaccine]. Depends on how much parents will be willing to spend!” [100]

Young adults called for governments to subsidize the cost of the vaccine, suggesting it would encourage more people to get vaccinated [51,52,75,96]: *“The main concern is the price. If the government provides more subsidies to make the vaccine cheaper, maybe (reducing) several hundred or hundred and something, it may make people more willing to be vaccinated”* [51].

**Finding 23. Side effects: Young adults expressed concerned about the side effects of the HPV vaccine.** Across many studies, participants expressed worries about potential side effects of vaccines. Participants were concerned about not knowing the long-term effects of the vaccine or how the vaccine will affect them [51,59,65,76]. Young adults were also concerned about the (unknown) ingredients of the vaccine and not wanting them in their body [59,64,71,72]. Concerns about the vaccines side effects were also fueled by the negative stories and experiences others had of getting vaccinated [54,65,69,88,101,103].

“I trust my body. To be honest, I don’t feel comfortable having it [HPV vaccine] in my body. I feel like I don’t need it. I’m not even active [sexually] like that. I don’t want to put something in my body if I don’t know how it’s going to affect me. I’d rather not risk it. I’m actually kind of scared to even do it” [59]

“Only one or two friends of mine have had the cervical cancer vaccine; they commented the shot was particularly more painful than other vaccines. Also, one of my friends suffered from fever, dizziness, extreme tiredness and a rash for some days just after the ﬁrst dose, and she dared not continue the remaining course, so I do not think about receiving the vaccine at this moment.” [101]

**Finding 24. Understanding of vaccine effectiveness: Young adults questioned the effectiveness of the HPV vaccine given the limited strains it covers and lack of 100% protection.** Doubts about the effectiveness of vaccines were expressed particularly given the vaccine does not guaranteeing 100% protection [62,76,106,110,115]: *“Um, of course, now it’s [risk for HPV] deﬁnitely much, much lower. There’s still a risk because the vaccine doesn’t work 100 percent,”* [106]. In one study where participants had also received cytology testing, individuals with abnormal cytology results who also received the vaccine expressed frustration with the vaccine: *“The vaccine was a waste of time. I would say it’s pointless and it’s not doing what it’s supposed to”* [115].

Some young adults also questioned the usefulness of vaccines if they were already sexually active or have had multiple partners, which they felt potentially negated the vaccine’s effectiveness [76,88,115]: *“Well, we are already sexually active, and you are supposed to get the vaccine before you become active, because then it is most....I forgot the word... most effective. Actually, at this point, you can’t be sure about the effectiveness of the vaccine”* [88].

Additionally, the knowledge that the HPV vaccine only protects against specific strains of HPV led some young adults to believe this undermined the effectiveness of the vaccine[54,65,96]:

‘‘You’ve got to take into consideration that vaccination is only preventative for certain kinds of HPV.’‘ [65]

“... because like what’s going on with swine flu right now and how they will vaccine like one part of the disease... but then there’ll be another outbreak of a different [type] so I kind of think about that with the HPV; like what if they vaccinate me and I still get cervical cancer, like a different type of cervical cancer so that why I’m leery…” [54]

**Finding 25. Development of the vaccine: Concerns were raised about the vaccine’s novelty, hastened development, and lack of long-term studies.** Some participants believed the vaccine development was rushed [54,88,113] and many others cited the absence of long-term studies on the vaccine’s effects, and subsequent wariness about being early adopters or “guinea pigs” for the vaccine [54,62,64,67,115]. Young adults also expressed concerns about the vaccine’s safety and efficacy, emphasizing the need for longer investigation to determine potential side effects and effectiveness [51,66,67,75,88,108,113].

“against it because it just wasn’t very tested; it seemed to me like they approved it before they even were finished testing it in everybody and I thought that was really jumping the gun and not very safe […] the regular vaccines like that you get through public school and... ones that have been around for a long time, I’m not that worried about. But since this is so new... that makes me kind of nervous.” [54]

“... I know that this vaccine has not been introduced for long, only around 10 years. Therefore, I think it requires long time observation to determine the effect.” [51]

Inaccurate knowledge about how vaccines work also fueled some of the wariness and skepticism [54,115]:

“[T]he same concerns I have with any vaccine is... you’re getting the virus, it’s a very weak virus but you’re still getting the virus and you’re taking the risk that... maybe if the vaccine that you’re getting is not as weak, like the virus as weak as it should be, then maybe you’re more susceptible to actually like breaking out and getting the STD. But also just HPV, it seemed really hastened... When it came out for everyone to get it and it didn’t seem like it was very tested to me.” [54]

In one study involving UK participants, the change to quadrivalent vaccine led some participants who received the bivalent vaccine to believe they may not be fully protected because the bivalent vaccine was not as effective: *“ It’s different now. It’s been researched and changed. [...]I hope that other girls aren’t thinking ‘Well I’m fine because I’ve got it [vaccination]”* [115].

**Finding 26. Pharmaceutical companies and business motive: Young adults expressed skepticism towards pharmaceutical companies and their motives, suggesting that public panic and greed may influence vaccination campaigns.** Participants questioned the motive of vaccination promotion and who stood to most benefit [76,101]. Young adults also criticized the advertising tactics used for the vaccine suggesting campaigns may be manipulative or misleading by not adequately informing people about potential risks and benefits [62,88]; they also questioned *who* (e.g., drug companies vs the government) should be promoting the vaccine [51,76] and were critical of the marketing of the vaccine as a product [88,101].

“Well nowadays, whatever they offer me a vaccination against, a medicine, or whatever it is, and they tell me it is very good, I will always keep in mind who will win the most. Is it my health, is it this guy’s pocket, the government or state, or whoever it is, does not matter” [101]

“I think it [marketing campaign] was quite directed...which then to me I think that’s wrong, you shouldn’t be advertising, you know thinking about the audience like that because it’s not a product, it’s er.. medical, you’re not trying to sell this!” [88]

“... the government instead of the drug companies should take the role to promote (the vaccine)...If the drug companies take the initial role to promote, it could only promote through limited media sources... But if this is the government to take the role, the vaccine can be introduced in sex education.” [51]

Theme 9: Gendered Perceptions and Bias

**Finding 27. Gendered perceptions of HPV risk: Many participants exhibit misconceptions about HPV being primarily a women’s disease.** Some young adult men believed HPV does not affect men and that only women needed to be vaccinated because HPV affected them the most [45,60,66,69,80]. In some instances, men expressed knowing they could be carriers of HPV and pass it on to their (female) partners without themselves getting sick [58,60,69].

“I think the vaccine is for woman... I heard it’s only, the vaccine...no no, the virus only target woman, even if the man got it, it wouldn’t affect them. But he would be the carrier of that [virus], he might transmit that [virus] to his partner who is a female....” [69]

One study also demonstrated they gendered perception was not solely a male issue; a female participant also expressed no knowing men could get vaccinated [72]:

“I really didn’t know that men could get even the vaccine. My friend and I were talking about it. He’s like, “Yeah, I had Gardasil, too,” and I was like, “Really? You don’t have cervix. You’re a guy.” He was like, “It’s a vaccine for HPV.” I was like, “What?” It didn’t make sense to me at that time because I thought this vaccine is for women only.”

**Finding 28. Gender biased promotion: The gendered marketing and promotion of HPV vaccines led to perceived unfairness in who gets access to vaccine and who is responsible for getting the vaccinated in the context of sexual health.** Some young adults criticize the gendered nature of vaccination campaigns that were predominantly aimed at women, questioned the fairness of offering the vaccine primarily to girls and women and advocated for equal access to vaccination for all genders/sexes [58,62,81,113]:

“I’m really skeptical about the vaccine, particularly because it’s mass marketed towards girls and women and absolutely no focus on boys and men. And you know it’s a vaccine for HPV, it’s not a vaccine for cervical cancer even though the strains it targets can lead to cervical cancer. But I just feel that if we actually want to reduce cancer we probably should be vaccinating everyone. I think it is interesting that they’re targeting only girls and women because there hasn’t been any long-term research so we don’t really know what’s going to happen ten years down the line, 25 years down the line.” [113]

“I think the biggest thing is just letting [boys and men] know it’s an option because I, obviously, when I was 18 they didn’t tell me, so I didn’t get it. But the second I was told about it, I did. So, I think it’s just a matter of being aware that it exists.” [81]

Men and transgender individuals particularly noted the lack of representation and targeted advertising towards their demographic; this led to them feeling less informed about the risks and importance of vaccination [47,58,66,69]:

“It was that commercial. And I it stands out to me so much because I remember “HPV can lead to cervical cancer.” And I just remember hearing it and it stuck in my brain forever, and I just remember being like “Thank god I don’t have ovaries.” (I: Mhm.) But then I googled it one day cause I got, I was like “What if I get it one day” and I was like, “wait it can affect me too?” [69]

‘‘The biggest thing, again, was primarily marketed towards women, which - cisgender women - is, then, the question of, well, then, why did I bother, as a male? You know, as a cisgender male, it doesn’t make sense for me.” [47]

‘‘There is a misconception that if you’re a gay man, you don’t need to get it [HPV vaccine].“ [58]

A couple of studies revealed a discussion on the unequal distribution of responsibility for sexual health between genders, with some feeling that HPV vaccination campaigns unfairly target women and reinforce gender biases. Young adult women raised concerns about the stigmatization of women’s sexuality and the unequal burden of responsibility placed on them in sexual relationships, being a “scapegoat” of men’s sexual risk-taking behavior [77,113]:

“Ita: It sends a message that it’s a girl’s responsibility. It’s a girl’s responsibility to be on the pill and its just perpetuating that. When I was a teenager it was always the girls responsibility to carry a condom, it wasn’t for boys to worry about. Boys need to be responsible for their sexual health too.

Anna: Yeah, straight men may get reassurance from the fact that their partner has had the vaccine so they cannot catch it.” [77]

“Okay, so I have a problem with the vaccine even though I got it. The way it’s being marketed towards girls stigmatizes girls’ and women’s sexualities as being the source of the problem. Just like when you look at, on having responsible protective sex, again a lot of the onus is put on girls to be sort of the arbitrators of sexuality so that they have to monitor their partner’s behaviour and make sure to take on that responsibility of being responsible for both her and her partner without attributing very much responsibility to the man who is involved in the sexual relationship.” [113]

Theme 10: Government and policy

**Finding 29. The government’s involvement and role (or lack thereof) in HPV vaccination promotion were influential in vaccine decision-making.** For some young adults, the government’s involvement in and approval of the HPV vaccine was viewed as confirmation of its importance [48,96]:

“Well that kind of reassures you as well. If the government is making sure you have those jabs and they’re paying £400 for you to have them, then it does work and it helps people…” [96]

“For me, if the government thinks that it’s important enough for you to use your CPF [Central Provident Fund] money for it or even Medisave money for it then it must be important enough. Enough important [to be] on the radar for them. I mean you won’t see them [government] giving flu vaccination and tapping from Medisave right?” [48]

Conversely, lack of government involvement in HPV vaccine promotion raised questions among participants [52,88]: *“Well, for me the primary reason is that, in reality, if the government wanted to support this thing, it would have provided more information, as a first step towards action”* [88]. Similarly, mistrust in the government led some participants to be critical of government initiatives: “*For Natives, vaccinations are crazy. The whole history with the IHS (Indian Health Service), I’m not sure people believe they can trust them[the government]*’‘ [65].

### SEM Categorization of findings

**Fig 2:**
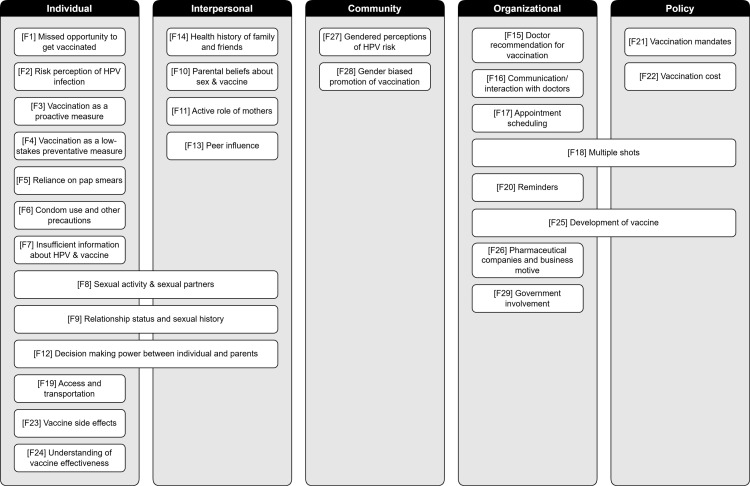
Socioecological characterization of review findings.

To enhance our understanding of the factors that influence young adult vaccination decision-making and uptake, we organized our review findings across the domains of the socioecological model (SEM). The SEM proposes an interplay and interdependence of factors within and across different ecological levels that influence health behaviors [117]. We acknowledge that not all findings neatly fit into the constructs of the SEM and that some findings may span multiple domains–we placed findings in ecological levels that was most representative of their level of influence as presented by the data (Fig 2).

## Discussion

This systematic review aimed to qualitatively synthesize the literature on young adults’ perspective on HPV vaccine decision-making and the associated barriers and facilitators that influence their vaccination uptake. The current review identified 68 studies from 71 articles that met inclusion criteria; of these, 42 studies exclusively sampled young adults between the ages of 18 and 26 years which formed the basis of the results. We identified 29 findings across 10 thematic categories which revealed a complexity of factors that are influential in decision-making and vaccination uptake among young adults. We found that some young adults believed that they had aged out of eligibility for HPV vaccination or that their sexual history would hinder the effectiveness of the vaccine and precluded them from the vaccine’s protective benefits. We also observed that some young adults considered condom use and regular screenings as alternatives to vaccination in preventing HPV infections. Lastly, we observed that young adults more frequently spoke of their mothers as playing an influential and active role in the vaccine decision-making and uptake process.

The studies that contributed to this review spanned multiple countries, representing various geographic, political, and economic contexts. The policies and guidelines adopted by different nations may influence and impact vaccination uptake. For example, the World Health Organization (WHO) recommends one or two HPV vaccine doses for girls and women 18–20 years old and 2 doses for women older than 21 years [8]; in contrast, the CDC in the US recommends 3 doses of the HPV vaccine for all individuals aged 18–26 [118]. The observed differences in the two policies of who is advised to get vaccinated, and the number of doses required, have implications on vaccination uptake as demonstrated by our findings. On the issue the vaccine schedule, our review findings showed that returning for multiple doses was a barrier for some young adults. On the matter of persons to get vaccinated, the CDC policy to vaccinate everyone regardless of gender/sex aligns with our findings advocating for gender balance in the marketing and promotion of the HPV vaccine and the responsibility of everyone – not just females – to get vaccinated. This in no way diminishes the vital importance of WHO’s position that has a primary focus on cervical cancer prevention [8] and optimizing vaccination in under-resourced countries who experience a disproportionate burden of cervical cancer[119]. However, given this review’s findings, we encourage that where feasible, the promotion of HPV vaccination be inclusive and gender neutral.

Findings from this review highlight several constraints young adults experience in getting vaccinated, including cost of and accessibility to the HPV vaccine; specifically, some participants cited lack of transportation access or long travel distances as a barrier to getting vaccinated, and others expressed difficulty in paying for the vaccine series due to lack of insurance, high out-of-pocket expense, or lack of government subsidy. Globally, 143 (75%) countries include the HPV vaccine in their national immunization programs (NIP), providing the vaccine at no or reduced cost [120]. One study conducted on NIPs in Asia-Pacific region found that only 3 of the 10 countries evaluated include catch-up vaccination in their immunization program –and notably no country included males in their NIP [121]. Further investigation should be undertaken on the eligibility and inclusion of HPV catchup vaccinations included in NIPs globally. Additionally, many primary HPV immunization programs offer vaccine delivery in school settings and these school-based programs have shown significant improvements in adolescent vaccine coverage [9,122]. For young adults, college campuses are posited as the ideal setting to address HPV vaccination disparities [123]. One challenge with college settings is that while HPV vaccines may be available at college health centers, the utilization rate of campus health services for vaccination may not be fully maximized. A national study conducted in the US found that only 9% of visits to campus health centers at 4-year institutions involved a vaccination of any type [124]. Future research should investigate the utilization of campus health services for HPV vaccination and additionally, evaluate the processes by which unvaccinated students are nudged/encouraged to get vaccinated. An assessment of vaccine policies across 96 higher education institutions in the US found that no institution required the HPV vaccine for school entry and only 36.5% of institutions recommended it for enrollment [125]. Given the prevalence and associated cancer burden of HPV infections, it is imperative that all avenues be pursued to increase the HPV vaccine coverage among young adults.

While young adults’ HPV vaccination decision is generally thought of to be autonomous and separate from parental attitudes, involvement, or supervision [123,126], our review findings suggest that parents may still be influential in the process—whether it was providing information, scheduling appointments, or being deferred to for decision-making. Additionally, our findings also contextualize the evidence supporting the importance and implications of physician recommendations for HPV vaccination; young adults who were willing to get the vaccine were met with either support or reluctance by physicians in providing information about the vaccine or advocating for its necessity. It also became evident from our findings that the nature of the interaction with physicians may play an important role; the “rushed” nature of medical appointments may discourage openness and dialogue that some young adults are seeking when deciding on vaccination. Parents and physicians are some of the key trusted sources of medical information for young adults [127,128] which may account for the importance of these entities in vaccine decision-making. Future interventions on catch-up vaccination should consider engaging vaccine-hesitant parents and physicians in some capacity, given the influential roles they play in the decision-making process.

There are several limitations to account for with this review. During the screening process, we encountered studies that also included participants under 18 years or over 26 years, and excluded articles where we were unable to extract data explicitly from participants in our age group of interest; consequently, literature providing important information on the topic may have been excluded from this review. Similarly, given the explicit focus on young adults aged 18–26, these findings may not be generalizable to other age groups. Additionally, given we purposively sampled articles to manage the volume of the qualitative data for analysis, we may have also excluded rich data to contribute to the review findings. It is also worth noting that the age cut of 18 years used for this review is based on the legal age of consent standard in the US; given we did not restrict the geography of studies during our search, it is possible that studies from other countries sampling young adults of legal consenting age younger than 18 years may have been excluded. Lastly, while this review included studies across 14 countries, we did not present the findings in a country or regions specific manner, which may hinder generalizability across geographic locations given differences in norms, culture and policies that may exist from country to country.

Despite these limitations, this review contributes valuable insights on HPV vaccination among young adults. The novelty of our study as one of the principal QES on catch-up HPV vaccination, presents findings that have been systematically evaluated and thus offer greater assurance in the review findings. Our findings also underscore the complexity of factors at play across multiple ecological levels, any combination of which may aid or impede vaccination uptake among young adults. There continues to be a need to provide accurate and reliable information about HPV and the vaccine; similarly, while efforts have been made by research and medical communities to focus messaging about the HPV vaccine on cancer prevention rather than sexual transmission [129], our findings illustrate a need for some education around sexual health and HPV that emphasizes the importance of HPV vaccination regardless of sexual history or sexual health behaviors (e.g., condom use or monogamous relationship status). Furthermore, our findings on parental influence on vaccine decision-making underscore the need for continued research assessing the impact of parental influence on older adolescents’ health behaviors; investigations should also explore social network influences of vaccine attitudes/beliefs on vaccination outcomes, particularly within a familial network. Lastly, efforts should also continue of help keep the cost of the HPV vaccine affordable, particularly through policy interventions that expand/increase insurance coverage for young adults or promote the adoption of national immunization plans that include young adults.

## Conclusion

HPV vaccines remain the most effective form of prevention against HPV infections and HPV-associated cancers. The findings from this review present important considerations to account for when designing and implementing interventions, programs, and policies aimed at addressing HPV vaccination disparities among young adults.

## Supporting information

S1 Appendix Database Search Terms. (DOCX)

S2 Appendix Identification of New References following Search Strategy Updates .
(DOCX)

S3 Appendix GRADE CERQUAL Evidence Table .
(DOCX)

S4 Appendix Prisma Checklist .
(DOCX)

S5 Appendix Excluded Studies .
(PDF)

## References

[1] Bray F, Laversanne M, Weiderpass E, Soerjomataram I. The ever-increasing importance of cancer as a leading cause of premature death worldwide. Cancer. 2021;127(16):3029–30. doi: 10.1002/cncr.33587 34086348

[2] de Martel C, Plummer M, Vignat J, Franceschi S. Worldwide burden of cancer attributable to HPV by site, country and HPV type. Int J Cancer. 2017;141(4):664–70. doi: 10.1002/ijc.30716 28369882 PMC5520228

[3] Williamson A-L. Recent developments in human papillomavirus (HPV) Vaccinology. Viruses. 2023;15(7):1440. doi: 10.3390/v15071440 37515128 PMC10384715

[4] Bray F, Laversanne M, Sung H, Ferlay J, Siegel RL, Soerjomataram I, et al. Global cancer statistics 2022: GLOBOCAN estimates of incidence and mortality worldwide for 36 cancers in 185 countries. CA Cancer J Clin. 2024;74(3):229–63. doi: 10.3322/caac.21834 38572751

[5] Guida F, Kidman R, Ferlay J, Schüz J, Soerjomataram I, Kithaka B, et al. Global and regional estimates of orphans attributed to maternal cancer mortality in 2020. Nat Med. 2022;28(12):2563–72. doi: 10.1038/s41591-022-02109-2 36404355 PMC9676732

[6] Centers for Disease Control and Prevention. HPV vaccine administration 2021. 2021. https://www.cdc.gov/vaccines/vpd/hpv/hcp/administration.html

[7] World Health Organization. WHO updates recommendations on HPV vaccination schedule. 2022. https://www.who.int/news/item/20-12-2022-WHO-updates-recommendations-on-HPV-vaccination-schedule

[8] World Health Organization. Human papillomavirus vaccines: WHO position paper (2022 update). Weekly Epidemiological Record. 2022;97(50):645–72.

[9] Bruni L, Saura-Lázaro A, Montoliu A, Brotons M, Alemany L, Diallo MS, et al. HPV vaccination introduction worldwide and WHO and UNICEF estimates of national HPV immunization coverage 2010-2019. Prev Med. 2021;144:106399. doi: 10.1016/j.ypmed.2020.106399 33388322

[10] Spayne J, Hesketh T. Estimate of global human papillomavirus vaccination coverage: analysis of country-level indicators. BMJ Open. 2021;11(9):e052016. doi: 10.1136/bmjopen-2021-052016 34475188 PMC8413939

[11] Shannon CL, Klausner JD. The growing epidemic of sexually transmitted infections in adolescents: a neglected population. Curr Opin Pediatr. 2018;30(1):137–43. doi: 10.1097/MOP.0000000000000578 29315111 PMC5856484

[12] Burger EA, Kim JJ, Sy S, Castle PE. Age of acquiring causal human papillomavirus (HPV) infections: Leveraging simulation models to explore the natural history of hpv-induced cervical cancer. Clin Infect Dis. 2017;65(6):893–9. doi: 10.1093/cid/cix475 28531261 PMC5850533

[13] Callahan ST, Cooper WO. Changes in ambulatory health care use during the transition to young adulthood. J Adolesc Health. 2010;46(5):407–13. doi: 10.1016/j.jadohealth.2009.09.010 20413075

[14] Arnett JJ. Emerging adulthood. A theory of development from the late teens through the twenties. Am Psychol. 2000;55(5):469–80. doi: 10.1037//0003-066x.55.5.469 10842426

[15] Arnett JJ. Emerging adulthood: The winding road from the late teens through the twenties. 2023. Oxford University Press.

[16] Barnard M, Cole AC, Ward L, Gravlee E, Cole ML, Compretta C. Interventions to increase uptake of the human papillomavirus vaccine in unvaccinated college students: a systematic literature review. Prev Med Rep. 2019;14:100884. doi: 10.1016/j.pmedr.2019.100884 31193049 PMC6513780

[17] Smulian EA, Mitchell KR, Stokley S. Interventions to increase HPV vaccination coverage: a systematic review. Hum Vaccin Immunother. 2016;12(6):1566–88. doi: 10.1080/21645515.2015.1125055 26838959 PMC4964671

[18] Lott BE, Okusanya BO, Anderson EJ, Kram NA, Rodriguez M, Thomson CA, et al. Interventions to increase uptake of Human Papillomavirus (HPV) vaccination in minority populations: a systematic review. Prev Med Rep. 2020;19:101163. doi: 10.1016/j.pmedr.2020.101163 32714778 PMC7372149

[19] Walling EB, Benzoni N, Dornfeld J, Bhandari R, Sisk BA, Garbutt J, et al. Interventions to Improve HPV Vaccine Uptake: a systematic review. Pediatrics. 2016;138(1):e20153863. doi: 10.1542/peds.2015-3863 27296865

[20] Fu LY, Bonhomme L-A, Cooper SC, Joseph JG, Zimet GD. Educational interventions to increase HPV vaccination acceptance: a systematic review. Vaccine. 2014;32(17):1901–20. doi: 10.1016/j.vaccine.2014.01.091 24530401 PMC4285433

[21] Escoffery C, Petagna C, Agnone C, Perez S, Saber LB, Ryan G, et al. A systematic review of interventions to promote HPV vaccination globally. BMC Public Health. 2023;23(1):1262. doi: 10.1186/s12889-023-15876-5 37386430 PMC10308645

[22] Rodriguez AM, Do TQN, Goodman M, Schmeler KM, Kaul S, Kuo Y-F. Human papillomavirus vaccine interventions in the U.S.: a systematic review and meta-analysis. Am J Prev Med. 2019;56(4):591–602. doi: 10.1016/j.amepre.2018.10.033 30773231

[23] Ferrer HB, Trotter C, Hickman M, Audrey S. Barriers and facilitators to HPV vaccination of young women in high-income countries: a qualitative systematic review and evidence synthesis. BMC Public Health. 2014;14:700. doi: 10.1186/1471-2458-14-700 25004868 PMC4100058

[24] Page M, McKenzie J, Bossuyt P, Boutron I, Hoffmann T, Mulrow C, et al. The PRISMA 2020 statement: an updated guideline for reporting systematic reviews. BMJ. 2021;372:e071. doi: 10.1136/bmj.n71 PMC8005924 33782057

[25] Noyes J, Booth A, Cargo M, Flemming K, Harden A, Harris J, et al. Qualitative evidence. Cochrane handbook for systematic reviews of interventions. 2019;525–45.

[26] Mantina NM, Nakayima Miiro F, Smith J, McClelland DJ, Magrath PA, Madhivanan P. Perspectives of HPV vaccination among young adults: a qualitative systematic review and evidence synthesis protocol. BMJ Open. 2023;13(12):e076234. doi: 10.1136/bmjopen-2023-076234 38072486 PMC10729288

[27] Bramer W, Bain P. Updating search strategies for systematic reviews using EndNote. J Med Libr Assoc. 2017;105(3):285–9. doi: 10.5195/jmla.2017.183 28670219 PMC5490709

[28] Ames HM, Glenton C, Lewin S. Parents’ and informal caregivers’ views and experiences of communication about routine childhood vaccination: a synthesis of qualitative evidence. Cochrane Database Syst Rev. 2017;2(2):CD011787. doi: 10.1002/14651858.CD011787.pub2 28169420 PMC5461870

[29] Hannes K, Booth A, Harris J, Noyes J. Celebrating methodological challenges and changes: reflecting on the emergence and importance of the role of qualitative evidence in Cochrane reviews. Syst Rev. 2013;2:84. doi: 10.1186/2046-4053-2-84 24135194 PMC3853345

[30] Ames H, Glenton C, Lewin S. Purposive sampling in a qualitative evidence synthesis: a worked example from a synthesis on parental perceptions of vaccination communication. BMC Med Res Methodol. 2019;19(1):26. doi: 10.1186/s12874-019-0665-4 30704402 PMC6357413

[31] Suri H. Purposeful sampling in qualitative research synthesis. Qual Res J. 2011;11(2):63–75. doi: 10.3316/qrj1102063

[32] Benoot C, Hannes K, Bilsen J. The use of purposeful sampling in a qualitative evidence synthesis: A worked example on sexual adjustment to a cancer trajectory. BMC Med Res Methodol. 2016;16:21. doi: 10.1186/s12874-016-0114-6 26891718 PMC4757966

[33] Harris PA, Taylor R, Thielke R, Payne J, Gonzalez N, Conde JG. Research electronic data capture (REDCap)--a metadata-driven methodology and workflow process for providing translational research informatics support. J Biomed Inform. 2009;42(2):377–81. doi: 10.1016/j.jbi.2008.08.010 18929686 PMC2700030

[34] Critical Appraisal Skills Programme. CASP qualitative research checklist 2018. 2018. https://casp-uk.net/images/checklist/documents/CASP-Qualitative-Studies-Checklist/CASP-Qualitative-Checklist-2018_fillable_form.pdf

[35] Karimi-Shahanjarini A, Shakibazadeh E, Rashidian A, Hajimiri K, Glenton C, Noyes J, et al. Barriers and facilitators to the implementation of doctor-nurse substitution strategies in primary care: a qualitative evidence synthesis. Cochrane Database Syst Rev. 2019;4(4):CD010412. doi: 10.1002/14651858.CD010412.pub2 30982950 PMC6462850

[36] Cooper S, Schmidt B-M, Sambala EZ, Swartz A, Colvin CJ, Leon N, et al. Factors that influence parents’ and informal caregivers’ views and practices regarding routine childhood vaccination: a qualitative evidence synthesis. Cochrane Database Syst Rev. 2021;10(10):CD013265. doi: 10.1002/14651858.CD013265.pub2 34706066 PMC8550333

[37] Braun V, Clarke V. Using thematic analysis in psychology. Qualitative Research in Psychology. 2006;3(2):77–101. doi: 10.1191/1478088706qp063oa

[38] Lewin S, Booth A, Glenton C, Munthe-Kaas H, Rashidian A, Wainwright M. Applying GRADE-CERQual to qualitative evidence synthesis findings: introduction to the series. Applying GRADE-CERQual to qualitative evidence synthesis findings. 2018:1–10. 10.1186/s13012-017-0688-3 PMC5791040 29384079

[39] Munthe-Kaas H, Bohren MA, Glenton C, Lewin S, Noyes J, Tunçalp Ö, et al. Applying GRADE-CERQual to qualitative evidence synthesis findings-paper 3: how to assess methodological limitations. Implement Sci. 2018;13(Suppl 1):9. doi: 10.1186/s13012-017-0690-9 29384078 PMC5791044

[40] Colvin CJ, Garside R, Wainwright M, Munthe-Kaas H, Glenton C, Bohren MA, et al. Applying GRADE-CERQual to qualitative evidence synthesis findings-paper 4: how to assess coherence. Implement Sci. 2018;13(Suppl 1):13. doi: 10.1186/s13012-017-0691-8 29384081 PMC5791039

[41] Glenton C, Carlsen B, Lewin S, Munthe-Kaas H, Colvin CJ, Tunçalp Ö, et al. Applying GRADE-CERQual to qualitative evidence synthesis findings-paper 5: how to assess adequacy of data. Implement Sci. 2018;13(Suppl 1):14. doi: 10.1186/s13012-017-0692-7 29384077 PMC5791045

[42] Noyes J, Booth A, Lewin S, Carlsen B, Glenton C, Colvin CJ, et al. Applying GRADE-CERQual to qualitative evidence synthesis findings-paper 6: How to assess relevance of the data. Implement Sci. 2018;13(Suppl 1):4. doi: 10.1186/s13012-017-0693-6 29384080 PMC5791042

[43] Norwegian Institute of Public Health. GRADE-CERQual interactive summary of qualitative findings (iSoQ). 2023.

[44] Lewin S, Bohren M, Rashidian A, Munthe-Kaas H, Glenton C, Colvin CJ, et al. Applying GRADE-CERQual to qualitative evidence synthesis findings-paper 2: how to make an overall CERQual assessment of confidence and create a Summary of Qualitative Findings table. Implement Sci. 2018;13(Suppl 1):10. doi: 10.1186/s13012-017-0689-2 29384082 PMC5791047

[45] Chern CJ, Beutler E. Biochemical and electrophoretic studies of erythrocyte pyridoxine kinase in white and black Americans. Am J Hum Genet. 1976;28(1):9–17. doi: 10.1016/j.jadohealth.2009.05.014 2009 PMC1684914

[46] Al-Naggar RA, Isa ZM. Perception and opinion of medical students about Pap smear test: a qualitative study. Asian Pac J Cancer Prev. 2010;11(2):435–40. 20843130

[47] Apaydin KZ, Fontenot HB, Shtasel D, Dale SK, Borba CPC, Lathan CS, et al. Facilitators of and barriers to HPV vaccination among sexual and gender minority patients at a Boston community health center. Vaccine. 2018;36(26):3868–75. doi: 10.1016/j.vaccine.2018.02.043 29778516 PMC5990434

[48] Basnyat I, Lim C. Perspectives of young Chinese Singaporean women on seeking and processing information to decide about vaccinating against human papillomavirus. Women Health. 2018;58(7):806–21. doi: 10.1080/03630242.2017.1342741 28682189

[49] Bunton V, Gilding M. Confidence at the expense of trust: the mass adoption of the Human Papillomavirus vaccine in Australia. Health Sociology Review. 2013;22(1):88–97.

[50] Carnegie E, Whittaker A, Gray Brunton C, Hogg R, Kennedy C, Hilton S, et al. Development of a cross-cultural HPV community engagement model within Scotland. Health Educ J. 2017;76(4):398–410. doi: 10.1177/0017896916685592 28596618 PMC5446167

[51] Chan CYZ, Lam CH, Lam DY, Lee LY, Ng KK, Wong ML. A qualitative study on HPV vaccination from a nursing perspective in Hong Kong. Asian Pac J Cancer Prev. 2011;12(10):2539–45. 22320952

[52] Chen AC-C, Astroth C, Reifsnider E, Yang H, Mao W, Chen H. Exploring Chinese college students’ hpv awareness, knowledge, attitudes, and intent of hpv vaccination: a qualitative study. J Cancer Educ. 2021;36(6):1211–8. doi: 10.1007/s13187-020-01750-0 32314310

[53] Clevenger L-M, Dreisbach S, Scandlyn J, Brett J. Access to Catch-Up HPV Vaccination among Latina University Students. Human Organization. 2012;71(1):44–53. doi: 10.17730/humo.71.1.r1x78526541441t1

[54] Cohen EL, Head KJ. Identifying knowledge-attitude-practice gaps to enhance HPV vaccine diffusion. J Health Commun. 2013;18(10):1221–34. doi: 10.1080/10810730.2013.778357 23767775

[55] Dai Z. What have we missed?: the knowledge of and access to the HPV Vaccine and Sex Education in China. Health Commun. 2020;35(1):96–8. doi: 10.1080/10410236.2018.1545168 30451533

[56] DeLauer V, McGill-O’Rourke A, Gordon C, Hamilton N, Desruisseaux R, DuarteCanela M, et al. Human papillomavirus and health decision-making: perceptions and accountability in college. Health Educ J. 2019;79(1):46–57. doi: 10.1177/0017896919862309

[57] Fields EJ, Hopfer S, Warren JR, BeLue R, Lebed J, Hecht ML. Motivators and barriers to hpv vaccination: a qualitative study of underserved women attending planned parenthood. Vaccines (Basel). 2022;10(7):1126. doi: 10.3390/vaccines10071126 35891290 PMC9317585

[58] Fontenot HB, Fantasia HC, Vetters R, Zimet GD. Increasing HPV vaccination and eliminating barriers: Recommendations from young men who have sex with men. Vaccine. 2016;34(50):6209–16. doi: 10.1016/j.vaccine.2016.10.075 27838067

[59] Garcia S, Hopfer S, Amaro H, Tanjasiri S. HPV vaccine delay and refusal among unvaccinated Mexican American young adult women: a qualitative investigation of Mexican-born and US-born HPV vaccine decision narratives. Journal of behavioral medicine. 2023;46(1):88-99. 35610490 10.1007/s10865-022-00326-1 PMC9130004

[60] Gerend MA, Madkins K, Crosby S, Korpak AK, Phillips GL 2nd, Bass M, et al. A Qualitative analysis of young sexual minority men’s perspectives on human papillomavirus vaccination. LGBT Health. 2019;6(7):350–6. doi: 10.1089/lgbt.2019.0086 31556791 PMC6797075

[61] Glenn BA, Nonzee NJ, Tieu L, Pedone B, Cowgill BO, Bastani R. Human papillomavirus (HPV) vaccination in the transition between adolescence and adulthood. Vaccine. 2021;39(25):3435–44. doi: 10.1016/j.vaccine.2021.04.019 33992435 PMC9063977

[62] Gray Brunton C, Farver I, Jäger M, Lenneis A, Parve K, Patarcic D, et al. Young women’s constructions of the hpv vaccine: a cross-cultural, qualitative study in scotland, spain, serbia and bulgaria. Int J Behav Med. 2014;21(1):11–9. doi: 10.1007/s12529-013-9357-3 24092427

[63] Head KJ, Cohen EL. Young women’s perspectives on cervical cancer prevention in appalachian kentucky. Qual Health Res. 2012;22(4):476–87. doi: 10.1177/1049732311425053 22068039

[64] Hirth JM, Batuuka DN, Gross TT, Cofie L, Berenson AB. Human papillomavirus vaccine motivators and barriers among community college students: considerations for development of a successful vaccination program. Vaccine. 2018;36(8):1032–7. doi: 10.1016/j.vaccine.2018.01.037 29366708 PMC5801161

[65] Hodge FS, Itty T, Cardoza B, Samuel-Nakamura C. HPV vaccine readiness among American Indian college students. Ethn Dis. 2011;21(4):415–20. 22428344

[66] Hodge F. American Indian male college students perception and knowledge of human papillomavirus (HPV). 2014; doi: DOIorotheridentifier

[67] Hopfer S, Clippard JR. College women’s HPV vaccine decision narratives. Qual Health Res. 2011;21(2):262–77. doi: 10.1177/1049732310383868 20841433

[68] Hopfer S, Garcia S, Duong HT, Russo JA, Tanjasiri SP. A narrative engagement framework to understand hpv vaccination among latina and vietnamese women in a planned parenthood setting. Health Educ Behav. 2017;44(5):738–47. doi: 10.1177/1090198117728761 28854812 PMC5741467

[69] Jaiswal J, LoSchiavo C, Maiolatesi A, Kapadia F, Halkitis PN. Misinformation, gendered perceptions, and low healthcare provider communication around hpv and the hpv vaccine among young sexual minority men in new york city: The p18 cohort study. J Community Health. 2020;45(4):702–11. doi: 10.1007/s10900-019-00784-w 32016677 PMC7774381

[70] Jin SW, Lee Y, Lee S, Jin H, Brandt HM. Factors Associated with college students’ human papillomavirus (HPV) vaccination and preferred strategies for catch-up vaccine promotion: a mixed-methods study. Vaccines (Basel). 2023;11(6):1124. doi: 10.3390/vaccines11061124 37376513 PMC10303150

[71] Pierre Joseph N, Clark JA, Mercilus G, Wilbur M, Figaro J, Perkins R. Racial and ethnic differences in HPV knowledge, attitudes, and vaccination rates among low-income African-American, haitian, latina, and caucasian young adult women. J Pediatr Adolesc Gynecol. 2014;27(2):83–92. doi: 10.1016/j.jpag.2013.08.011 24602302 PMC3950833

[72] Kim M, Lee H, Kiang P, Kim D. Human papillomavirus: qualitative study of korean american female college students’ attitudes toward vaccination. Clin J Oncol Nurs. 2017;21(5):E239–47. doi: 10.1188/17.CJON.E239-E247 28945722

[73] Kidder GW, Montgomery CW. Oxygenation of frog gastric mucosa in vitro. Am J Physiol. 1975;229(6):1510–3. doi: 10.1152/ajplegacy.1975.229.6.1510 2018

[74] Lee PWH, Kwan TTC, Tam KF, Chan KKL, Young PMC, Lo SST, et al. Beliefs about cervical cancer and human papillomavirus (HPV) and acceptability of HPV vaccination among Chinese women in Hong Kong. Prev Med. 2007;45(2–3):130–4. doi: 10.1016/j.ypmed.2007.07.013 17707077

[75] Lim ASE, Lim RBT. Facilitators and barriers of human papillomavirus vaccine uptake in young females 18-26 years old in Singapore: A qualitative study. Vaccine. 2019;37(41):6030–8. doi: 10.1016/j.vaccine.2019.08.053 31473002

[76] Mancuso F, Polzer J. “ It’s your body but.”: young women’s narratives of declining human papillomavirus (HPV) Vaccination. Canadian Woman Studies/les cahiers de la femme. 2010.

[77] Share JB. Review of drug treatment for Down’s syndrome persons. Am J Ment Defic. 1976;80(4):388–93. doi: 10.1108/09654281111180481 2011

[78] McClelland A, Liamputtong P. Knowledge and acceptance of human papillomavirus vaccination: perspectives of young Australians living in Melbourne, Australia. Sex Health. 2006;3(2):95–101. doi: 10.1071/sh05035 16800395

[79] McComb E, Ramsden V, Olatunbosun O, Williams-Roberts H. Knowledge, attitudes and barriers to human papillomavirus (HPV) vaccine uptake among an immigrant and refugee catch-up group in a western canadian province. J Immigr Minor Health. 2018;20(6):1424–8. doi: 10.1007/s10903-018-0709-6 29445898

[80] Mehta P, Sharma M, Lee RC. Using the health belief model in qualitative focus groups to identify HPV vaccine acceptability in college men. Int Q Community Health Educ. 2012;33(2):175–87. doi: 10.2190/IQ.33.2.f 23661418

[81] Miller-Day M, Craw E, Harris D, Hecht ML. Men’s stories: an account of translating vaccine decision narratives from young men in the U.S. into a targeted public health intervention. J Appl Communi Res. 2022;51(2):185–203. doi: 10.1080/00909882.2022.2099228

[82] Mills LA, Head KJ, Vanderpool RC. HPV vaccination among young adult women: a perspective from Appalachian Kentucky. Prev Chronic Dis. 2013;10:E17. doi: 10.5888/pcd10.120183 23391293 PMC3567923

[83] Morales-Campos DY, Snipes SA, Villarreal EK, Crocker LC, Guerrero A, Fernandez ME. Cervical cancer, human papillomavirus (HPV), and HPV vaccination: Exploring gendered perspectives, knowledge, attitudes, and cultural taboos among Mexican American adults. Ethn Health. 2021;26(2):206–24. doi: 10.1080/13557858.2018.1494821 29998738 PMC6330137

[84] Mortensen GL. Drivers and barriers to acceptance of human-papillomavirus vaccination among young women: a qualitative and quantitative study. BMC Public Health. 2010;10:68. doi: 10.1186/1471-2458-10-68 20152055 PMC2829006

[85] Nadarzynski T, Smith H, Richardson D, Pollard A, Llewellyn C. Perceptions of HPV and attitudes towards HPV vaccination amongst men who have sex with men: a qualitative analysis. Br J Health Psychol. 2017;22(2):345–61. doi: 10.1111/bjhp.12233 28191723

[86] Durbin RP. Letter: Acid secretion by gastric mucous membrane. Am J Physiol. 1975;229(6):1726. doi: 10.1152/ajplegacy.1975.229.6.1726 2020

[87] Pčolkina K, Sherman S, MOSS E, Redman C, Zodzika J, Rezeberga D. Barriers and motivators for uptake of cervical cancer prevention strategies in Eastern Europe: Perspective from Latvia. Acta Dermatovenerologica Alpina, Pannonica et Adriatica. 2019;87. 31545387

[88] Petrova D, Brunton CG, Jaeger M, Lenneis A, Munoz R, Garcia-Retamero R, et al. The Views of Young Women on HPV Vaccine Communication in Four European Countries. Curr HIV Res. 2015;13(5):347–58. doi: 10.2174/1570162x13666150511124743 26149158

[89] Pierre Joseph N, Belizaire M, Porter CL, Walsh JP, Esang M, Goff G, et al. Ethnic differences in perceived benefits and barriers to HPV vaccine acceptance: a qualitative analysis of young African American, Haitian, Caucasian, and Latino men. Clin Pediatr (Phila). 2014;53(2):177–85. doi: 10.1177/0009922813515944 24403292

[90] Kidder GW, Montgomery CW. Oxygenation of frog gastric mucosa in vitro. Am J Physiol. 1975;229(6):1510–3. doi: 10.1152/ajplegacy.1975.229.6.1510 2018

[91] Schwabbauer ML. Use of the latent image technique to develop and evaluate problem-solving skills. Am J Med Technol. 1975;41(12):457–62. doi: 10.1080/13691050802541674 2010

[92] Pratt R, Njau SW, Ndagire C, Chaisson N, Toor S, Ahmed N, et al. “We are Muslims and these diseases don’t happen to us”: A qualitative study of the views of young Somali men and women concerning HPV immunization. Vaccine. 2019;37(15):2043–50. doi: 10.1016/j.vaccine.2019.03.006 30871929

[93] Berkmen YM, Lande A. Chest roentgenography as a window to the diagnosis of Takayasu’s arteritis. Am J Roentgenol Radium Ther Nucl Med. 1975;125(4):842–6. doi: 10.2214/ajr.125.4.842 2023

[94] Rahim MQ, Jacob SA, Coven SL, Miller M, Meagher CG, Lozano G, et al. Identifying Barriers to HPV vaccination for patients with sickle cell disease and childhood cancer survivors. J Pediatr Hematol Oncol. 2023;45(8):e940–7. doi: 10.1097/MPH.0000000000002752 37696002 PMC10615738

[95] Poole-Wilson PA, Langer GA. Effect of pH on ionic exchange and function in rat and rabbit myocardium. Am J Physiol. 1975;229(3):570–81. doi: 10.1152/ajplegacy.1975.229.3.570 2014

[96] Ross A, Sadler L, Brabin L. Developing a model of human papillomavirus vaccine acceptance among older teenagers. Int J Cancer Res and Preve. 2010;3:187–94.

[97] Salad J, Verdonk P, de Boer F, Abma TA. “A Somali girl is Muslim and does not have premarital sex. Is vaccination really necessary?” A qualitative study into the perceptions of Somali women in the Netherlands about the prevention of cervical cancer. Int J Equity Health. 2015;14:68. doi: 10.1186/s12939-015-0198-3 26293806 PMC4546144

[98] Poole-Wilson PA, Langer GA. Effect of pH on ionic exchange and function in rat and rabbit myocardium. Am J Physiol. 1975;229(3):570–81. doi: 10.1152/ajplegacy.1975.229.3.570 2014

[99] Schwendener CL, Kiener LM, Jafflin K, Rouached S, Juillerat A, Meier V, et al. HPV vaccine awareness, knowledge and information sources among youth in Switzerland: a mixed methods study. BMJ Open. 2022;12(1):e054419. doi: 10.1136/bmjopen-2021-054419 35105636 PMC8808397

[100] Shetty S, Shetty V, Badiger S, Shetty AK. An exploratory study of undergraduate healthcare student perspectives regarding human papillomavirus and vaccine intent in India. Womens Health (Lond). 2021;17:17455065211055304. doi: 10.1177/17455065211055304 34713762 PMC8558803

[101] Siu JY. Barriers to receiving human papillomavirus vaccination among female students in a university in Hong Kong. Cult Health Sex. 2013;15(9):1071–84. doi: 10.1080/13691058.2013.807518 23826650

[102] Stephens DP, Thomas TL. Social networks influence hispanic college women’s HPV vaccine uptake decision-making processes. Womens Reprod Health (Phila). 2014;1(2):120–37. doi: 10.1080/23293691.2014.966034 25599082 PMC4295831

[103] Stephens DP, Tamir H, Thomas TL. Factors motivating HPV vaccine uptake among vaccinated and nonvaccinated hispanic young adult women. Hisp Health Care Int. 2016;14(4):184–91. doi: 10.1177/1540415316679808 27913692

[104] Sullivan-Blum ZC, Brophy M, Didde R, Nagireddy R, Swagerty H, Weir S, et al. PrEP patient attitudes, beliefs and perceived barriers surrounding HPV vaccination: a qualitative study of semistructured interviews with PrEP patients in primary care clinics in Kansas and Missouri. BMJ Open. 2022;12(4):e058510. doi: 10.1136/bmjopen-2021-058510 35379639 PMC8981353

[105] Teitelman AM, Kim SK, Waas R, DeSenna A, Duncan R. Development of the nowiknow mobile application to promote completion of hpv vaccine series among young adult women. J Obstet Gynecol Neonatal Nurs. 2018;47(6):844–52. doi: 10.1016/j.jogn.2018.06.001 30036509

[106] Thompson EL, Vamos CA, Straub DM, Sappenfield WM, Daley EM. “We’ve been together. We don’t have it. We’re fine.” How relationship status impacts human papillomavirus vaccine behavior among young adult women. Womens Health Issues. 2017;27(2):228–36. doi: 10.1016/j.whi.2016.09.011 28277236

[107] Thompson EL, Vamos CA, Straub DM, Sappenfield WM, Daley EM. Human papillomavirus vaccine information, motivation, and behavioral skills among young adult US women. J Health Psychol. 2018;23(14):1832–41. doi: 10.1177/1359105316672924 28810358

[108] Tu Y-C, Wang H-H. An exploration of human papillomavirus-related cervical cancer prevention experiences among college women: a descriptive qualitative approach. J Clin Nurs. 2013;22(23–24):3300–9. doi: 10.1111/jocn.12051 23521597

[109] Waters A, Mann K, Vaca Lopez P, Kepka D, Wu Y, Kirchhoff A. HPV vaccine experiences and preferences among young adult cancer survivors and caregivers of childhood cancer survivors. Journal of Cancer Education. 2021;1:1–6. 10.1007/s13187-021-01992-6 PMC8454692 33740231

[110] Wheldon CW, Daley EM, Buhi ER, Baldwin JA, Nyitray AG, Giuliano AR. HPV vaccine decision-making among young men who have sex with men. Health Educ J. 2017;76(1):52–65. doi: 10.1177/0017896916647988 39371403 PMC11451994

[111] Wong LP. Young multiethnic women’s attitudes toward the HPV vaccine and HPV vaccination. Int J Gynaecol Obstet. 2008;103(2):131–5. doi: 10.1016/j.ijgo.2008.07.005 18768178

[112] Wong LP. HPV information needs, educational messages and channel of delivery preferences: views from developing country with multiethnic populations. Vaccine. 2009;27(9):1410–5. doi: 10.1016/j.vaccine.2008.12.045 19150379

[113] Wyndham-West CM. ‘It’s really complicated’: how canadian university women students navigate gendered risk and human papillomavirus (HPV) vaccine decision-making. Health, Risk & Society. 2016;18(1–2):59–76. doi: 10.1080/13698575.2016.1176127

[114] Yarmohammadi S, Ghaffari M, Mashayekhi P, Ramezankhani A, Mirzaei J. Strategies for improving participation in human papillomavirus vaccination among young adults in the capital of iran: A qualitative-exploratory study. Int J Prev Med. 2022;13:1. doi: 10.4103/ijpvm.IJPVM_599_20 35281978 PMC8883668

[115] Young A, Cotton S, Cruickshank ME. Information needs of young women vaccinated against HPV attending colposcopy: a qualitative study. BMC Womens Health. 2018;18(1):200. doi: 10.1186/s12905-018-0691-0 30541542 PMC6292045

[116] World Bank. World Bank Country and Lending Groups [cited 2024 June 30]. Available from: https://datahelpdesk.worldbank.org/knowledgebase/articles/906519-world-bank-country-and-lending-groups.

[117] US Department of Health and Human Services. Theory at a glance: A guide for health promotion practice: Lulu. com; 2018.

[118] Meites E, Szilagyi PG, Chesson HW, Unger ER, Romero JR, Markowitz LE. Human papillomavirus vaccination for adults: Updated recommendations of the advisory committee on immunization practices. MMWR Morb Mortal Wkly Rep. 2019;68(32):698–702. doi: 10.15585/mmwr.mm6832a3 31415491 PMC6818701

[119] Sung H, Ferlay J, Siegel RL, Laversanne M, Soerjomataram I, Jemal A, et al. Global cancer statistics 2020: Globocan estimates of incidence and mortality worldwide for 36 cancers in 185 countries. CA Cancer J Clin. 2021;71(3):209–49. doi: 10.3322/caac.21660 33538338

[120] World Health Organization. Immunization, Vaccines and Biologicals. 2023. https://www.who.int/teams/immunization-vaccines-and-biologicals/diseases/human-papillomavirus-vaccines-(HPV)/hpv-clearing-house/hpv-dashboard

[121] Phongsamart W, Lou P-J, Sukarom I, Wu Y-H, Zaidi O, Du F, et al. Integrative literature review on human papillomavirus vaccination recommendations in national immunization programs in select areas in the Asia-Pacific region. Hum Vaccin Immunother. 2024;20(1):2362449. doi: 10.1080/21645515.2024.2362449 38925146 PMC11210899

[122] Hull B, Hendry A, Dey A, Brotherton J, Macartney K, Beard F. Annual immunisation coverage report 2020. Commun Dis Intell (2018). 2022;46:10.33321/cdi.2022.46.60. doi: 10.33321/cdi.2022.46.60 36154654

[123] Thompson VLS, Butler-Barnes ST, Jones BD, Wells AA, Cunningham-Williams RM, Williams S-L. Factors associated with human papillomavirus vaccination status at U.S. colleges and universities. Health Soc Work. 2017;42(1):e1–7. doi: 10.1093/hsw/hlw050 28395066

[124] Turner JC, Keller A. College health surveillance network: epidemiology and health care utilization of college students at us 4-year universities. J Am Coll Health. 2015;63(8):530–8. doi: 10.1080/07448481.2015.1055567 26086428 PMC4673518

[125] Barraza L, Hodge JG Jr, Gulinson CL, Hensley D, Castagne M. Immunization Laws and Policies Among U.S. Institutes of Higher Education. J Law Med Ethics. 2019;47(2):342–6. doi: 10.1177/1073110519857292 31298094

[126] Anhang Price R, Tiro JA, Saraiya M, Meissner H, Breen N. Use of human papillomavirus vaccines among young adult women in the United States: an analysis of the 2008 national health interview survey. Cancer. 2011;117(24):5560–8. doi: 10.1002/cncr.26244 21732336 PMC3189421

[127] Benavidez G, Asare M, Lanning B, Ylitalo K, Fakhoury C, Thompson N, et al. Young adults’ human papillomavirus-related knowledge: Source of medical information matters. Public Health. 2020;182:125–30. doi: 10.1016/j.puhe.2020.01.020 32272289

[128] Rosenthal SL, Weiss TW, Zimet GD, Ma L, Good MB, Vichnin MD. Predictors of HPV vaccine uptake among women aged 19-26: importance of a physician’s recommendation. Vaccine. 2011;29(5):890–5. doi: 10.1016/j.vaccine.2009.12.063 20056186

[129] Cartmell KB, Mzik CR, Sundstrom BL, Luque JS, White A, Young-Pierce J. HPV vaccination communication messages, messengers, and messaging strategies. J Cancer Edu. 2019;34(5):1014–23. doi: 10.1007/s13187-018-1405-x PubMed -61491-023. 30054900 PMC9673969

