# Supplementary material for: Perspectives of HPV vaccine decision-making among young adults: A qualitative systematic review and evidence synthesis
Source: PLoS One. 2025 May 5;20(5):e0321448. doi: 10.1371/journal.pone.0321448 (PMC12052141; doi:10.1371/journal.pone.0321448)
Supplement: S1 Appendix — (DOCX) [file pone.0321448.s001.docx]

| **Database** | **Search Terms** |
| --- | --- |
| PubMed | ("human papillomavirus viruses"[MeSH Terms] OR "human papillomavirus*" OR "human papilloma virus*" OR "hpv") AND ("young adult"[MeSH Terms] OR "young adult*" OR "emerging adult*" OR "legal adult*" OR "older adolescen*" OR "late adolescen*" OR "youth*" OR "student*" OR “young person*” OR “young people”) AND ("vaccination"[MeSH Terms] OR "vaccines"[MeSH Terms] OR "vaccin*" OR "immunis*" OR "immuniz*") AND ("qualitative research"[MeSH Terms] OR "interviews as topic"[MeSH Terms] OR "focus groups"[MeSH Terms] OR "mixed method*"[tiab] OR "qualitativ*"[tiab] OR "focus group*"[tiab] OR "interview*"[tiab] OR "conversation*"[tiab] OR "observation*"[tiab] OR "photovoice"[tiab] OR "themes"[tiab] OR "thematic"[tiab] OR "narrative*"[tiab] OR "grounded theory"[tiab] OR "content analysis"[tiab] OR "ethnography"[tiab] OR “phenomenolog*”[tiab]) AND 2006/01/01:3000/12/31[Date - Publication] |
| SCOPUS (Elsevier) | ( TITLE-ABS-KEY ( "human papillomavirus*" OR "human papilloma virus*" OR "hpv" ) AND TITLE-ABS-KEY ( "young adult*" OR "emerging adult*" OR "legal adult*" OR "older adolescen*" OR "late adolescen*" OR "youth*" OR "student*" OR "young person**" OR "young people*" ) AND TITLE-ABS-KEY ( "vaccin*" OR "immunis*" OR "immuniz*" ) AND TITLE-ABS-KEY ( "mixed method*" OR "qualitativ*" OR "focus group*" OR "interview*" OR "conversation*" OR "observation*" OR "photovoice" OR "themes" OR "thematic" OR "narrative*" OR "grounded theory" OR "content analysis" OR "ethnography" OR "phenomenolog*" ) ) AND PUBYEAR > 2005 |
| Embase (Elsevier) | ('wart virus'/syn OR 'wart virus' OR 'human papillomavirus*' OR 'human papilloma virus*' OR 'hpv'/exp OR 'hpv') AND ('young adult'/syn OR 'young adult' OR 'student/syn' OR 'young adult*' OR 'emerging adult*' OR 'legal adult*' OR 'youth*' OR 'student*' OR ‘young person’ OR ‘young people’) AND ('vaccine'/exp OR 'vaccine' OR 'vaccin*' OR 'immunis*' OR 'immuniz*') AND ('qualitative research'/syn OR 'qualitative research' OR 'qualitative analysis'/exp OR 'qualitative analysis' OR 'interview'/syn OR 'interview' OR 'conversation'/exp OR 'conversation' OR 'thematic analysis'/exp OR 'thematic analysis' OR 'narrative'/exp OR 'narrative' OR 'mixed method*' OR 'qualitativ*' OR 'focus group*' OR 'interview*' OR 'conversation*' OR 'observation*' OR 'photovoice'/exp OR 'photovoice' OR themes OR thematic OR 'narrative*' OR 'grounded theory'/exp OR 'grounded theory' OR 'content analysis'/exp OR 'content analysis' OR 'ethnography'/exp OR 'ethnography' OR 'phenomenology'/exp OR 'phenomenology') AND [2006-2023]/py |
| Cochrane Library (Wiley) | ("human papillomavirus*” OR "human papilloma virus*” OR "hpv"):ti,ab,kw AND ("young adult*" OR "emerging adult*" OR "legal adult*" OR “older adolescen*” OR “late adolescen*” OR “youth*” OR “student*” OR "young person*" OR "young people*"):ti,ab,kw AND (vaccin* OR immunis* OR immuniz*):ti,ab,kw AND (mixed method* OR qualitativ* OR focus group* OR interview* OR conversation* OR observation* OR photovoice OR Themes OR Thematic OR narrative* OR grounded theory OR content analysis OR ethnography OR phenomenolog*):ti,ab,kw" |
| PsycINFO (EBSCOhost) | ( (DE "Human Papillomavirus") OR "human papillomavirus*” OR "human papilloma virus*” OR "hpv" ) AND ( (DE "Emerging Adulthood") OR (DE "Late Adolescence") OR "young adult*" OR "emerging adult*" OR "legal adult*" OR “older adolescen*” OR “late adolescen*” “youth*” OR “student*” OR "young person*" OR "young people*" ) AND ( (DE "Vaccine Attitudes") OR (DE "Vaccination") OR (DE "Immunization") OR "vaccin*" OR "immunis*" OR "immuniz*” ) AND ( (DE "Qualitative Methods") OR (DE "Qualitative Measures") OR (DE "Thematic Analysis") OR (DE "Participant Observation") OR (DE "Narrative Analysis") OR (DE "Mixed Methods Research") OR (DE "Interpretative Phenomenological Analysis") OR (DE "Grounded Theory") OR (DE "Focus Group") OR (DE "Content Analysis") OR (DE "Semi-Structured Interview") OR (DE “Interviews”) OR (DE “Observation Methods”) OR (DE “Ethnography”) OR "mixed method*” OR “qualitativ*” OR "focus group*" OR “interview*” OR “conversation*” OR “observation*” OR “photovoice” OR Themes OR Thematic OR “narrative*” OR “grounded theory” OR “content analysis” OR “ethnography” )  Limiter: Published Date: 20060101-20231231 |
| CINAHL (EBSCOhost) | ( (MH "Papillomaviruses") OR "human papillomavirus*” OR "human papilloma virus*” OR "hpv" ) ) AND ( (MH "Young Adult") OR "young adult*" OR "emerging adult*" OR "legal adult*" OR “older adolescen*” OR “late adolescen*” OR “youth*” OR “student*” OR "young person*" OR "young people*" ) AND ( ( (MH "Papillomavirus Vaccine") OR (MH "Vaccines") OR (MH "Immunization") OR (MH "Attitude to Vaccines") OR "vaccin*" OR "immunis*" OR "immuniz*” ) AND ( (MH "Qualitative Studies") OR (MH "Grounded Theory") OR (MH "Narratives") OR (MH "Observational Methods") OR (MH "Interviews") OR (MH "Content Analysis") OR (MH "Conversation") OR (MH "Thematic Analysis") OR (MH "Ethnographic Research") OR (MH "Focus Groups") OR (MH "Phenomenology") OR (MH "Phenomenological Research") OR "mixed method*” OR “qualitativ*” OR "focus group*" OR “interview*” OR “conversation*” OR “observation*” OR “photovoice” OR Themes OR Thematic OR “narrative*” OR “grounded theory” OR “content analysis” OR “ethnography” )  Limiters: Published Date: 20060101-20231231 |
| CABI Global Health (EBSCOhost) | ( "human papillomavirus*” OR "human papilloma virus*” OR "hpv" ) AND ( (DE "young adults”) OR "young adult*" OR "emerging adult*" OR "legal adult*" OR “older adolescen*” OR “late adolescen*” OR “youth*” OR “student*” OR "young person*" OR "young people*" ) AND ( (DE "Vaccine Attitudes") OR (DE "vaccination") OR "vaccin*" OR "immunis*" OR "immuniz*” ) AND ( (DE "qualitative techniques") OR (DE "qualitative analysis") OR (DE "interviews") OR "mixed method*” OR “qualitativ*” OR "focus group*" OR “interview*” OR “conversation*” OR “observation*” OR “photovoice” OR Themes OR Thematic OR “narrative*” OR “grounded theory” OR “content analysis” OR “ethnography” OR "phenomenolog*" )  Limiter: Publication Year: 20060101-20231231 |
