# Supplementary material for: Perspectives of HPV vaccine decision-making among young adults: A qualitative systematic review and evidence synthesis
Source: PLoS One. 2025 May 5;20(5):e0321448. doi: 10.1371/journal.pone.0321448 (PMC12052141; doi:10.1371/journal.pone.0321448)
Supplement: S2 Appendix — . (DOCX) [file pone.0321448.s002.docx]

| **Step 1** | Search rerun in all the databases with updated terms |
| --- | --- |
| **Step 2** | All database search files imported and deduplicated in EndNote   - 2909 records retrieved from 7 databases - 1464 duplicates removed = 1445 records for screening - Copy of EndNote Library file containing updated duplicates search results saved for reference |
| **Step 3** | Old deduplicated search results from the original search (1306 records) copied to EndNote containing updated deduplicates search results |
| **Step 4** | Identify and remove duplicate records by iteratively adjusting EndNote’s filter settings to identify duplicates   - Filter 1: Author, Year, Title, Secondary Title (Journal) 🡪 2600 duplicate removed - Filter 2: Author, Year, Title, Pages fields 🡪 6 duplicates removed - Filter 3: Title 🡪 3 duplicates removed |
| **Step 5** | The remaining results (142 records) represented the unique new studies from the updated search. The EndNote file with these results was imported to DistillerSR for review screening |
