# Supplementary material for: Perspectives of HPV vaccine decision-making among young adults: A qualitative systematic review and evidence synthesis
Source: PLoS One. 2025 May 5;20(5):e0321448. doi: 10.1371/journal.pone.0321448 (PMC12052141; doi:10.1371/journal.pone.0321448)
Supplement: S3 Appendix — . (DOCX) [file pone.0321448.s003.docx]

| **#** | **Summarized review finding** | **Methodological limitations** | **Coherence** | **Adequacy** | **Relevance** | **GRADE-CERQual assessment of confidence** | **References** |
| --- | --- | --- | --- | --- | --- | --- | --- |
| **THEME 1: INDIVIDUAL FACTORS** | | | | | | | |
| 1 | Missed opportunity: Some young adults believed they missed the window for vaccination because they were too old to benefit from it. | **Minor concerns**  4 studies with minor to moderate concerns primarily due to lack of reflexivity. | **No/Very minor concerns** | **Minor concerns**  5 studies contributed to this finding offering superficial data. However, the finding is simple and descriptive, and the supporting data provides sufficient information to understand the meaning/context. | **No/Very minor concerns** | **Moderate confidence**  5 studies reflecting minor concerns regarding methodological limitations, and minor concerns regarding adequacy. | Hodge et al. 2011; Jaiswal et al. 2020; Kim 2017; Tu & Wang 2013; Wheldon et al. 2017; |
| 2 | Risk perception: Some young adults believed they were not at risk for HPV-related illnesses or that they still had time to decide on vaccination. | **Minor concerns**  4 studies with minor to moderate concerns due to lack of reflexivity. 1 study with moderate concern due to unclear sampling strategy | **Minor concerns**  While there is evidence of variation in risk perception within the data, the finding highlights a key pattern observed in the data. | **Minor concerns**  7 studies contributed to this finding offering superficial data. However, the finding is simple and descriptive, and the supporting data provides sufficient information to understand the meaning/context. | **No/Very minor concerns** | **Moderate confidence**  7 studies reflecting minor concerns regarding methodological limitations, minor concerns regarding coherence, and minor concerns regarding adequacy. | Allen et al. 2009; Hodge 2014; Hopfer & Clippard 2011; Lim & Lim 2019; Miller-Day et al. 2023; Siu 2013; Wheldon et al. 2017; |
| 3 | Proactive measure: Young adults viewed vaccination as a proactive measure to safeguard their health in the present and prevent future regret if they didn’t get vaccinated. | **Minor concerns**  11 studies with minor to moderate concerns primarily due to lack of reflexivity. | **No/Very minor concerns** | **No/Very minor concerns** | **No/Very minor concerns** | **High confidence**  13 studies reflecting minor concerns regarding methodological limitations. | Apaydin et al. 2018; Chen et al. 2021; Gerend et al. 2019; Hirth et al. 2018; Hopfer & Clippard 2011; Joseph et al. 2014; McClelland & Liamputtong 2006; Pierre Joseph et al. 2014; Shetty et al. 2021; Stephens et al. 2016; Thompson et al. 2017; Wheldon et al. 2017; Wyndham-West 2016; |
| 4 | Low-risk preventive measure: Several individuals mention the simplicity of the decision to get vaccinated, seeing it as a low-risk preventive measure with potentially significant benefits. | **Minor concerns**  5 studies with minor to moderate concerns primarily due to lack of reflexivity. | **No/Very minor concerns** | **Moderate concerns**  5 studies contributed to this finding offering thin and superficial data, which limited a deeper understanding of the phenomenon | **No/Very minor concerns** | **Low confidence**  5 studies reflecting minor concerns regarding methodological limitations, and moderate concerns regarding adequacy. | Cohen & Head 2013; Hirth et al. 2018; Kim 2017; Lim & Lim 2019; McClelland & Liamputtong 2006; Pierre Joseph et al. 2014; |
| 5 | Health history of family and friends was a motivating factor in the decision to get vaccinated. | **Minor concerns**  4 studies with minor to moderate concerns primarily due to lack of reflexivity. | **No/Very minor concerns** | **Minor concerns**  5 studies contributed to this finding offering thin and superficial data. However, the finding is simple and descriptive, and the supporting data provides sufficient information to understand the meaning/context. | **No/Very minor concerns** | **Moderate confidence**  5 studies reflecting minor concerns regarding methodological limitations and minor concerns regarding adequacy. | Chen et al. 2021; Head & Cohen 2012; Pierre Joseph et al. 2014; Stephens et al. 2016; Wyndham-West 2016; |
| **THEME 2: ALTERNATIVES TO VACCINATION** | | | | | | | |
| 6 | Reliance on pap smears: Young adult women believed that regular Pap testing negated the need for HPV vaccination. | **Moderate concerns**  2 studies with minor to moderate concerns primarily due to lack of reflexivity. 1 study with moderate to severe concerns due to lack of sampling strategy and ethical consideration. | **No/Very minor concerns** | **Minor concerns**  4 studies contributed to this finding offering thin and superficial data. However, the finding is simple and descriptive, and the supporting data provides sufficient information to understand the meaning/context. | **No/Very minor concerns** | **Moderate confidence**  4 studies reflecting moderate concerns regarding methodological limitations, and minor concerns regarding adequacy. | Hodge et al. 2011; Mancuso & Polzer 2010; Petrova et al. 2015; Tu & Wang 2013; |
| 7 | Condom use and other precautions: Young adults expressed precautions such as condom use, abstinence, and decisions on sexual partners were sufficient to protect against HPV, rendering the vaccine unnecessary. | **Minor concerns**  5 studies with minor to moderate concerns primarily due to lack of reflexivity. | **No/Very minor concerns** | **Minor concerns**  6 studies contributed to this finding offering superficial data. However, the finding is simple and descriptive, and the supporting data provides sufficient information to understand the meaning/context. | **No/Very minor concerns** | **Moderate confidence**  6 studies reflecting minor concerns regarding methodological limitations, and minor concerns regarding adequacy. | Cohen & Head 2013; Hopfer & Clippard 2011; Miller-Day et al. 2023; Pierre Joseph et al. 2014; Thompson et al. 2017; Tu & Wang 2013; |
| **THEME 3: KNOWLEDGE AND INFORMATION** | | | | | | | |
| 8 | Many young adults expressed having insufficient information on HPV or the vaccine before deciding to get vaccinated. There was an expressed desire more information about HPV and the HPV vaccine. | **Minor concerns**  12 studies with minor to moderate concerns primarily due to lack of reflexivity. | **No/Very minor concerns** | **No/Very minor concerns** | **No/Very minor concerns** | **High confidence**  13 studies reflecting minor concerns regarding methodological limitations. | Cohen & Head 2013; Fontenot et al. 2016; Garcia et al. 2023; Gray Brunton et al. 2014; Head & Cohen 2012; Hirth et al. 2018; Hodge 2014; Hodge et al. 2011; Joseph et al. 2014; Kim 2017; McClelland & Liamputtong 2006; Miller-Day et al. 2023; Pierre Joseph et al. 2014; |
| **THEME 4: SEX AND ROMANTIC RELATIONSHIPS** | | | | | | | |
| 9 | Sexual activity and behavior: Some individuals believe that vaccination against HPV is only necessary if one plans to engage in sexual activity or has many sexual partners. | **Minor concerns**  9 studies with minor to moderate concerns primarily due to lack of reflexivity. | **No/Very minor concerns** | **No/Very minor concerns** | **No/Very minor concerns** | **High confidence**  12 studies reflecting minor concerns regarding methodological limitations. | Cohen & Head 2013; Garcia et al. 2023; Head & Cohen 2012; Hopfer & Clippard 2011; Joseph et al. 2014; Lim & Lim 2019; Miller-Day et al. 2023; Petrova et al. 2015; Siu 2013; Stephens et al. 2016; Thompson et al. 2017; Wheldon et al. 2017; |
| 10 | Relationship status and sexual history influenced risk perception among young adults. | **Minor concerns**  6 studies with minor to moderate concerns primarily due to lack of reflexivity. | **No/Very minor concerns** | **No/Very minor concerns** | **No/Very minor concerns** | **High confidence**  7 studies reflecting minor concerns regarding methodological limitations. | Hopfer & Clippard 2011; McClelland & Liamputtong 2006; Mehta et al. 2013; Miller-Day et al. 2023; Pierre Joseph et al. 2014; Thompson et al. 2017; Thompson et al. 2018; |
| 11 | Parental beliefs about sex and the HPV vaccine influenced young adults’ vaccine attitudes and decisions. | **Minor concerns**  4 studies with minor to moderate concerns primarily due to lack of reflexivity. | **Minor concerns**  While there is evidence of some variation in in parents' beliefs about sex and the HPV vaccine, the finding highlights a key pattern observed in the data. | **Minor concerns**  6 studies contributed to this finding offering superficial data. However, the finding is simple and descriptive, and the supporting data provides sufficient information to understand the meaning/context. | **No/Very minor concerns** | **Moderate confidence**  6 studies reflecting minor concerns regarding methodological limitations, minor concerns regarding coherence, minor concerns regarding adequacy. | Fontenot et al. 2016; Hodge et al. 2011; Hopfer & Clippard 2011; McComb et al. 2018; Shetty et al. 2021; Siu 2013; |
| **THEME 5: PARENTS AND PEERS** | | | | | | | |
| 12 | Mothers more than fathers played an active role in HPV vaccination process among young adults. | Minor concerns  9 studies with minor to moderate concerns primarily due to lack of reflexivity. | No/Very minor concerns | **No/Very minor concerns** | **No/Very minor concerns** | **High confidence**  12 studies reflecting minor concerns regarding methodological limitations. | Basnyat & Lim 2018; Cohen & Head 2013; Garcia et al. 2023; Gerend et al. 2019; Hirth et al. 2018; Hopfer & Clippard 2011; Kim 2017; Lim & Lim 2019; McClelland & Liamputtong 2006; Miller-Day et al. 2023; Ross et al. 2010; Stephens & Thomas 2014; |
| 13 | Decision-making power: There was a mix of deferred or shared the decision to get vaccinated between young adults and parents. | **Minor concerns**  4 studies with minor to moderate concerns primarily due to lack of reflexivity. | **Minor concerns**  The way some of the data is presented and framed provides plausibility that some young adults may independently decide on vaccination without parental inclusion. | **Minor concerns**  5 studies contributed to this finding offering thin and superficial data. However, the finding is simple and descriptive, and the supporting data provides sufficient information to understand the meaning/context. | **No/Very minor concerns** | **Moderate confidence**  5 studies reflecting minor concerns regarding methodological limitations, minor concerns regarding coherence, and minor concerns regarding adequacy. | Cohen & Head 2013; Hirth et al. 2018; Miller-Day et al. 2023; Mills et al. 2013; Stephens et al. 2016; |
| 14 | Peer influence: Friends and acquaintances played a significant role in shaping vaccination decisions. | **Minor concerns**  7 studies with minor to moderate concerns primarily due to lack of reflexivity. | **No/Very minor concerns** | **Minor concerns**  7 studies contributed to this finding offering superficial data. However, the finding is simple and descriptive, and the supporting data provides sufficient information to understand the meaning/context. | **No/Very minor concerns** | **Moderate confidence**  Minor concerns regarding methodological limitations, No/Very minor concerns regarding coherence, Minor concerns regarding adequacy, and No/Very minor concerns regarding relevance | Basnyat & Lim 2018; Cohen & Head 2013; Head & Cohen 2012; Hopfer & Clippard 2011; Ross et al. 2010; Siu 2013; Thompson et al. 2018; Wheldon et al. 2017; |
| **THEME 6: PHYSICIANS** | | | | | | | |
| 15 | Doctor recommendation: Physician recommendations (positive or negative) were influential in vaccination uptake. | **Minor concerns**  17 with minor to moderate concerns primarily due to lack of reflexivity. | **No/Very minor concerns** | **No/Very minor concerns** | **No/Very minor concerns** | **High confidence**  18 studies reflecting minor concerns regarding methodological limitations. | Apaydin et al. 2018; Chan et al. 2011; Clevenger et al. 2012; Cohen & Head 2013; Gerend et al. 2019; Hirth et al. 2018; Hopfer & Clippard 2011; Jaiswal et al. 2020; Joseph et al. 2014; McComb et al. 2018; Miller-Day et al. 2023; Pierre Joseph et al. 2014; Ross et al. 2010; Stephens & Thomas 2014; Thompson et al. 2017; Thompson et al. 2018; Wheldon et al. 2017; Wyndham-West 2016; |
| 16 | Communication gap with doctors: Young adults desired better communication and more informed conversations with their doctors. | **Minor concerns**  9 studies with minor to moderate concerns primarily due to lack of reflexivity. | **No/Very minor concerns** | **No/Very minor concerns**  9 studies contributed to this finding offering rich and detailed data to understand and contextualize this phenomenon | **No/Very minor concerns** | **High confidence**  9 studies reflecting minor concerns regarding methodological limitations. | Apaydin et al. 2018; Fontenot et al. 2016; Garcia et al. 2023; Jaiswal et al. 2020; Mills et al. 2013; Petrova et al. 2015; Pierre Joseph et al. 2014; Siu 2013; Wheldon et al. 2017; |
| **THEME 7: LOGISTICS** | | | | | | | |
| 17 | Appointments: Scheduling and appointment challenges impacted young adults’ ability to get vaccinated. | **Minor concerns**  9 studies with minor to moderate concerns primarily due to lack of reflexivity. | **No/Very minor concerns** | **Minor concerns**  10 studies contributed to this finding offering superficial data. However, the finding is simple and descriptive, and the supporting data provides sufficient information to understand the meaning/context. | **No/Very minor concerns** | **Moderate confidence**  10 studies reflecting minor concerns regarding methodological limitations and minor concerns regarding adequacy. | Apaydin et al. 2018; Fontenot et al. 2016; Head & Cohen 2012; Hirth et al. 2018; Jaiswal et al. 2020; Mills et al. 2013; Pierre Joseph et al. 2014; Stephens & Thomas 2014; Stephens et al. 2016; Teitelman et al. 2018; |
| 18 | Multiple shots: Some young adults found the process of going back multiple times for the three doses burdensome, leading to forgetfulness or avoidance. | **Minor concerns**  7 studies with minor to moderate concerns primarily due to lack of reflexivity. | **No/Very minor concerns** | **Minor concerns**  8 studies contributed to this finding offering superficial data. However, the finding is simple and descriptive, and the supporting data provides sufficient information to understand the meaning/context. | **No/Very minor concerns** | **Moderate confidence**  8 studies reflecting minor concerns regarding methodological limitations and minor concerns regarding adequacy. | Apaydin et al. 2018; Head & Cohen 2012; Hopfer & Clippard 2011; Jaiswal et al. 2020; Lim & Lim 2019; Miller-Day et al. 2023; Ross et al. 2010; Teitelman et al. 2018; |
| 19 | Accessibility: Transportation and access challenges hindered HPV vaccination uptake. | **Moderate concerns**  4 studies with minor to moderate concerns primarily due to lack of reflexivity. 1 study with severe concerns due to lack of clear research statement, sampling strategy, analysis plan, and ethical consideration. | **No/Very minor concerns** | **Minor concerns**  8 studies contributed to this finding offering superficial data. However, the finding is simple and descriptive, and the supporting data provides sufficient information to understand the meaning/context. | **No/Very minor concerns** | **Moderate confidence**  8 studies reflecting moderate concerns regarding methodological limitations and minor concerns regarding adequacy. | Dai 2020; Hirth et al. 2018; Hodge et al. 2011; Hopfer & Clippard 2011; Lim & Lim 2019; Mills et al. 2013; Ross et al. 2010; Wheldon et al. 2017; |
| 20 | Reminders: Reminders were cited as helpful in ensuring adherence to the vaccination schedule. | **Minor concerns**  4 studies with minor to moderate concerns primarily due to lack of reflexivity. | **No/Very minor concerns** | **Minor concerns**  5 studies contributed to this finding offering superficial data. However, the finding is simple and descriptive, and the supporting data provides sufficient information to understand the meaning/context. | **No/Very minor concerns** | **Moderate confidence**  5 studies reflecting minor concerns regarding methodological limitations ad minor concerns regarding adequacy. | Apaydin et al. 2018; Hirth et al. 2018; Hopfer & Clippard 2011; Miller-Day et al. 2023; Ross et al. 2010; |
| 21 | Mandates: Young adults had mixed views on mandates for HPV vaccination. | **Minor concerns**  6 studies with minor to moderate concerns primarily due to lack of reflexivity. | **No/Very minor concerns** | **Minor concerns**  7 studies contributed to this finding offering superficial data. However, the finding is simple and descriptive, and the supporting data provides sufficient information to understand the meaning/context. | **No/Very minor concerns** | **Moderate confidence**  7 studies reflecting minor concerns regarding methodological limitations and minor concerns regarding adequacy. | Hirth et al. 2018; Joseph et al. 2014; Lim & Lim 2019; Miller-Day et al. 2023; Pierre Joseph et al. 2014; Pratt et al. 2019; Shetty et al. 2021; |
| **THEME 8: THE VACCINE** | | | | | | | |
| 22 | Vaccine cost: Young adults expressed concerns about paying for the HPV vaccine, citing high out-of-pocket costs and need for government subsidies. | **Minor concerns**  15 studies with minor to moderate concerns primarily due to lack of reflexivity. | **No/Very minor concerns** | **No/Very minor concerns** | **No/Very minor concerns** | **High confidence**  18 studies reflecting minor concerns regarding methodological limitations. | Chan et al. 2011; Chen et al. 2021; Clevenger et al. 2012; Cohen & Head 2013; Gray Brunton et al. 2014; Head & Cohen 2012; Hirth et al. 2018; Hodge et al. 2011; Hopfer & Clippard 2011; Lim & Lim 2019; Miller-Day et al. 2023; Pierre Joseph et al. 2014; Ross et al. 2010; Shetty et al. 2021; Siu 2013; Teitelman et al. 2018; Tu & Wang 2013; Wheldon et al. 2017; |
| 23 | Side effects: Young adults expressed concerned about the side effects of the HPV vaccine. | **Minor concerns**  10 studies with minor to moderate concerns primarily due to lack of reflexivity. | **No/Very minor concerns** | **No/Very minor concerns** | **No/Very minor concerns** | **High confidence**  12 studies reflecting minor concerns regarding methodological limitations. | Chan et al. 2011; Cohen & Head 2013; Garcia et al. 2023; Hirth et al. 2018; Hodge et al. 2011; Jaiswal et al. 2020; Joseph et al. 2014; Kim 2017; Mancuso & Polzer 2010; Petrova et al. 2015; Siu 2013; Stephens et al. 2016; |
| 24 | Understanding of vaccine effectiveness: Young adults questioned the effectiveness of the HPV vaccine given the limited strains it covers and lack of 100% protection. | **Minor concerns**  8 studies with minor to moderate concerns primarily due to lack of reflexivity. | **Minor concerns**  While there is data suggesting some young adults are not concerned about the effectiveness of the HPV vaccine, the descriptive finding highlights a key pattern observed in the data on the topic. | **Minor concerns**  9 studies contributed to this finding offering superficial data. However, the finding is simple and descriptive, and the supporting data provides sufficient information to understand the meaning/context. | **No/Very minor concerns** | **Moderate confidence**  9 studies reflecting minor concerns regarding methodological limitations, minor concerns regarding coherence, and minor concerns regarding adequacy. | Cohen & Head 2013; Gray Brunton et al. 2014; Hodge et al. 2011; Mancuso & Polzer 2010; Petrova et al. 2015; Ross et al. 2010; Thompson et al. 2017; Wheldon et al. 2017; Young et al. 2018; |
| 25 | Development of the vaccine: Concerns were raised about the vaccine's novelty, hastened development, and lack of long-term studies. | **Minor concerns**  9 studies with minor to moderate concerns primarily due to lack of reflexivity. | **No/Very minor concerns** | **No/Very minor concerns** | **No/Very minor concerns** | **High confidence**  11 studies reflecting minor concerns regarding methodological limitations. | Chan et al. 2011; Cohen & Head 2013; Gray Brunton et al. 2014; Hirth et al. 2018; Hodge 2014; Hopfer & Clippard 2011; Lim & Lim 2019; Petrova et al. 2015; Tu & Wang 2013; Wyndham-West 2016; Young et al. 2018; |
| 26 | Pharmaceutical companies and business motive: Young adults expressed skepticism towards pharmaceutical companies and their motives, suggesting that public panic and greed may influence vaccination campaigns. | **Minor concerns**  5 studies with minor to moderate concerns primarily due to lack of reflexivity. | **No/Very minor concerns** | **Minor concerns**  5 studies contributed to this finding offering a mix of rich and superficial data. However, the finding is simple and descriptive, and the supporting data provides sufficient information to understand the meaning/ context. | **No/Very minor concerns** | **Moderate confidence**  5 studies reflecting minor concerns regarding methodological limitations and Minor concerns regarding adequacy. | Chan et al. 2011; Gray Brunton et al. 2014; Mancuso & Polzer 2010; Petrova et al. 2015; Siu 2013; |
| **THEME 9: GENDERED PERCEPTIONS AND BIAS** | | | | | | | |
| 27 | Gendered perceptions of HPV risk: Many participants exhibit misconceptions about HPV being primarily a women's disease. | **Moderate concerns**  6 studies with minor to moderate concerns primarily due to lack of reflexivity and 1 study with moderate concerns due to lack of sampling strategy. | **No/Very minor concerns** | **Minor concerns**  7 studies contributed to this finding offering superficial data. However, the finding is simple and descriptive, and the supporting data provides sufficient information | **No/Very minor concerns** | **Moderate confidence**  7 studies reflecting moderate concerns regarding methodological limitations and minor concerns regarding adequacy. | Allen et al. 2009; Fontenot et al. 2016; Gerend et al. 2019; Hodge 2014; Jaiswal et al. 2020; Kim 2017; Mehta et al. 2013; |
| 28 | Gender biased promotion: The gendered marketing and promotion of HPV vaccines led to perceived unfairness in who gets access to vaccine and who is responsible for getting the vaccinated in the context of sexual health. | **Minor concerns**  8 studies with minor to moderate concerns primarily due to lack of reflexivity. | **No/Very minor concerns** | **No/Very minor concerns**  9 studies contributed to this finding offering rich and detailed data to understand and contextualize this phenomenon | **No/Very minor concerns** | **High confidence**  8 studies reflecting minor concerns regarding methodological limitations. | Apaydin et al. 2018; Fontenot et al. 2016; Gray Brunton et al. 2014; Hodge 2014; Jaiswal et al. 2020; Martin et al. 2011; Miller-Day et al. 2023; Wyndham-West 2016; |
| **THEME 10: GOVERNMENT AND POLICY** | | | | | | | |
| 29 | The government’s involvement and role (or lack thereof) in HPV vaccination promotion were influential in vaccine decision making. | **Minor concerns**  4 studies with minor to moderate concerns primarily due to lack of reflexivity. | **No/Very minor concerns** | **Minor concerns**  5 studies contributed to this finding offering thin and superficial data. However, the finding is simple and descriptive, and the supporting data provides sufficient information to understand the meaning/ context. | **No/Very minor concerns** | **Moderate confidence**  5 studies reflecting minor concerns regarding methodological limitations, and minor concerns regarding adequacy. | Basnyat & Lim 2018; Chen et al. 2021; Hodge et al. 2011; Petrova et al. 2015; Ross et al. 2010; |
