# Supplementary material for: Perspectives of HPV vaccine decision-making among young adults: A qualitative systematic review and evidence synthesis
Source: PLoS One. 2025 May 5;20(5):e0321448. doi: 10.1371/journal.pone.0321448 (PMC12052141; doi:10.1371/journal.pone.0321448)
Supplement: S5 Appendix — . (PDF) [file pone.0321448.s005.pdf]

Result #	Reference	Exclusion Reason
1	RefID: 1, AACR International Conference on the Science of Cancer Health Disparities 2011<br><br>Level: 1, State: Excluded	Wrong publication type (eg reviews, opinions, conference proceedings)
2	RefID: 2, SAHM 2011 Annual Meeting<br><br>Level: 1, State: Excluded	Wrong publication type (eg reviews, opinions, conference proceedings)
3	RefID: 3, Review of the 2010-2011 winter influenza season, northern hemisphere<br><br>Level: 1, State: Excluded	Not about HPV vaccination/vax attitudes (eg HPV infection/serology/prevalence; cervical cancer; HPV vax safety)
5	RefID: 5, Society for Adolescent Health and Medicine Annual Meeting: Impact of Trauma on Teens: Building the Safety Net 2012<br><br>Level: 1, State: Excluded	Wrong publication type (eg reviews, opinions, conference proceedings)
6	RefID: 6, Adult vaccination coverAge - Young adults 18-26yrs not included /outside age range - United States, 2010<br><br>Level: 1, State: Excluded	Not about HPV vaccination/vax attitudes (eg HPV infection/serology/prevalence; cervical cancer; HPV vax safety)
7	RefID: 7, Noninfluenza vaccination coverAge - Young adults 18-26yrs not included /outside age range among adults - United States, 2011<br><br>Level: 1, State: Excluded	Not about HPV vaccination/vax attitudes (eg HPV infection/serology/prevalence; cervical cancer; HPV vax safety)
8	RefID: 8, 2014 STD Prevention Conference<br><br>Level: 1, State: Excluded	Wrong publication type (eg reviews, opinions, conference proceedings)
9	RefID: 9, Uptake of HPV Vaccination Increasing Among U.S. Women Age - Young adults 18-26yrs not included /outside age ranged 18-26, but Disparities Persist<br><br>Level: 1, State: Excluded	Not about HPV vaccination/vax attitudes (eg HPV infection/serology/prevalence; cervical cancer; HPV vax safety)
10	RefID: 10, SAHM - 2015 Annual Meeting<br><br>Level: 1, State: Excluded	Wrong publication type (eg reviews, opinions, conference proceedings)
11	RefID: 11, Missed opportunities to immunize young adult males with the HPV vaccine to reduce oropharyngeal cancer<br><br>Level: 1, State: Excluded	Not about HPV vaccination/vax attitudes (eg HPV infection/serology/prevalence; cervical cancer; HPV vax safety)
12	RefID: 12, QuickStats: PercentAge - Young adults 18-26yrs not included /outside age range* of Adults Age - Young adults 18-26yrs not included /outside age ranged 18-26 Years Who Ever Received a Human Papillomavirus Vaccine,† by Race and Hispanic Origin§ and Sex - National Health Interview Survey, United States, 2019¶<br><br>Level: 1, State: Excluded	Study Design - Not qualitative (methods or analysis) OR Qualitative survey data not analyzed qualitatively (only numeric stats)

13	RefID: 13, Lessons learnt during the national introduction of human papillomavirus (HPV) vaccination programmes in 6 African countries: Stakeholders' perspectives<br>Abdullahi, L. H., Hussey, G. D., Wiysonge, C. S., Kagina, B. M.<br>Level: 2, State: Excluded	Wrong/no population (eg parents, providers, children, policy makers, does not include 18-26yrs olds; social media posts)
14	RefID: 14, Improving vaccination uptake among adolescents<br>Abdullahi, L. H., Kagina, B. M., Ndze, V. N., Hussey, G. D., Wiysonge, C. S.<br>Level: 1, State: Excluded	Wrong publication type (eg reviews, opinions, conference proceedings)
15	RefID: 15, Knowledge and attitude of Uyghur women in Xinjiang province of China related to the prevention and early detection of cervical cancer<br>Abudukadeer, A., Azam, S., Mutailipu, A. Z., Qun, L., Guilin, G., Mijiti, S.<br>Level: 1, State: Excluded	Not about HPV vaccination/vax attitudes (eg HPV infection/serology/prevalence; cervical cancer; HPV vax safety)
16	RefID: 16, Knowledge and Awareness of Parents Towards Human Papillomavirus (HPV) and HPV Vaccines, and Vaccine Acceptability in Northern Cyprus<br>Abuduxike, G., Asut, O., Cali, S., Vaizoglu, S.<br>Level: 1, State: Excluded	Wrong/no population (eg parents, providers, children, policy makers, does not include 18-26yrs olds; social media posts)
17	RefID: 17, Effect of nursing guideline about genital human papilloma virus infection on knowledge and attitude of female university students<br>Abuel-Zahab, N. H., Ahmed El-Sheikh, M., Abdel-Fattah, H., Metwally, N. S.<br>Level: 1, State: Excluded	Study Design - Not qualitative (methods or analysis) OR Qualitative survey data not analyzed qualitatively (only numeric stats)
19	RefID: 19, Unmasking in an observational vaccine safety study: Using type 2 diabetes mellitus as an example<br>Ackerson, B. K., Sy, L. S., Slezak, J., Chao, C. R., Hechter, R. C., Takhar, H. S., Jacobsen, S. J.<br>Level: 1, State: Excluded	Not about HPV vaccination/vax attitudes (eg HPV infection/serology/prevalence; cervical cancer; HPV vax safety)
20	RefID: 20, Delay in Diagnosis of Cervical Cancer in Afghanistan: A Pilot Cross-Sectional Survey<br>Acuti Martellucci, C., Delsoz, M., Qaderi, S., Madadi, S., Bhandari, D., Ozaki, A., Mousavi, S. H.<br>Level: 1, State: Excluded	Not about HPV vaccination/vax attitudes (eg HPV infection/serology/prevalence; cervical cancer; HPV vax safety)
21	RefID: 21, Beliefs and knowledge related to human papillomavirus (HPV) vaccine among African Americans and African immigrants young adults<br>Adegboyega, A., Obielodan, O., Wiggins, A. T., Dignan, M., Williams, L. B.<br>Level: 2, State: Excluded	Multiple Wrong/no population (eg parents, providers, children, policy makers, does not include 18-26yrs olds; social media posts)s - unable to extract data specific to young adults (18-26 yrs)
22	RefID: 22, Correlates of human papillomavirus (HPV) vaccination initiation and completion among 18-26 year olds in the United States<br>Adjei Boakye, E., Lew, D., Muthukrishnan, M., Tobo, B. B., Rohde, R. L., Varvares, M. A., Osazuwa-Peters, N.<br>Level: 1, State: Excluded	Study Design - Not qualitative (methods or analysis) OR Qualitative survey data not analyzed qualitatively (only numeric stats)
23	RefID: 23, Human papillomavirus vaccination uptake among Native Hawaiian and Pacific Islander adults in the United States<br>Adjei Boakye, E., Stierwalt, T., Grundy, S., Osazuwa-Peters, N., Lee, M.,	Study Design - Not qualitative (methods or analysis) OR Qualitative survey data not

	Elgee, M., Schootman, M.<br>Level: 1, State: Excluded	analyzed qualitatively (only numeric stats)
24	RefID: 24, Differences in HPV vaccine uptake by nativity status among American men Age - Young adults 18-26yrs not included /outside age ranged 18-32<br>Adjei Boakye, E., Zeng, W., Anderson, L., Chen, J., Tobo, B. B., Osazuwa-Peters, N.<br>Level: 1, State: Excluded	Study Design - Not qualitative (methods or analysis) OR Qualitative survey data not analyzed qualitatively (only numeric stats)
25	RefID: 25, Differences in human papillomavirus (HPV) vaccine uptake by nativity status among men Age - Young adults 18-26yrs not included /outside age ranged 18-34 years<br>Adjei Boakye, E., Zeng, W., Governor, S., NAge - Young adults 18-26yrs not included /outside age rangendra, S., Tobo, B. B., Simpson, M. C., Osazuwa-Peters, N.<br>Level: 1, State: Excluded	Not about HPV vaccination/vax attitudes (eg HPV infection/serology/prevalence; cervical cancer; HPV vax safety)
27	RefID: 27, Correlation between level of knowledge and attitude toward human papillomavirus vaccine in high school students<br>Adrianzén Peralta, A. L., Villalobos Popuche, V. A., Gonzales, H. L.<br>Level: 1, State: Excluded	Study Design - Not qualitative (methods or analysis) OR Qualitative survey data not analyzed qualitatively (only numeric stats)
28	RefID: 28, Intersectional nativity and racial/ethnic disparities in human papillomavirus vaccination initiation among U.S. women: a national Wrong/no population (eg parents, providers, children, policy makers, does not include 18-26yrs olds; social media posts)-based study<br>Agénor, M., Abboud, S., Delgadillo, J. G., Pérez, A. E., Peitzmeier, S. M., Borrero, S.<br>Level: 1, State: Excluded	Study Design - Not qualitative (methods or analysis) OR Qualitative survey data not analyzed qualitatively (only numeric stats)
29	RefID: 29, Impact of the Affordable Care Act on human papillomavirus vaccination initiation among lesbian, bisexual, and heterosexual U.S. women<br>Agénor, M., Murchison, G. R., Chen, J. T., Bowen, D. J., Rosenthal, M. B., Haneuse, S., Austin, S. B.<br>Level: 1, State: Excluded	Study Design - Not qualitative (methods or analysis) OR Qualitative survey data not analyzed qualitatively (only numeric stats)
30	RefID: 30, Sexual orientation identity disparities in human papillomavirus vaccination initiation and completion among young adult US women and men<br>Agénor, Madina, Peitzmeier, Sarah, Gordon, Allegra, Charlton, Brittany, Haneuse, Sebastien, Potter, Jennifer, Austin, S., Agénor, Madina, Peitzmeier, Sarah M., Gordon, Allegra R., Charlton, Brittany M., Austin, S. Bryn<br>Level: 1, State: Excluded	Not about HPV vaccination/vax attitudes (eg HPV infection/serology/prevalence; cervical cancer; HPV vax safety)
32	RefID: 32, Sexual orientation identity disparities in human papillomavirus vaccination initiation and completion in a national sample of young adult U.S. women and men<br>Agénor, M., Peitzmeier, S. M., Gordon, A. R., Charlton, B. M., Haneuse, S. J. P. A., Potter, J. E., Austin, S. B.<br>Level: 1, State: Excluded	Study Design - Not qualitative (methods or analysis) OR Qualitative survey data not analyzed qualitatively (only numeric stats)
33	RefID: 33, Racial/ethnic disparities in human papillomavirus vaccination initiation and completion among U.S. women in the post-Affordable Care Act era	Study Design - Not qualitative (methods or analysis) OR Qualitative survey data not

	Agénor, M., Pérez, A. E., Peitzmeier, S. M., Borrero, S.<br>Level: 1, State: Excluded	analyzed qualitatively (only numeric stats)
34	RefID: 34, Knowledge, attitudes and behaviors of sex workers about cervical cancer in a brothel from Adana, Turkey<br>Akcali, N., Nazlican, E., Akbaba, M., Okyay, R. A., Nayir, T.<br>Level: 1, State: Excluded	Not about HPV vaccination/vax attitudes (eg HPV infection/serology/prevalence; cervical cancer; HPV vax safety)
36	RefID: 36, Perception and opinion of medical students about Pap smear test: a qualitative study<br>Al-Naggar, R. A., Isa, Z. M.<br>Level: 1, State: Excluded	Not about HPV vaccination/vax attitudes (eg HPV infection/serology/prevalence; cervical cancer; HPV vax safety)
37	RefID: 37, Small area estimation of human papillomavirus vaccination coverAge - Young adults 18-26yrs not included /outside age range among school-Age - Young adults 18-26yrs not included /outside age range children in Alabama counties<br>Albright, D. L., Lee, H. Y., McDaniel, J. T., Kroner, D., Davis, J., Godfrey, K., Li, Q.<br>Level: 1, State: Excluded	Not about HPV vaccination/vax attitudes (eg HPV infection/serology/prevalence; cervical cancer; HPV vax safety)
38	RefID: 38, Noninitiation and Noncompletion of HPV Vaccine Among English- and Spanish-Speaking Parents of Adolescent Girls: A Qualitative Study<br>Albright, K., Barnard, J., O'Leary, S. T., Lockhart, S., Jimenez-Zambrano, A., Stokley, S., Dempsey, A., Kempe, A.<br>Level: 1, State: Excluded	Wrong/no population (eg parents, providers, children, policy makers, does not include 18-26yrs olds; social media posts)
39	RefID: 39, Acceptance of human papillomavirus (HPV) vaccination among young women in a country with a high prevalence of HPV infection<br>Alder, S., Perinetti, C., Mints, M., Belki, K., Sundström, K., Sandin, S., Weiderpass, E., Andersson, S.<br>Level: 1, State: Excluded	Study Design - Not qualitative (methods or analysis) OR Qualitative survey data not analyzed qualitatively (only numeric stats)
42	RefID: 42, Acceptance, Awareness, and Knowledge of Human Papillomavirus Vaccine in Eastern Province, Saudi Arabia<br>Almaghlouth, A. K., Bohamad, A. H., Alabbad, R. Y., Alghanim, J. H., Alqattan, D. J., Alkhalaf, R. A.<br>Level: 1, State: Excluded	Study Design - Not qualitative (methods or analysis) OR Qualitative survey data not analyzed qualitatively (only numeric stats)
43	RefID: 43, Awareness of human papillomavirus infection complications, cervical cancer, and vaccine among the saudi Wrong/no population (eg parents, providers, children, policy makers, does not include 18-26yrs olds; social media posts): A cross-sectional survey<br>Almehmadi, M. M., Salih, M. M., Al-Hazmi, A. S.<br>Level: 1, State: Excluded	Not about HPV vaccination/vax attitudes (eg HPV infection/serology/prevalence; cervical cancer; HPV vax safety)
44	RefID: 44, Evaluating associations between sources of information, knowledge of the human papillomavirus, and human papillomavirus vaccine uptake for adult women in California<br>Almeida, C. M., Tiro, J. A., Rodriguez, M. A., Diamant, A. L.<br>Level: 1, State: Excluded	Study Design - Not qualitative (methods or analysis) OR Qualitative survey data not analyzed qualitatively (only numeric stats)
45	RefID: 45, Human papillomavirus prevalence and type distribution among women attending routine gynecological examinations in Saudi Arabia<br>AlObaid, A., Al-Badawi, I. A., Al-Kadri, H., Gopala, K., Kandeil, W., Quint,	Age - Young adults 18-26yrs not included /outside age range

	W., Al-Aker, M., DeAntonio, R.<br>Level: 1, State: Excluded	
46	RefID: 46, Characteristics of HPV-unvaccinated undergraduate health students in Switzerland, a cross sectional study<br>Amadane, M., de Pree, C., Viviano, M., Vassilakos, P., Jeannot, E., Petignat, P.<br>Level: 1, State: Excluded	Not about HPV vaccination/vax attitudes (eg HPV infection/serology/prevalence; cervical cancer; HPV vax safety)
47	RefID: 47, HPV Vaccination Adherence in Working-Age - Young adults 18-26yrs not included /outside age range Men: A Systematic Review and Meta-Analysis<br>Amantea, C., Foschi, N., Gavi, F., Borrelli, I., Rossi, M. F., Spuntarelli, V., Russo, P., Gualano, M. R., Santoro, P. E., Moscato, U.<br>Level: 1, State: Excluded	Wrong publication type (eg reviews, opinions, conference proceedings)
48	RefID: 48, Safety of 4-valent human papillomavirus vaccine in males: a large observational post-marketing study<br>Amend, K. L., Turnbull, B., Zhou, L., Marks, M. A., Velicer, C., Saddier, P., Seeger, J. D.<br>Level: 1, State: Excluded	Study Design - Not qualitative (methods or analysis) OR Qualitative survey data not analyzed qualitatively (only numeric stats)
49	RefID: 49, Ontology-based dialogue systems for improved patient HPV vaccine knowledge and perception<br>Amith, M.<br>Level: 1, State: Excluded	Wrong publication type (eg reviews, opinions, conference proceedings)
50	RefID: 50, Examining Potential Usability and Health Beliefs Among Young Adults Using a Conversational Age - Young adults 18-26yrs not included /outside age rangent for HPV Vaccine Counseling<br>Amith, M., Lin, R., Cunningham, R., Wu, Q. L., Savas, L. S., Gong, Y., Boom, J. A., Tang, L., Tao, C.<br>Level: 1, State: Excluded	Not about HPV vaccination/vax attitudes (eg HPV infection/serology/prevalence; cervical cancer; HPV vax safety)
51	RefID: 51, Knowledge and intention to participate in cervical cancer screening after the human papillomavirus vaccine<br>Anhang Price, R., Koshiol, J., Kobrin, S., Tiro, J. A.<br>Level: 1, State: Excluded	Study Design - Not qualitative (methods or analysis) OR Qualitative survey data not analyzed qualitatively (only numeric stats)
52	RefID: 52, Use of human papillomavirus vaccines among young adult women in the United States: an analysis of the 2008 National Health Interview Survey<br>Anhang Price, R., Tiro, J. A., Saraiya, M., Meissner, H., Breen, N.<br>Level: 1, State: Excluded	Study Design - Not qualitative (methods or analysis) OR Qualitative survey data not analyzed qualitatively (only numeric stats)
55	RefID: 55, Prevalence of sexually transmitted diseases in female athletes in São Paulo, Brazil<br>Araujo, M. P., Kleine, H. T., Parmigiano, T. R., Gomes, N. T., Caparroz, G. P., Silva, I. D., Girão, M. J., Sartori, M. G.<br>Level: 1, State: Excluded	Not about HPV vaccination/vax attitudes (eg HPV infection/serology/prevalence; cervical cancer; HPV vax safety)
56	RefID: 56, Model-based estimates of long-term persistence of induced HPV antibodies: a flexible subject-specific approach<br>Aregay, M., Shkedy, Z., Molenberghs, G., David, M. P., Tibaldi, F.<br>Level: 1, State: Excluded	Not about HPV vaccination/vax attitudes (eg HPV infection/serology/prevalence; cervical cancer; HPV vax safety)
57	RefID: 57, Readiness for cancer cervix control in a north indian Wrong/no population (eg parents, providers, children, policy makers,	Not about HPV vaccination/vax attitudes (eg HPV

	does not include 18-26yrs olds; social media posts): Identifying the gaps<br>Arfin, M. I., Mahmood, S. E., Ahmad, A., Muzammil, K., Ahmad, M. T., Al-Musa, H. M.<br>Level: 1, State: Excluded	infection/serology/prevalence; cervical cancer; HPV vax safety)
58	RefID: 58, Perception and CoverAge - Young adults 18-26yrs not included /outside age range of Conventional Vaccination among University Students from Rouen (Normandy), France in 2021<br>Arias, A., Ladner, J., Tavolacci, M. P.<br>Level: 1, State: Excluded	Not about HPV vaccination/vax attitudes (eg HPV infection/serology/prevalence; cervical cancer; HPV vax safety)
59	RefID: 59, Cervical cancer screening and hpv vaccine acceptability among rural women in rajnandgaon, chhattisgarh: Barriers &obstacles<br>Armo, M., Sainik, S.<br>Level: 1, State: Excluded	Study Design - Not qualitative (methods or analysis) OR Qualitative survey data not analyzed qualitatively (only numeric stats)
60	RefID: 60, Prophylaxis of cervical cancer and related cervical disease: a review of the cost-effectiveness of vaccination against oncogenic HPV types<br>Armstrong, E. P.<br>Level: 1, State: Excluded	Not about HPV vaccination/vax attitudes (eg HPV infection/serology/prevalence; cervical cancer; HPV vax safety)
61	RefID: 61, Dental Hygiene Students' Perceptions Regarding the Importance of and Confidence with Using Brief Motivational Interviewing during HPV Patient Counseling<br>Arnett, M. C., Evans, M. D., Stull, C.<br>Level: 1, State: Excluded	Not about HPV vaccination/vax attitudes (eg HPV infection/serology/prevalence; cervical cancer; HPV vax safety)
62	RefID: 62, Brief Motivational Interviewing: Evaluation of a skills-based education program<br>Arnett, M. C., Evans, M. D., Stull, C. L.<br>Level: 1, State: Excluded	Wrong/no population (eg parents, providers, children, policy makers, does not include 18-26yrs olds; social media posts)
63	RefID: 63, Acceptability and uptake of HPV vaccine in Argentina before its inclusion in the immunization program: a Wrong/no population (eg parents, providers, children, policy makers, does not include 18-26yrs olds; social media posts)-based survey<br>Arrossi, S., Maceira, V., Paolino, M., Sankaranarayanan, R.<br>Level: 1, State: Excluded	Study Design - Not qualitative (methods or analysis) OR Qualitative survey data not analyzed qualitatively (only numeric stats)
64	RefID: 64, Arguments in favor and against the new HPV school entry implementation in Puerto Rico: Contentanalysis of online media coverAge - Young adults 18-26yrs not included /outside age range<br>Arroyo-Morales, G. O., Rivera-Figueroa, V., Soto-Abreu, R., Rivera-Encarnacion, M. E., Diaz-Miranda, O. L., Medina-Laabels, D. T., Ortiz-Martinez, A. P., Suarez-Perez, E. L., Fernandez, M. E., Hull, P. C., Colon-Lopez, V.<br>Level: 1, State: Excluded	Wrong/no population (eg parents, providers, children, policy makers, does not include 18-26yrs olds; social media posts)
65	RefID: 65, Risk factors associated with the incidence of cervical cancer in Dr. H. Abdul Moeloek hospital Bandar Lampung in 2019<br>Aryawati, W., Arianingsih, N. M., Aryastuti, N.<br>Level: 1, State: Excluded	Not about HPV vaccination/vax attitudes (eg HPV infection/serology/prevalence; cervical cancer; HPV vax safety)
66	RefID: 66, Sexual Mixing Patterns and Anal Human Papillomavirus Among Young Gay, Bisexual, and Other Men Who Have Sex With Men and Transgender Women in 2 Cities in the United States, 2012-2014	Study Design - Not qualitative (methods or analysis) OR Qualitative survey data not

	Assaf, R. D., Javanbakht, M., Meites, E., Gratzer, B., Steinau, M., Crosby, R. A., Markowitz, L. E., Unger, E. R., Gorbach, P. M.<br>Level: 1, State: Excluded	analyzed qualitatively (only numeric stats)
67	RefID: 67, The attitudes of parents working in the health sector about their children to be vaccinated for human papilloma virus<br>Ateş, S., Taşdelen-Öğülmen, D., Işik, M. E.<br>Level: 1, State: Excluded	Wrong/no population (eg parents, providers, children, policy makers, does not include 18-26yrs olds; social media posts)
68	RefID: 68, The effect of intrauterine devices on acquisition and clearance of human papillomavirus<br>Averbach, S. H., Ma, Y., Smith-McCune, K., Shiboski, S., Moscicki, A. B.<br>Level: 1, State: Excluded	Not about HPV vaccination/vax attitudes (eg HPV infection/serology/prevalence; cervical cancer; HPV vax safety)
69	RefID: 69, Inpatient Immunization With HPV Vaccine: A Qualitative Study With Postpartum Women<br>Avni-Singer, L., Oliveira, C. R., Torres, A., Shapiro, E. D., Niccolai, L. M., Sheth, S. S.<br>Level: 2, State: Excluded	Multiple Wrong/no population (eg parents, providers, children, policy makers, does not include 18-26yrs olds; social media posts)s - unable to extract data specific to young adults (18-26 yrs)
70	RefID: 70, Texting and Mobile Phone App Interventions for Improving Adherence to Preventive Behavior in Adolescents: A Systematic Review<br>Badawy, S. M., Kuhns, L. M.<br>Level: 1, State: Excluded	Wrong publication type (eg reviews, opinions, conference proceedings)
71	RefID: 71, Perceptions of cervical cancer prevention among a group of ethnic minority women in Denmark-A qualitative study<br>Badre-Esfahani, S., Petersen, L. K., Tatari, C. R., Blaakær, J., Andersen, B., Seibæk, L.<br>Level: 2, State: Excluded	Wrong/no population (eg parents, providers, children, policy makers, does not include 18-26yrs olds; social media posts)
72	RefID: 72, Implementation of HPV vaccination guidelines in a diverse Wrong/no population (eg parents, providers, children, policy makers, does not include 18-26yrs olds; social media posts) in Los Angeles: Results from an environmental scan of local HPV resources and needs<br>Baezconde-Garbanati, L., Lienemann, B. A., Robles, M., Johnson, E., Sanchez, K., Singhal, R., Steinberg, J., Jaque, J. M., Pentz, M. A., Gruber, S.<br>Level: 1, State: Excluded	Wrong/no population (eg parents, providers, children, policy makers, does not include 18-26yrs olds; social media posts)
73	RefID: 73, Comparing one dose of HPV vaccine in girls Age - Young adults 18-26yrs not included /outside age ranged 9-14 years in Tanzania (DoRIS) with one dose of HPV vaccine in historical cohorts: an immunobridging analysis of a randomised controlled trial<br>Baisley, K., Kemp, T. J., Kreimer, A. R., Basu, P., Changalucha, J., Hildesheim, A., Porras, C., Whitworth, H., Herrero, R., Lacey, C. J., et al.<br>Level: 1, State: Excluded	Not about HPV vaccination/vax attitudes (eg HPV infection/serology/prevalence; cervical cancer; HPV vax safety)
77	RefID: 77, Awareness Regarding Cervical Cancer among Reproductive Age - Young adults 18-26yrs not included /outside age range Women Residing in Nuwakot District<br>Bajracharya, S. L., Chalise, P.<br>Level: 1, State: Excluded	Wrong/no population (eg parents, providers, children, policy makers, does not include 18-26yrs olds; social media posts)
78	RefID: 78, Knowledge, attitude, and practice of cervical cancer screening among Greek students: a short report<br>Bakogianni, G. D., Goutsou, S. C., Liti, M. V., Rizopoulou, S. I.,	Not about HPV vaccination/vax attitudes (eg HPV

	Nikolakopoulos, K. M., Nikolakopoulou, N. M.<br>Level: 1, State: Excluded	infection/serology/prevalence; cervical cancer; HPV vax safety)
80	RefID: 80, HPV vaccine acceptance among female Greek students<br>Bakogianni, G. D., Nikolakopoulos, K., Nikolakopoulou, N. M., Bakogianni, Giannoula D., Nikolakopoulos, Konstantinos M., Nikolakopoulou, Nikoleta M.<br>Level: 1, State: Excluded	Study Design - Not qualitative (methods or analysis) OR Qualitative survey data not analyzed qualitatively (only numeric stats)
82	RefID: 82, HPV Types Distribution in General Female Wrong/no population (eg parents, providers, children, policy makers, does not include 18-26yrs olds; social media posts) and in Women Diagnosed with Cervical Cancer Across Western Kazakhstan<br>Balmagambetova, S. K., Tinelli, A., Urazayev, O. N., Sakieva, K. Z., Koyshybaev, A. K., Zholmukhamedova, D. A., Urazayeva, S. T.<br>Level: 1, State: Excluded	Wrong/no population (eg parents, providers, children, policy makers, does not include 18-26yrs olds; social media posts)
83	RefID: 83, Investigating Bangladeshi Rural Women's Awareness and Knowledge of Cervical Cancer and Attitude Towards HPV Vaccination: a Community-Based Cross-Sectional Analysis<br>Banik, R., Naher, S., Rahman, M., Gozal, D.<br>Level: 1, State: Excluded	Study Design - Not qualitative (methods or analysis) OR Qualitative survey data not analyzed qualitatively (only numeric stats)
84	RefID: 84, Awareness of cervical cancer and willingness to be vaccinated against human papillomavirus in Mozambican adolescent girls<br>Bardají, A., Mindu, C., Augusto, O. J., Casellas, A., Cambaco, O., Simbine, E., Matsinhe, G., Macete, E., Menéndez, C., Sevene, E., Munguambe, K.<br>Level: 1, State: Excluded	Study Design - Not qualitative (methods or analysis) OR Qualitative survey data not analyzed qualitatively (only numeric stats)
85	RefID: 85, Risk of spontaneous abortion and other pregnancy outcomes in 15-25 year old women exposed to human papillomavirus-16/18 AS04-adjuvanted vaccine in the United Kingdom<br>Baril, L., Rosillon, D., Willame, C., Angelo, M. G., Zima, J., Bosch, J. H. van den, Staa, T. van, Boggon, R., Bunge, E. M., Hernandez-Diaz, S., Chambers, C. D.<br>Level: 1, State: Excluded	Not about HPV vaccination/vax attitudes (eg HPV infection/serology/prevalence; cervical cancer; HPV vax safety)
87	RefID: 87, Focus group discussions in community-based participatory research to inform the development of a human papillomavirus (HPV) educational intervention for Latinas in San Diego<br>Barnack-Tavlaris, J. L., Garcini, L., Sanchez, O., Hernandez, I., Navarro, A. M.<br>Level: 2, State: Excluded	Wrong/no population (eg parents, providers, children, policy makers, does not include 18-26yrs olds; social media posts)
88	RefID: 88, Human Papillomavirus Vaccination Awareness and Acceptability Among U.S.-Born and U.S. Foreign-Born Women Living in California<br>Barnack-Tavlaris, J. L., Garcini, L. M., Macera, C. A., Brodine, S., Klonoff, E. A.<br>Level: 1, State: Excluded	Study Design - Not qualitative (methods or analysis) OR Qualitative survey data not analyzed qualitatively (only numeric stats)
89	RefID: 89, Interventions to increase uptake of the human papillomavirus vaccine in unvaccinated college students: A systematic literature review<br>Barnard, M., Cole, A. C., Ward, L., Gravlee, E., Cole, M. L., Compretta, C.<br>Level: 1, State: Excluded	Wrong publication type (eg reviews, opinions, conference proceedings)

90	RefID: 90, Cervical cancer prevention services at college health centers: Historically Black Colleges and Universities (HBCUs) compared to Predominantly White Institutions (PWIs)<br>Barnett, K. B., McRee, A. L., Reiter, P. L., Paskett, E. D., Katz, M. L.<br>Level: 1, State: Excluded	Not about HPV vaccination/vax attitudes (eg HPV infection/serology/prevalence; cervical cancer; HPV vax safety)
91	RefID: 91, New prophylactics human papilloma virus (HPV) vaccines against cervical cancer<br>Barra, F., Leone Roberti Maggiore, U., Bogani, G., Ditto, A., Signorelli, M., Martinelli, F., Chiappa, V., Lorusso, D., Raspagliesi, F., Ferrero, S.<br>Level: 1, State: Excluded	Wrong publication type (eg reviews, opinions, conference proceedings)
94	RefID: 94, Knowledge, Perceptions, and Uptake of the Human Papillomavirus Vaccine in a Sample of US High School Adolescents<br>Barrett, C., Scoular, S., Borgelt, L. M.<br>Level: 1, State: Excluded	Study Design - Not qualitative (methods or analysis) OR Qualitative survey data not analyzed qualitatively (only numeric stats)
95	RefID: 95, A content analysis of HPV related posts on instagram<br>Basch Ch EdD, M. P. H., MacLean, Sa Ba<br>Level: 1, State: Excluded	Wrong/no population (eg parents, providers, children, policy makers, does not include 18-26yrs olds; social media posts)
96	RefID: 96, Awareness of women about cervical smear, human papilloma virus and human papilloma virus vaccine<br>Başer, E., Onat, T., Kırmızı, D. A., Çaltekin, M. D., Kara, M., Yalvaç, E. S.<br>Level: 1, State: Excluded	Wrong/no population (eg parents, providers, children, policy makers, does not include 18-26yrs olds; social media posts)
98	RefID: 98, Acceptability of human papillomavirus vaccine among the urban, affluent and educated parents of young girls residing in Kolkata, Eastern India<br>Basu, P., Mittal, S.<br>Level: 1, State: Excluded	Wrong/no population (eg parents, providers, children, policy makers, does not include 18-26yrs olds; social media posts)
99	RefID: 99, A single dose of quadrivalent human papillomavirus (HPV) vaccine is immunogenic and reduces HPV detection rates in young women in Mongolia, six years after vaccination<br>Batmunkh, T., Dalmau, M. T., Munkhsaikhan, M. E., Khorolsuren, T., Namjil, N., Surenjav, U., Toh, Z. Q., Licciardi, P. V., Russell, F. M., Garland, S. M., Mulholland, K., von Mollendorf, C.<br>Level: 1, State: Excluded	Study Design - Not qualitative (methods or analysis) OR Qualitative survey data not analyzed qualitatively (only numeric stats)
100	RefID: 100, Which social representations about cancer related to HPV infection and the associated vaccine from the teenAge - Young adults 18-26yrs not included /outside age rangers?<br>Bauquier, C., Préau, M.<br>Level: 1, State: Excluded	Wrong/no population (eg parents, providers, children, policy makers, does not include 18-26yrs olds; social media posts)
101	RefID: 101, How French adolescents use imAge - Young adults 18-26yrs not included /outside age ranges to represent HPV vaccination (during school-based workshops) adolescents and HPV vaccination<br>Bauquier, C., Préau, M.<br>Level: 1, State: Excluded	Wrong/no population (eg parents, providers, children, policy makers, does not include 18-26yrs olds; social media posts)
102	RefID: 102, Low Human Papillomavirus Literacy Among Asian-American Women in California: an Analysis of the California Health Interview Survey	Study Design - Not qualitative (methods or analysis) OR Qualitative survey data not

	Becerra, M. B., Avina, R. M., Mshigeni, S., Becerra, B. J.<br>Level: 1, State: Excluded	analyzed qualitatively (only numeric stats)
103	RefID: 103, Human papillomavirus vaccine uptake and barriers: association with perceived risk, actual risk and race/ethnicity among female students at a New York State university, 2010<br>Bednarczyk, R. A., Birkhead, G. S., Morse, D. L., Doleyres, H., McNutt, L. A.<br>Level: 2, State: Excluded	Study Design - Not qualitative (methods or analysis) OR Qualitative survey data not analyzed qualitatively (only numeric stats)
104	RefID: 104, "I'm not ashamed to talk on it!": African-American women's decisions about cervical cancer prevention and control in South Carolina<br>Bellinger, J. D., Millegan, W., Abdalla, A. E.<br>Level: 2, State: Excluded	Multiple Wrong/no population (eg parents, providers, children, policy makers, does not include 18-26yrs olds; social media posts)s - unable to extract data specific to young adults (18-26 yrs)
105	RefID: 105, Awareness and attitude towards HPV and its vaccines among market women in Bodija Market, Ibadan<br>Bello, O. O., Oluwasola, T. A. O., Odukogbe, A. A.<br>Level: 1, State: Excluded	Study Design - Not qualitative (methods or analysis) OR Qualitative survey data not analyzed qualitatively (only numeric stats)
106	RefID: 106, HPV Literacy and Associated Factors Among Hmong American Immigrants: Implications for Reducing Cervical Cancer Disparity<br>Beltran, R., Simms, T., Lee, H. Y., Kwon, M.<br>Level: 1, State: Excluded	Study Design - Not qualitative (methods or analysis) OR Qualitative survey data not analyzed qualitatively (only numeric stats)
107	RefID: 107, Human Papillomavirus Vaccine Uptake After a Tailored, Online Educational Intervention for Female University Students: a Randomized Controlled Trial<br>Bennett, A. T., Patel, D. A., Carlos, R. C., Zochowski, M. K., Pennewell, S. M., Chi, A. M., Dalton, V. K.<br>Level: 1, State: Excluded	Study Design - Not qualitative (methods or analysis) OR Qualitative survey data not analyzed qualitatively (only numeric stats)
108	RefID: 108, Quantitative and qualitative assessment of an all-inclusive postpartum human papillomavirus vaccination program<br>Berenson, Abbey B., Hirth, Jacqueline M., Kuo, Yong-Fang, Rupp, Richard E.<br>Level: 1, State: Excluded	Wrong/no population (eg parents, providers, children, policy makers, does not include 18-26yrs olds; social media posts)
110	RefID: 110, Assessing the need for and acceptability of a free-of-charge postpartum HPV vaccination program<br>Berenson, A. B., Male, E., Lee, T. G., Barrett, A., Sarpong, K. O., Rupp, R. E., Rahman, M.<br>Level: 1, State: Excluded	Study Design - Not qualitative (methods or analysis) OR Qualitative survey data not analyzed qualitatively (only numeric stats)
111	RefID: 111, Comparison of different human papillomavirus (HPV) vaccine types and dose schedules for prevention of HPV-related disease in females and males<br>Bergman, H., Buckley, B. S., Villanueva, G., Petkovic, J., Garritty, C., Lutje, V., Riveros-Balta, A. X., Low, N., Henschke, N.<br>Level: 1, State: Excluded	Wrong publication type (eg reviews, opinions, conference proceedings)
113	RefID: 113, Characteristics associated with initiation of the human papillomavirus vaccine among a national sample of male and female young adults	Study Design - Not qualitative (methods or analysis) OR Qualitative survey data not

	Bernat, Debra H., Gerend, Mary A., Chevallier, Kenya, Zimmerman, Marc A., Bauermeister, Jose A.<br>Level: 1, State: Excluded	analyzed qualitatively (only numeric stats)
114	RefID: 114, An instrument assessing attitudes and beliefs toward human papillomavirus vaccination<br>Bertulfo, T. F., Heo, S., Troyan, P., Randolph, J., An, M.<br>Level: 1, State: Excluded	Study Design - Not qualitative (methods or analysis) OR Qualitative survey data not analyzed qualitatively (only numeric stats)
115	RefID: 115, Voice-based conversational Age - Young adults 18-26yrs not included /outside age rangents for the prevention and manAge - Young adults 18-26yrs not included /outside age rangement of chronic and mental health conditions: Systematic literature review<br>Bérubé, C., Schachner, T., Keller, R., Fleisch, E., Wangenheim, F. V., Barata, F., Kowatsch, T.<br>Level: 1, State: Excluded	Wrong publication type (eg reviews, opinions, conference proceedings)
116	RefID: 116, Human papillomavirus vaccination uptake and its associated factors among adolescent school girls in Ambo town, Oromia region, Ethiopia, 2020<br>Beyen, M. W., Bulto, G. A., Chaka, E. E., Debelo, B. T., Roga, E. Y., Wakgari, N., Danusa, K. T., Fekene, D. B.<br>Level: 2, State: Excluded	Multiple Wrong/no population (eg parents, providers, children, policy makers, does not include 18-26yrs olds; social media posts)s - unable to extract data specific to young adults (18-26 yrs)
117	RefID: 117, Prevalence of oral HPV infection in unvaccinated young adults in Brazil<br>Bidinotto, A. B., Kops, N. L., Bessel, M., Maranhão, A. G. K., Moreno, F., Pereira, G. F. M., Villa, L. L., Wendland, E. M.<br>Level: 1, State: Excluded	Not about HPV vaccination/vax attitudes (eg HPV infection/serology/prevalence; cervical cancer; HPV vax safety)
118	RefID: 118, Effective or ineffective: attribute framing and the human papillomavirus (HPV) vaccine<br>Bigman, C. A., Cappella, J. N., Hornik, R. C.<br>Level: 1, State: Excluded	Not about HPV vaccination/vax attitudes (eg HPV infection/serology/prevalence; cervical cancer; HPV vax safety)
119	RefID: 119, Did the recommendation of the French public health council modify the antipapillomavirus vaccines' prescription level?<br>Birebent, J., Dupouy, J., Lempégnat, J., Bourrel, R., Bismuth, M., Oustric, S.<br>Level: 1, State: Excluded	Wrong/no population (eg parents, providers, children, policy makers, does not include 18-26yrs olds; social media posts)
120	RefID: 120, Willingness to accept human papillomavirus vaccination in Jimma town, Ethiopia<br>Biyazin, T., Yetwale, A., Fenta, B.<br>Level: 1, State: Excluded	Study Design - Not qualitative (methods or analysis) OR Qualitative survey data not analyzed qualitatively (only numeric stats)
121	RefID: 121, Knowledge and attitude about human papillomavirus vaccine among female high school students at Jimma town, Ethiopia<br>Biyazin, T., Yilma, A., Yetwale, A., Fenta, B., Dagnaw, Y.<br>Level: 1, State: Excluded	Study Design - Not qualitative (methods or analysis) OR Qualitative survey data not analyzed qualitatively (only numeric stats)
122	RefID: 122, Between youth and adulthood: focus group discussions with 30-year-old women about cervical cancer and its prevention in urban Sweden<br>Blomberg, Karin, Widmark, Catarina, Ternestedt, Britt-Marie, Tornberg,	Not about HPV vaccination/vax attitudes (eg HPV infection/serology/prevalence; cervical cancer; HPV vax safety)

	Sven, Tishelman, Carol<br>Level: 1, State: Excluded	
124	RefID: 124, Researching and Respecting the Intricacies of Isolated Communities<br>Blumling, Amy A., Thomas, Tami L., Stephens, Dionne P.<br>Level: 1, State: Excluded	Wrong/no population (eg parents, providers, children, policy makers, does not include 18-26yrs olds; social media posts)
127	RefID: 127, Factors associated with HPV vaccination initiation and completion among 18-26 year olds in the United States<br>Boakye, E. A., Tobo, B. B., Lew, D., Muthukrishnan, M., Pham, V. T., Rohde, R., Burroughs, T., Varvares, M. A., Osazuwa-Peters, N.<br>Level: 1, State: Excluded	Study Design - Not qualitative (methods or analysis) OR Qualitative survey data not analyzed qualitatively (only numeric stats)
128	RefID: 128, Human Papillomavirus Vaccine Messaging on TikTok: Social Media Content Analysis<br>Boatman, D. D., Eason, S., Conn, M. E., Kennedy-Rea, S. K.<br>Level: 1, State: Excluded	Wrong/no population (eg parents, providers, children, policy makers, does not include 18-26yrs olds; social media posts)
129	RefID: 129, Sexual behavior, use of contraceptive methods and risk factors for HPV infections of students living in central Italy: implications for vaccination strategies<br>Boccalini, S., Tiscione, E., Bechini, A., Levi, M., Mencacci, M., Petrucci, F., Bani Assad, G., Santini, M. G., Bonanni, P.<br>Level: 1, State: Excluded	Study Design - Not qualitative (methods or analysis) OR Qualitative survey data not analyzed qualitatively (only numeric stats)
130	RefID: 130, WHIM syndrome: Presumptive diagnosis based on myelokathexis on bone marrow smear<br>Bock, I., Dugué, F., Loppinet, E., Bellanné-Chantelot, C., Bénet, B.<br>Level: 1, State: Excluded	Not about HPV vaccination/vax attitudes (eg HPV infection/serology/prevalence; cervical cancer; HPV vax safety)
131	RefID: 131, Racial and Ethnic Group Knowledge, Perceptions and Behaviors about Human Papillomavirus, Human Papillomavirus Vaccination, and Cervical Cancer among Adolescent Females<br>Bond, S. M., Cartmell, K. B., Lopez, C. M., Ford, M. E., Brandt, H. M., Gore, E. I., Zapka, J. G., Alberg, A. J.<br>Level: 2, State: Excluded	Multiple Wrong/no population (eg parents, providers, children, policy makers, does not include 18-26yrs olds; social media posts)s - unable to extract data specific to young adults (18-26 yrs)
133	RefID: 133, HPV vaccine narratives on Twitter during the COVID-19 pandemic: a social network, thematic, and sentiment analysis<br>Boucher, J. C., Kim, S. Y., Jessiman-Perreault, G., Edwards, J., Smith, H., Frenette, N., Badami, A., Scott, L. A.<br>Level: 1, State: Excluded	Wrong/no population (eg parents, providers, children, policy makers, does not include 18-26yrs olds; social media posts)
134	RefID: 134, Prevalence and distribution of hpv genotypes in immunosuppressed patients in lorraine region<br>Boudes, M., Venard, V., Routiot, T., Buzzi, M., Maillot, F.<br>Level: 1, State: Excluded	Not about HPV vaccination/vax attitudes (eg HPV infection/serology/prevalence; cervical cancer; HPV vax safety)
135	RefID: 135, Barriers and facilitators to HPV vaccination among rural Alabama adolescents and their caregivers<br>Boyd, E. D., Phillips, J. M., Schoenberger, Y. M., Simpson, T.<br>Level: 2, State: Excluded	Multiple Wrong/no population (eg parents, providers, children, policy makers, does not include 18-26yrs olds; social media posts)s - unable to extract data specific to young adults (18-26 yrs)
137	RefID: 137, How often people google for vaccination: Qualitative and quantitative insights from a systematic search of the web-based activities using Google Trends	Wrong/no population (eg parents, providers, children, policy makers,

	Bragazzi, N. L., Barberis, I., Rosselli, R., Gianfredi, V., Nucci, D., Moretti, M., Salvatori, T., Martucci, G., Martini, M.<br>Level: 1, State: Excluded	does not include 18-26yrs olds; social media posts)
138	RefID: 138, Evaluating a Technology-Mediated HPV Vaccination Awareness Intervention: A Controlled, Quasi-Experimental, Mixed Methods Study<br>Brandt, H. M., Sundstrom, B., Monroe, C. M., Turner-McGrievy, G., Larsen, C., Stansbury, M., Magradey, K., Gibson, A., West, D. S.<br>Level: 2, State: Excluded	Multiple Wrong/no population (eg parents, providers, children, policy makers, does not include 18-26yrs olds; social media posts)s - unable to extract data specific to young adults (18-26 yrs)
139	RefID: 139, The Development of a Culturally Relevant, Theoretically Driven HPV Prevention Intervention for Urban Adolescent Females and Their Parents/Guardians<br>Brawner, Bridgette M., Baker, Jillian L., Voytek, Chelsea D., Leader, Amy, Cashman, Rebecca R., Silverman, Randee, Peter, Nadja, Buchner, Bradley J., Barnes, Christopher A., Jemmott, Loretta S., Frank, Ian<br>Level: 1, State: Excluded	Study Design - Not qualitative (methods or analysis) OR Qualitative survey data not analyzed qualitatively (only numeric stats)
140	RefID: 140, Pharmacist authority to provide HPV vaccine: Novel partners in cervical cancer prevention<br>Brewer, N. T., Chung, J. K., Baker, H. M., Rothholz, M. C., Smith, J. S.<br>Level: 1, State: Excluded	Wrong/no population (eg parents, providers, children, policy makers, does not include 18-26yrs olds; social media posts)
141	RefID: 141, Longitudinal predictors of human papillomavirus vaccine initiation among adolescent girls in a high-risk geographic area<br>Brewer, N. T., Gottlieb, S. L., Reiter, P. L., McRee, A. L., Liddon, N., Markowitz, L., Smith, J. S.<br>Level: 1, State: Excluded	Wrong/no population (eg parents, providers, children, policy makers, does not include 18-26yrs olds; social media posts)
143	RefID: 143, Primary outcomes from implementing a behavioral counseling intervention program in four federally qualified health centers<br>Brookmeyer, K., Copen, C., Lentine, D., Hogben, M.<br>Level: 1, State: Excluded	Not about HPV vaccination/vax attitudes (eg HPV infection/serology/prevalence; cervical cancer; HPV vax safety)
144	RefID: 144, Early effect of the HPV vaccination programme on cervical abnormalities in Victoria, Australia: an ecological study<br>Brotherton, J. M., Fridman, M., May, C. L., Chappell, G., Saville, A. M., Gertig, D. M.<br>Level: 1, State: Excluded	Not about HPV vaccination/vax attitudes (eg HPV infection/serology/prevalence; cervical cancer; HPV vax safety)
145	RefID: 145, Human papillomavirus (HPV) vaccination coverAge - Young adults 18-26yrs not included /outside age range in young Australian women is higher than previously estimated: independent estimates from a nationally representative mobile phone survey<br>Brotherton, J. M., Liu, B., Donovan, B., Kaldor, J. M., Saville, M.<br>Level: 1, State: Excluded	Study Design - Not qualitative (methods or analysis) OR Qualitative survey data not analyzed qualitatively (only numeric stats)
148	RefID: 148, Estimating human papillomavirus vaccination coverAge - Young adults 18-26yrs not included /outside age range among young women in Victoria and reasons for non-vaccination<br>Brotherton, J. M. L., Piers, L. S., Vaughan, L.<br>Level: 2, State: Excluded	Study Design - Not qualitative (methods or analysis) OR Qualitative survey data not analyzed qualitatively (only numeric stats)
149	RefID: 149, Peruvian Female Sex Workers' Ethical Perspectives on Their Participation in an HPV Vaccine Clinical Trial	Not about HPV vaccination/vax attitudes (eg HPV

	Brown, Brandon, Davtyan, Mariam, Fisher, Celia B.<br>Level: 2, State: Excluded	infection/serology/prevalence; cervical cancer; HPV vax safety)
150	RefID: 150, Human papillomavirus (HPV) vaccination: What can be found on the Web? Qualitative analysis of the Doctissimo.fr forum data<br>Bruel, S., Peyrard-Chevrier, K., Ginzarly, M., Frappé, P., Savall, A.<br>Level: 1, State: Excluded	Wrong/no population (eg parents, providers, children, policy makers, does not include 18-26yrs olds; social media posts)
151	RefID: 151, Beliefs about HPV vaccination and awareness of vaccination status: Gender differences among Northern Italy adolescents<br>Brunelli, L., Bravo, G., Romanese, F., Righini, M., Lesa, L., De Odorico, A., Bastiani, E., Pascut, S., Miceli, S., Brusaferro, S.<br>Level: 1, State: Excluded	Study Design - Not qualitative (methods or analysis) OR Qualitative survey data not analyzed qualitatively (only numeric stats)
152	RefID: 152, Human papillomavirus vaccine initiation in Asian Indians and Asian subWrong/no population (eg parents, providers, children, policy makers, does not include 18-26yrs olds; social media posts)s: a case for examining disaggregated data in public health research<br>Budhwani, H., De, P.<br>Level: 1, State: Excluded	Study Design - Not qualitative (methods or analysis) OR Qualitative survey data not analyzed qualitatively (only numeric stats)
153	RefID: 153, Developing a Clinic-Based, Vaccine-Promoting Intervention for African American Youth in Rural Alabama: Protocol for a Pilot Cluster-Randomized Controlled Implementation Science Trial<br>Budhwani, H., Sharma, V., Long, D., Simpson, T.<br>Level: 2, State: Excluded	studytype
154	RefID: 154, Determinants of HPV knowledge and HPV vaccine uptake among a U. S. military Wrong/no population (eg parents, providers, children, policy makers, does not include 18-26yrs olds; social media posts)<br>Buechel, J.<br>Level: 1, State: Excluded	Study Design - Not qualitative (methods or analysis) OR Qualitative survey data not analyzed qualitatively (only numeric stats)
156	RefID: 156, Optimizing intersectoral collaboration between health and education: The Health Bridges study<br>Burgess, T., Braunack-Mayer, A., Tooher, R., Collins, J., O'Keefe, M., Skinner, R., Watson, M., Ashmeade, H., Proeve, C., Marshall, H.<br>Level: 1, State: Excluded	Not about HPV vaccination/vax attitudes (eg HPV infection/serology/prevalence; cervical cancer; HPV vax safety)
157	RefID: 157, Protecting our Khmer daughters: ghosts of the past, uncertain futures, and the human papillomavirus vaccine<br>Burke, N. J., Do, H. H., Talbot, J., Sos, C., Ros, S., Taylor, V. M.<br>Level: 1, State: Excluded	Wrong/no population (eg parents, providers, children, policy makers, does not include 18-26yrs olds; social media posts)
158	RefID: 158, Constructions of young women's health and wellbeing in neoliberal times: A case study of the HPV vaccination program in Australia<br>Burns, K., Davies, C.<br>Level: 2, State: Excluded	studytype
160	RefID: 160, Influences to HPV completion via a school-based immunisation program<br>Burns, Sharyn, Selvey, Linda, Roux, Felicity<br>Level: 1, State: Excluded	Wrong/no population (eg parents, providers, children, policy makers, does not include 18-26yrs olds; social media posts)
161	RefID: 161, Seroprevalence and awareness of human papillomavirus infection and cervical cancer screening results among reproductive-Age - Young adults 18-26yrs not included /outside age ranged Georgian	Not about HPV vaccination/vax attitudes (eg HPV

	women<br>Butsashvili, Maia, Abzianidze, Tinatin, Kajaia, Maia, Agladze, Dodo, Kldiashvili, Ekaterine, Bednarczyk, Robert, McNutt, Louise-Anne, Kamkamidze, George<br>Level: 1, State: Excluded	infection/serology/prevalence; cervical cancer; HPV vax safety)
163	RefID: 163, Knowledge, beliefs, and attitudes related to human papillomavirus infection and vaccination, pap tests, and cervical intraepithelial neoplasia among adolescent girls and young women<br>Bynum, S. A., Wright, M. S., Brandt, H. M., Burgis, J. T., Bacon, J. L.<br>Level: 2, State: Excluded	Multiple Wrong/no population (eg parents, providers, children, policy makers, does not include 18-26yrs olds; social media posts)s - unable to extract data specific to young adults (18-26 yrs)
164	RefID: 164, Knowledge on human papillomavirus (HPV), HPV screening and HPV vaccine among sexual health clinic patients in Martinique, French West Indies<br>Cabras, O., Sylvanise, L., Marquise, A., Cabié, A., Cuzin, L.<br>Level: 1, State: Excluded	Study Design - Not qualitative (methods or analysis) OR Qualitative survey data not analyzed qualitatively (only numeric stats)
165	RefID: 165, Understanding the determinants of vaccine hesitancy and vaccine confidence among adolescents: a systematic review<br>Cadeddu, C., Castagna, C., Sapienza, M., Lanza, T. E., Messina, R., Chiavarini, M., Ricciardi, W., de Waure, C.<br>Level: 1, State: Excluded	Wrong publication type (eg reviews, opinions, conference proceedings)
166	RefID: 166, Exploring the Role of Ethnic Identity on the Attitudes Towards HPV Vaccine Advertising Among Puerto Ricans: A Qualitative Analysis<br>Calo, William, Fernández, Maria, Fernández-Espada, Natalie, Colón-López, Vivian<br>Level: 2, State: Excluded	Not about HPV vaccination/vax attitudes (eg HPV infection/serology/prevalence; cervical cancer; HPV vax safety)
167	RefID: 167, The Healthy, Immunized Communities Study: A pilot intervention to increase parents' intentions to get vaccines for their middle school children<br>Calo, W. A., Hivner, E. A., Hoke, A. M., Bufalini, C. M., Lehman, E. B., Kraschnewski, J. L.<br>Level: 1, State: Excluded	Study Design - Not qualitative (methods or analysis) OR Qualitative survey data not analyzed qualitatively (only numeric stats)
168	RefID: 168, Sexually transmitted infections in youth with controlled and uncontrolled HIV<br>Camacho-Gonzalez, A. F., Chernoff, M. C., Williams, P. L., Chahroudi, A., Oleske, J. M., Chakraborty, R., Traite, S., Purswani, M. U., Abzug, M. J.<br>Level: 1, State: Excluded	Not about HPV vaccination/vax attitudes (eg HPV infection/serology/prevalence; cervical cancer; HPV vax safety)
169	RefID: 169, Sexually Transmitted Infections in Youth With Controlled and Uncontrolled Human Immunodeficiency Virus Infection<br>Camacho-Gonzalez, A. F., Chernoff, M. C., Williams, P. L., Chahroudi, A., Oleske, J. M., Traite, S., Chakraborty, R., Purswani, M. U., Abzug, M. J.<br>Level: 1, State: Excluded	Not about HPV vaccination/vax attitudes (eg HPV infection/serology/prevalence; cervical cancer; HPV vax safety)
170	RefID: 170, Incidence of genital warts in adolescents and young adults in an integrated health care delivery system in the United States before human papillomavirus vaccine recommendations<br>Camenga, D. R., Dunne, E. F., Desai, M. M., Gee, J., Markowitz, L. E., Desiliva, A., Klein, N. P.<br>Level: 1, State: Excluded	Not about HPV vaccination/vax attitudes (eg HPV infection/serology/prevalence; cervical cancer; HPV vax safety)

171	RefID: 171, Participatory action for Human Papillomivirus (HPV) vaccine acceptability among young-adult men: Case study<br>Campbell, Lilieth<br>Level: 1, State: Excluded	Study Design - Not qualitative (methods or analysis) OR Qualitative survey data not analyzed qualitatively (only numeric stats)
172	RefID: 172, Factors related to vaccine uptake by young adult women in the catch-up phase of the National HPV Vaccination Program in Australia: Results from an observational study<br>Canfell, K., Egger, S., Velentzis, L. S., Brown, J. D., O'Connell, D. L., Banks, E., Sitas, F.<br>Level: 1, State: Excluded	Study Design - Not qualitative (methods or analysis) OR Qualitative survey data not analyzed qualitatively (only numeric stats)
173	RefID: 173, Determinants of the HPV vaccination among girls attending Spanish primary care settings<br>Cano, B. C., Martin-Perez, M., Montero-Corominas, D., Martin-Merino, E.<br>Level: 1, State: Excluded	Age - Young adults 18-26yrs not included /outside age range
174	RefID: 174, Barriers and Facilitators to Improving Virginia's HPV Vaccination Rate: A Stakeholder Analysis With Implications for Pediatric Nurses<br>Carhart, Miev Y., Schminkey, Donna L., Mitchell, Emma M., Keim-Malpass, Jessica<br>Level: 1, State: Excluded	Wrong/no population (eg parents, providers, children, policy makers, does not include 18-26yrs olds; social media posts)
175	RefID: 175, HPV Vaccination Communication MessAge - Young adults 18-26yrs not included /outside age ranges, Messengers, and Messaging Strategies<br>Cartmell, K. B., Mzik, C. R., Sundstrom, B. L., Luque, J. S., White, A., Young-Pierce, J.<br>Level: 1, State: Excluded	Wrong/no population (eg parents, providers, children, policy makers, does not include 18-26yrs olds; social media posts)
176	RefID: 176, Print news coverAge - Young adults 18-26yrs not included /outside age range of school-based human papillomavirus vaccine mandates<br>Casciotti, D. M., Smith, K. C., Andon, L., Vernick, J., Tsui, A., Klassen, A. C.<br>Level: 1, State: Excluded	Wrong/no population (eg parents, providers, children, policy makers, does not include 18-26yrs olds; social media posts)
178	RefID: 178, Epidemiology and cost of treatment of genital warts in Spain<br>Castellsagué, X., Cohet, C., Puig-Tintoré, L. M., Acebes, L. O., Salinas, J., San Martin, M., Breitscheidel, L., Rémy, V.<br>Level: 1, State: Excluded	Not about HPV vaccination/vax attitudes (eg HPV infection/serology/prevalence; cervical cancer; HPV vax safety)
179	RefID: 179, Using the Theory of Planned Behavior to predict HPV vaccination intentions of college men<br>Catalano, H. P., Knowlden, A. P., Birch, D. A., Leeper, J. D., Paschal, A. M., Usdan, S. L.<br>Level: 1, State: Excluded	Study Design - Not qualitative (methods or analysis) OR Qualitative survey data not analyzed qualitatively (only numeric stats)
180	RefID: 180, Racial differences in HPV knowledge, HPV vaccine acceptability, and related beliefs among rural, southern women<br>Cates, J. R., Brewer, N. T., Fazekas, K. I., Mitchell, C. E., Smith, J. S.<br>Level: 1, State: Excluded	Study Design - Not qualitative (methods or analysis) OR Qualitative survey data not analyzed qualitatively (only numeric stats)

181	RefID: 181, Partnering with middle school students to design text messAge - Young adults 18-26yrs not included /outside age ranges about HPV vaccination<br>Cates, J. R., Ortiz, R. R., North, S., Martin, A., Smith, R., Coyne-Beasley, T.<br>Level: 1, State: Excluded	Wrong/no population (eg parents, providers, children, policy makers, does not include 18-26yrs olds; social media posts)
182	RefID: 182, Inuit women's attitudes and experiences towards cervical cancer and prevention strategies in Nunavik, Quebec<br>Cerigo, H., Macdonald, M. E., Franco, E. L., Brassard, P.<br>Level: 2, State: Excluded	Study Design - Not qualitative (methods or analysis) OR Qualitative survey data not analyzed qualitatively (only numeric stats)
183	RefID: 183, Anogenital human papillomavirus virus DNA and sustained response to the quadrivalent HPV vaccine in women living with HIV-1<br>Cespedes, M. S., Kang, M., Kojic, E. M., Umbleja, T., Godfrey, C., Webster-Cyriaque, J. Y., Masih, R., Firnhaber, C., Grinsztejn, B., Saah, A., Cu-Uvin, S., Aberg, J. A.<br>Level: 1, State: Excluded	Not about HPV vaccination/vax attitudes (eg HPV infection/serology/prevalence; cervical cancer; HPV vax safety)
184	RefID: 184, Evaluation of fotonovela to increase human papillomavirus vaccine knowledge, attitudes, and intentions in a low-income Hispanic community<br>Chan, A., Brown, B., Sepulveda, E., Teran-Clayton, L.<br>Level: 1, State: Excluded	Study Design - Not qualitative (methods or analysis) OR Qualitative survey data not analyzed qualitatively (only numeric stats)
187	RefID: 187, Epidemiologic risk profile of infection with different groups of human papillomaviruses<br>Chan, P. K., Ho, W. C., Wong, M. C., Chang, A. R., Chor, J. S., Yu, M. Y.<br>Level: 1, State: Excluded	Not about HPV vaccination/vax attitudes (eg HPV infection/serology/prevalence; cervical cancer; HPV vax safety)
189	RefID: 189, Would Chinese Men Who Have Sex With Men Take Up Human Papillomavirus (HPV) Screening as an Alternative Prevention Strategy to HPV Vaccination?<br>Chan, P. S. F., Fang, Y., Chidgey, A., Fong, F., Ip, M., Wang, Z.<br>Level: 1, State: Excluded	Study Design - Not qualitative (methods or analysis) OR Qualitative survey data not analyzed qualitatively (only numeric stats)
190	RefID: 190, [CONDYDAV: A multicentre observational study of patients presenting external genital warts in France]<br>Chanal, J., Fouéré, S., Yassir-Oria, F., Spenatto, N., Bouscarat, F., Picot, E., Martinet, P., Vernay-Vaisse, C., Pelletier, F., Courtieu, C., Baclet, V., Bernier, C., Aymar-Moulene, D., Dupuis-Fourdan, F., Passeron, A., Bara-Passot, C., Pinault, A. L., Misery, L., Janier, M., Dupin, N.<br>Level: 1, State: Excluded	Not about HPV vaccination/vax attitudes (eg HPV infection/serology/prevalence; cervical cancer; HPV vax safety)
191	RefID: 191, A double-blind, randomized controlled trial to compare the effectiveness and safety of purified protein derivative of tuberculin antigen with Mycobacterium w vaccine in the treatment of multiple viral warts<br>Chandra, S., Sil, A., Datta, A., Pal, S., Das, N. K.<br>Level: 1, State: Excluded	Not about HPV vaccination/vax attitudes (eg HPV infection/serology/prevalence; cervical cancer; HPV vax safety)
192	RefID: 192, Does electronic consent improve the logistics and uptake of HPV vaccination in adolescent girls? A mixed-methods theory informed evaluation of a pilot intervention<br>Chantler, T., Pringle, E., Bell, S., Cooper, R., Edmundson, E., Nielsen, H., Roberts, S., Edelstein, M., Mounier-Jack, S.<br>Level: 1, State: Excluded	Wrong/no population (eg parents, providers, children, policy makers, does not include 18-26yrs olds; social media posts)

193	RefID: 193, Surveillance of autoimmune conditions following routine use of quadrivalent human papillomavirus vaccine<br>Chao, C., Klein, N. P., Velicer, C. M., Sy, L. S., Slezak, J. M., Takhar, H., Ackerson, B., Cheetham, T. C., Hansen, J., Deosaransingh, K., Emery, M., Liaw, K. L., Jacobsen, S. J.<br>Level: 1, State: Excluded	Not about HPV vaccination/vax attitudes (eg HPV infection/serology/prevalence; cervical cancer; HPV vax safety)
194	RefID: 194, Correlates for human papillomavirus vaccination of adolescent girls and young women in a manAge - Young adults 18-26yrs not included /outside age ranged care organization<br>Chao, C., Velicer, C., Slezak, J. M., Jacobsen, S. J.<br>Level: 1, State: Excluded	Study Design - Not qualitative (methods or analysis) OR Qualitative survey data not analyzed qualitatively (only numeric stats)
195	RefID: 195, Multistakeholder knowledge levels and perspectives of human papillomavirus and its vaccination: An exploratory qualitative study<br>Chau, J. P. C., Lo, S. H. S., Lee, V. W. Y., Lui, G. C. Y., Butt, L., Chan, K. M., Kwok, C. Y., Lau, A. Y. L.<br>Level: 1, State: Excluded	Wrong/no population (eg parents, providers, children, policy makers, does not include 18-26yrs olds; social media posts)
199	RefID: 199, Analysis of the interactive meaning of journalistic imAge - Young adults 18-26yrs not included /outside age ranges of the human papillomavirus vaccine and the perceptions of female undergraduate students<br>Chen, J., Tao, R., Guo, Q.<br>Level: 1, State: Excluded	Wrong/no population (eg parents, providers, children, policy makers, does not include 18-26yrs olds; social media posts)
200	RefID: 200, HPV Vaccination Among Young Adults in the US<br>Chen, M. M., Mott, N., Clark, S. J., Harper, D. M., Shuman, A. G., Prince, M. E. P., Dossett, L. A.<br>Level: 1, State: Excluded	Not about HPV vaccination/vax attitudes (eg HPV infection/serology/prevalence; cervical cancer; HPV vax safety)
201	RefID: 201, Thrombocytopenic Purpura and vaccination: Where do we stand?<br>Chenaf, C., Zenut, M., Eschalier, A.<br>Level: 1, State: Excluded	Not about HPV vaccination/vax attitudes (eg HPV infection/serology/prevalence; cervical cancer; HPV vax safety)
202	RefID: 202, Multiple types of human papillomavirus infection and anal precancerous lesions in HIV-infected men in Taiwan: a cross-sectional study<br>Cheng, S. H., Liao, K. S., Wang, C. C., Cheng, C. Y., Chu, F. Y.<br>Level: 1, State: Excluded	Not about HPV vaccination/vax attitudes (eg HPV infection/serology/prevalence; cervical cancer; HPV vax safety)
203	RefID: 203, Perception and knowledge of human papillomavirus (HPV) vaccine for cervical cancer prevention among fully vaccinated female university students in the era of HPV vaccination: a cross-sectional study<br>Chew, KahTeik, Nirmala, Kampan, Mohamad Nasir, Shafiee<br>Level: 1, State: Excluded	Study Design - Not qualitative (methods or analysis) OR Qualitative survey data not analyzed qualitatively (only numeric stats)
205	RefID: 205, Human papillomavirus prevalence and behavioral risk factors among HIV-infected and HIV-uninfected men who have sex with men in Taiwan<br>Chia-Chun, Lin, Ming-Chang, Hsieh, Hung-Chang, Hung, Shih-Ming, Tsao, Shiuan-Chih, Chen, Hao-Jan, Yang, Yuan-Ti, Lee, Lin, Chia-Chun, Hsieh, Ming-Chang, Hung, Hung-Chang, Tsao, Shih-Ming, Chen, Shiuan-Chih, Yang, Hao-Jan, Lee, Yuan-Ti<br>Level: 1, State: Excluded	Study Design - Not qualitative (methods or analysis) OR Qualitative survey data not analyzed qualitatively (only numeric stats)

206	RefID: 206, Knowledge and awareness of human papillomavirus and intention with regard to human papillomavirus vaccine uptake by female tertiary students in the Eastern Cape province<br>Chikandiwa, A., Van Wyk, B. E.<br>Level: 1, State: Excluded	Study Design - Not qualitative (methods or analysis) OR Qualitative survey data not analyzed qualitatively (only numeric stats)
207	RefID: 207, Girls' knowledge about HPV vaccine and cervical cancer<br>Chivu, C., Clarke, A., Hundt, G.<br>Level: 2, State: Excluded	studytype
208	RefID: 208, Preliminary efficacy of a tailored narrative intervention to increase human papillomavirus vaccination intention among a multi-ethnic sample of female students<br>Cho, D., Wang, C., Pierce, J., Dawkins-Moultin, L., Lu, Q.<br>Level: 1, State: Excluded	Study Design - Not qualitative (methods or analysis) OR Qualitative survey data not analyzed qualitatively (only numeric stats)
209	RefID: 209, Acceptability and uptake of female adolescent HPV vaccination in Hong Kong: a survey of mothers and adolescents<br>Choi, H. C., Leung, G. M., Woo, P. P., Jit, M., Wu, J. T.<br>Level: 1, State: Excluded	Study Design - Not qualitative (methods or analysis) OR Qualitative survey data not analyzed qualitatively (only numeric stats)
211	RefID: 211, Rapid fall in quadrivalent vaccine targeted human papillomavirus genotypes in heterosexual men following the Australian female HPV vaccination programme: An observational study from 2004 to 2015<br>Chow, E., Machalek, D., Tabrizi, S., Danielewski, J., Fehler, G., Bradshaw, C., Garland, S., Chen, M., Fairley, C., Walker, S.<br>Level: 1, State: Excluded	Not about HPV vaccination/vax attitudes (eg HPV infection/serology/prevalence; cervical cancer; HPV vax safety)
212	RefID: 212, Quadrivalent vaccine-targeted human papillomavirus genotypes in heterosexual men after the Australian female human papillomavirus vaccination programme: a retrospective observational study<br>Chow, E. P. F., Machalek, D. A., Tabrizi, S. N., Danielewski, J. A., Fehler, G., Bradshaw, C. S., Garland, S. M., Chen, M. Y., Fairley, C. K.<br>Level: 1, State: Excluded	Study Design - Not qualitative (methods or analysis) OR Qualitative survey data not analyzed qualitatively (only numeric stats)
213	RefID: 213, Prevalence of human papillomavirus in young men who have sex with men after the implementation of gender-neutral HPV vaccination: a repeated cross-sectional study<br>Chow, E. P. F., Tabrizi, S. N., Fairley, C. K., Wigan, R., Machalek, D. A., Garland, S. M., Cornall, A. M., Atchison, S., Hocking, J. S., Bradshaw, C. S., Balgovind, P., Murray, G. L., Chen, M. Y.<br>Level: 1, State: Excluded	Study Design - Not qualitative (methods or analysis) OR Qualitative survey data not analyzed qualitatively (only numeric stats)
214	RefID: 214, Knowledge, attitude, and practices regarding human papillomavirus and its' vaccination among the young medical professionals and students of Bangladesh<br>Chowdhury, S., Ara, R., Roy, S., Tanvir, S. M. S., Eva, F. N., Neela, T. M., Moonmoon, A. A., Sifat, S., Zamila, M., Hawlader, M. D. H.<br>Level: 1, State: Excluded	Wrong/no population (eg parents, providers, children, policy makers, does not include 18-26yrs olds; social media posts)
215	RefID: 215, Health care provider's experience and perspective of cervical cancer screening in Singapore: A qualitative study<br>Chua, B. W. B., Neo, P., Ma, V. Y., Lim, L. M., Ng, J. S. Y., Wee, H. L.<br>Level: 1, State: Excluded	Not about HPV vaccination/vax attitudes (eg HPV infection/serology/prevalence; cervical cancer; HPV vax safety)

216	RefID: 216, Monkeypox outbreak in Genoa, Italy: Clinical, laboratory, histopathologic features, manAge - Young adults 18-26yrs not included /outside age rangement, and outcome of the infected patients<br>Ciccarese, G., Di Biagio, A., Bruzzone, B., Guadagno, A., Taramasso, L., Oddenino, G., Brucci, G., Labate, L., De Pace, V., Mastrolonardo, M., Broccolo, F., Robello, G., Drago, F., Bassetti, M., Parodi, A.<br>Level: 1, State: Excluded	Not about HPV vaccination/vax attitudes (eg HPV infection/serology/prevalence; cervical cancer; HPV vax safety)
218	RefID: 218, HPV knowledge, screening barriers and facilitators, and sources of health information among women living with HIV: perspectives from the DC community during the COVID-19 pandemic<br>Ciceron, A. C., Jeon, M. J., Monroe, A. K., Clausen, M. E., Magnus, M., Le, D.<br>Level: 1, State: Excluded	Not about HPV vaccination/vax attitudes (eg HPV infection/serology/prevalence; cervical cancer; HPV vax safety)
221	RefID: 221, The determination of the knowledge level and behavior of Turkish women from various occupations about human papillomavirus, cervical cancer, and pap smear test<br>Cimke, Vildan, Borekci, Gulay, Cimke, Vildan Savas<br>Level: 1, State: Excluded	Not about HPV vaccination/vax attitudes (eg HPV infection/serology/prevalence; cervical cancer; HPV vax safety)
223	RefID: 223, Knowledge and Behavior of University Students toward Human Papillomavirus and Vaccination<br>Cinar, İlgun, Ozkan, Sevgi, Aslan, Gulbahar, Alatas, Erkan<br>Level: 1, State: Excluded	Study Design - Not qualitative (methods or analysis) OR Qualitative survey data not analyzed qualitatively (only numeric stats)
225	RefID: 225, Level of behavior and knowledge concerning human papillomavirus among university students of a nursing college<br>Cirilo, C. A., Barbosa, A. S., Zambrano, E.<br>Level: 1, State: Excluded	Study Design - Not qualitative (methods or analysis) OR Qualitative survey data not analyzed qualitatively (only numeric stats)
227	RefID: 227, Parent Perception of Provider Interactions Influences HPV Vaccination Status of Adolescent Females<br>Clark, S. J., Cowan, A. E., Filipp, S. L., Fisher, A. M., Stokley, S.<br>Level: 1, State: Excluded	Wrong/no population (eg parents, providers, children, policy makers, does not include 18-26yrs olds; social media posts)
229	RefID: 229, Decline in hospitalization for genital warts in the Veneto region after an HPV vaccination program: an observational study<br>Cocchio, S., Baldovin, T., Bertoncello, C., Buja, A., Furlan, P., Saia, M., Baldo, V.<br>Level: 1, State: Excluded	Not about HPV vaccination/vax attitudes (eg HPV infection/serology/prevalence; cervical cancer; HPV vax safety)
230	RefID: 230, HPV vaccination uptake among foreign-born Blacks in the US: insights from the National Health Interview Survey 2013-2017<br>Cofie, L. E., Tailor, H. D., Lee, M. H., Xu, L.<br>Level: 1, State: Excluded	Study Design - Not qualitative (methods or analysis) OR Qualitative survey data not analyzed qualitatively (only numeric stats)
233	RefID: 233, Factors Associated with HPV Vaccine Use Among Hispanic College Students<br>Cohen, Timmerie F., Legg, Jeffrey S.<br>Level: 1, State: Excluded	Study Design - Not qualitative (methods or analysis) OR Qualitative survey data not analyzed qualitatively (only numeric stats)
234	RefID: 234, Understanding the influential factors to human papillomavirus vaccination among youth in Harris County, Texas: A	Wrong/no population (eg parents, providers, children, policy makers,

	qualitative study<br>Coleman, A., Mallory, M., Montealegre, J., Smith, P., Buzi, R., Scheurer, M., Daheri, M., Jibaja-Weiss, M., Anderson, M.<br>Level: 1, State: Excluded	does not include 18-26yrs olds; social media posts)
235	RefID: 235, ¡Habla de VPH! An Educational Activity for College Students in Puerto Rico<br>Colón-López, V., Ayala-Marín, A., Vélez-Alamo, C., Soto-Salgado, M., Medina-Cortés, L., Acevedo-Fontanez, A. I., Ortiz, A. P., Fernández-Espada, N., Sánchez-Aracil, M., Salgado-Cruz, O.<br>Level: 1, State: Excluded	Study Design - Not qualitative (methods or analysis) OR Qualitative survey data not analyzed qualitatively (only numeric stats)
238	RefID: 238, Communication about sex and HPV among Puerto Rican mothers and daughters<br>Colón-López, V., Fernández-Espada, N., Vélez, C., Gonzalez, V. J., Diaz-Toro, E. C., Calo, W. A., Savas, L. S., Pattatucci, A., Fernández, M. E.<br>Level: 2, State: Excluded	Multiple Wrong/no population (eg parents, providers, children, policy makers, does not include 18-26yrs olds; social media posts)s - unable to extract data specific to young adults (18-26 yrs)
239	RefID: 239, Prevalence and Correlates of Penile HPV Infection in a Clinic-Based Sample of Hispanic Males<br>Colón-López, V., Ortiz, A. P., Del Toro-Mejías, L., Clatts, M., Durán-Guzmán, G., Pérez, N., DaCosta, M., Palefsky, J.<br>Level: 1, State: Excluded	Not about HPV vaccination/vax attitudes (eg HPV infection/serology/prevalence; cervical cancer; HPV vax safety)
241	RefID: 241, Content analysis of digital media coverAge - Young adults 18-26yrs not included /outside age range of the human papillomavirus vaccine school-entry requirement policy in Puerto Rico<br>Colón-López, V., Rivera-Figueroa, V., Arroyo-Morales, G. O., Medina-Laabes, D. T., Soto-Abreu, R., Rivera-Encarnación, M., Díaz-Miranda, O. L., Ortiz, A. P., Wells, K. B., Vázquez-Otero, C., Hull, P. C.<br>Level: 1, State: Excluded	Study Design - Not qualitative (methods or analysis) OR Qualitative survey data not analyzed qualitatively (only numeric stats)
242	RefID: 242, Implementation of the human papillomavirus school-entry requirement in Puerto Rico: barriers and facilitators using the consolidated framework for implementation research<br>Colón-López, V., Soto-Abreu, R., Medina-Laabes, D. T., Díaz-Miranda, O. L., Ortiz, A. P., Suárez, E. L., Hull, P. C.<br>Level: 1, State: Excluded	Wrong/no population (eg parents, providers, children, policy makers, does not include 18-26yrs olds; social media posts)
243	RefID: 243, Characteristics associated with HPV-related external genital lesions among young adults in Brazil<br>Comerlato, J., Bessel, M., Kops, N., Horvath, J., Fernandes, B. V., Jacobsen, A., Wendland, E.<br>Level: 1, State: Excluded	Not about HPV vaccination/vax attitudes (eg HPV infection/serology/prevalence; cervical cancer; HPV vax safety)
244	RefID: 244, Sex differences in the prevalence and determinants of HPV-related external genital lesions in young adults: a national cross-sectional survey in Brazil<br>Comerlato, J., Kops, N. L., Bessel, M., Horvath, J. D., Fernandes, B. V., Villa, L. L., de Souza, F. M. A., Pereira, G. F. M., Wendland, E. M.<br>Level: 1, State: Excluded	Not about HPV vaccination/vax attitudes (eg HPV infection/serology/prevalence; cervical cancer; HPV vax safety)
246	RefID: 246, Epidemiology of Humanpapilloma virus infection among women in Fujian, China<br>Conglian, Wu, Xianjin, Zhu, Yanli, Kang, Yinping, Cao, Pingxia, Lu, Wenjuan, Zhou, Hong, Zhou, Yang, Zhang, Yanfang, Song, Wu, Conglian, Zhu, Xianjin, Kang, Yanli, Cao, Yinping, Lu, Pingxia, Zhou, Wenjuan,	Not about HPV vaccination/vax attitudes (eg HPV infection/serology/prevalence; cervical cancer; HPV vax safety)

	Zhou, Hong, Zhang, Yang, Song, Yanfang<br>Level: 1, State: Excluded	
247	RefID: 247, School-based vaccination: A systematic review of process evaluations<br>Cooper Robbins, S. C., Ward, K., Skinner, S. R.<br>Level: 1, State: Excluded	Wrong publication type (eg reviews, opinions, conference proceedings)
248	RefID: 248, Exploring knowledge, attitudes and acceptance of HPV vaccination in adolescent males<br>Cooper, S., Reddington, K., Hillman, R., Burns, K., Davies, C., Skinner, R.<br>Level: 1, State: Excluded	
249	RefID: 249, Perception about barriers and facilitators of the school-based HPV vaccine program of Manizales, Colombia: A qualitative study in school-enrolled girls and their parents<br>Cordoba-Sanchez, V., Tovar-Aguirre, O. L., Franco, S., Arias Ortiz, N. E., Louie, K., Sanchez, G. I., Garces-Palacio, I. C.<br>Level: 2, State: Excluded	Wrong/no population (eg parents, providers, children, policy makers, does not include 18-26yrs olds; social media posts)
250	RefID: 250, Sexual and reproductive health in Spanish University Students. A comparison between medical and law students<br>Coronado, P. J., Delgado-Miguel, C., Rey-Cañas, A., Herráiz, M. A.<br>Level: 1, State: Excluded	Study Design - Not qualitative (methods or analysis) OR Qualitative survey data not analyzed qualitatively (only numeric stats)
251	RefID: 251, Effects of Educational Interventions on Human Papillomavirus Vaccine Acceptability: A Randomized Controlled Trial<br>Cory, L., Cha, B., Ellenberg, S., Bogner, H. R., Hwang, W. T., Smith, J. S., Haggerty, A., Morgan, M., Burger, R., Chu, C., Ko, E. M.<br>Level: 1, State: Excluded	Study Design - Not qualitative (methods or analysis) OR Qualitative survey data not analyzed qualitatively (only numeric stats)
252	RefID: 252, HPV and HPV vaccination: knowledge and consciousness of young women<br>Coşar, E., Gencer, M., Hacivelioğlu, S. O., Güngör, A. C., Uysal, A.<br>Level: 2, State: Excluded	Study Design - Not qualitative (methods or analysis) OR Qualitative survey data not analyzed qualitatively (only numeric stats)
254	RefID: 254, Wrong/no population (eg parents, providers, children, policy makers, does not include 18-26yrs olds; social media posts)-based frequency assessment of HPV-induced lesions in patients with borderline Pap tests in the Emilia-Romagna Region: the PATER study<br>Costa, S., Venturoli, S., Mennini, F. S., Marcellusi, A., Pesaresi, M., Leo, E., Falasca, A., Marra, E., Cricca, M., Santini, D., Zerbini, M., Pelusi, G.<br>Level: 1, State: Excluded	Not about HPV vaccination/vax attitudes (eg HPV infection/serology/prevalence; cervical cancer; HPV vax safety)
255	RefID: 255, The role of the social media in Public Health: A new opportunity to meet the communication needs of the general Wrong/no population (eg parents, providers, children, policy makers, does not include 18-26yrs olds; social media posts)<br>Costantino, C.<br>Level: 1, State: Excluded	Wrong publication type (eg reviews, opinions, conference proceedings)
256	RefID: 256, Improving HPV Vaccination Rates in a Racially and Ethnically Diverse Pediatric Wrong/no population (eg parents, providers, children, policy makers, does not include 18-26yrs olds; social media posts)<br>Cox, J. E., Bogart, L. M., Elliott, M. N., Starmer, A. J., Meleedy-Rey, P., Goggin, K., Banerjee, T., Samuels, R. C., Hahn, P. D., Epee-Bounya, A.,	Wrong/no population (eg parents, providers, children, policy makers, does not include 18-26yrs olds; social media posts)

	Allende-Richter, S., Fu, C. M., Schuster, M. A.<br>Level: 1, State: Excluded	
257	RefID: 257, "Who will take the blame?": understanding the reasons why Romanian mothers decline HPV vaccination for their daughters<br>Craciun, C., Baban, A.<br>Level: 1, State: Excluded	Wrong/no population (eg parents, providers, children, policy makers, does not include 18-26yrs olds; social media posts)
258	RefID: 258, Possible adverse effects of the quadrivalent human papillomavirus vaccine in the Region of Southern Denmark: a retrospective, descriptive cohort study<br>Cramon, C., Poulsen, C. L., Hartling, U. B., Holden, I. K., Johansen, I. S.<br>Level: 1, State: Excluded	Study Design - Not qualitative (methods or analysis) OR Qualitative survey data not analyzed qualitatively (only numeric stats)
259	RefID: 259, Recommending the HPV Vaccine: New Skills for a New Recommendation in Oral Health<br>Crespo, E., Oliphant, J. A.<br>Level: 1, State: Excluded	Wrong/no population (eg parents, providers, children, policy makers, does not include 18-26yrs olds; social media posts)
260	RefID: 260, Gardasil for guys: Correlates of intent to be vaccinated<br>Crosby, R. A., DiClemente, R. J., Salazar, L. F., Nash, R., Younge, S.<br>Level: 1, State: Excluded	Study Design - Not qualitative (methods or analysis) OR Qualitative survey data not analyzed qualitatively (only numeric stats)
261	RefID: 261, Human papillomavirus vaccine intention among college men: what's oral sex got to do with it?<br>Crosby, R. A., DiClemente, R. J., Salazar, L. F., Nash, R., Younge, S., Head, S.<br>Level: 2, State: Excluded	Study Design - Not qualitative (methods or analysis) OR Qualitative survey data not analyzed qualitatively (only numeric stats)
262	RefID: 262, Cervical cancer screening and HPV vaccine acceptability among rural and urban women in Kilimanjaro Region, Tanzania<br>Cunningham, M. S., Skrastins, E., Fitzpatrick, R., Jindal, P., Oneko, O., Yeates, K., Booth, C. M., Carpenter, J., Aronson, K. J.<br>Level: 1, State: Excluded	Study Design - Not qualitative (methods or analysis) OR Qualitative survey data not analyzed qualitatively (only numeric stats)
263	RefID: 263, Healthcare providers' practice protocols, strategies, and needed tools to address parental HPV vaccine hesitancy: An exploratory study<br>Cunningham-Erves, J., Hull, P. C., Wilkins, C. H., Edwards, K. M., Davis, M., Jones, J., Graham, J., Adekunle, A., Dempsey, A. F.<br>Level: 1, State: Excluded	Wrong/no population (eg parents, providers, children, policy makers, does not include 18-26yrs olds; social media posts)
264	RefID: 264, Development of a Theory-based, Sociocultural Instrument to Assess Black Maternal Intentions to Vaccinate Their Daughters Age - Young adults 18-26yrs not included /outside age ranged 9 to 12 Against HPV<br>Cunningham-Erves, Jennifer, Talbott, Laura, O'Neal, Marcia, Ivankova, Nataliya, Wallston, Kenneth, Talbott, Laura L., O'Neal, Marcia R., Ivankova, Nataliya V., Wallston, Kenneth A.<br>Level: 1, State: Excluded	Wrong/no population (eg parents, providers, children, policy makers, does not include 18-26yrs olds; social media posts)
265	RefID: 265, HPV vaccination of college males: Strategizing against HPV infection in college students<br>Cunningham-Erves, Jennifer, Talbott, Laura L.<br>Level: 1, State: Excluded	Wrong/no population (eg parents, providers, children, policy makers, does not include 18-26yrs olds; social media posts)

268	RefID: 268, Preventive innovation: an Australian case study on HPV vaccination<br>D'Souza, C., Mort, G. S., Zyngier, S., Robinson, P., Schlotterlein, M.<br>Level: 2, State: Excluded	Multiple Wrong/no population (eg parents, providers, children, policy makers, does not include 18-26yrs olds; social media posts)s - unable to extract data specific to young adults (18-26 yrs)
269	RefID: 269, Prevalence of HPV infection and genotypes in women with normal cervical cytology in the state of Paraná, Brazil<br>da Silva, M. C., Martins, H. P., de Souza, J. L., Tognim, M. C., Svidzinski, T. I., Teixeira, J. J., Consolaro, M. E.<br>Level: 1, State: Excluded	Not about HPV vaccination/vax attitudes (eg HPV infection/serology/prevalence; cervical cancer; HPV vax safety)
272	RefID: 272, Parental support for human papilloma virus vaccination by adolescents in Ibadan North Local Government Area, Ibadan, Nigeria<br>Dairo, M. D., Adeleke, M. O., Salawu, A. T., Adewole, A. D.<br>Level: 1, State: Excluded	Wrong/no population (eg parents, providers, children, policy makers, does not include 18-26yrs olds; social media posts)
273	RefID: 273, Exploring awareness, attitudes, and perceived role among oral health providers regarding HPV-related oral cancers<br>Daley, E., DeBate, R., Dodd, V., Dyer, K., Fuhrmann, H., Helmy, H., Smith, S. A.<br>Level: 1, State: Excluded	Wrong/no population (eg parents, providers, children, policy makers, does not include 18-26yrs olds; social media posts)
274	RefID: 274, The psychosocial burden of HPV: a mixed-method study of knowledge, attitudes and behaviors among HPV+ women<br>Daley, E. M., Perrin, K. M., McDermott, R. J., Vamos, C. A., Rayko, H. L., Packing-Ebuen, J. L., Webb, C., McFarlane, M.<br>Level: 2, State: Excluded	Not about HPV vaccination/vax attitudes (eg HPV infection/serology/prevalence; cervical cancer; HPV vax safety)
275	RefID: 275, Prevalence of High-Risk Genotypes of Human Papillomavirus: Women Diagnosed with Premalignant and Malignant Pap Smear Tests in Southern Ecuador<br>Dalgo Aguilar, P., Loján González, C., Córdova Rodríguez, A., Acurio Paéz, K., Arévalo, A. P., Bobokova, J.<br>Level: 1, State: Excluded	Not about HPV vaccination/vax attitudes (eg HPV infection/serology/prevalence; cervical cancer; HPV vax safety)
276	RefID: 276, Human papillomavirus (HPV) vaccine coverAge - Young adults 18-26yrs not included /outside age range rates (VCRs) in France: A French claims data study<br>Dalon, F., Majed, L., Belhassen, M., Jacoud, F., Bérard, M., Lévy-Bachelot, L., de Pouvourville, G., Rouzier, R., Raude, J., Baldauf, J. J.<br>Level: 1, State: Excluded	Study Design - Not qualitative (methods or analysis) OR Qualitative survey data not analyzed qualitatively (only numeric stats)
277	RefID: 277, A multilevel approach to accelerating the human papillomavirus (HPV) vaccine at a rural clinic for Native and non-Native youth<br>Dang, J. H. T., LeTran, D., Gori, A., Mojadedi, A., Martens, T., McClure, S., Wadhwa, I., Austin, C., Chen, M. S.<br>Level: 1, State: Excluded	Wrong/no population (eg parents, providers, children, policy makers, does not include 18-26yrs olds; social media posts)
278	RefID: 278, Human Papillomavirus Vaccination in the United States: Uneven Uptake by Gender, Race/Ethnicity, and Sexual Orientation<br>Daniel-Ulloa, J., Gilbert, P. A., Parker, E. A.<br>Level: 1, State: Excluded	Age - Young adults 18-26yrs not included /outside age range
279	RefID: 279, Impact of reduced human papillomavirus vaccination coverAge - Young adults 18-26yrs not included /outside age range rates	Study Design - Not qualitative (methods or analysis) OR

	due to COVID-19 in the United States: A model based analysis<br>Daniels, V., Saxena, K., Roberts, C., Kothari, S., Corman, S., Yao, L., Niccolai, L.<br>Level: 1, State: Excluded	Qualitative survey data not analyzed qualitatively (only numeric stats)
280	RefID: 280, mHealth video gaming for human papillomavirus vaccination among college men-qualitative inquiry for development<br>Darville-Sanders, G., Anderson-Lewis, C., Stellefson, M., Lee, Y. H., MacInnes, J., Pigg, R. M., Mercado, R., Gaddis, C.<br>Level: 2, State: Excluded	Not about HPV vaccination/vax attitudes (eg HPV infection/serology/prevalence; cervical cancer; HPV vax safety)
282	RefID: 282, HPV Vaccine Communication Competency Scale for Medical Trainees: Interdisciplinary Development Study<br>Darville-Sanders, G., Reinoso, H., MacInnes, J., Corluyan, E., Munroe, D., Mathis, M. W., Madden, S. L., Hamrick, J., Dickerson, L., Gaddis, C.<br>Level: 1, State: Excluded	Wrong/no population (eg parents, providers, children, policy makers, does not include 18-26yrs olds; social media posts)
283	RefID: 283, Effect of a School-Based Educational Intervention About the Human Papillomavirus Vaccine on Psychosocial Outcomes Among Adolescents: Analysis of Secondary Outcomes of a Cluster Randomized Trial<br>Davies, C., Marshall, H. S., Zimet, G., McCaffery, K., Brotherton, J. M. L., Kang, M., Garland, S., Kaldor, J., McGeechan, K., Skinner, S. R.<br>Level: 1, State: Excluded	Wrong/no population (eg parents, providers, children, policy makers, does not include 18-26yrs olds; social media posts)
284	RefID: 284, 'Is it like one of those infectious kind of things?' The importance of educating young people about HPV and HPV vaccination at school<br>Davies, Cristyn, Skinner, Susan Rachel, Stoney, Tanya, Marshall, Helen Siobhan, Collins, Joanne, Jones, Jane, Hutton, Heidi, Parrella, Adriana, Cooper, Spring, McGeechan, Kevin, Zimet, Gregory<br>Level: 1, State: Excluded	Study Design - Not qualitative (methods or analysis) OR Qualitative survey data not analyzed qualitatively (only numeric stats)
285	RefID: 285, Vaccinating against HPV: physicians' and medical students' point of view<br>de Carvalho, N. S., Teixeira, L. M., Pradel, E. M., Gabardo, J., Joly, C., Urbanetz, A. A.<br>Level: 1, State: Excluded	Wrong/no population (eg parents, providers, children, policy makers, does not include 18-26yrs olds; social media posts)
287	RefID: 287, Prevalence of oncogenic human papillomavirus in pregnant adolescents, association with colpocytological changes, risk factors and obstetric outcomes<br>de Souza, H. D., Waissman, A. L., Diório, G. R. M., Peres, S. V., Francisco, R. P. V., Galletta, M. A. K.<br>Level: 1, State: Excluded	Not about HPV vaccination/vax attitudes (eg HPV infection/serology/prevalence; cervical cancer; HPV vax safety)
288	RefID: 288, HPV vaccination prevalence, parental barriers and motivators to vaccinating children in Hawai'i<br>Dela Cruz, M. R. I., Braun, K. L., Tsark, J. A. U., Albright, C. L., Chen, J. J.<br>Level: 1, State: Excluded	Wrong/no population (eg parents, providers, children, policy makers, does not include 18-26yrs olds; social media posts)
291	RefID: 291, The efficacy and duration of vaccine protection against human papillomavirus: a systematic review and meta-analysis<br>Deleré, Y., Wichmann, O., Klug, S. J., van der Sande, M., Terhardt, M., Zepp, F., Harder, T.<br>Level: 1, State: Excluded	Wrong publication type (eg reviews, opinions, conference proceedings)

293	RefID: 293, Determinants of vaccine hesitancy in Switzerland: study protocol of a mixed-methods national research programme<br>Deml, M. J., Jafflin, K., Merten, S., Huber, B., Buhl, A., Frau, E., Mettraux, V., Sonderegger, J., Kliem, P., Cattalani, R., Krüerke, D., Pfeiffer, C., Burton-Jeangros, C., Tarr, P. E.<br>Level: 2, State: Excluded	studytype
294	RefID: 294, PCORI Final Research Reports<br>Dempsey, A. F., Maertens, J., Jimenez-Zambrano, A., Sevick, C.<br>Level: 2, State: Excluded	Not about HPV vaccination/vax attitudes (eg HPV infection/serology/prevalence; cervical cancer; HPV vax safety)
295	RefID: 295, Modification and validation of the Treatment Self Regulation Questionnaire to assess parental motivation for HPV vaccination of adolescents<br>Denman, D. C., Baldwin, A. S., Marks, E. G., Lee, S. C., Tiro, J. A.<br>Level: 1, State: Excluded	Wrong/no population (eg parents, providers, children, policy makers, does not include 18-26yrs olds; social media posts)
296	RefID: 296, HPV vaccination in women living with HIV in the United States (133)<br>Desravines, N., Yanek, L., Beavis, A., Powell, A., Keller, J., Anderson, J., Dumas, K., Livingston, A., Levinson, K.<br>Level: 1, State: Excluded	Study Design - Not qualitative (methods or analysis) OR Qualitative survey data not analyzed qualitatively (only numeric stats)
297	RefID: 297, A model for the implementation of cervical cancer screening and human papillomavirus vaccination in the ED: A pilot study<br>Devine, A. S., Mason, J., Rankins, N., Favor, T. T., Baur, V., Parker, R., Baddorf, S., McCarthy, M., Ford, L., Cunningham, T. D.<br>Level: 1, State: Excluded	Study Design - Not qualitative (methods or analysis) OR Qualitative survey data not analyzed qualitatively (only numeric stats)
298	RefID: 298, A Systematic Literature Review of HPV Vaccination Barriers Among Adolescent and Young Adult Males<br>Dibble, K. E., Maksut, J. L., Siembida, E. J., Hutchison, M., Bellizzi, K. M.<br>Level: 1, State: Excluded	Wrong publication type (eg reviews, opinions, conference proceedings)
299	RefID: 299, Is male intent to be vaccinated against HPV a function of the promotion messAge - Young adults 18-26yrs not included /outside age range?<br>DiClemente, R. J., Crosby, R. A., Salazar, L. F., Nash, R., Younge, S.<br>Level: 1, State: Excluded	Study Design - Not qualitative (methods or analysis) OR Qualitative survey data not analyzed qualitatively (only numeric stats)
300	RefID: 300, Characteristics associated with human papillomavirus vaccination initiation and completion among young adults<br>Ding, X., Tian, C., Wang, H., Wang, W., Luo, X.<br>Level: 1, State: Excluded	Study Design - Not qualitative (methods or analysis) OR Qualitative survey data not analyzed qualitatively (only numeric stats)
301	RefID: 301, Mothers' willingness to pay for daughters' HPV vaccine in northern Vietnam<br>Dinh Thu, Ha, Nguyen Thanh, Huong, Hua Thanh, Thuy, Nguyen Hai, Le, Tran Thi, Van, Nguyen Manh, Tri, Buvé, Anne<br>Level: 1, State: Excluded	Wrong/no population (eg parents, providers, children, policy makers, does not include 18-26yrs olds; social media posts)
302	RefID: 302, HPV vaccine knowledge and beliefs among Cambodian American parents and community leaders<br>Do, H., Seng, P., Talbot, J., Acorda, E., Coronado, G. D., Taylor, V. M.<br>Level: 1, State: Excluded	Wrong/no population (eg parents, providers, children, policy makers, does not include 18-26yrs olds; social media posts)

303	RefID: 303, Impact of human papillomavirus-related genital diseases on quality of life and psychosocial wellbeing: results of an observational, health-related quality of life study in the UK<br>Dominiak-Felden, G., Cohet, C., Atrux-Tallau, S., Gilet, H., Tristram, A., Fiander, A.<br>Level: 1, State: Excluded	Not about HPV vaccination/vax attitudes (eg HPV infection/serology/prevalence; cervical cancer; HPV vax safety)
304	RefID: 304, Near real-time surveillance to assess the safety of the 9-valent human papillomavirus vaccine<br>Donahue, J. G., Kieke, B. A., Lewis, E. M., Weintraub, E. S., Hanson, K. E., McClure, D. L., Vickers, E. R., Gee, J., Daley, M. F., DeStefano, F., Hechter, R. C., Jackson, L. A., Klein, N. P., Naleway, A. L., Nelson, J. C., Belongia, E. A.<br>Level: 1, State: Excluded	Study Design - Not qualitative (methods or analysis) OR Qualitative survey data not analyzed qualitatively (only numeric stats)
305	RefID: 305, Measuring vaccine effectiveness against persistent HPV infections: a comparison of different statistical approaches<br>Donken, R., Hoes, J., Knol, M. J., Ogilvie, G. S., Dobson, S., King, A. J., Singer, J., Woestenberg, P. J., Bogaards, J. A., Meijer, Cjlm, de Melker, H. E.<br>Level: 1, State: Excluded	Wrong publication type (eg reviews, opinions, conference proceedings)
307	RefID: 307, Internet and HPV: A possibility for health education among adolescents?<br>Dos Santos, C. A., De Carvalho, F. Z. S., Passos, M. S., Garcia, L. F., Macuch, R. D. S., Bernuci, M. P.<br>Level: 1, State: Excluded	Wrong/no population (eg parents, providers, children, policy makers, does not include 18-26yrs olds; social media posts)
308	RefID: 308, Provider and Practice Experience Integrating the Dose-HPV Intervention into Clinical Practice<br>Drainoni, M. L., Biancarelli, D., Jansen, E., Bernstein, J., Joseph, N., Eun, T. J., Fenton, Ahtr, Clark, J. A., Hanchate, A., Legler, A., Schuch, T. J., Leschly, K., Perkins, R. B.<br>Level: 1, State: Excluded	Wrong/no population (eg parents, providers, children, policy makers, does not include 18-26yrs olds; social media posts)
310	RefID: 310, Awareness and knowledge about human papillomavirus among Latina immigrants<br>Drewry, J., Garcés-Palacio, I. C., Scarinci, I.<br>Level: 1, State: Excluded	Study Design - Not qualitative (methods or analysis) OR Qualitative survey data not analyzed qualitatively (only numeric stats)
311	RefID: 311, Human papillomavirus among medical students at a federal public university<br>Drumond, D. G., de Toledo, L. M. W., Martins, M. B. M., Dias, Z. M. M.<br>Level: 1, State: Excluded	Study Design - Not qualitative (methods or analysis) OR Qualitative survey data not analyzed qualitatively (only numeric stats)
312	RefID: 312, Challenges and opportunities of school-based HPV vaccination in Canada<br>Dubé, E., Gagnon, D., Clément, P., Bettinger, J. A., Comeau, J. L., Deeks, S., Guay, M., MacDonald, S., MacDonald, N. E., Mijovic, H., Paragg, J., Rubincam, C., SauvAge - Young adults 18-26yrs not included /outside age rangeau, C., Steenbeck, A., Wilson, S.<br>Level: 1, State: Excluded	Wrong/no population (eg parents, providers, children, policy makers, does not include 18-26yrs olds; social media posts)
313	RefID: 313, “It takes time to build trust”: a survey Ontario’s school-based HPV immunization program ten years post-implementation	Wrong/no population (eg parents, providers, children, policy makers,

	Dubé, E., Wilson, S., Gagnon, D., Deeks, S. L., Dubey, V.<br>Level: 1, State: Excluded	does not include 18-26yrs olds; social media posts)
315	RefID: 315, Mass gatherings: a review of the scope for meningococcal vaccination in the Indian context<br>Dubey, A. P., Hazarika, R. D., Abitbol, V., Kolhapure, S., Agrawal, S.<br>Level: 1, State: Excluded	Not about HPV vaccination/vax attitudes (eg HPV infection/serology/prevalence; cervical cancer; HPV vax safety)
316	RefID: 316, Shot clinic: Engaging student pharmacists through educational immunization poster competition<br>Duncan, J., Hunt, J., Castillo, S., Ohri, L.<br>Level: 1, State: Excluded	Not about HPV vaccination/vax attitudes (eg HPV infection/serology/prevalence; cervical cancer; HPV vax safety)
317	RefID: 317, Predictors and consequences of conversations about health promoting media messAge - Young adults 18-26yrs not included /outside age ranges<br>Dunlop, S. M., Kashima, Y., Wakefield, M.<br>Level: 1, State: Excluded	Study Design - Not qualitative (methods or analysis) OR Qualitative survey data not analyzed qualitatively (only numeric stats)
318	RefID: 318, "Let's Chat": process evaluation of an intergenerational group chat intervention to increase cancer prevention screening among Vietnamese American families<br>Duong, H. T., Hopfer, S.<br>Level: 2, State: Excluded	Not about HPV vaccination/vax attitudes (eg HPV infection/serology/prevalence; cervical cancer; HPV vax safety)
319	RefID: 319, Let's Chat: Development of a Family Group Chat Cancer Prevention Intervention for Vietnamese Families<br>Duong, H. T., Hopfer, S.<br>Level: 2, State: Excluded	Study Design - Not qualitative (methods or analysis) OR Qualitative survey data not analyzed qualitatively (only numeric stats)
320	RefID: 320, Exploring Intergenerational Communication on Social Media Group Chats as a Cancer Prevention Intervention Opportunity Among Vietnamese American Families: Qualitative Study<br>Duong, H. T., Hopfer, S.<br>Level: 1, State: Excluded	Not about HPV vaccination/vax attitudes (eg HPV infection/serology/prevalence; cervical cancer; HPV vax safety)
321	RefID: 321, Women's knowledge about human papillomavirus and their acceptance of HPV vaccine<br>Dursun, P., Altuntas, B., Kuscu, E., Ayhan, A.<br>Level: 1, State: Excluded	Study Design - Not qualitative (methods or analysis) OR Qualitative survey data not analyzed qualitatively (only numeric stats)
322	RefID: 322, Vaccination's Role in Prevention of Human Papillomavirus (HPV) Infection and the Contribution of Community Nurses<br>Economou, Fotini, Kalemikerakis, Ioannis, Drakopoulou, Marianna, Kavga-Paltoglou, Anna<br>Level: 1, State: Excluded	Wrong publication type (eg reviews, opinions, conference proceedings)
323	RefID: 323, HPV vaccination, knowledge, and attitudes among young cervical cancer survivors in the Deep South<br>Edler, M., Fernandez, A., Anderson, K., Pierce, J. Y., Scalici, J., Daniel, C. L.<br>Level: 2, State: Excluded	Wrong/no population (eg parents, providers, children, policy makers, does not include 18-26yrs olds; social media posts)
324	RefID: 324, Challenges and lessons from a school-based human papillomavirus (HPV) vaccination program for adolescent girls in a rural Nigerian community	Multiple Wrong/no population (eg parents, providers, children, policy makers, does not include 18-26yrs olds; social media posts)s

	Egbon, M., Ojo, T., Aliyu, A., Bagudu, Z. S.<br>Level: 2, State: Excluded	- unable to extract data specific to young adults (18-26 yrs)
325	RefID: 325, Awareness and knowledge associated to Human papillomavirus infection among university students in Morocco: A cross-sectional study<br>El Mansouri, N., Ferrera, L., Kharbach, A., Achbani, A., Kassidi, F., Rogua, H., Ait Wahmane, S., Belmouden, A., Chouham, S., Nejmeddine, M.<br>Level: 1, State: Excluded	Not about HPV vaccination/vax attitudes (eg HPV infection/serology/prevalence; cervical cancer; HPV vax safety)
326	RefID: 326, Awareness and knowledge associated to Human papillomavirus infection among university students in Morocco: A crosssectional study<br>El Mansouri, N., Ferrera, L., Kharbach, A., Achbani, A., Kassidi, F., Rogua, H., Wahmane, S. A., Belmouden, A., Chouham, S., Nejmeddine, M.<br>Level: 1, State: Excluded	Not about HPV vaccination/vax attitudes (eg HPV infection/serology/prevalence; cervical cancer; HPV vax safety)
327	RefID: 327, Human Papillomavirus Infection and Transmission Among Couples Through Heterosexual Activity (HITCH) Cohort Study: Protocol Describing Design, Methods, and Research Goals<br>El-Zein, M., Coutlée, F., Tellier, P. P., Roger, M., Franco, E. L., Burchell, A. N.<br>Level: 1, State: Excluded	Not about HPV vaccination/vax attitudes (eg HPV infection/serology/prevalence; cervical cancer; HPV vax safety)
328	RefID: 328, A Content Analysis of Arabic and English Newspapers before, during, and after the Human Papillomavirus Vaccination Campaign in the United Arab Emirates<br>Elbarazi, I., Raheel, H., Cummings, K., Loney, T.<br>Level: 1, State: Excluded	Wrong publication type (eg reviews, opinions, conference proceedings)
329	RefID: 329, Detection of HPV vaccine-specific antibodies in young women with perinatally acquired HIV: An observational cross-sectional cohort study<br>Elliott, T., Kim, S., Beddows, S., Elegunde, B., Khan, M., Ayres, S., Pasvol, T., Foster, C., Fidler, S.<br>Level: 1, State: Excluded	Not about HPV vaccination/vax attitudes (eg HPV infection/serology/prevalence; cervical cancer; HPV vax safety)
330	RefID: 330, School-based vaccination programs and the HPV vaccine in 16 Appalachian Kentucky school districts: results from a pilot study<br>Ely, G. E., Fields, M., Dignan, M.<br>Level: 1, State: Excluded	Wrong/no population (eg parents, providers, children, policy makers, does not include 18-26yrs olds; social media posts)
331	RefID: 331, Barriers and facilitators of implementing a collaborative HPV vaccine program in an incarcerated Wrong/no population (eg parents, providers, children, policy makers, does not include 18-26yrs olds; social media posts): A case study<br>Emerson, A., Allison, M., Kelly, P. J., Ramaswamy, M.<br>Level: 2, State: Excluded	Wrong/no population (eg parents, providers, children, policy makers, does not include 18-26yrs olds; social media posts)
332	RefID: 332, Awareness, acceptability and uptake of cervical cancer vaccination services among female secondary school teachers in Enugu, Nigeria: a cross-sectional study<br>Enebe, J. T., Enebe, N. O., Agunwa, C. C., Nduagubam, O. C., Okafor, II, Aniwada, E. C., Aguwa, E. N.<br>Level: 1, State: Excluded	Not about HPV vaccination/vax attitudes (eg HPV infection/serology/prevalence; cervical cancer; HPV vax safety)
334	RefID: 334, An observational study comparing HPV prevalence and type distribution between HPV-vaccinated and -unvaccinated girls after	Not about HPV vaccination/vax attitudes (eg HPV

	introduction of school-based HPV vaccination in Norway<br>Enerly, E., Flingtorp, R., Christiansen, I. K., Campbell, S., Hansen, M., Myklebust, TÅ, Weiderpass, E., Nygård, M.<br>Level: 1, State: Excluded	infection/serology/prevalence; cervical cancer; HPV vax safety)
336	RefID: 336, Post-marketing surveillance study of the safety of the HPV-16/18 vaccine in Korea (2017–2021)<br>Eun, B. W., Bahar, E., Xavier, S., Kim, H., Borys, D.<br>Level: 1, State: Excluded	Not about HPV vaccination/vax attitudes (eg HPV infection/serology/prevalence; cervical cancer; HPV vax safety)
337	RefID: 337, Cost-effectiveness of HPV vaccination in the prevention of cervical cancer in Malaysia<br>Ezat, W. P., Aljunid, S.<br>Level: 1, State: Excluded	Study Design - Not qualitative (methods or analysis) OR Qualitative survey data not analyzed qualitatively (only numeric stats)
339	RefID: 339, Awareness and uptake of human papilloma virus vaccines among female secondary school students in Benin City, Nigeria<br>Ezeanochie, M., Olasimbo, P.<br>Level: 1, State: Excluded	Study Design - Not qualitative (methods or analysis) OR Qualitative survey data not analyzed qualitatively (only numeric stats)
340	RefID: 340, Risk factors for HIV positivity among more than 3,400 Tanzanian women<br>Faber, M. T., Munk, C., MwaiselAge - Young adults 18-26yrs not included /outside age range, J., Dartell, M., Kahesa, C., Iftner, T., Rasch, V., Kjaer, S. K.<br>Level: 1, State: Excluded	Study Design - Not qualitative (methods or analysis) OR Qualitative survey data not analyzed qualitatively (only numeric stats)
341	RefID: 341, [Epidemiology of genital warts in female Wrong/no population (eg parents, providers, children, policy makers, does not include 18-26yrs olds; social media posts) of Czech Republic]<br>Fait, T., Dvořák, V., Skřivánek, A., Rokyta, Z., Pilka, R.<br>Level: 1, State: Excluded	Not about HPV vaccination/vax attitudes (eg HPV infection/serology/prevalence; cervical cancer; HPV vax safety)
342	RefID: 342, HPV type-specific prevalence a decade after the implementation of the vaccination program: Results from a pilot study<br>Fappani, C., Bianchi, S., Panatto, D., Petrelli, F., Colzani, D., Scuri, S., Gori, M., Amendola, A., Grappasonni, I., Tanzi, E., Amicizia, D.<br>Level: 1, State: Excluded	Not about HPV vaccination/vax attitudes (eg HPV infection/serology/prevalence; cervical cancer; HPV vax safety)
343	RefID: 343, Understanding Public Perceptions of the HPV Vaccination Based on Online Comments to Canadian News Articles<br>Feinberg, Y., Pereira, J. A., Quach, S., Kwong, J. C., Crowcroft, N. S., Wilson, S. E., Guay, M., Lei, Y., Deeks, S. L.<br>Level: 1, State: Excluded	Wrong publication type (eg reviews, opinions, conference proceedings)
344	RefID: 344, Women's knowledge of human papillomavirus (HPV) and their attitudes toward HPV vaccine: preparing for HPV vaccination in China<br>Feng, S., Xu, X., Jin, Y., Yao, X.<br>Level: 1, State: Excluded	Study Design - Not qualitative (methods or analysis) OR Qualitative survey data not analyzed qualitatively (only numeric stats)
345	RefID: 345, Knowledge, attitudes, and beliefs about human papillomavirus (HPV) vaccination among Puerto Rican mothers and daughters, 2010: a qualitative study<br>Fernández, María E., Le, Yen-Chi L., Fernández-Espada, Natalie, Calo, William A., Savas, Lara S., Vélez, Camille, Aragon, Angela Pattatucci,	Multiple Wrong/no population (eg parents, providers, children, policy makers, does not include 18-26yrs olds; social media posts)s

	Colón-López, Vivian<br>Level: 2, State: Excluded	- unable to extract data specific to young adults (18-26 yrs)
348	RefID: 348, Preferred HPV and HPV Vaccine Learning Methods to Guide Future HPV Prevention Interventions Among Rural Hispanics<br>Fernandez-Pineda, M., Cianelli, R., Villegas, N., Matsuda, Y., Iriarte, E., Fernandez, M., Montano, N. P.<br>Level: 1, State: Excluded	Wrong/no population (eg parents, providers, children, policy makers, does not include 18-26yrs olds; social media posts)
349	RefID: 349, Barriers and facilitators to HPV vaccination of young women in high-income countries: a qualitative systematic review and evidence synthesis<br>Ferrer, H., Trotter, C., Hickman, M., Audrey, S.<br>Level: 1, State: Excluded	Wrong publication type (eg reviews, opinions, conference proceedings)
351	RefID: 351, Association between cervical dysplasia and human papillomavirus in HIV seropositive women from Johannesburg South Africa<br>Firnhaber, Cynthia, Van Le, Hoa, Pettifor, Audrey, Schulze, Doreen, Michelow, Pam, Sanne, Ian M., Lewis, David A., Williamson, Anna-Lise, Allan, Bruce, Williams, Sophia, Rinas, Allen, Levin, Simon, Smith, Jennifer S.<br>Level: 1, State: Excluded	Not about HPV vaccination/vax attitudes (eg HPV infection/serology/prevalence; cervical cancer; HPV vax safety)
353	RefID: 353, Co-production of an educational packAge - Young adults 18-26yrs not included /outside age range for the universal human papillomavirus (HPV) vaccination programme tailored for schools with low uptake: a participatory study protocol<br>Fisher, H., Audrey, S., Chantler, T., Finn, A., Letley, L., Mounier-Jack, S., Thomas, C., Yates, J., Hickman, M.<br>Level: 1, State: Excluded	Wrong/no population (eg parents, providers, children, policy makers, does not include 18-26yrs olds; social media posts)
354	RefID: 354, Development of an educational packAge - Young adults 18-26yrs not included /outside age range for the universal human papillomavirus (HPV) vaccination programme: a co-production study with young people and key informants<br>Fisher, H., Chantler, T., Finn, A., Kesten, J., Hickman, M., Letley, L., Mounier-Jack, S., Thomas, C., Worthington, K., Yates, J., Audrey, S.<br>Level: 1, State: Excluded	Wrong/no population (eg parents, providers, children, policy makers, does not include 18-26yrs olds; social media posts)
355	RefID: 355, Human papillomavirus vaccine communication materials for young people in English-speaking countries: A content analysis<br>Fisher, H., Chantler, T., Mounier-Jack, S., Audrey, S.<br>Level: 1, State: Excluded	Wrong/no population (eg parents, providers, children, policy makers, does not include 18-26yrs olds; social media posts)
356	RefID: 356, Young women's autonomy and information needs in the schools-based hpv vaccination programme: a qualitative study<br>Fisher, H., Evans, K., Ferrie, J., Yates, J., Roderick, M., Audrey, S.<br>Level: 2, State: Excluded	Wrong/no population (eg parents, providers, children, policy makers, does not include 18-26yrs olds; social media posts)
357	RefID: 357, Prevalence of anogenital warts among participants in private health plans in the United States, 2006-2014: Potential impact of hpv vaccination<br>Flagg, E. W.<br>Level: 1, State: Excluded	Not about HPV vaccination/vax attitudes (eg HPV infection/serology/prevalence; cervical cancer; HPV vax safety)
358	RefID: 358, Addressing the need for education and early detection services for HPV and cervical cancer in the Denver Latina community	studytype

	Flink, D. M., Wheeler, L. J., Chavez, S., Carroll, K. M., Russum, M.<br>Level: 2, State: Excluded	
359	RefID: 359, Long-term Cross-reactivity against Nonvaccine Human Papillomavirus Types 31 and 45 after 2- or 3-Dose Schedules of the AS04-Adjuvanted Human HPV-16/18 Vaccine<br>Folschweiller, N., Behre, U., Dionne, M., Durando, P., Esposito, S., Ferguson, L., Ferguson, M., Hillemanns, P., McNeil, S. A., Peters, K., et al.<br>Level: 1, State: Excluded	Not about HPV vaccination/vax attitudes (eg HPV infection/serology/prevalence; cervical cancer; HPV vax safety)
360	RefID: 360, College males' HPV risks, vaccination rates, and vaccine perceptions<br>Fontenot, H., Fantasia, H., Sutherland, M.<br>Level: 1, State: Excluded	Wrong publication type (eg reviews, opinions, conference proceedings)
361	RefID: 361, The Impact of Advertisement Messaging on Enrollment of Young Men Who Have Sex With Men for Web-Based Research: Observational Study<br>Fontenot, H. B., Abuelezam, N. N., Rosenberger, J. G., Novak, D., Mayer, K. H., Zimet, G.<br>Level: 1, State: Excluded	Study Design - Not qualitative (methods or analysis) OR Qualitative survey data not analyzed qualitatively (only numeric stats)
363	RefID: 363, YMSM's preferences and perspectives for a mobile health tool designed facilitate HPV vaccination<br>Fontenot, H. B., Rosenberger, J. G., Mayer, K. H., Zimet, G. D.<br>Level: 2, State: Excluded	studytype
364	RefID: 364, Perspectives and preferences for a mobile health tool designed to facilitate HPV vaccination among young men who have sex with men<br>Fontenot, H. B., Rosenberger, J. G., McNair, K. T., Mayer, K. H., Zimet, G.<br>Level: 2, State: Excluded	Not about HPV vaccination/vax attitudes (eg HPV infection/serology/prevalence; cervical cancer; HPV vax safety)
365	RefID: 365, Chlamydia Vaccination: Parent Opinions and Implications for Future Promotion Programs<br>Footman, A., Kanney, N., Niccolai, L. M., Zimet, G. D., Overton, E. T., Davies, S. L., Van Der Pol, B.<br>Level: 1, State: Excluded	Not about HPV vaccination/vax attitudes (eg HPV infection/serology/prevalence; cervical cancer; HPV vax safety)
366	RefID: 366, Increasing adolescent vaccination: barriers and strategies in the context of policy, legal, and financial issues<br>Ford, C. A., English, A., Davenport, A. F., Stinnett, A. J.<br>Level: 1, State: Excluded	Wrong/no population (eg parents, providers, children, policy makers, does not include 18-26yrs olds; social media posts)
367	RefID: 367, Options for design of real-world impact studies of single-dose vaccine schedules<br>Franceschi, S., Clifford, G. M., Baussano, I.<br>Level: 1, State: Excluded	Wrong/no population (eg parents, providers, children, policy makers, does not include 18-26yrs olds; social media posts)
368	RefID: 368, A qualitative analysis of South African women's knowledge, attitudes, and beliefs about HPV and cervical cancer prevention, vaccine awareness and acceptance, and maternal-child communication about sexual health<br>Francis, S. A., Battle-Fisher, M., Liverpool, J., Hipple, L., Mosavel, M., Soogun, S., Mofammere, N.<br>Level: 2, State: Excluded	Multiple Wrong/no population (eg parents, providers, children, policy makers, does not include 18-26yrs olds; social media posts)s - unable to extract data specific to young adults (18-26 yrs)

369	RefID: 369, The HPV vaccine: a comparison of focus groups conducted in South Africa and Ohio Appalachia<br>Francis, S. A., Katz, M. L.<br>Level: 2, State: Excluded	Multiple Wrong/no population (eg parents, providers, children, policy makers, does not include 18-26yrs olds; social media posts)s - unable to extract data specific to young adults (18-26 yrs)
370	RefID: 370, Human Papillomavirus knowledge and vaccine status among university students: How have health care providers influenced the vaccine decision?<br>Freudenthaler, Tracy<br>Level: 2, State: Excluded	studytype
371	RefID: 371, The Sexual behavior and protective conduct among university students in Germany - Chances and approaches to tackle spread of sexually transmitted diseases<br>Fuchs, C., Teichert, N., Neu, K., Clanner-Engelshofen, B., Zippel, S., French, L. E., Reinholz, M.<br>Level: 1, State: Excluded	Not about HPV vaccination/vax attitudes (eg HPV infection/serology/prevalence; cervical cancer; HPV vax safety)
372	RefID: 372, HPV vaccine acceptance among African-American mothers and their daughters: an inquiry grounded in culture<br>Galbraith-Gyan, K. V., Lechuga, J., Jenerette, C. M., Palmer, M. H., Moore, A. D., Hamilton, J. B.<br>Level: 2, State: Excluded	Wrong/no population (eg parents, providers, children, policy makers, does not include 18-26yrs olds; social media posts)
373	RefID: 373, HPV knowledge, burden and genital wart location among heterosexually identified versus homosexually identified men who have sex with men in Lima, Peru: cross-sectional results from a cohort study<br>Galea, J. T., León, S. R., Peinado, J., Calvo, G., Zamora, J., Sánchez, H., Brown, B. J.<br>Level: 1, State: Excluded	Not about HPV vaccination/vax attitudes (eg HPV infection/serology/prevalence; cervical cancer; HPV vax safety)
374	RefID: 374, HPV vaccine knowledge and acceptability among Peruvian men who have sex with men and transgender women: A pilot, qualitative study<br>Galea, J. T., Monsour, E., Nureña, C. R., Blas, M. M., Brown, B.<br>Level: 2, State: Excluded	Multiple Wrong/no population (eg parents, providers, children, policy makers, does not include 18-26yrs olds; social media posts)s - unable to extract data specific to young adults (18-26 yrs)
375	RefID: 375, Human papillomavirus (HPV) vaccine coverAge - Young adults 18-26yrs not included /outside age range achievements in low and middle-income countries 2007-2016<br>Gallagher, K. E., Howard, N., Kabakama, S., Mounier-Jack, S., Burchett, H. E. D., LaMontagne, D. S., Watson-Jones, D.<br>Level: 1, State: Excluded	Wrong publication type (eg reviews, opinions, conference proceedings)
377	RefID: 377, Awareness and knowledge about cervical cancer prevention methods among Tunisian women<br>Gamaoun, R.<br>Level: 1, State: Excluded	Study Design - Not qualitative (methods or analysis) OR Qualitative survey data not analyzed qualitatively (only numeric stats)
378	RefID: 378, Vaccination concerns, beliefs and practices among Ukrainian migrants in Poland: a qualitative study<br>Ganczak, M., Bielecki, K., Drozd-Dąbrowska, M., Topczewska, K., Biesiada, D., Molas-Biesiada, A., Dubiel, P., Gorman, D.<br>Level: 1, State: Excluded	Not about HPV vaccination/vax attitudes (eg HPV infection/serology/prevalence; cervical cancer; HPV vax safety)

379	RefID: 379, Health System Barriers to Child Mandatory and Optional Vaccination among Ukrainian Migrants in Poland in the Context of MMR and HPV Vaccines-A Qualitative Study<br>Ganczak, M., Kalinowski, P., Pasek, O., Duda-Duma, Ł, Sobieraj, E., Goławski, J., Biesiada, D., Jansen, D., Vervoort, J. P. M., Edelstein, M., Kowalska, M.<br>Level: 2, State: Excluded	Not about HPV vaccination/vax attitudes (eg HPV infection/serology/prevalence; cervical cancer; HPV vax safety)
381	RefID: 381, Focus Group Study of Chinese International Students' Knowledge and Beliefs About HPV Vaccination, Before and After Reading an Informational Pamphlet About Gardasil<br>Gao, Haijuan, Okoror, Titilayo, Hyner, Gerald<br>Level: 2, State: Excluded	Multiple Wrong/no population (eg parents, providers, children, policy makers, does not include 18-26yrs olds; social media posts)s - unable to extract data specific to young adults (18-26 yrs)
383	RefID: 383, Retrospective Review of Sexual and Reproductive Health Conversations During Initial Visits of Adolescents Seeking Gender-Affirming Testosterone<br>Garborcauskas, G., Boskey, E. R., Guss, C. E., Grimstad, F. W.<br>Level: 1, State: Excluded	Wrong publication type (eg reviews, opinions, conference proceedings)
384	RefID: 384, Barriers and facilitators to HPV vaccination in primary care practices: a mixed methods study using the Consolidated Framework for Implementation Research<br>Garbutt, J. M., Dodd, S., Walling, E., Lee, A. A., Kulka, K., Lobb, R.<br>Level: 1, State: Excluded	Wrong/no population (eg parents, providers, children, policy makers, does not include 18-26yrs olds; social media posts)
387	RefID: 387, Development, theoretical framework, and evaluation from implementation of a parent and teacherdelivered intervention to enhance adolescent vaccination<br>Gargano, L. M., Herbert, N. L., Painter, J. E., Sales, J. M., Morfaw, C., Jones, L. M., Murray, D., DiClemente, R. J., Hughes, J. M.<br>Level: 1, State: Excluded	Study Design - Not qualitative (methods or analysis) OR Qualitative survey data not analyzed qualitatively (only numeric stats)
388	RefID: 388, Development, Theoretical Framework, and Evaluation of a Parent and Teacher–Delivered Intervention on Adolescent Vaccination<br>Gargano, Lisa M., Herbert, Natasha L., Painter, Julia E., Sales, Jessica M., Vogt, Tara M., Morfaw, Christopher, Jones, LaDawna M., Murray, Dennis, DiClemente, Ralph J., Hughes, James M.<br>Level: 1, State: Excluded	Study Design - Not qualitative (methods or analysis) OR Qualitative survey data not analyzed qualitatively (only numeric stats)
389	RefID: 389, Community-based household assessment of human papillomavirus (HPV) vaccination coverAge - Young adults 18-26yrs not included /outside age range and acceptability - HPV vaccine demonstration program, Cambodia - 2017<br>Garon, J., Wuddhika, I. V., Sreenivasan, N., Wannemuehler, K., Vutthikol, Y., Chhorvann, C., Loharikar, A.<br>Level: 1, State: Excluded	Not about HPV vaccination/vax attitudes (eg HPV infection/serology/prevalence; cervical cancer; HPV vax safety)
391	RefID: 391, CoverAge - Young adults 18-26yrs not included /outside age range of the human papillomavirus vaccine by US and Canadian medical schools<br>Gee, R. E., Shacter, H. E., Long, J. A.<br>Level: 1, State: Excluded	Not about HPV vaccination/vax attitudes (eg HPV infection/serology/prevalence; cervical cancer; HPV vax safety)
392	RefID: 392, Racial Disparities in Human Papillomavirus Vaccination: Does Access Matter?<br>Gelman, Amanda, Miller, Elizabeth, Bimla Schwarz, Eleanor, Akers,	Age - Young adults 18-26yrs not included /outside age range

	Aletha Y., Kwonho, Jeonge, Borrero, Sonya<br>Level: 1, State: Excluded	
393	RefID: 393, Promoting sexual health and disease prevention: Embracing advances in behavioural and therapeutic approaches towards ending hiv and human papillomavirus related diseases in the Bahamas<br>George, C.<br>Level: 2, State: Excluded	studytype
395	RefID: 395, Predictors of human papillomavirus vaccination among daughters of low-income Latina mothers: the role of acculturation<br>Gerend, M. A., Zapata, C., Reyes, E.<br>Level: 1, State: Excluded	Study Design - Not qualitative (methods or analysis) OR Qualitative survey data not analyzed qualitatively (only numeric stats)
396	RefID: 396, Factors Influencing the Intention of Getting the HPV Vaccine among College Women: An Application of the Reasoned Action Approach<br>Geshnizjani, Alireza, Jozkowski, Kristen N., Middlestadt, Susan E.<br>Level: 2, State: Excluded	Study Design - Not qualitative (methods or analysis) OR Qualitative survey data not analyzed qualitatively (only numeric stats)
397	RefID: 397, A retrospective analysis of the costs and manAge - Young adults 18-26yrs not included /outside age rangement of genital warts in Italy<br>Gianino, M. M., Delmonte, S., Lovato, E., Martinese, M., Rondoletti, S., Bernengo, M. G., Zotti, C. M.<br>Level: 1, State: Excluded	Not about HPV vaccination/vax attitudes (eg HPV infection/serology/prevalence; cervical cancer; HPV vax safety)
398	RefID: 398, Acceptabililty of HPV vaccination among women attending the University of Saskatchewan student health services<br>Giede, C., McFadden, L., Komonoski, P., Agrawal, A., Stauffer, A., Pierson, R.<br>Level: 2, State: Excluded	studytype
399	RefID: 399, The acceptability of HPV vaccination among women attending the University of Saskatchewan Student Health Services<br>Giede, C., McFadden, L. L., Komonoski, P., Agrawal, A., Stauffer, A., Pierson, R.<br>Level: 2, State: Excluded	Study Design - Not qualitative (methods or analysis) OR Qualitative survey data not analyzed qualitatively (only numeric stats)
400	RefID: 400, Antibody persistence after a single dose of quadrivalent HPV vaccine and the effect of a dose of nonavalent vaccine given 3-8 years later–an exploratory study<br>Gilca, V., SauvAge - Young adults 18-26yrs not included /outside age rangeau, C., Panicker, G., De Serres, G., Ouakki, M., Unger, E. R.<br>Level: 1, State: Excluded	Not about HPV vaccination/vax attitudes (eg HPV infection/serology/prevalence; cervical cancer; HPV vax safety)
401	RefID: 401, Assessing the Relationship Between Motherhood and Cervical Cancer Screening and Prevention Behaviors<br>Ginocchi, Annalisa, Rogan, Erika M., Conley, Claire C.<br>Level: 1, State: Excluded	Study Design - Not qualitative (methods or analysis) OR Qualitative survey data not analyzed qualitatively (only numeric stats)
402	RefID: 402, Prevalence of cervical human papillomavirus infection and types among women immigrated to Sicily, Italy<br>Giovannelli, L., Vassallo, R., Matranga, D., Affronti, M., Caleca, M. P., Bellavia, C., Perino, A., Ammatuna, P.<br>Level: 1, State: Excluded	Not about HPV vaccination/vax attitudes (eg HPV infection/serology/prevalence; cervical cancer; HPV vax safety)

403	RefID: 403, Reproductive health concerns of parents of girls with cerebral palsy and intellectual disability<br>Glader, L., Christensen, S., Khan, A., Williams, D., Gray, S.<br>Level: 1, State: Excluded	Not about HPV vaccination/vax attitudes (eg HPV infection/serology/prevalence; cervical cancer; HPV vax safety)
405	RefID: 405, Market segmentation and targeted messaging to improve HPV vaccine intentions in the college Age - Young adults 18-26yrs not included /outside age ranged Wrong/no population (eg parents, providers, children, policy makers, does not include 18-26yrs olds; social media posts)<br>Godwin, Ashley Caitlin<br>Level: 2, State: Excluded	studytype
406	RefID: 406, Case 10-2009: A 23-year-old woman with an abnormal papanicolaou smear<br>Goldstein, M. A., Goodman, A., Del Carmen, M. G., Wilbur, D. C.<br>Level: 1, State: Excluded	Not about HPV vaccination/vax attitudes (eg HPV infection/serology/prevalence; cervical cancer; HPV vax safety)
407	RefID: 407, Characterization of Immunoglobulin A/G Responses during 3 Doses of the Human Papillomavirus-16/18 ASO4-Adjuvanted Vaccine<br>Gonçalves, A. K., Giraldo, P. C., Farias, K. J., Machado, P. R., Costa, A. P. F., De Souza, L. C., Crispim, J. C., Eleutério, J., Witkin, S. S.<br>Level: 1, State: Excluded	Not about HPV vaccination/vax attitudes (eg HPV infection/serology/prevalence; cervical cancer; HPV vax safety)
408	RefID: 408, Rationale and design of a long term follow-up study of women who did and did not receive HPV 16/18 vaccination in Guanacaste, Costa Rica<br>Gonzalez, P., Hildesheim, A., Herrero, R., Katki, H., Wacholder, S., Porras, C., Safaeian, M., Jimenez, S., Darragh, T. M., Cortes, B., Befano, B., Schiffman, M., Carvajal, L., Palefsky, J., Schiller, J., Ocampo, R., Schussler, J., Lowy, D., Guillen, D., Stoler, M. H., Quint, W., Morales, J., Avila, C., Rodriguez, A. C., Kreimer, A. R.<br>Level: 1, State: Excluded	Not about HPV vaccination/vax attitudes (eg HPV infection/serology/prevalence; cervical cancer; HPV vax safety)
410	RefID: 410, Candidate HPV genotypes not included in the 9-valent vaccine for prevention of CIN 2-3<br>Gonzalez-Bosquet, E., Gibert, M., Serra, M., Hernandez-Saborit, A., Gonzalez-Fernandez, A.<br>Level: 1, State: Excluded	Not about HPV vaccination/vax attitudes (eg HPV infection/serology/prevalence; cervical cancer; HPV vax safety)
411	RefID: 411, HPV Vaccine Initiation and Completion Among Native Hawaiian and Pacific Islander Adults, United States, 2014<br>Gopalani, S. V., Janitz, A. E., Martinez, S. A., Campbell, J. E., Chen, S.<br>Level: 1, State: Excluded	Study Design - Not qualitative (methods or analysis) OR Qualitative survey data not analyzed qualitatively (only numeric stats)
412	RefID: 412, A qualitative study of vaccination behaviour amongst female Polish migrants in Edinburgh, Scotland<br>Gorman, D. R., Bielecki, K., Willocks, L. J., Pollock, K. G.<br>Level: 2, State: Excluded	Wrong/no population (eg parents, providers, children, policy makers, does not include 18-26yrs olds; social media posts)
413	RefID: 413, Availability of human papillomavirus vaccine at medical practices in an area with elevated rates of cervical cancer<br>Gottlieb, S. L., Brewer, N. T., Smith, J. S., Keating, K. M., Markowitz, L. E.<br>Level: 1, State: Excluded	Not about HPV vaccination/vax attitudes (eg HPV infection/serology/prevalence; cervical cancer; HPV vax safety)
414	RefID: 414, Human papillomavirus vaccine initiation in an area with elevated rates of cervical cancer	Age - Young adults 18-26yrs not included /outside age range

	Gottlieb, S. L., Brewer, N. T., Sternberg, M. R., Smith, J. S., Ziarnowski, K., Liddon, N., Markowitz, L. E.<br>Level: 1, State: Excluded	
415	RefID: 415, Catch-up HPV vaccination status of adolescents in relation to socioeconomic factors, individual beliefs and sexual behaviour<br>Grandahl, M., Larsson, M., Dalianis, T., Stenhammar, C., Tydén, T., Westerling, R., Nevéus, T.<br>Level: 1, State: Excluded	Study Design - Not qualitative (methods or analysis) OR Qualitative survey data not analyzed qualitatively (only numeric stats)
416	RefID: 416, Barriers towards HPV vaccinations for boys and young men: a narrative review<br>Grandahl, M., Nevéus, T.<br>Level: 1, State: Excluded	Wrong publication type (eg reviews, opinions, conference proceedings)
417	RefID: 417, 'I also want to be vaccinated!' - adolescent boys' awareness and thoughts, perceived benefits, information sources, and intention to be vaccinated against Human papillomavirus (HPV)<br>Grandahl, M., Nevéus, T., Dalianis, T., Larsson, M., Tydén, T., Stenhammar, C.<br>Level: 2, State: Excluded	Multiple Wrong/no population (eg parents, providers, children, policy makers, does not include 18-26yrs olds; social media posts)s - unable to extract data specific to young adults (18-26 yrs)
419	RefID: 419, School-based intervention for the prevention of HPV among adolescents: a cluster randomised controlled study<br>Grandahl, M., Rosenblad, A., Stenhammar, C., Tydén, T., Westerling, R., Larsson, M., Oscarsson, M., Andrae, B., Dalianis, T., Nevéus, T.<br>Level: 1, State: Excluded	Not about HPV vaccination/vax attitudes (eg HPV infection/serology/prevalence; cervical cancer; HPV vax safety)
420	RefID: 420, Immigrant women's experiences and views on the prevention of cervical cancer: a qualitative study<br>Grandahl, M., Tydén, T., Gottvall, M., Westerling, R., Oscarsson, M.<br>Level: 2, State: Excluded	Multiple Wrong/no population (eg parents, providers, children, policy makers, does not include 18-26yrs olds; social media posts)s - unable to extract data specific to young adults (18-26 yrs)
422	RefID: 422, Occurrence of human papillomavirus (HPV) type replacement by sexual risk-taking behaviour group: post-hoc analysis of a community randomized clinical trial up to 9 years after vaccination (IV)<br>Gray, P., Luostarinen, T., Vänskä, S., Eriksson, T., Lagheden, C., Man, I., Palmroth, J., Pimenoff, V. N., Söderlund-Strand, A., Dillner, J., et al.<br>Level: 1, State: Excluded	Not about HPV vaccination/vax attitudes (eg HPV infection/serology/prevalence; cervical cancer; HPV vax safety)
424	RefID: 424, Safety and immunogenicity of the quadrivalent human papillomavirus vaccine in patients with juvenile dermatomyositis: a real-world multicentre study<br>Grein, I. H. R., Pinto, N. B. F., Groot, N., Martins, C. B., Lobo, A., Aikawa, N. E., Barbosa, C., Terreri, M. T., da Fraga, A. C. M., de Oliveira, S. K. F., Sztajnbok, F., Paim Marques, L. B., Islabão, A. G., Appenzeller, S., Bica, B., de Oliveira Sato, J., Magalhães, C. S., Ferriani, V., Pasmans, H., Schepp, R., van der Klis, F., de Roock, S., Wulffraat, N., Pileggi, G. S.<br>Level: 1, State: Excluded	Not about HPV vaccination/vax attitudes (eg HPV infection/serology/prevalence; cervical cancer; HPV vax safety)
425	RefID: 425, Agreement between patients' self-report and medical records for vaccination: the PGRx database<br>Grimaldi-Bensouda, L., Aubrun, E., Leighton, P., Benichou, J., Rossignol, M., Abenhaim, L.<br>Level: 1, State: Excluded	Age - Young adults 18-26yrs not included /outside age range

426	RefID: 426, Seductive Delusions: How Everyday People Catch STIs<br>Grimes, J.<br>Level: 1, State: Excluded	Wrong publication type (eg reviews, opinions, conference proceedings)
427	RefID: 427, Incidence of new-onset autoimmune disease in girls and women with pre-existing autoimmune disease after quadrivalent human papillomavirus vaccination: a cohort study<br>Grönlund, O., Herweijer, E., Sundström, K., Arnheim-Dahlström, L.<br>Level: 1, State: Excluded	Not about HPV vaccination/vax attitudes (eg HPV infection/serology/prevalence; cervical cancer; HPV vax safety)
428	RefID: 428, Prevalence of HPV infection among sexually active adolescents and young adults in Brazil: the POP-Brazil Study<br>Group, P. OP-Brazil Study, Wendland, E. M., Villa, L. L., Unger, E. R., Domingues, C. M., Benzaken, A. S.<br>Level: 1, State: Excluded	Not about HPV vaccination/vax attitudes (eg HPV infection/serology/prevalence; cervical cancer; HPV vax safety)
429	RefID: 429, What is the most useful tool in HPV vaccine promotion? Results from an experimental study<br>Gualano, M. R., Thomas, R., Stillo, M., Mussa, M. V., Quattrocolo, F., Borraccino, A., Zotti, C.<br>Level: 1, State: Excluded	Study Design - Not qualitative (methods or analysis) OR Qualitative survey data not analyzed qualitatively (only numeric stats)
430	RefID: 430, Determinants of human papilloma virus vaccination (HPVV) among Quebec (Canada) teenAge - Young adults 18-26yrs not included /outside age rangers<br>Guay, M., Clément, P., Hamid, A., Dubé, E., SauvAge - Young adults 18-26yrs not included /outside age rangeau, C., Boulianne, N., Landry, M., Lemaire, J.<br>Level: 1, State: Excluded	Wrong/no population (eg parents, providers, children, policy makers, does not include 18-26yrs olds; social media posts)
431	RefID: 431, Determinants of vaccination against the human papillomavirus among QuEbec teenAge - Young adults 18-26yrs not included /outside age rangers: Parental and teen perspectives<br>Guay, M., Clément, P., Hamid, A., Dubé, È, SauveAge - Young adults 18-26yrs not included /outside age rangeau, C., Boulianne, N., Landry, M., Lemaire, J.<br>Level: 1, State: Excluded	Age - Young adults 18-26yrs not included /outside age range
432	RefID: 432, Comparison of HPV prevalence between HPV-vaccinated and non-vaccinated young adult women (20-26 years)<br>Guo, F., Hirth, J. M., Berenson, A. B.<br>Level: 1, State: Excluded	Study Design - Not qualitative (methods or analysis) OR Qualitative survey data not analyzed qualitatively (only numeric stats)
433	RefID: 433, Human Papillomavirus Vaccination and Pap Smear Uptake Among Young Women in the United States: Role of Provider and Patient<br>Guo, F., Hirth, J. M., Berenson, A. B.<br>Level: 1, State: Excluded	Not about HPV vaccination/vax attitudes (eg HPV infection/serology/prevalence; cervical cancer; HPV vax safety)
434	RefID: 434, Socioeconomic inequalities to accessing vaccination against human papillomavirus in France: results of the health, health care and insurance survey, 2012<br>Guthmann, J. P., Pelat, C., Célant, N., Chatelet, I. P. du, Duport, N., Rochereau, T., Lévy-Bruhl, D.<br>Level: 1, State: Excluded	Age - Young adults 18-26yrs not included /outside age range

436	RefID: 436, Acceptability of the human papillomavirus vaccine among urban adolescent males<br>Gutierrez, B., Jr., Leung, A., Jones, K. T., Smith, P., Silverman, R., Frank, I., Leader, A. E.<br>Level: 2, State: Excluded	Multiple Wrong/no population (eg parents, providers, children, policy makers, does not include 18-26yrs olds; social media posts)s - unable to extract data specific to young adults (18-26 yrs)
438	RefID: 438, Strategies and ethical considerations for the recruitment of young men who have sex with men: challenges of a vaccination trial in Mexico<br>Gutiérrez-Luna, A., Angeles-Llerenas, A., Wirtz, V. J., Del Río, A. A., Zamilpa-Mejía, L., Aranda-Flores, C., Viramontes, J. L., Lazcano-Ponce, E.<br>Level: 1, State: Excluded	Not about HPV vaccination/vax attitudes (eg HPV infection/serology/prevalence; cervical cancer; HPV vax safety)
439	RefID: 439, A case-control study to evaluate awareness level of human papillomavirus among women healthcare professionals in tertiary health care facility<br>Güven, S. E., Sağlam, Z. A., İnci, A.<br>Level: 1, State: Excluded	Study Design - Not qualitative (methods or analysis) OR Qualitative survey data not analyzed qualitatively (only numeric stats)
440	RefID: 440, The HPV vaccine: a content analysis of online news stories<br>Habel, M. A., Liddon, N., Stryker, J. E.<br>Level: 1, State: Excluded	Wrong/no population (eg parents, providers, children, policy makers, does not include 18-26yrs olds; social media posts)
441	RefID: 441, A cross-sectional study to assess the awareness and practices related to adult immunization among nursing students in a metropolitan city<br>Hadaye, R. S., Shastri, S., Lavangare, S. R.<br>Level: 1, State: Excluded	Not about HPV vaccination/vax attitudes (eg HPV infection/serology/prevalence; cervical cancer; HPV vax safety)
442	RefID: 442, Disparities of perceptions and practices related to cervical cancer prevention and the acceptability of HPV vaccination according to educational level in a French cross-sectional survey of 18-65 years old women<br>Haesebaert, J., Lutringer-Magnin, D., Kalecinski, J., Barone, G., Jacquard, A. C., Leocmach, Y., Régnier, V., Vanhems, P., Chauvin, F., Lasset, C.<br>Level: 1, State: Excluded	Age - Young adults 18-26yrs not included /outside age range
443	RefID: 443, Prevalence of Genital Human Papillomavirus Infection and Human Papillomavirus Vaccination Rates Among US Adult Men: National Health and Nutrition Examination Survey (NHANES) 2013-2014<br>Han, J. J., Beltran, T. H., Song, J. W., Klaric, J., Choi, Y. S.<br>Level: 1, State: Excluded	Age - Young adults 18-26yrs not included /outside age range
444	RefID: 444, Acceptability of School-Based Health Centers for Human Papillomavirus Vaccination Visits: A Mixed-Methods Study<br>Hansen, Caitlin E., Okoloko, Edirin, Ogunbajo, Adedotun, North, Anna, Niccolai, Linda M.<br>Level: 2, State: Excluded	Multiple Wrong/no population (eg parents, providers, children, policy makers, does not include 18-26yrs olds; social media posts)s - unable to extract data specific to young adults (18-26 yrs)
445	RefID: 445, HPV vaccination initiation among white, black and Middle East North African (MENA) males<br>Harper, D. M., Rego, R., Tariq, M., Patel, M. R., Resnicow, K., Sheinfeld Gorin, S.<br>Level: 1, State: Excluded	Not about HPV vaccination/vax attitudes (eg HPV infection/serology/prevalence; cervical cancer; HPV vax safety)

446	RefID: 446, The role of trust in HPV vaccine uptake among racial and ethnic minorities in the United States: a narrative review<br>Harrington, N., Chen, Y., O'Reilly, A. M., Fang, C. Y.<br>Level: 1, State: Excluded	Wrong publication type (eg reviews, opinions, conference proceedings)
447	RefID: 447, Human Papillomavirus vaccination clinical decision support for young adults in an upper midwestern healthcare system: a clinic cluster-randomized control trial<br>Harry, M. L., Asche, S. E., Freitag, L. A., Sperl-Hillen, J. M., Saman, D. M., Ekstrom, H. L., Chrenka, E. A., Truitt, A. R., Allen, C. I., O'Connor, P. J., et al.<br>Level: 1, State: Excluded	Wrong/no population (eg parents, providers, children, policy makers, does not include 18-26yrs olds; social media posts)
448	RefID: 448, Early lessons learned from extramural school programs that offer HPV vaccine<br>Hayes, K. A., Entzel, P., Berger, W., Caskey, R. N., Shlay, J. C., Stubbs, B. W., Smith, J. S., Brewer, N. T.<br>Level: 1, State: Excluded	Wrong/no population (eg parents, providers, children, policy makers, does not include 18-26yrs olds; social media posts)
450	RefID: 450, Health care providers' perspectives on low HPV vaccine uptake and adherence in Appalachian Kentucky<br>Head, K. J., Vanderpool, R. C., Mills, L. A.<br>Level: 2, State: Excluded	Wrong/no population (eg parents, providers, children, policy makers, does not include 18-26yrs olds; social media posts)
451	RefID: 451, HPV Vaccine Intent among Adult Women Receiving Care at Community Health Centers<br>Hecht, M. L., BeLue, R., Ray, A., Hopfer, S., Miller-Day, M., McKee, F.<br>Level: 1, State: Excluded	Study Design - Not qualitative (methods or analysis) OR Qualitative survey data not analyzed qualitatively (only numeric stats)
452	RefID: 452, HPV Vaccination Practices Among Juvenile Justice Facilities in the United States<br>Henderson, C. E., Rich, J. D., Lally, M. A.<br>Level: 1, State: Excluded	Study Design - Not qualitative (methods or analysis) OR Qualitative survey data not analyzed qualitatively (only numeric stats)
453	RefID: 453, Talking about human papillomavirus and cancer: development of consultation guides through lay and professional stakeholder coproduction using qualitative, quantitative and secondary data<br>Hendry, M., Pasterfield, D., Gollins, S., Adams, R., Evans, M., Fiander, A., Robling, M., Campbell, C., Bekkers, M. J., Hiscock, J., Nafees, S., Rose, J., Stanley, M., Williams, O., Makin, M., Wilkinson, C.<br>Level: 1, State: Excluded	Wrong publication type (eg reviews, opinions, conference proceedings)
454	RefID: 454, User-Centered Design for Developing Interventions to Improve Clinician Recommendation of Human Papillomavirus Vaccination<br>Henninger, M. L., McMullen, C. K., Firemark, A. J., Naleway, A. L., Henrikson, N. B., Turcotte, J. A.<br>Level: 1, State: Excluded	Not about HPV vaccination/vax attitudes (eg HPV infection/serology/prevalence; cervical cancer; HPV vax safety)
455	RefID: 455, Uptake of HPV Vaccine among young adults with disabilities, 2011 to 2018<br>Herbert, C., Curtin, C., Epstein, M., Wang, B., Lapane, K.<br>Level: 1, State: Excluded	Not about HPV vaccination/vax attitudes (eg HPV infection/serology/prevalence; cervical cancer; HPV vax safety)

456	RefID: 456, 'MALEFICENCE VERSUS BENEFICENCE': YOUNG ADULTS' PERSPECTIVES OF BEING CHILD SIMULATED PATIENTS<br>Herbertson, S.<br>Level: 1, State: Excluded	Not about HPV vaccination/vax attitudes (eg HPV infection/serology/prevalence; cervical cancer; HPV vax safety)
457	RefID: 457, Prevalence of Anal HPV Infection Among HIV-Positive Men Who Have Sex With Men in India<br>Hernandez, A. L., Karthik, R., Sivasubramanian, M., Raghavendran, A., Gnanamony, M., Lensing, S., Lee, J. Y., Kannangai, R., Abraham, P., Mathai, D., Palefsky, J. M.<br>Level: 1, State: Excluded	Age - Young adults 18-26yrs not included /outside age range
458	RefID: 458, (Mis)perceptions of HIV and HPV among female college students: A qualitative study<br>Hernandez, R.<br>Level: 2, State: Excluded	Multiple Wrong/no population (eg parents, providers, children, policy makers, does not include 18-26yrs olds; social media posts)s - unable to extract data specific to young adults (18-26 yrs)
459	RefID: 459, Comparison of adaptive and innate immune responses induced by licensed vaccines for Human Papillomavirus<br>Herrin, D. M., Coates, E. E., Costner, P. J., Kemp, T. J., Nason, M. C., Saharia, K. K., Pan, Y., Sarwar, U. N., Holman, L., Yamshchikov, G., et al.<br>Level: 1, State: Excluded	Not about HPV vaccination/vax attitudes (eg HPV infection/serology/prevalence; cervical cancer; HPV vax safety)
461	RefID: 461, Caregiver acceptance of a patient navigation program to increase human papillomavirus vaccination in pediatric clinics: a qualitative program evaluation<br>Hirth, J. M., Berenson, A. B., Cofie, L. E., Matsushita, L., Kuo, Y. F., Rupp, R. E.<br>Level: 1, State: Excluded	Wrong/no population (eg parents, providers, children, policy makers, does not include 18-26yrs olds; social media posts)
462	RefID: 462, To get the shot or not: HPV vaccines and American Indian female student decision-making<br>Hodge, Felicia Schanche<br>Level: 2, State: Excluded	studytype
465	RefID: 465, High vaccine effectiveness persists for ten years after HPV16/18 vaccination among young Dutch women<br>Hoes, J., King, A. J., Berkhof, J., Melker, H. E. de<br>Level: 1, State: Excluded	Study Design - Not qualitative (methods or analysis) OR Qualitative survey data not analyzed qualitatively (only numeric stats)
466	RefID: 466, Human Papillomavirus Vaccination and Cervical Cytology Outcomes Among Urban Low-Income Minority Females<br>Hofstetter, A. M., Ompad, D. C., Stockwell, M. S., Rosenthal, S. L., Soren, K.<br>Level: 1, State: Excluded	Age - Young adults 18-26yrs not included /outside age range
467	RefID: 467, Factors impacting HPV vaccination: lessons for health care professionals<br>Hofstetter, A. M., Rosenthal, S. L.<br>Level: 1, State: Excluded	Wrong/no population (eg parents, providers, children, policy makers, does not include 18-26yrs olds; social media posts)
468	RefID: 468, A dynamic transmission model with Age - Young adults 18-26yrs not included /outside age range-dependent infectiousness and reactivation for cytomegalovirus in the United States: Potential impact of vaccination strategies on congenital infection	Not about HPV vaccination/vax attitudes (eg HPV infection/serology/prevalence; cervical cancer; HPV vax safety)

	Hogea, C., Dieussaert, I., Van Effelterre, T., Guignard, A., Mols, J.<br>Level: 1, State: Excluded	
469	RefID: 469, Designing a Pro-Equity HPV Vaccine Delivery Program for Girls Who Have Dropped Out of School: Community Perspectives From Uttar Pradesh, India<br>Holroyd, Taylor A., Yan, Shirley D., Srivastava, Vineet, Srivastava, Ashish, Wahl, Brian, Morgan, Christopher, Kumar, Somesh, Yadav, Amit K., Jennings, Mary Carol<br>Level: 2, State: Excluded	Wrong/no population (eg parents, providers, children, policy makers, does not include 18-26yrs olds; social media posts)
470	RefID: 470, The association between HPV, intraepithelial lesions and HIV-1 shedding in anogenital specimens in two contrasting Wrong/no population (eg parents, providers, children, policy makers, does not include 18-26yrs olds; social media posts)s: Senegalese women and American MSM<br>Hood, J. E., Gottlieb, G. S., Kiviat, N. B., Sow, P. S., Toure, M., Feng, Q., Hawes, S. E.<br>Level: 1, State: Excluded	Not about HPV vaccination/vax attitudes (eg HPV infection/serology/prevalence; cervical cancer; HPV vax safety)
471	RefID: 471, Effects of a narrative HPV vaccination intervention aimed at reaching college women: a randomized controlled trial<br>Hopfer, S.<br>Level: 1, State: Excluded	Not about HPV vaccination/vax attitudes (eg HPV infection/serology/prevalence; cervical cancer; HPV vax safety)
473	RefID: 473, Health Information Source Characteristics Matter: Adapting the Dissemination of an HPV Vaccine Intervention to Reach Latina and Vietnamese Women<br>Hopfer, S., Duong, H. T., Garcia, S., Tanjasiri, S. P.<br>Level: 2, State: Excluded	Not about HPV vaccination/vax attitudes (eg HPV infection/serology/prevalence; cervical cancer; HPV vax safety)
474	RefID: 474, Culturally grounded HPV vaccine decision narratives and communication channel preferences among african-american, vietnamese, and latina young adult women attending planned parenthood clinics<br>Hopfer, S., Garcia, S., Duong, H.<br>Level: 2, State: Excluded	studytype
477	RefID: 477, Adaptation and Dissemination of a National Cancer Institute HPV Vaccine Evidence-Based Cancer Control Program to the Social Media Messaging Environment<br>Hopfer, S., Phillips, K. K., Weinzierl, M., Vasquez, H. E., Alkhatib, S., Harabagiu, S. M.<br>Level: 1, State: Excluded	Wrong/no population (eg parents, providers, children, policy makers, does not include 18-26yrs olds; social media posts)
479	RefID: 479, Taking an HPV vaccine research-tested intervention to scale in a clinical setting<br>Hopfer, S., Ray, A. E., Hecht, M. L., Miller-Day, M., Belue, R., Zimet, G., Evans, W. D., McKee, F. X.<br>Level: 2, State: Excluded	Not about HPV vaccination/vax attitudes (eg HPV infection/serology/prevalence; cervical cancer; HPV vax safety)
480	RefID: 480, A novel teen community health advisor program to reduce sexual risk behaviors<br>Howard, C., Litton, A. G., Reshard, S., Schoenberger, Y. M., Kempf, M. C., Simpson, T.<br>Level: 1, State: Excluded	Not about HPV vaccination/vax attitudes (eg HPV infection/serology/prevalence; cervical cancer; HPV vax safety)

481	RefID: 481, An Observational Study of Deep Learning and Automated Evaluation of Cervical ImAge - Young adults 18-26yrs not included /outside age ranges for Cancer Screening<br>Hu, L., Bell, D., Antani, S., Xue, Z., Yu, K., Horning, M. P., Gachuhi, N., Wilson, B., Jaiswal, M. S., Befano, B., Long, L. R., Herrero, R., Einstein, M. H., Burk, R. D., Demarco, M., GAge - Young adults 18-26yrs not included /outside age range, J. C., Rodriguez, A. C., Wentzensen, N., Schiffman, M.<br>Level: 1, State: Excluded	Not about HPV vaccination/vax attitudes (eg HPV infection/serology/prevalence; cervical cancer; HPV vax safety)
482	RefID: 482, Role of human papillomavirus status after conization for high-grade cervical intraepithelial neoplasia<br>Huang, H. J., Tung, H. J., Yang, L. Y., Chao, A., Tang, Y. H., Chou, H. H., Chang, W. Y., Wu, R. C., Huang, C. C., Lin, C. Y., Liao, M. J., Chen, W. C., Lin, C. T., Chen, M. Y., Huang, K. G., Wang, C. J., Chang, T. C., Lai, C. H.<br>Level: 1, State: Excluded	Not about HPV vaccination/vax attitudes (eg HPV infection/serology/prevalence; cervical cancer; HPV vax safety)
483	RefID: 483, Designing and Implementing an Educational Social Media Campaign to Increase HPV Vaccine Awareness among Men on a Large College Campus<br>Hughes, Connor T., Kirtz, Susan, Ramondetta, Lois M., Lu, Qian, Cho, Dalnim, Katzin, Charlotte, Kahlor, Lee Ann<br>Level: 1, State: Excluded	Not about HPV vaccination/vax attitudes (eg HPV infection/serology/prevalence; cervical cancer; HPV vax safety)
484	RefID: 484, Knowledge towards human papilloma virus (HPV) infection and attitude towards its vaccine in the Kingdom of Bahrain: cross-sectional study<br>Husain, Y., Alalwan, A., Al-Musawi, Z., Abdulla, G., Hasan, K., Jassim, G.<br>Level: 1, State: Excluded	Age - Young adults 18-26yrs not included /outside age range
485	RefID: 485, Attitudes and perceptions towards HPV vaccination among young women in Saudi Arabia<br>Hussain, A. N., Abdullah, Alkhenizan, McWalter, P., Nusrat, Qazi, Amal, Alshmassi, Samina, Farooqi, Ahmed, Abdulkarim<br>Level: 1, State: Excluded	Study Design - Not qualitative (methods or analysis) OR Qualitative survey data not analyzed qualitatively (only numeric stats)
486	RefID: 486, Perception of human papillomavirus infection, cervical cancer and HPV vaccination in North Indian Wrong/no population (eg parents, providers, children, policy makers, does not include 18-26yrs olds; social media posts)<br>Hussain, S., Nasare, V., Kumari, M., Sharma, S., Khan, M. A., Das, B. C., Bharadwaj, M.<br>Level: 2, State: Excluded	Study Design - Not qualitative (methods or analysis) OR Qualitative survey data not analyzed qualitatively (only numeric stats)
487	RefID: 487, Human papillomavirus infection, vaccination, and cervical cancer communication: the protection dilemma faced by women in southern Appalachia<br>Hutson, S. P., Dorgan, K. A., Duvall, K. L., Garrett, L. H.<br>Level: 2, State: Excluded	Multiple Wrong/no population (eg parents, providers, children, policy makers, does not include 18-26yrs olds; social media posts)s - unable to extract data specific to young adults (18-26 yrs)
488	RefID: 488, Association Between Human Papillomavirus Vaccination and Primary Ovarian Insufficiency in a Nationwide Cohort<br>Hviid, A., Myrup Thiesson, E.<br>Level: 1, State: Excluded	Not about HPV vaccination/vax attitudes (eg HPV infection/serology/prevalence; cervical cancer; HPV vax safety)

489	RefID: 489, Review of cervical carcinoma screening program in Tamil Nadu, the current trend and recommendations from a histopathologist's viewpoint<br>Ibrahim, S. S., Kesavaraj, K., Arun, M., Ameen, S. A. M., Sankar, R.<br>Level: 1, State: Excluded	Wrong publication type (eg reviews, opinions, conference proceedings)
490	RefID: 490, Prevalence of low-risk and high-risk types of human papillomavirus and other risk factors for HPV infection in Germany within different Age - Young adults 18-26yrs not included /outside age range groups in women up to 30 years of Age - Young adults 18-26yrs not included /outside age range: an epidemiological observational study<br>Iftner, T., Eberle, S., Iftner, A., Holz, B., Banik, N., Quint, W., Straube, A. N.<br>Level: 1, State: Excluded	Not about HPV vaccination/vax attitudes (eg HPV infection/serology/prevalence; cervical cancer; HPV vax safety)
491	RefID: 491, Prevalence and Risk Factors of Human Papillomavirus Infection in 18-Year-Old Women: Baseline Report of a Prospective Study on Human Papillomavirus Vaccine<br>Igidbashian, S., Boveri, S., Bottari, F., Vidal Urbinati, A., Preti, E., Casadio, C., Landoni, F., Sideri, M., Sandri, M. T.<br>Level: 1, State: Excluded	Study Design - Not qualitative (methods or analysis) OR Qualitative survey data not analyzed qualitatively (only numeric stats)
492	RefID: 492, Factors influencing Human papillomavirus (HPV) vaccination series completion in Mississippi Medicaid<br>Inguva, S., Barnard, M., Ward, L. M., Yang, Y., Pittman, E., Banahan, B. F., Kirby, T. R., Noble, S. L.<br>Level: 1, State: Excluded	Study Design - Not qualitative (methods or analysis) OR Qualitative survey data not analyzed qualitatively (only numeric stats)
493	RefID: 493, Changes in human papillomavirus genotypes associated with cervical intraepithelial neoplasia grade 2 lesions in a cohort of young women (2013-2016)<br>Innes, C. R., Sykes, P. H., Harker, D., Williman, J. A., Van der Griend, R. A., Whitehead, M., Hibma, M., Lawton, B. A., Fitzgerald, P., Dudley, N. M., Petrich, S., Faherty, J., Bergzoll, C., Eva, L., Sadler, L., Simcock, B. J.<br>Level: 1, State: Excluded	Study Design - Not qualitative (methods or analysis) OR Qualitative survey data not analyzed qualitatively (only numeric stats)
495	RefID: 495, Impact of human papillomavirus vaccination on rates of abnormal cervical cytology and histology in young New Zealand women<br>Innes, C. R. H., Williman, J. A., Simcock, B. J., Hider, P., SAge - Young adults 18-26yrs not included /outside age range, M., Dempster-Rivett, K., Lawton, B. A., Sykes, P. H.<br>Level: 1, State: Excluded	Not about HPV vaccination/vax attitudes (eg HPV infection/serology/prevalence; cervical cancer; HPV vax safety)
497	RefID: 497, Knowledge of cervical cancer and HPV vaccine in Bangladeshi women: a Wrong/no population (eg parents, providers, children, policy makers, does not include 18-26yrs olds; social media posts) based, cross-sectional study<br>Islam, J. Y., Fatema, Khatun, Anadil, Alam, Sultana, F., Bhuiyan, A., Alam, N., Reichenbach, L., Marions, L., Mustafizur, Rahman, Quamrun, Nahar<br>Level: 1, State: Excluded	Age - Young adults 18-26yrs not included /outside age range
499	RefID: 499, A mobile, web-based storytelling HPV intervention to promote HPV vaccine uptake among Korean college women<br>Isrctn<br>Level: 1, State: Excluded	Study Design - Not qualitative (methods or analysis) OR Qualitative survey data not analyzed qualitatively (only numeric stats)

500	RefID: 500, UNderstanding uptake of Immunisations in TravellIng aNd Gypsy communities (UNITING): a qualitative interview study<br>Jackson, C., Dyson, L., Bedford, H., Cheater, F. M., Condon, L., Crocker, A., Emslie, C., Ireland, L., Kemsley, P., Kerr, S., Lewis, H. J., Mytton, J., Overend, K., Redsell, S., Richardson, Z., Shepherd, C., Smith, L.<br>Level: 2, State: Excluded	Multiple Wrong/no population (eg parents, providers, children, policy makers, does not include 18-26yrs olds; social media posts)s - unable to extract data specific to young adults (18-26 yrs)
501	RefID: 501, Sexual orientation identity disparities in health behaviors, outcomes, and services use among men and women in the United States: a cross-sectional study<br>Jackson, Chandra L., Agénor, Madina, Johnson, Dayna A., Austin, Bryn, Ichiro, Kawachi, Austin, S. Bryn, Kawachi, Ichiro<br>Level: 1, State: Excluded	Not about HPV vaccination/vax attitudes (eg HPV infection/serology/prevalence; cervical cancer; HPV vax safety)
503	RefID: 503, An unmasking phenomenon in an observational post-licensure safety study of adolescent girls and young women<br>Jacobsen, S. J., Sy, L. S., Ackerson, B. K., Chao, C. R., Slezak, J. M., Cheetham, T. C., Takhar, H. S., Velicer, C. M., Hansen, J., Klein, N. P.<br>Level: 1, State: Excluded	Study Design - Not qualitative (methods or analysis) OR Qualitative survey data not analyzed qualitatively (only numeric stats)
504	RefID: 504, Immunogenicity and tolerability to human papillomavirus-like particle vaccine in girls and young women with inflammatory bowel disease<br>Jacobson, D. L., Bousvaros, A., Ashworth, L., Carey, R., Shrier, L. A., Burchett, S. K., Renna, H., Lu, Y.<br>Level: 1, State: Excluded	Age - Young adults 18-26yrs not included /outside age range
505	RefID: 505, Impact of HPV vaccination with Gardasil® in Switzerland<br>Jacot-Guillarmod, M., Pasquier, J., Greub, G., Bongiovanni, M., Achtari, C., Sahli, R.<br>Level: 1, State: Excluded	Not about HPV vaccination/vax attitudes (eg HPV infection/serology/prevalence; cervical cancer; HPV vax safety)
506	RefID: 506, Risk Reduction Interventions for Human Papillomavirus in Rural Maryland<br>Jafari, S. D. G., Appel, S. J., Shorter, D. G.<br>Level: 1, State: Excluded	Age - Young adults 18-26yrs not included /outside age range
507	RefID: 507, Public-private knowledge transfer and access to medicines: a systematic review and qualitative study of perceptions and roles of scientists involved in HPV vaccine research<br>Jahn, R., Müller, O., Nöst, S., Bozorgmehr, K.<br>Level: 1, State: Excluded	Wrong publication type (eg reviews, opinions, conference proceedings)
508	RefID: 508, Human papillomavirus (HPV) awareness and vaccination initiation among women in the United States, National Immunization Survey-Adult 2007<br>Jain, N., Euler, G. L., Shefer, A., Lu, P., Yankey, D., Markowitz, L.<br>Level: 1, State: Excluded	Not about HPV vaccination/vax attitudes (eg HPV infection/serology/prevalence; cervical cancer; HPV vax safety)
510	RefID: 510, Parental acceptance of human papillomavirus (HPV) vaccination in Indonesia: a cross-sectional study<br>Jaspers, L., Budiningsih, S., Wolterbeek, R., Henderson, F. C., Peters, A. A.<br>Level: 1, State: Excluded	
512	RefID: 512, Risk for cervical intraepithelial neoplasia grade 3 or worse in relation to smoking among women with persistent human papillomavirus infection	Wrong/no population (eg parents, providers, children, policy makers,

	Jensen, K. E., Schmiedel, S., Frederiksen, K., Norrild, B., Iftner, T., Kjær, S. K.<br>Level: 1, State: Excluded	does not include 18-26yrs olds; social media posts)
513	RefID: 513, A community intervention effectiveness study of single dose or two doses of bivalent HPV vaccine (CERVARIX®) in female school students in Thailand<br>Jiamsiri, S., Rhee, C., Ahn, H. S., Poudyal, N., Seo, H. W., Klinsupa, W., Nilyanimit, P., Premsri, N., Namwat, C., Vonpunsawad, S., Chon, Y., Park, S., Kim, D. R., Unger, E. R., Markowitz, L., Poovorawan, Y., Rerks-Ngarm, S., Excler, J. L., Lynch, J.<br>Level: 1, State: Excluded	Not about HPV vaccination/vax attitudes (eg HPV infection/serology/prevalence; cervical cancer; HPV vax safety)
514	RefID: 514, CDC National Health Report: leading causes of morbidity and mortality and associated behavioral risk and protective factors--United States, 2005-2013<br>Johnson, N. B., Hayes, L. D., Brown, K., Hoo, E. C., Ethier, K. A.<br>Level: 1, State: Excluded	Not about HPV vaccination/vax attitudes (eg HPV infection/serology/prevalence; cervical cancer; HPV vax safety)
515	RefID: 515, Using branded behaviour change communication to create demand for the HPV vaccine among girls in Malawi: An evaluation of Girl Effect's Zathu mini magazine<br>Jones, A., Kawesa-Newell, N.<br>Level: 1, State: Excluded	Not about HPV vaccination/vax attitudes (eg HPV infection/serology/prevalence; cervical cancer; HPV vax safety)
517	RefID: 517, Knowledge, attitudes, and beliefs regarding HPV vaccination: ethnic and cultural differences between African-American and Haitian immigrant women<br>Joseph, N. P., Clark, J. A., Bauchner, H., Walsh, J. P., Mercilus, G., Figaro, J., Bibbo, C., Perkins, R. B.<br>Level: 1, State: Excluded	Wrong/no population (eg parents, providers, children, policy makers, does not include 18-26yrs olds; social media posts)
519	RefID: 519, Attribution of 12 high-risk human papillomavirus genotypes to infection and cervical disease<br>Joura, E. A., Ault, K. A., Bosch, F. X., Brown, D., Cuzick, J., Ferris, D., Garland, S. M., Giuliano, A. R., Hernandez-Avila, M., Huh, W., et al.<br>Level: 1, State: Excluded	Study Design - Not qualitative (methods or analysis) OR Qualitative survey data not analyzed qualitatively (only numeric stats)
520	RefID: 520, Knowledge, attitudes, and practices among Saudi women regarding cervical cancer, human papillomavirus (HPV) and corresponding vaccine<br>Jradi, H., Bawazir, A.<br>Level: 2, State: Excluded	Not about HPV vaccination/vax attitudes (eg HPV infection/serology/prevalence; cervical cancer; HPV vax safety)
521	RefID: 521, Foundation scholar studies HPV vaccine uptake in college women<br>Judge, Kate<br>Level: 1, State: Excluded	Multiple Wrong/no population (eg parents, providers, children, policy makers, does not include 18-26yrs olds; social media posts)s - unable to extract data specific to young adults (18-26 yrs)
523	RefID: 523, Potential impact of the human papillomavirus vaccine on the incidence proportion of genital warts in French women (EFFICAE study): a multicentric prospective observational study<br>Judlin, P., Jacquard, A. C., Carcopino, X., Aubin, F., Dahlab, A., Mistretta, F., Not, D., Boelle, P. Y., Aynaud, O., Soubeyrand, B.<br>Level: 1, State: Excluded	Wrong publication type (eg reviews, opinions, conference proceedings)

524	RefID: 524, HPV vaccine promotion: does referring to both cervical cancer and genital warts affect intended and actual vaccination behavior?<br>Juraskova, I., Bari, R. A., O'Brien, M. T., McCaffery, K. J.<br>Level: 1, State: Excluded	Not about HPV vaccination/vax attitudes (eg HPV infection/serology/prevalence; cervical cancer; HPV vax safety)
525	RefID: 525, Provider-Level Barriers to Human Papillomavirus Vaccination in Survivors of Childhood and Young Adult Cancers<br>Kacew, A. J., Jacobson, S., Sheade, J., Patel, A. A., Hlubocky, F. J., Lee, N. K., Henderson, T. O., Schneider, J. A., Strohbehn, G. W.<br>Level: 1, State: Excluded	Study Design - Not qualitative (methods or analysis) OR Qualitative survey data not analyzed qualitatively (only numeric stats)
526	RefID: 526, Behavioral, immunologic, and virologic correlates of oral human papillomavirus infection in HIV-infected youth<br>Kahn, J. A., Rudy, B. J., Xu, J., Secord, E. A., Kapogiannis, B. G., Thornton, S., Gillison, M. L.<br>Level: 1, State: Excluded	Wrong/no population (eg parents, providers, children, policy makers, does not include 18-26yrs olds; social media posts)
527	RefID: 527, Level of Awareness About HPV Infection and Vaccine Among the Medical Students: A Comprehensive Review from India<br>Kamath, A., Yadav, A., Baghel, J., Bansal, P., Mundle, S.<br>Level: 1, State: Excluded	Study Design - Not qualitative (methods or analysis) OR Qualitative survey data not analyzed qualitatively (only numeric stats)
528	RefID: 528, Assessment of knowledge about cervical cancer and its prevention among female students Age - Young adults 18-26yrs not included /outside age ranged 17-26 years<br>Kamzol, W., Jaglarz, K., Tomaszewski, K. A., Puskulluoglu, M., Krzemieniecki, K.<br>Level: 1, State: Excluded	Study Design - Not qualitative (methods or analysis) OR Qualitative survey data not analyzed qualitatively (only numeric stats)
529	RefID: 529, Human papilloma virus vaccination: Perceptions of young Korean women<br>Kang, Hee Sun, Shin, Hyunsook, Hyun, Myung-Sun, Kim, Mi Ja<br>Level: 2, State: Excluded	Study Design - Not qualitative (methods or analysis) OR Qualitative survey data not analyzed qualitatively (only numeric stats)
531	RefID: 531, Greek health professionals' perceptions of the HPV vaccine, state policy recommendations and their own role with regards to communication of relevant health information<br>Karamanidou, C., Dimopoulos, K.<br>Level: 1, State: Excluded	Multiple Wrong/no population (eg parents, providers, children, policy makers, does not include 18-26yrs olds; social media posts)s - unable to extract data specific to young adults (18-26 yrs)
532	RefID: 532, Human Papillomavirus Vaccination Prevalence Among Adults Age - Young adults 18-26yrs not included /outside age ranged 19-45 Years: An Analysis of the 2017 National Health Interview Survey<br>Kasting, M. L., Giuliano, A. R., Christy, S. M., Rouse, C. E., Robertson, S. E., Thompson, E. L.<br>Level: 1, State: Excluded	Wrong/no population (eg parents, providers, children, policy makers, does not include 18-26yrs olds; social media posts)
534	RefID: 534, A qualitative analysis of factors influencing HPV vaccine uptake in Soweto, South Africa among adolescents and their caregivers<br>Katz, I. T., Nkala, B., Dietrich, J., Wallace, M., Bekker, L. G., Pollenz, K., Bogart, L. M., Wright, A. A., Tsai, A. C., Bangsberg, D. R., Gray, G. E.<br>Level: 2, State: Excluded	Not about HPV vaccination/vax attitudes (eg HPV infection/serology/prevalence; cervical cancer; HPV vax safety)

535	RefID: 535, Acceptance of the HPV vaccine among women, parents, community leaders, and healthcare providers in Ohio Appalachia<br>Katz, M. L., Reiter, P. L., Heaner, S., Ruffin, M. T., Post, D. M., Paskett, E. D.<br>Level: 2, State: Excluded	Multiple Wrong/no population (eg parents, providers, children, policy makers, does not include 18-26yrs olds; social media posts)s - unable to extract data specific to young adults (18-26 yrs)
536	RefID: 536, Cervical cancer screening in developing regions: Observations from Paraiso, an underserved community in the Dominican republic<br>Kaumaya, M., Guraker, M., Dinnel, J., Ryan, M., Pryor, R., Bearman, G., Stevens, M.<br>Level: 1, State: Excluded	Multiple Wrong/no population (eg parents, providers, children, policy makers, does not include 18-26yrs olds; social media posts)s - unable to extract data specific to young adults (18-26 yrs)
537	RefID: 537, LETTER TO THE EDITOR<br>Keegan, Sarah Maeve<br>Level: 1, State: Excluded	Age - Young adults 18-26yrs not included /outside age range
538	RefID: 538, Health communication messaging about HPV vaccine in Papua New Guinea<br>Kelly-Hanku, A., Newland, J., Aggleton, P., Ase, S., Fiya, V., Aeno, H., Vallely, L. M., Mola, G. D. L., Kaldor, J. M., Vallely, A. J.<br>Level: 2, State: Excluded	Wrong publication type (eg reviews, opinions, conference proceedings)
539	RefID: 539, Knowledge of human papillomavirus among publicly and privately insured women<br>Kennedy, S., Osgood, R., Rosenbloom, L., Feinglass, J., Simon, M.<br>Level: 1, State: Excluded	Not about HPV vaccination/vax attitudes (eg HPV infection/serology/prevalence; cervical cancer; HPV vax safety)
540	RefID: 540, Is provider type associated with cancer screening and prevention: Advanced practice registered nurses, physician assistants, and physicians<br>Kepka, D., Smith, A., Zeruto, C., Yabroff, K. R.<br>Level: 1, State: Excluded	Study Design - Not qualitative (methods or analysis) OR Qualitative survey data not analyzed qualitatively (only numeric stats)
541	RefID: 541, Low Human Papillomavirus (HPV) Vaccine Knowledge Among Latino Parents in Utah<br>Kepka, Deanna, Warner, Echo, Kinney, Anita, Spigarelli, Michael, Mooney, Kathi<br>Level: 1, State: Excluded	Not about HPV vaccination/vax attitudes (eg HPV infection/serology/prevalence; cervical cancer; HPV vax safety)
542	RefID: 542, Mixed-methods study in England and Northern Ireland to understand young men who have sex with men's knowledge and attitudes towards human papillomavirus vaccination<br>Kesten, J. M., Flannagan, C., Ruane-McAteer, E., Merriel, S. W. D., Nadarzynski, T., Shapiro, G., Rosberger, Z., Prue, G.<br>Level: 2, State: Excluded	Wrong/no population (eg parents, providers, children, policy makers, does not include 18-26yrs olds; social media posts)
543	RefID: 543, Is category B working? Uptake patterns of meningococcal group B vaccine among US adolescents and young adults<br>Khan, F. L., Swerdlow, D. L., York, L. J., Balmer, P., Isturiz, R. E., McLaughlin, J. M.<br>Level: 1, State: Excluded	Multiple Wrong/no population (eg parents, providers, children, policy makers, does not include 18-26yrs olds; social media posts)s - unable to extract data specific to young adults (18-26 yrs)
544	RefID: 544, Receipt of human papillomavirus vaccine among privately insured adult women in a U.S. Midwestern Health Maintenance Organization	Not about HPV vaccination/vax attitudes (eg HPV

	Kharbanda, E. O., Parker, E., Nordin, J. D., Hedblom, B., Rolnick, S. J.<br>Level: 1, State: Excluded	infection/serology/prevalence; cervical cancer; HPV vax safety)
545	RefID: 545, Risk of spontaneous abortion after inadvertent human papillomavirus vaccination in pregnancy<br>Kharbanda, E. O., Vazquez-Benitez, G., Lipkind, H. S., Sheth, S. S., Zhu, JingYi, Naleway, A. L., Klein, N. P., Hechter, R., Daley, M. F., Donahue, J. G., Jackson, M. L., Kawai, A. T., Lakshmi, Sukumaran, Nordin, J. D.<br>Level: 1, State: Excluded	Study Design - Not qualitative (methods or analysis) OR Qualitative survey data not analyzed qualitatively (only numeric stats)
547	RefID: 547, Awareness of cervical cancer and its prevention among women attending a tertiary-care hospital in northern Delhi, India<br>Khatuja, R., Renjhen, P., Prasad, S., Kadiyan, M., Attuluri, L.<br>Level: 1, State: Excluded	Not about HPV vaccination/vax attitudes (eg HPV infection/serology/prevalence; cervical cancer; HPV vax safety)
548	RefID: 548, Awareness of cervical cancer and HPV vaccination and its affordability among rural folks in Penang Malaysia<br>Khoo, C. L., Teoh, S., Rashid, A. K., Zakaria, U. U., Mansor, S., Salleh, F. N., Nawi, M. N.<br>Level: 1, State: Excluded	Not about HPV vaccination/vax attitudes (eg HPV infection/serology/prevalence; cervical cancer; HPV vax safety)
549	RefID: 549, The human papillomavirus vaccine: A systematic review elucidating the dynamics of ethical concerns and public health policies<br>Kiekenbush, C.<br>Level: 1, State: Excluded	Study Design - Not qualitative (methods or analysis) OR Qualitative survey data not analyzed qualitatively (only numeric stats)
550	RefID: 550, Vaccine hesitancy and HPV vaccine uptake among male and female youth in Switzerland: a cross-sectional study<br>Kiener, L. M., Schwendener, C. L., Jafflin, K., Meier, A., Reber, N., Schärli Maurer, S., Muggli, F., Gültekin, N., Huber, B. M., Merten, S., Deml, M. J., Tarr, P. E.<br>Level: 1, State: Excluded	Wrong publication type (eg reviews, opinions, conference proceedings)
551	RefID: 551, Six-year multi-centre, observational, post-marketing surveillance of the safety of the HPV-16/18 AS04-adjuvanted vaccine in women Age - Young adults 18-26yrs not included /outside age ranged 10-25 years in Korea<br>Kim, C. J., Song, R., Chen, J., Tavares Da Silva, F., Gopala, K. B., Kim, J. H., Bi, D., Park, J. S.<br>Level: 1, State: Excluded	Study Design - Not qualitative (methods or analysis) OR Qualitative survey data not analyzed qualitatively (only numeric stats)
553	RefID: 553, The Interplay between Framing and Regulatory Focus in Processing Narratives about HPV Vaccination in Singapore<br>Kim, H. K., Lee, T. K., Kong, W. Y.<br>Level: 1, State: Excluded	Age - Young adults 18-26yrs not included /outside age range
554	RefID: 554, The Impact of Narrative Strategy on Promoting HPV Vaccination among College Students in Korea: The Role of Anticipated Regret<br>Kim, J.<br>Level: 1, State: Excluded	Study Design - Not qualitative (methods or analysis) OR Qualitative survey data not analyzed qualitatively (only numeric stats)
555	RefID: 555, Temporal Framing Effects Differ for Narrative Versus Non-Narrative MessAge - Young adults 18-26yrs not included /outside age ranges: The Case of Promoting HPV Vaccination<br>Kim, J., Nan, X.<br>Level: 1, State: Excluded	Study Design - Not qualitative (methods or analysis) OR Qualitative survey data not analyzed qualitatively (only numeric stats)

556	RefID: 556, Knowledge, perceptions, and decision making about human papillomavirus vaccination among Korean American women: A focus group study<br>Kim, Kyounghae, Kim, Boyoung, Choi, Eunsuk, Song, Youngshin, Han, Hae-Ra<br>Level: 2, State: Excluded	Study Design - Not qualitative (methods or analysis) OR Qualitative survey data not analyzed qualitatively (only numeric stats)
558	RefID: 558, Development and acceptability of a peer-paired, cross-cultural and cross-generational storytelling HPV intervention for Korean American college women<br>Kim, Minjin, Lee, Haeok, Kiang, Peter, Allison, Jeroan<br>Level: 2, State: Excluded	Wrong/no population (eg parents, providers, children, policy makers, does not include 18-26yrs olds; social media posts)
561	RefID: 561, Assessing the epidemiological impact on cervical cancer of switching from 4-valent to 9-valent HPV vaccine within a gender-neutral vaccination programme in Switzerland<br>Kind, A. B., Pavelyev, A., Kothari, S., El Mouaddin, N., Schmidt, A., Morais, E., Guggisberg, P., Lienert, F.<br>Level: 1, State: Excluded	Not about HPV vaccination/vax attitudes (eg HPV infection/serology/prevalence; cervical cancer; HPV vax safety)
562	RefID: 562, Human papillomavirus DNA in men who have sex with men: type-specific prevalence, risk factors and implications for vaccination strategies<br>King, E. M., Gilson, R., Beddows, S., Soldan, K., Panwar, K., Young, C., Prah, P., Jit, M., Edmunds, W. J., Sonnenberg, P.<br>Level: 1, State: Excluded	Not about HPV vaccination/vax attitudes (eg HPV infection/serology/prevalence; cervical cancer; HPV vax safety)
563	RefID: 563, Human papillomavirus vaccination rates in young cancer survivors<br>Klosky, J. L., Hudson, M. M., Chen, YanJun, Connelly, J. A., Wasilewski-Masker, K., Sun, CanLan, Francisco, L., Gustafson, L., Russell, K. M., Sabbatini, G., Flynn, J. S., York, J. M., Giuliano, A. R., Robison, L. L., Wong, F. L., Smita, Bhatia, Landier, W.<br>Level: 1, State: Excluded	Not about HPV vaccination/vax attitudes (eg HPV infection/serology/prevalence; cervical cancer; HPV vax safety)
566	RefID: 566, Knowledge of HPV among United States Hispanic women: opportunities and challenges for cancer prevention<br>Kobetz, E., Kornfeld, J., Vanderpool, R. C., Finney Rutten, L. J., Parekh, N., O'Bryan, G., Menard, J.<br>Level: 1, State: Excluded	Age - Young adults 18-26yrs not included /outside age range
568	RefID: 568, Perceptions of HPV and cervical cancer among Haitian immigrant women: implications for vaccine acceptability<br>Kobetz, E., Menard, J., Hazan, G., Koru-Sengul, T., Joseph, T., Nissan, J., Barton, B., Blanco, J., Kornfeld, J.<br>Level: 2, State: Excluded	Study Design - Not qualitative (methods or analysis) OR Qualitative survey data not analyzed qualitatively (only numeric stats)
569	RefID: 569, European code against cancer (ECAC): Actions taken by polish ecac youth ambassador<br>Koczkodaj, P.<br>Level: 1, State: Excluded	Multiple Wrong/no population (eg parents, providers, children, policy makers, does not include 18-26yrs olds; social media posts)s - unable to extract data specific to young adults (18-26 yrs)
570	RefID: 570, Impact of Parental Knowledge and Beliefs on HPV Vaccine Hesitancy in Kenya—Findings and Implications<br>Kolek, C. O., Opanga, S. A., Okalebo, F., Birichi, A., Kurdi, A., Godman, B.,	Not about HPV vaccination/vax attitudes (eg HPV infection/serology/prevalence; cervical cancer; HPV vax safety)

	Meyer, J. C.<br>Level: 1, State: Excluded	
571	RefID: 571, High-risk human papillomavirus infection in HIV-positive African women living in Europe<br>Konopnicki, D., Manigart, Y., Gilles, C., Barlow, P., de Marchin, J., Feoli, F., Larsimont, D., Delforge, M., De Wit, S., Clumeck, N.<br>Level: 1, State: Excluded	Wrong/no population (eg parents, providers, children, policy makers, does not include 18-26yrs olds; social media posts)
572	RefID: 572, National prevalence of oral HPV infection in vaccinated and unvaccinated young adults in Brazil<br>Kops, N. L., Comerlato, J., Bandeira, I., Bessel, M., Maranhão, A. G. K., Villa, L. L., Mota, G., De Souza, F. M. A., Pereira, G. F. M., Wendland, E.<br>Level: 1, State: Excluded	Age - Young adults 18-26yrs not included /outside age range
573	RefID: 573, Knowledge about HPV and vaccination among young adult men and women: Results of a national survey<br>Kops, N. L., Hohenberger, G. F., Bessel, M., Correia Horvath, J. D., Domingues, C., Kalume Maranhão, A. G., Alves de Souza, F. M., Benzaken, A., Pereira, G. F., Wendland, E. M.<br>Level: 1, State: Excluded	Age - Young adults 18-26yrs not included /outside age range
574	RefID: 574, The impact of socioeconomic status on HPV infection among young Brazilians in a nationwide multicenter study<br>Kops, N. L., Horvath, J. D. C., Bessel, M., Souza, F. M. A., Benzaken, A. S., Pereira, G. F. M., Villa, L. L., Wendland, E. M.<br>Level: 1, State: Excluded	Study Design - Not qualitative (methods or analysis) OR Qualitative survey data not analyzed qualitatively (only numeric stats)
575	RefID: 575, Sources of the uncertainty experienced by women with HPV<br>Kosenko, K. A., Hurley, R. J., Harvey, J. A.<br>Level: 2, State: Excluded	Not about HPV vaccination/vax attitudes (eg HPV infection/serology/prevalence; cervical cancer; HPV vax safety)
576	RefID: 576, College Students' Digital Media Preferences for future HPV Vaccine Campaigns<br>Koskan, A., Cantley, A., Li, R., Silvestro, K., Helitzer, D.<br>Level: 2, State: Excluded	Not about HPV vaccination/vax attitudes (eg HPV infection/serology/prevalence; cervical cancer; HPV vax safety)
578	RefID: 578, Anal Cancer Prevention Perspectives Among Foreign-Born Latino HIV-Infected Gay and Bisexual Men<br>Koskan, A. M., Fernandez-Pineda, M.<br>Level: 1, State: Excluded	Not about HPV vaccination/vax attitudes (eg HPV infection/serology/prevalence; cervical cancer; HPV vax safety)
579	RefID: 579, Human papillomavirus-specific antibody status among unvaccinated subjects in the region of Vojvodina, Serbia<br>Kovačević, G., Božić Nedeljković, B., Patić, A., Radovanov, J., Hrnjaković-Cvjetković, I.<br>Level: 1, State: Excluded	Not about HPV vaccination/vax attitudes (eg HPV infection/serology/prevalence; cervical cancer; HPV vax safety)
580	RefID: 580, High Prevalence of Anal Oncogenic Human Papillomavirus Infection in Young Men Who Have Sex with Men Living in Bamako, Mali<br>Koyalta, Donato, Mboumba Bouassa, Ralph-Sydney, Maiga, Almoustapha, Balde, Aliou, BAge - Young adults 18-26yrs not included /outside age rangendabanga, Jules Bashi, Alinity, Almahdy Ag, Veyer, David, Péré, Hélène, Bélec, Laurent<br>Level: 1, State: Excluded	Not about HPV vaccination/vax attitudes (eg HPV infection/serology/prevalence; cervical cancer; HPV vax safety)
581	RefID: 581, Collateral DamAge - Young adults 18-26yrs not included /outside age range and Critical Turning Points: Public Health	Age - Young adults 18-26yrs not included /outside age range

	Implications of HPV Vaccine News CoverAge - Young adults 18-26yrs not included /outside age range for Boys and Men in 2011<br>Krakow, M., Rogers, B.<br>Level: 1, State: Excluded	
582	RefID: 582, Death narratives and cervical cancer: Impact of character death on narrative processing and HPV vaccination<br>Krakow, M., Yale, R. N., Perez Torres, D., Christy, K., Jensen, J. D.<br>Level: 2, State: Excluded	Wrong publication type (eg reviews, opinions, conference proceedings)
584	RefID: 584, Telling stories for cervical cancer prevention: The impact of narrative features and processes on young women's HPV vaccination intentions<br>Krakow, Melinda Michele<br>Level: 2, State: Excluded	Not about HPV vaccination/vax attitudes (eg HPV infection/serology/prevalence; cervical cancer; HPV vax safety)
585	RefID: 585, What do they know and what do they think they know? The influence of knowledge and perceived knowledge on human papillomavirus (HPV) vaccination decision-making<br>Krawczyk, A., Lau, E., Perez, S., Rosberger, Z.<br>Level: 1, State: Excluded	studytype
586	RefID: 586, Evaluation of Durability of a Single Dose of the Bivalent HPV Vaccine: The CVT Trial<br>Kreimer, Aimée R., Sampson, Joshua N., Porras, Carolina, Schiller, John T., Kemp, Troy, Herrero, Rolando, Wagner, Sarah, Boland, Joseph, Schussler, John, Lowy, Douglas R., Chanock, Stephen, Roberson, David, Sierra, Mónica S., Tsang, Sabrina H., Schiffman, Mark, Rodriguez, Ana Cecilia, Cortes, Bernal, Gail, Mitchell H., Hildesheim, Allan, Gonzalez, Paula<br>Level: 1, State: Excluded	Study Design - Not qualitative (methods or analysis) OR Qualitative survey data not analyzed qualitatively (only numeric stats)
590	RefID: 590, Risky sexual behaviour and contraceptive use among young women in the Czech Republic<br>Křepelka, P., Fait, T., Urbánková, I., Hanáček, J., Krofta, L., Dvořák, V.<br>Level: 1, State: Excluded	Not about HPV vaccination/vax attitudes (eg HPV infection/serology/prevalence; cervical cancer; HPV vax safety)
591	RefID: 591, Beliefs and acceptance of human papillomavirus (HPV) vaccine among parents in urban community in yogyakarta<br>Kristina, S. A., Lienaningrum, A. S., Wulandari, G. P.<br>Level: 1, State: Excluded	Study Design - Not qualitative (methods or analysis) OR Qualitative survey data not analyzed qualitatively (only numeric stats)
592	RefID: 592, Systematic review: Does a positive human papillomavirus vaccination status increase the risk of unsafe sexual health practice in Australian women?<br>Kuk, N., To, J., McBride, C., Hong, A., Ng, E., Li, N., Teo, M., Zhang, V., Velasco, D., Ling, L., Sun, Y., Keem, M.<br>Level: 1, State: Excluded	Wrong/no population (eg parents, providers, children, policy makers, does not include 18-26yrs olds; social media posts)
593	RefID: 593, On Sekiz Yaş Üzeri Kadınların Genital Verru, Servikal Kanser ve Human Papilloma Virüs Aşısı Hakkında Bilgi, Tutum ve Davranışları<br>Kurtipek, Gülcan Saylam, Cihan, Fatma Gökşin, Ataseven, Arzu, Özer, İlkay, Turhan, Zeynep Can<br>Level: 1, State: Excluded	Wrong publication type (eg reviews, opinions, conference proceedings)
594	RefID: 594, Safety monitoring of Influenza A/H1N1 pandemic vaccines in EudraVigilance	Not about HPV vaccination/vax attitudes (eg HPV

	Kurz, X., Domergue, F., Slattery, J., Segec, A., Szmigiel, A., Hidalgo-Simon, A.<br>Level: 1, State: Excluded	infection/serology/prevalence; cervical cancer; HPV vax safety)
595	RefID: 595, Knowledge, perception and attitude towards human papillomavirus among pre-university students in Malaysia<br>Kwang, N. B., Yee, C. M., Shan, L. P., Teik, C. K., Chandralega, K. N., Abdul Kadir, A. K.<br>Level: 1, State: Excluded	Not about HPV vaccination/vax attitudes (eg HPV infection/serology/prevalence; cervical cancer; HPV vax safety)
597	RefID: 597, HPV Vaccine Promotion: The church as an Age - Young adults 18-26yrs not included /outside age rangent of change<br>Lahijani, A. Y., King, A. R., Gullatte, M. M., Hennink, M., Bednarczyk, R. A.<br>Level: 1, State: Excluded	Study Design - Not qualitative (methods or analysis) OR Qualitative survey data not analyzed qualitatively (only numeric stats)
598	RefID: 598, Diverse Families' Experiences with HPV Vaccine Information Sources: A Community-Based Participatory Approach<br>Lai, D., Bodson, J., Davis, F. A., Lee, D., Tavake-Pasi, F., Napia, E., Villalta, J., Mukundente, V., Mooney, R., Coulter, H., Stark, L. A., Sanchez-Birkhead, A. C., Kepka, D.<br>Level: 1, State: Excluded	Wrong/no population (eg parents, providers, children, policy makers, does not include 18-26yrs olds; social media posts)
599	RefID: 599, Discordance Between Human Papillomavirus Twitter ImAge - Young adults 18-26yrs not included /outside age ranges and Disparities in Human Papillomavirus Risk and Disease in the United States: Mixed-Methods Analysis<br>Lama, Y., Chen, T., Dredze, M., Jamison, A., Quinn, S. C., Broniatowski, D. A.<br>Level: 1, State: Excluded	Wrong/no population (eg parents, providers, children, policy makers, does not include 18-26yrs olds; social media posts)
600	RefID: 600, Characterizing Trends in Human Papillomavirus Vaccine Discourse on Reddit (2007-2015): An Observational Study<br>Lama, Y., Hu, D., Jamison, A., Quinn, S. C., Broniatowski, D. A.<br>Level: 1, State: Excluded	Not about HPV vaccination/vax attitudes (eg HPV infection/serology/prevalence; cervical cancer; HPV vax safety)
601	RefID: 601, Improving Human Papillomavirus Vaccination Uptake in College Students: A Socioecological Perspective<br>Lanning, Beth, Golman, Mandy, Crosslin, Katie<br>Level: 1, State: Excluded	Wrong publication type (eg reviews, opinions, conference proceedings)
602	RefID: 602, Predictors of non-vaccination against human papillomavirus among us women Age - Young adults 18-26yrs not included /outside age ranged 18-26: Data from the 2010 national health interview survey<br>Larson, H. E., Caffrey, A., Paiva, A., Willey, C.<br>Level: 1, State: Excluded	Study Design - Not qualitative (methods or analysis) OR Qualitative survey data not analyzed qualitatively (only numeric stats)
603	RefID: 603, HPV Vaccine and College-Age - Young adults 18-26yrs not included /outside age range Men: A Scoping Review<br>Laserson, A. K., Oliffe, J. L., Krist, J., Kelly, M. T.<br>Level: 1, State: Excluded	Study Design - Not qualitative (methods or analysis) OR Qualitative survey data not analyzed qualitatively (only numeric stats)
604	RefID: 604, Human papillomavirus vaccine uptake: What works and what can we do better?<br>Lavelanet, A. F., Stampler, K. M., Dunton, C. J.<br>Level: 1, State: Excluded	Wrong publication type (eg reviews, opinions, conference proceedings)

605	RefID: 605, Attitudes of parents of female secondary school students towards the HPV vaccine<br>Lawless, K., Poole, C., Murphy, P.<br>Level: 1, State: Excluded	Wrong/no population (eg parents, providers, children, policy makers, does not include 18-26yrs olds; social media posts)
606	RefID: 606, Human papillomavirus vaccine uptake among 18- to 26-year-old women in the United States: National Health Interview Survey, 2010<br>Laz, T. H., Rahman, M., Berenson, A. B.<br>Level: 1, State: Excluded	Wrong/no population (eg parents, providers, children, policy makers, does not include 18-26yrs olds; social media posts)
607	RefID: 607, Prevention and control of neoplasms associated with HPV in high-risk groups in Mexico City: The Condesa Study<br>Lazcano-Ponce, E., Salmerón, J., González, A., Allen-Leigh, B., León-Maldonado, L., Magis, C., Aranda-Flores, C., Conde-González, C., Portillo-Romero, A. J., Yunes-Díaz, E., Rivera-Rivera, L., Vargas, G., Nyitray, A. G., Giuliano, A. R.<br>Level: 1, State: Excluded	Not about HPV vaccination/vax attitudes (eg HPV infection/serology/prevalence; cervical cancer; HPV vax safety)
608	RefID: 608, The impact of pharmacist involvement on immunization uptake and other outcomes: An updated systematic review and meta-analysis<br>Le, L. M., Veettil, S. K., Donaldson, D., Kategeaw, W., Hutubessy, R., Lambach, P., Chaiyakunapruk, N.<br>Level: 1, State: Excluded	Not about HPV vaccination/vax attitudes (eg HPV infection/serology/prevalence; cervical cancer; HPV vax safety)
609	RefID: 609, The impact of HPV vaccine narratives on social media: Testing narrative engAge - Young adults 18-26yrs not included /outside age rangement theory with a diverse sample of young adults<br>Leader, A. E., Miller-Day, M., Rey, R. T., Selvan, P., Pezalla, A. E., Hecht, M. L.<br>Level: 1, State: Excluded	Wrong publication type (eg reviews, opinions, conference proceedings)
610	RefID: 610, mHealth pilot study: Text messaging intervention to promote HPV vaccination<br>Lee, Hee Yun, Koopmeiners, Joseph S., McHugh, Jennifer, Raveis, Victoria H., Ahluwalia, Jasjit S.<br>Level: 1, State: Excluded	Study Design - Not qualitative (methods or analysis) OR Qualitative survey data not analyzed qualitatively (only numeric stats)
611	RefID: 611, Parents' knowledge and perceptions of HPV male outcomes and intention to vaccinate sons<br>Lees, A. C., Desousa, N., Bailey, S. L., Coyne-Beasley, T.<br>Level: 1, State: Excluded	Study Design - Not qualitative (methods or analysis) OR Qualitative survey data not analyzed qualitatively (only numeric stats)
612	RefID: 612, Overall efficacy of HPV-16/18 AS04-adjuvanted vaccine against grade 3 or greater cervical intraepithelial neoplasia: 4-year end-of-study analysis of the randomised, double-blind PATRICIA trial<br>Lehtinen, M., Paavonen, J., Wheeler, C. M., Jaisamrarn, U., Garland, S. M., Castellsagué, X., Skinner, S. R., Apter, D., Naud, P., Salmerón, J., et al.<br>Level: 1, State: Excluded	Wrong/no population (eg parents, providers, children, policy makers, does not include 18-26yrs olds; social media posts)
613	RefID: 613, Young adults and acceptance of the human papillomavirus vaccine<br>Lenselink, C. H., Schmeink, C. E., Melchers, W. J., Massuger, L. F., Hendriks, J. C., van Hamont, D., Bekkers, R. L.<br>Level: 2, State: Excluded	Not about HPV vaccination/vax attitudes (eg HPV infection/serology/prevalence; cervical cancer; HPV vax safety)

616	RefID: 616, Physical assessment in pharmacy practice: Perspectives from pharmacists, nonpharmacist health care providers and the public<br>Leong, C., Soufi, L.<br>Level: 1, State: Excluded	Study Design - Not qualitative (methods or analysis) OR Qualitative survey data not analyzed qualitatively (only numeric stats)
617	RefID: 617, Effect of a group educational intervention on rural Chinese women's knowledge and attitudes about human papillomavirus (HPV) and HPV vaccines<br>Li, J., Kang, L. N., Li, B., Pang, Y., Huang, R., Qiao, Y. L.<br>Level: 1, State: Excluded	Not about HPV vaccination/vax attitudes (eg HPV infection/serology/prevalence; cervical cancer; HPV vax safety)
619	RefID: 619, Valuation of the economic benefits of human papillomavirus vaccine in Taiwan<br>Liao, C. H., Liu, J. T., Pwu, R. F., You, S. L., Chow, I., Tang, C. H.<br>Level: 1, State: Excluded	Study Design - Not qualitative (methods or analysis) OR Qualitative survey data not analyzed qualitatively (only numeric stats)
620	RefID: 620, Is use of the human papillomavirus vaccine among female college students related to human papillomavirus knowledge and risk perception?<br>Licht, A. S., Murphy, J. M., Hyland, A. J., Fix, B. V., Hawk, L. W., Mahoney, M. C.<br>Level: 1, State: Excluded	Not about HPV vaccination/vax attitudes (eg HPV infection/serology/prevalence; cervical cancer; HPV vax safety)
621	RefID: 621, Acceptability of human papillomavirus vaccine for males: a review of the literature<br>Liddon, N., Hood, J., Wynn, B. A., Markowitz, L. E.<br>Level: 1, State: Excluded	Study Design - Not qualitative (methods or analysis) OR Qualitative survey data not analyzed qualitatively (only numeric stats)
622	RefID: 622, HPV infection and the genital cytokine milieu in women at high risk of HIV acquisition<br>Liebenberg, L. J. P., McKinnon, L. R., Yende-Zuma, N., Garrett, N., Baxter, C., Kharsany, A. B. M., Archary, D., Rositch, A., Samsunder, N., Mansoor, L. E., Passmore, J. A. S., Abdool Karim, S. S., Abdool Karim, Q.<br>Level: 1, State: Excluded	Wrong publication type (eg reviews, opinions, conference proceedings)
627	RefID: 627, Human papillomavirus prevalence and behavioral risk factors among HIV-infected and HIVuninfected men who have sex with men in Taiwan<br>Lin, C. C., Hsieh, M. C., Hung, H. C., Tsao, S. M., Chen, S. C., Yang, H. J., Lee, Y. T.<br>Level: 1, State: Excluded	Not about HPV vaccination/vax attitudes (eg HPV infection/serology/prevalence; cervical cancer; HPV vax safety)
628	RefID: 628, Latinx fathers report low awareness and knowledge of the human papillomavirus vaccine, but high willingness to vaccinate their children if recommended by a healthcare provider: A qualitative study<br>Lindsay, A. C., Delgado, D., Valdez, M. J., Granberry, P.<br>Level: 1, State: Excluded	Study Design - Not qualitative (methods or analysis) OR Qualitative survey data not analyzed qualitatively (only numeric stats)
629	RefID: 629, "I don't Think He Needs the HPV Vaccine Cause Boys Can't Have Cervical Cancer": a Qualitative Study of Latina Mothers' (Mis) Understandings About Human Papillomavirus Transmission, Associated Cancers, and the Vaccine<br>Lindsay, A. C., Delgado, D., Valdez, M. J., Restrepo, E., Guzman, Y. M.<br>Level: 1, State: Excluded	Wrong/no population (eg parents, providers, children, policy makers, does not include 18-26yrs olds; social media posts)

631	RefID: 631, Central American Immigrant Parents' Awareness, Acceptability, and Willingness to Vaccinate Their Adolescent Children Against Human Papillomavirus: A Pilot Cross-Sectional Study<br>Lindsay, A. C., Pineda, J. A., Valdez, M. J., Torres, M. I., Granberry, P. J.<br>Level: 1, State: Excluded	Wrong/no population (eg parents, providers, children, policy makers, does not include 18-26yrs olds; social media posts)
632	RefID: 632, Maternal and infant outcomes following inadvertent HPV vaccination<br>Lipkind, H. S., Vazquez-Benitez, G., Nordin, J. D., Naleway, A. L., Klein, N. P., Hechter, R. C., Jackson, M. L., Omer, S. B., Lee, G. M., Kharbanda, E. O.<br>Level: 1, State: Excluded	Wrong/no population (eg parents, providers, children, policy makers, does not include 18-26yrs olds; social media posts)
633	RefID: 633, Maternal and Infant Outcomes After Human Papillomavirus Vaccination in the Periconceptional Period or During Pregnancy<br>Lipkind, H. S., Vazquez-Benitez, G., Nordin, J. D., Romitti, P. A., Naleway, A. L., Klein, N. P., Hechter, R. C., Jackson, M. L., Hambidge, S. J., Lee, G. M., Sukumaran, L., Kharbanda, E. O.<br>Level: 1, State: Excluded	Not about HPV vaccination/vax attitudes (eg HPV infection/serology/prevalence; cervical cancer; HPV vax safety)
635	RefID: 635, Validation of decisional balance and self-efficacy measures for HPV vaccination in college women<br>Lipschitz, J. M., Fernandez, A. C., Larson, H. E., Blaney, C. L., Meier, K. S., Redding, C. A., Prochaska, J. O., Paiva, A. L.<br>Level: 1, State: Excluded	Not about HPV vaccination/vax attitudes (eg HPV infection/serology/prevalence; cervical cancer; HPV vax safety)
636	RefID: 636, ACA Provisions Associated With Increase In PercentAge - Young adults 18-26yrs not included /outside age range Of Young Adult Women Initiating And Completing The HPV Vaccine<br>Lipton, B. J., Decker, S. L.<br>Level: 1, State: Excluded	Not about HPV vaccination/vax attitudes (eg HPV infection/serology/prevalence; cervical cancer; HPV vax safety)
638	RefID: 638, Perceptions of teachers, parents and adolescents about HPV, cervical cancer and HPV vaccination<br>Lismidiati, W., Emilia, O., Widyawati<br>Level: 1, State: Excluded	Study Design - Not qualitative (methods or analysis) OR Qualitative survey data not analyzed qualitatively (only numeric stats)
639	RefID: 639, Need vs. Financing Capability: Human Papillomavirus Vaccinations among Adolescents<br>Lismidiati, W., Emilia, O., Widyawati, W.<br>Level: 2, State: Excluded	Wrong/no population (eg parents, providers, children, policy makers, does not include 18-26yrs olds; social media posts)
640	RefID: 640, Genital warts and chlamydia in Australian women: comparison of national Wrong/no population (eg parents, providers, children, policy makers, does not include 18-26yrs olds; social media posts)-based surveys in 2001 and 2011<br>Liu, B., Donovan, B., Brotherton, J. M., Saville, M., Kaldor, J. M.<br>Level: 1, State: Excluded	Wrong/no population (eg parents, providers, children, policy makers, does not include 18-26yrs olds; social media posts)
642	RefID: 642, Now or future? Analyzing the effects of messAge - Young adults 18-26yrs not included /outside age range frame and format in motivating Chinese females to get HPV vaccines for their children<br>Liu, S., Yang, J. Z., Chu, H.<br>Level: 1, State: Excluded	Not about HPV vaccination/vax attitudes (eg HPV infection/serology/prevalence; cervical cancer; HPV vax safety)
643	RefID: 643, Prevalence and genotypes of anal human papillomavirus infection among HIV-positive vs. HIV-negative men in Taizhou, China	Study Design - Not qualitative (methods or analysis) OR

	Liu, X., Lin, H., Chen, X., Shen, W., Ye, X., Lin, Y., Lin, Z., Zhou, S., Gao, M., Ding, Y., He, N.<br>Level: 1, State: Excluded	Qualitative survey data not analyzed qualitatively (only numeric stats)
644	RefID: 644, Active Surveillance of Adverse Events Following Human Papillomavirus Vaccination: Feasibility Pilot Study Based on the Regional Health Care Information Platform in the City of Ningbo, China<br>Liu, Z., Zhang, L., Yang, Y., Meng, R., Fang, T., Dong, Y., Li, N., Xu, G., Zhan, S.<br>Level: 1, State: Excluded	Not about HPV vaccination/vax attitudes (eg HPV infection/serology/prevalence; cervical cancer; HPV vax safety)
645	RefID: 645, Mucosal and cutaneous Human Papillomavirus seroprevalence among adults in the prevaccine era in Germany - Results from a nationwide Wrong/no population (eg parents, providers, children, policy makers, does not include 18-26yrs olds; social media posts)-based survey<br>Loenenbach, A. D., Poethko-Müller, C., Pawlita, M., Thamm, M., Harder, T., Waterboer, T., Schröter, J., Deleré, Y., Wichmann, O., Wiese-Posselt, M.<br>Level: 1, State: Excluded	Not about HPV vaccination/vax attitudes (eg HPV infection/serology/prevalence; cervical cancer; HPV vax safety)
647	RefID: 647, Five medical education podcasts you need to know<br>Lomayesva, N. L., Martin, A. S., Dowley, P. A., Davies, N. W., Olyha, S. J., Wijesekera, T. P.<br>Level: 1, State: Excluded	Age - Young adults 18-26yrs not included /outside age range
648	RefID: 648, Targeting human papillomavirus (HPV) vaccination in international college students<br>Long, A., Roberts, C., Parrish, E. B.<br>Level: 1, State: Excluded	Not about HPV vaccination/vax attitudes (eg HPV infection/serology/prevalence; cervical cancer; HPV vax safety)
649	RefID: 649, Treatment options for high-grade squamous intraepithelial lesions<br>Long, S., Leeman, L.<br>Level: 1, State: Excluded	Not about HPV vaccination/vax attitudes (eg HPV infection/serology/prevalence; cervical cancer; HPV vax safety)
650	RefID: 650, Hospitalizations associated with malignant neoplasia and in situ carcinoma in the anus and penis in men and women during a 5-year period (2009-2013) in Spain: An epidemiological study<br>López, N., Gil-de-Miguel, Á, Pascual-García, R., Gil-Prieto, R.<br>Level: 1, State: Excluded	Not about HPV vaccination/vax attitudes (eg HPV infection/serology/prevalence; cervical cancer; HPV vax safety)
653	RefID: 653, Hospitalization burden associated with malignant neoplasia and in situ carcinoma in vulva and vagina during a 5-year period (2009-2013) in Spain: An epidemiological study<br>López, N., Gil-de-Miguel, Á, Pascual-García, R., Ramón, Y. Cajal J. M., Gil-Prieto, R.<br>Level: 1, State: Excluded	Not about HPV vaccination/vax attitudes (eg HPV infection/serology/prevalence; cervical cancer; HPV vax safety)
654	RefID: 654, Results on exposure during pregnancy from a pregnancy registry for AS04-HPV-16/18 vaccine<br>López-Fauqued, M., Zima, J., Angelo, M. G., Stegmann, J. U.<br>Level: 1, State: Excluded	Not about HPV vaccination/vax attitudes (eg HPV infection/serology/prevalence; cervical cancer; HPV vax safety)
655	RefID: 655, Interventions to increase uptake of Human Papillomavirus (HPV) vaccination in minority Wrong/no population (eg parents, providers, children, policy makers, does not include 18-26yrs olds; social media posts)s: A systematic review	Not about HPV vaccination/vax attitudes (eg HPV infection/serology/prevalence; cervical cancer; HPV vax safety)

	Lott, B. E., Okusanya, B. O., Anderson, E. J., Kram, N. A., Rodriguez, M., Thomson, C. A., Rosales, C., Ehiri, J. E.<br>Level: 1, State: Excluded	
656	RefID: 656, Opsoclonus myoclonus syndrome in a young adult after a measles, mumps and rubella vaccine<br>Loustalot, M. C., Savini, H., Taugourdeau-Raymond, S., Rouby, F., Simon, F., Jean-Pastor, M. J.<br>Level: 1, State: Excluded	Wrong publication type (eg reviews, opinions, conference proceedings)
657	RefID: 657, Preventing multiple types of cancer through HPV vaccination<br>Lowy, D. R.<br>Level: 1, State: Excluded	Not about HPV vaccination/vax attitudes (eg HPV infection/serology/prevalence; cervical cancer; HPV vax safety)
658	RefID: 658, Current and future vaccines to prevent HPV-associated cancers<br>Lowy, D. R., Day, P. M., Kines, R. C., Thompson, C. D., Schiller, J. T.<br>Level: 1, State: Excluded	Not about HPV vaccination/vax attitudes (eg HPV infection/serology/prevalence; cervical cancer; HPV vax safety)
659	RefID: 659, Human papillomavirus vaccine initiation and awareness: U.S. young men in the 2010 National Health Interview Survey<br>Lu, P., Williams, W. W., Li, J., Dorell, C., Yankey, D., Kepka, D., Dunne, E. F.<br>Level: 1, State: Excluded	Study Design - Not qualitative (methods or analysis) OR Qualitative survey data not analyzed qualitatively (only numeric stats)
660	RefID: 660, Impact of health insurance status on vaccination coverAge - Young adults 18-26yrs not included /outside age range among adult Wrong/no population (eg parents, providers, children, policy makers, does not include 18-26yrs olds; social media posts)s<br>Lu, Peng-jun, O'Halloran, Alissa, Williams, Walter W.<br>Level: 1, State: Excluded	Study Design - Not qualitative (methods or analysis) OR Qualitative survey data not analyzed qualitatively (only numeric stats)
661	RefID: 661, Racial and ethnic disparities in vaccination coverAge - Young adults 18-26yrs not included /outside age range among adult Wrong/no population (eg parents, providers, children, policy makers, does not include 18-26yrs olds; social media posts)s in the US<br>Lu, Peng-jun, O'Halloran, Alissa, Williams, Walter W., Lindley, Megan C., Farrall, Susan, Bridges, Carolyn B.<br>Level: 1, State: Excluded	Not about HPV vaccination/vax attitudes (eg HPV infection/serology/prevalence; cervical cancer; HPV vax safety)
664	RefID: 664, Surveillance of Vaccination CoverAge - Young adults 18-26yrs not included /outside age range Among Adult Wrong/no population (eg parents, providers, children, policy makers, does not include 18-26yrs olds; social media posts)s -United States, 2018<br>Lu, P. J., Hung, M. C., Srivastav, A., Grohskopf, L. A., Kobayashi, M., Harris, A. M., Dooling, K. L., Markowitz, L. E., Rodriguez-Lainz, A., Williams, W. W.<br>Level: 1, State: Excluded	Study Design - Not qualitative (methods or analysis) OR Qualitative survey data not analyzed qualitatively (only numeric stats)
668	RefID: 668, Prevalence of high-risk human papillomavirus and cervical lesion risk factors: A Wrong/no population (eg parents, providers, children, policy makers, does not include 18-26yrs olds; social media posts)-based study in Zhejiang, China 2010–2019<br>Lu, W., Chen, T., Yao, Y., Chen, P.<br>Level: 1, State: Excluded	Not about HPV vaccination/vax attitudes (eg HPV infection/serology/prevalence; cervical cancer; HPV vax safety)

669	RefID: 669, Association of HIV infection with distribution and viral load of HPV types in Kenya: a survey with 820 female sex workers<br>Luchters, S. M., Broeck, D. V., Chersich, M. F., Nel, A., Delva, W., Mandaliya, K., Depuydt, C. E., Claeys, P., Bogers, J. P., Temmerman, M., Luchters, Stanley M. F., Vanden Broeck, Davy, Chersich, Matthew F., Nel, Annalene, Delva, Wim, Mandaliya, Kishor, Depuydt, Christophe E., Claeys, Patricia, Bogers, John-Paul, Temmerman, Marleen<br>Level: 1, State: Excluded	Not about HPV vaccination/vax attitudes (eg HPV infection/serology/prevalence; cervical cancer; HPV vax safety)
672	RefID: 672, DON'T STOP HPV VACCINE WITH ONE DOSE LEFT, GO SEE AN ALLERGIST!<br>Lukas, M., Tankersley, M.<br>Level: 1, State: Excluded	Not about HPV vaccination/vax attitudes (eg HPV infection/serology/prevalence; cervical cancer; HPV vax safety)
673	RefID: 673, Prognostic role of immunohistochemical overexpression of the p16 protein in women under the Age - Young adults 18-26yrs not included /outside age range of 35 and diagnosed with HSIL (CIN2) subjected to "cervix sparing" excision<br>Lukic, A., Rossi, S., Frega, A., Ruscito, I., Bianchi, P., Nobili, F., Caserta, D., Vecchione, A.<br>Level: 1, State: Excluded	Not about HPV vaccination/vax attitudes (eg HPV infection/serology/prevalence; cervical cancer; HPV vax safety)
674	RefID: 674, Long-term follow-up observation of the safety, immunogenicity, and effectiveness of Gardasil™ in adult women<br>Luna, J., Plata, M., Gonzalez, M., Correa, A., Maldonado, I., Nossa, C., Radley, D., Vuocolo, S., Haupt, R. M., Saah, A.<br>Level: 1, State: Excluded	Not about HPV vaccination/vax attitudes (eg HPV infection/serology/prevalence; cervical cancer; HPV vax safety)
675	RefID: 675, Formative Research on HPV Vaccine Acceptability Among Latina Farmworkers<br>Luque, John S., Castañeda, Heide, Tyson, Dinorah Martinez, Vargas, Natalia, Meade, Cathy D.<br>Level: 2, State: Excluded	Not about HPV vaccination/vax attitudes (eg HPV infection/serology/prevalence; cervical cancer; HPV vax safety)
676	RefID: 676, [Gynaecologists' attitudes and practices towards HPV vaccination: a quantitative-qualitative study in Rhône-Alpes]<br>Lutringer-Magnin, D., Kalecinski, J., Barone, G., Borne, H., Regnier, V., Vanhems, P., Chauvin, F., Lasset, C.<br>Level: 1, State: Excluded	Multiple Wrong/no population (eg parents, providers, children, policy makers, does not include 18-26yrs olds; social media posts)s - unable to extract data specific to young adults (18-26 yrs)
677	RefID: 677, Prevention of sexually transmitted infections among girls and young women in relation to their HPV vaccination status<br>Lutringer-Magnin, Delphine, Kalecinski, Julie, Cropet, Claire, Barone, Giovanna, Ronin, Vincent, Régnier, Véronique, Leocmach, Yann, Jacquard, Anne-Carole, Vanhems, Philippe, Chauvin, Franck, Lasset, Christine<br>Level: 1, State: Excluded	Wrong/no population (eg parents, providers, children, policy makers, does not include 18-26yrs olds; social media posts)
678	RefID: 678, Presence of human papillomavirus in semen of healthy men is firmly associated with HPV infections of the penile epithelium<br>Luttmer, R., Dijkstra, M. G., Snijders, P. J. F., Jordanova, E. S., King, A. J., Pronk, D. T. M., Foresta, C., Garolla, A., Hompes, P. G. A., Berkhof, J., Bleeker, M. C. G., Doorbar, J., Heideman, D. A. M., Meijer, Cjlm<br>Level: 1, State: Excluded	Age - Young adults 18-26yrs not included /outside age range
679	RefID: 679, Barriers and supports for uptake of human papillomavirus vaccination in Indigenous people globally: A systematic review	Not about HPV vaccination/vax attitudes (eg HPV

	MacDonald, S. E., Kenzie, L., Letendre, A., Bill, L., Shea-Budgell, M., Henderson, R., Barnabe, C., Guichon, J. R., Colquhoun, A., Ganshorn, H., Bedingfield, N., Vandenboogaard, P. D., Bednarczyk, R. A., Glaze, S., Nelson, G.<br>Level: 1, State: Excluded	infection/serology/prevalence; cervical cancer; HPV vax safety)
680	RefID: 680, Natural Antibodies to Human Papillomavirus 16 and Recurrence of Vulvar High-Grade Intraepithelial Neoplasia (VIN3)<br>Madeleine, M. M., Johnson, L. G., Doody, D. R., Tipton, E. R., Carter, J. J., Galloway, D. A.<br>Level: 1, State: Excluded	Wrong publication type (eg reviews, opinions, conference proceedings)
681	RefID: 681, Using Community EngAge - Young adults 18-26yrs not included /outside age rangement to Develop a Web-Based Intervention for Latinos about the HPV Vaccine<br>Maertens, J. A., Jimenez-Zambrano, A. M., Albright, K., Dempsey, A. F.<br>Level: 2, State: Excluded	Study Design - Not qualitative (methods or analysis) OR Qualitative survey data not analyzed qualitatively (only numeric stats)
682	RefID: 682, Knowledge of Human Papillomavirus Infection, Cervical Cancer and Willingness to pay for Cervical Cancer Vaccination among Ethnically Diverse Medical Students in Malaysia<br>Maharajan, M. K., Rajiah, K., Num, K. S., Yong, N. J.<br>Level: 1, State: Excluded	Not about HPV vaccination/vax attitudes (eg HPV infection/serology/prevalence; cervical cancer; HPV vax safety)
684	RefID: 684, Students' knowledge about cervical cancer and vaccine against human papillomavirus (HPV)<br>Maksimiuk, M., Sobiborowicz, A., Beranek, O., Sobol, M., Badowska-Kozakiewicz, A. M.<br>Level: 1, State: Excluded	Study Design - Not qualitative (methods or analysis) OR Qualitative survey data not analyzed qualitatively (only numeric stats)
685	RefID: 685, Factors influencing human papillomavirus school-based immunization in Alberta: A mixed-methods study protocol<br>Malkin, J., Allen Scott, L., Alberga Machado, A., Teare, G., Snider, J., Ali Tirmizi, S. F., Bandara, T., Rathwell, M., Neudorf, C.<br>Level: 1, State: Excluded	Study Design - Not qualitative (methods or analysis) OR Qualitative survey data not analyzed qualitatively (only numeric stats)
687	RefID: 687, 'what does it matter?' young sexual minority men discuss their conversations with sexual partners about hpv vaccination<br>Malone, Molly A., Gower, Amy L., Reiter, Paul L., Kiss, Dale E., McRee, Annie-Laurie<br>Level: 1, State: Excluded	Wrong/no population (eg parents, providers, children, policy makers, does not include 18-26yrs olds; social media posts)
690	RefID: 690, Evaluating the proposal of paediatric virology: An interview with Professor Tina Dalianis, Professor of Tumour Virology at Karolinska Institutet<br>Mammas, I. N., Spandidos, D. A.<br>Level: 1, State: Excluded	Not about HPV vaccination/vax attitudes (eg HPV infection/serology/prevalence; cervical cancer; HPV vax safety)
691	RefID: 691, Epidemiological patterns of cervical human papillomavirus infection among women presenting for cervical cancer screening in North-Eastern Nigeria<br>Manga, M. M., Fowotade, A., Abdullahi, Y. M., El-Nafaty, A. U., Adamu, D. B., Pindiga, H. U., Bakare, R. A., Osoba, A. O.<br>Level: 1, State: Excluded	Wrong publication type (eg reviews, opinions, conference proceedings)
693	RefID: 693, HPV vaccination among a community sample of young adult women<br>Manhart, L. E., Burgess-Hull, A. J., Fleming, C. B., Bailey, J. A., Haggerty,	Not about HPV vaccination/vax attitudes (eg HPV

	K. P., Catalano, R. F.<br>Level: 1, State: Excluded	infection/serology/prevalence; cervical cancer; HPV vax safety)
694	RefID: 694, Barriers and Facilitators of Romanian HPV (Human Papillomavirus) Vaccination<br>Manolescu, L. S. C., Zugravu, C., Zaharia, C. N., Dumitrescu, A. I., Prasacu, I., Radu, M. C., Letiția, G. D., Nita, I., Cristache, C. M., Gales, L. N.<br>Level: 1, State: Excluded	Study Design - Not qualitative (methods or analysis) OR Qualitative survey data not analyzed qualitatively (only numeric stats)
695	RefID: 695, Human papillomavirus vaccine initiation and completion among heterosexual and sexual minority young adult men (18-26 years) in the United States, 2013-2014<br>Mansh, M., Liszewski, W., Arron, S.<br>Level: 1, State: Excluded	Study Design - Not qualitative (methods or analysis) OR Qualitative survey data not analyzed qualitatively (only numeric stats)
696	RefID: 696, Human papillomavirus vaccine initiation and completion among heterosexual and sexual minority young adult women in the United States<br>Mansh, M., Liszewski, W., Arron, S.<br>Level: 1, State: Excluded	Not about HPV vaccination/vax attitudes (eg HPV infection/serology/prevalence; cervical cancer; HPV vax safety)
697	RefID: 697, One Vax Two Lives: a social media campaign and research program to address COVID-19 vaccine hesitancy in pregnancy<br>Marcell, L., Dokania, E., Navia, I., Baxter, C., Crary, I., Rutz, S., Soto Monteverde, M. J., Simlai, S., Hernandez, C., Huebner, E. M., Sanchez, M., Cox, E., Stonehill, A., Koltai, K., Adams Waldorf, K. M.<br>Level: 1, State: Excluded	Not about HPV vaccination/vax attitudes (eg HPV infection/serology/prevalence; cervical cancer; HPV vax safety)
698	RefID: 698, RW3 HUMAN PAPILLOMA VIRUS IN ITALY: RETROSPECTIVE COHORT ANALYSIS AND VACCINATION EFFECT FROM REAL-WORLD DATA<br>Marcellusi, A., Fabiano, G., Sciattella, P., Favato, G., Mennini, F. S.<br>Level: 1, State: Excluded	Not about HPV vaccination/vax attitudes (eg HPV infection/serology/prevalence; cervical cancer; HPV vax safety)
699	RefID: 699, HPV vaccination in young males: a glimpse of coverAge - Young adults 18-26yrs not included /outside age range, parental attitude and need of additional information from Lombardy region, Italy<br>Mari, A., Gianolio, L., Edefonti, V., Hashemian, D. K., Casini, F., Bergamaschi, F., Sala, A., Verduci, E., Calcaterra, V., Zuccotti, G. V., Fabiano, V.<br>Level: 1, State: Excluded	Study Design - Not qualitative (methods or analysis) OR Qualitative survey data not analyzed qualitatively (only numeric stats)
700	RefID: 700, Effects of Human Papillomavirus Awareness and Knowledge on Psychological State of Women Referred to Cervical Cancer Screening<br>Markovic-Denic, L., Djuric, O., Maksimovic, N., Popovac, S., Kesic, V.<br>Level: 1, State: Excluded	Wrong/no population (eg parents, providers, children, policy makers, does not include 18-26yrs olds; social media posts)
701	RefID: 701, Prevalence of HPV After Introduction of the Vaccination Program in the United States<br>Markowitz, L. E., Liu, G., Hariri, S., Steinau, M., Dunne, E. F., Unger, E. R.<br>Level: 1, State: Excluded	Not about HPV vaccination/vax attitudes (eg HPV infection/serology/prevalence; cervical cancer; HPV vax safety)
702	RefID: 702, Human papillomavirus (HPV) information needs: a theoretical framework<br>Marlow, L. A., Wardle, J., Grant, N., Waller, J.<br>Level: 2, State: Excluded	Not about HPV vaccination/vax attitudes (eg HPV infection/serology/prevalence; cervical cancer; HPV vax safety)

704	RefID: 704, Parental and societal support for adolescent immunization through school based immunization programs<br>Marshall, H. S., Collins, J., Sullivan, T., Tooher, R., O'Keefe, M., Skinner, S. R., Watson, M., Burgess, T., Ashmeade, H., Braunack-Mayer, A.<br>Level: 1, State: Excluded	Wrong/no population (eg parents, providers, children, policy makers, does not include 18-26yrs olds; social media posts)
706	RefID: 706, An Electronic Medical Record Alert Intervention to Improve HPV Vaccination Among Eligible Male College Students at a University Student Health Center<br>Martin, S., Warner, E. L., Kirchhoff, A. C., Mooney, R., Martel, L., Kepka, D.<br>Level: 1, State: Excluded	Wrong/no population (eg parents, providers, children, policy makers, does not include 18-26yrs olds; social media posts)
707	RefID: 707, The recording of human papillomavirus (HPV) vaccination in BIFAP primary care database: A validation study<br>Martín-Merino, E., Llorente-García, A., Montero-Corominas, D., Huerta, C.<br>Level: 1, State: Excluded	Wrong/no population (eg parents, providers, children, policy makers, does not include 18-26yrs olds; social media posts)
708	RefID: 708, [Prevalence of HPV high-risk serotypes detected by PCR in patients with normal cervical cytology at the Hospital Regional Adolfo López Mateos, ISSSTE]<br>Martínez-Portilla, R. J., López-Velázquez, J. L., Martínez-Rojas, G. C., Aguilar-Villagómez, M. I., De la Torre-Rendón, F. E., Villafán-Bernal, J. R.<br>Level: 1, State: Excluded	Not about HPV vaccination/vax attitudes (eg HPV infection/serology/prevalence; cervical cancer; HPV vax safety)
709	RefID: 709, Comprehensive sexuality education for fraternity-affiliated undergraduates: a pilot program to improve sexual and reproductive health knowledge, attitudes, and communication<br>Master, S. O., Garbers, S., Lynch, L., Bell, D. L., Catallozzi, M., Santelli, J.<br>Level: 1, State: Excluded	Not about HPV vaccination/vax attitudes (eg HPV infection/serology/prevalence; cervical cancer; HPV vax safety)
710	RefID: 710, Someone you love: Raising human papilloma-virus awareness<br>Mathews, E., James, T., Neill, K.<br>Level: 1, State: Excluded	Not about HPV vaccination/vax attitudes (eg HPV infection/serology/prevalence; cervical cancer; HPV vax safety)
711	RefID: 711, Lost cohorts in the HPV vaccination due to the covid-19 pandemic and potential effects on future HPV associated cancers<br>Maulbecker-Armstrong, C., Moser, K., Ouedraogo, N.<br>Level: 1, State: Excluded	Not about HPV vaccination/vax attitudes (eg HPV infection/serology/prevalence; cervical cancer; HPV vax safety)
712	RefID: 712, HPV vaccination of adult women: an audit of Australian general practitioners<br>Mazza, D., Petrovic, K., Chakraborty, S.<br>Level: 1, State: Excluded	Not about HPV vaccination/vax attitudes (eg HPV infection/serology/prevalence; cervical cancer; HPV vax safety)
713	RefID: 713, Effect of a multi-modal intervention on immunization rates in obstetrics and gynecology clinics<br>Mazzoni, S. E., Brewer, S. E., Pyrzanowski, J. L., Durfee, M. J., Dickinson, L. M., Barnard, J. G., Dempsey, A. F., O'Leary, S. T.<br>Level: 1, State: Excluded	Wrong/no population (eg parents, providers, children, policy makers, does not include 18-26yrs olds; social media posts)
715	RefID: 715, Unusual and unique distribution of anal high-risk human papillomavirus (HR-HPV) among men who have sex with men living in the Central African Republic<br>Mboumba Bouassa, R. S., Mbeko Simaleko, M., Camengo, S. P., Mossoro-Kpinde, C. D., Veyer, D., Matta, M., Robin, L., Longo, J. D.,	Wrong/no population (eg parents, providers, children, policy makers, does not include 18-26yrs olds; social media posts)

	Grésenguet, G., Péré, H., Meye, J. F., Belec, L.<br>Level: 1, State: Excluded	
716	RefID: 716, Human papillomavirus prevalence in South African women and men according to Age - Young adults 18-26yrs not included /outside age range and human immunodeficiency virus status<br>Mbulawa, Z. Z., Coetzee, D., Williamson, A. L.<br>Level: 1, State: Excluded	Age - Young adults 18-26yrs not included /outside age range
720	RefID: 720, HPV prevalence among young adult women living with and without HIV in Botswana for future HPV vaccine impact monitoring<br>McClung, N., Mathoma, A., Gargano, J. W., Nyepetsi, N. G., Querec, T. D., Onyekwuluje, J., Mine, M., Morroni, C., Luckett, R., Markowitz, L. E., Ramogola-Masire, D.<br>Level: 1, State: Excluded	Not about HPV vaccination/vax attitudes (eg HPV infection/serology/prevalence; cervical cancer; HPV vax safety)
721	RefID: 721, The efficacy of the quadrivalent human papillomavirus vaccine in girls and women living with human immunodeficiency virus<br>McClymont, E., Lee, M., Raboud, J., Coutlée, F., Walmsley, S., Lipsky, N., Loutfy, M., Trottier, S., Smaill, F., Klein, M. B., Harris, M., Cohen, J., Yudin, M. H., Wobeser, W., Money, D.<br>Level: 1, State: Excluded	Not about HPV vaccination/vax attitudes (eg HPV infection/serology/prevalence; cervical cancer; HPV vax safety)
723	RefID: 723, Perceptions of vaccination within a Christian homeschooling community in Pennsylvania<br>McCoy, J. D., Painter, J. E., Jacobsen, K. H.<br>Level: 1, State: Excluded	Not about HPV vaccination/vax attitudes (eg HPV infection/serology/prevalence; cervical cancer; HPV vax safety)
724	RefID: 724, Transcending barriers and creating opportunities: Program evaluation of a cervical cytology/HPV screening program in the Dominican Republic<br>McDonough, E., Hall, C., TofthAge - Young adults 18-26yrs not included /outside age rangen, C.<br>Level: 1, State: Excluded	Wrong/no population (eg parents, providers, children, policy makers, does not include 18-26yrs olds; social media posts)
725	RefID: 725, Race, nativity, and sex disparities in human papillomavirus vaccination among young adults in the USA<br>McElfish, Pearl A., Narcisse, Marie-Rachelle, Felix, Holly C., Cascante, Diana C., Nagarsheth, Nirav, Teeter, Ben, Faramawi, Mohammed F.<br>Level: 1, State: Excluded	Not about HPV vaccination/vax attitudes (eg HPV infection/serology/prevalence; cervical cancer; HPV vax safety)
727	RefID: 727, "If you can't treat HPV, why test for it?" Women's attitudes to the changing face of cervical cancer prevention: a focus group study<br>McRae, J., Martin, C., O'Leary, J., Sharp, L.<br>Level: 2, State: Excluded	Not about HPV vaccination/vax attitudes (eg HPV infection/serology/prevalence; cervical cancer; HPV vax safety)
728	RefID: 728, Vaccinating adolescent girls against human papillomavirus—Who decides?<br>McRee, Annie-Laurie, Reiter, Paul L., Brewer, Noel T.<br>Level: 1, State: Excluded	Multiple Wrong/no population (eg parents, providers, children, policy makers, does not include 18-26yrs olds; social media posts)s - unable to extract data specific to young adults (18-26 yrs)
729	RefID: 729, Mother–Daughter Communication About HPV Vaccine<br>McRee, Annie-Laurie, Reiter, Paul L., Gottlieb, Sami L., Brewer, Noel T.<br>Level: 1, State: Excluded	Wrong/no population (eg parents, providers, children, policy makers, does not include 18-26yrs olds; social media posts)

732	RefID: 732, Outsmart HPV: acceptability and short-term effects of a web-based HPV vaccination intervention for young adult gay and bisexual men<br>McRee, A. L., Shoben, A., Bauermeister, J. A., Katz, M. L., Paskett, E. D., Reiter, P. L.<br>Level: 1, State: Excluded	Study Design - Not qualitative (methods or analysis) OR Qualitative survey data not analyzed qualitatively (only numeric stats)
733	RefID: 733, Effects of a pilot randomized controlled trial of a web-based HPV vaccination intervention for young gay and bisexual men: the outsmart HPV project<br>McRee, A. L., Shoben, A. B., Reiter, P. L.<br>Level: 1, State: Excluded	Study Design - Not qualitative (methods or analysis) OR Qualitative survey data not analyzed qualitatively (only numeric stats)
734	RefID: 734, A fixed herbal combination-a new approach in hpv cervical infection treatment<br>Mehedințu, C., Brătilă, E., Cîrstoiu, M., Vlădăreanu, R., Antonovici, M. R., Brîndușe, L. A., Berceanu, C., Gherghiceanu, F., Navolan, D. A. N., Ionescu, O. M., Criveanu, M.<br>Level: 1, State: Excluded	Study Design - Not qualitative (methods or analysis) OR Qualitative survey data not analyzed qualitatively (only numeric stats)
735	RefID: 735, Human papillomavirus vaccination history among women with precancerous cervical lesions: disparities and barriers<br>Mehta, N. R., Julian, P. J., Meek, J. I., Sosa, L. E., Bilinski, A., Hariri, S., Markowitz, L. E., Hadler, J. L., Niccolai, L. M.<br>Level: 1, State: Excluded	Not about HPV vaccination/vax attitudes (eg HPV infection/serology/prevalence; cervical cancer; HPV vax safety)
738	RefID: 738, Monitoring for Human Papillomavirus Vaccine Impact Among Gay, Bisexual, and Other Men Who Have Sex With Men-United States, 2012-2014<br>Meites, E., Gorbach, P. M., Gratzer, B., Panicker, G., Steinau, M., Collins, T., Parrish, A., Randel, C., McGrath, M., Carrasco, S., Moore, J., Zaidi, A., Braxton, J., Kerndt, P. R., Unger, E. R., Crosby, R. A., Markowitz, L. E.<br>Level: 1, State: Excluded	Study Design - Not qualitative (methods or analysis) OR Qualitative survey data not analyzed qualitatively (only numeric stats)
740	RefID: 740, Low coverAge - Young adults 18-26yrs not included /outside age range of HPV vaccination in the national immunization programme in Brazil: Parental vaccine refusal or barriers in health-service based vaccine delivery?<br>Mendes Lobão, W., Duarte, F. G., Burns, J. D., de Souza Teles Santos, C. A., Chagas de Almeida, M. C., Reingold, A., Duarte Moreira, E. Junior<br>Level: 1, State: Excluded	Not about HPV vaccination/vax attitudes (eg HPV infection/serology/prevalence; cervical cancer; HPV vax safety)
741	RefID: 741, Knowledge and attitude towards cervical cancer among reproductive Age - Young adults 18-26yrs not included /outside age range group women in Gondar town, North West Ethiopia<br>Mengesha, A., Messele, A., Beletew, B.<br>Level: 1, State: Excluded	Wrong/no population (eg parents, providers, children, policy makers, does not include 18-26yrs olds; social media posts)
742	RefID: 742, Time trade-off procedure for measuring health utilities loss with human papillomavirus-induced diseases: a multicenter, retrospective, observational pilot study in Italy<br>Mennini, F. S., Panatto, D., Marcellusi, A., Cristoforoni, P., De Vincenzo, R., Di Capua, E., Ferrandina, G., Petrillo, M., Sasso, T., Ricci, C., Trivellizzi, N., Capone, A., Scambia, G., Gasparini, R.<br>Level: 1, State: Excluded	Study Design - Not qualitative (methods or analysis) OR Qualitative survey data not analyzed qualitatively (only numeric stats)
743	RefID: 743, Treatment patterns and associated costs for genital warts in Italy	Not about HPV vaccination/vax attitudes (eg HPV

	Merito, M., Largeron, N., Cohet, C., Timelli, L., Boselli, F., Matteelli, A., Naldi, L., Vittori, G.<br>Level: 1, State: Excluded	infection/serology/prevalence; cervical cancer; HPV vax safety)
745	RefID: 745, Sexually Transmitted Infections<br>Mermelstein, S., Plax, K.<br>Level: 1, State: Excluded	Not about HPV vaccination/vax attitudes (eg HPV infection/serology/prevalence; cervical cancer; HPV vax safety)
746	RefID: 746, Exploring Young Adult Comfort with Oral Health Care Providers' Discussions Related to HPV: A mixed methods study<br>Merrell, Laura K., Bishop, James M., Henry, Dayna S., Ott Walter, Katherine, Azariah, Wilhelmina Y., Loughlin, Kyra C.<br>Level: 2, State: Excluded	Not about HPV vaccination/vax attitudes (eg HPV infection/serology/prevalence; cervical cancer; HPV vax safety)
748	RefID: 748, Knowledge about the HPV vaccine and factors associated with acceptance in girls of 9 to 12 years old<br>Millán-Morales, R. C., Medina-Gómez, O. S., Villegas-Lara, B.<br>Level: 1, State: Excluded	Not about HPV vaccination/vax attitudes (eg HPV infection/serology/prevalence; cervical cancer; HPV vax safety)
749	RefID: 749, Provision of Adolescent Health Care in Resource-Limited Settings: Perceptions and Training Needs of Health Care Workers<br>Miller, K. K., Saftner, M., Abeso, J., McMorris, B., Olupot-Olupot, P.<br>Level: 1, State: Excluded	Wrong/no population (eg parents, providers, children, policy makers, does not include 18-26yrs olds; social media posts)
750	RefID: 750, Views on Human Papillomavirus Vaccination: A Mixed-Methods Study of Urban Youth<br>Miller, Melissa, Wickliffe, Joi, Jahnke, Sara, Linebarger, Jennifer, Humiston, Sharon<br>Level: 2, State: Excluded	Not about HPV vaccination/vax attitudes (eg HPV infection/serology/prevalence; cervical cancer; HPV vax safety)
755	RefID: 755, Effectiveness of a health talk education program on human papillomavirus (HPV) knowledge, attitudes, and intentions to vaccinate children among mothers of secondary school boys in Thua Thien Hue Province, Vietnam<br>Minh, D. N., Taneepanichskul, N., Hajek, R.<br>Level: 1, State: Excluded	Multiple Wrong/no population (eg parents, providers, children, policy makers, does not include 18-26yrs olds; social media posts)s - unable to extract data specific to young adults (18-26 yrs)
756	RefID: 756, "I Want to Know More about the HPV Vaccine": Stories by Korean American College Women<br>MinJin, Kim<br>Level: 1, State: Excluded	Wrong/no population (eg parents, providers, children, policy makers, does not include 18-26yrs olds; social media posts)
758	RefID: 758, Perceptions on the importance of vaccination and vaccine refusal in a medical school<br>Mizuta, A. H., De Menezes Succi, G., Montalli, V. A. M., De Menezes Succi, R. C.<br>Level: 1, State: Excluded	Study Design - Not qualitative (methods or analysis) OR Qualitative survey data not analyzed qualitatively (only numeric stats)
759	RefID: 759, Using Facebook to reach adolescents for human papillomavirus (HPV) vaccination<br>Mohanty, S., Leader, A. E., Gibeau, E., Johnson, C.<br>Level: 1, State: Excluded	Wrong/no population (eg parents, providers, children, policy makers, does not include 18-26yrs olds; social media posts)
760	RefID: 760, A Constructed Reality? A Fairclough-Inspired Critical Discourse Analysis of the Danish HPV Controversy<br>Mohr, S., Frederiksen, K.<br>Level: 1, State: Excluded	Wrong/no population (eg parents, providers, children, policy makers, does not include 18-26yrs olds; social media posts)

761	RefID: 761, Acceptability of vaccination against human papillomavirus in health students<br>Molero, S. M. M., Alburqueque-Melgarejo, J., Roque-Quezada, J. C., Puicon, W. D. C., Olcese, J. E. V.<br>Level: 1, State: Excluded	Wrong publication type (eg reviews, opinions, conference proceedings)
762	RefID: 762, Knowledge on the HPV vaccine among university students<br>Monteiro, D. L. M., Brollo, L. C. S., de Souza, T. P., Dos Santos, J. R. P., Santos, G. R., Correa, T., da Costa, J. T., de Oliveira, M. A. P., Trajano, A. J. B.<br>Level: 1, State: Excluded	Study Design - Not qualitative (methods or analysis) OR Qualitative survey data not analyzed qualitatively (only numeric stats)
764	RefID: 764, An epidemiological study assessing the prevalence of human papillomavirus types in women in the Kingdom of Bahrain<br>Moosa, K., Alsayyad, A. S., Quint, W., Gopala, K., DeAntonio, R.<br>Level: 1, State: Excluded	Study Design - Not qualitative (methods or analysis) OR Qualitative survey data not analyzed qualitatively (only numeric stats)
765	RefID: 765, A tale of two themes: implementing HPV prevention and assessing stress in the workplace<br>Morabia, A., Costanza, M. C.<br>Level: 1, State: Excluded	Not about HPV vaccination/vax attitudes (eg HPV infection/serology/prevalence; cervical cancer; HPV vax safety)
766	RefID: 766, Hispanic mothers' and high school girls' perceptions of cervical cancer, human papilloma virus, and the human papilloma virus vaccine<br>Morales-Campos, D. Y., Markham, C. M., Peskin, M. F., Fernandez, M. E.<br>Level: 2, State: Excluded	Not about HPV vaccination/vax attitudes (eg HPV infection/serology/prevalence; cervical cancer; HPV vax safety)
768	RefID: 768, Extending cancer 101: Public health graduate students' perception of newly developed HPV module<br>Moreno, L., Marrero, M., Ruiz, L., Vadaparampil, S., Jimenez, J., Giuliano, A., Quinn, G., Antonia, T.<br>Level: 1, State: Excluded	Wrong/no population (eg parents, providers, children, policy makers, does not include 18-26yrs olds; social media posts)
769	RefID: 769, Oral, genital and anal human papillomavirus infections among female sex workers in Ibadan, Nigeria<br>Morhason-Bello, I. O., Baisley, K., Pavon, M. A., Adewole, I. F., Bakare, R. A., de Sanjosé, S., Francis, S. C., Watson-Jones, D.<br>Level: 1, State: Excluded	
770	RefID: 770, Introduction of human papilloma virus vaccine in a low resource setting: A survey of the views of Nigerian gynaecologists<br>Morhason-Bello, I. O., Oladokun, A., Adedokun, B. O., Adesina, O. A., Awolude, O. A., Aimakhu, C. O., Okolo, C. A., Akinwunmi, B. O., Adewole, I. F.<br>Level: 1, State: Excluded	Not about HPV vaccination/vax attitudes (eg HPV infection/serology/prevalence; cervical cancer; HPV vax safety)
771	RefID: 771, Prevalence of potential sexual abuse in adolescents and young adults and feasibility of an assessment and manAge - Young adults 18-26yrs not included /outside age rangement plan used in three research projects<br>Morrow, C., Thomas, R., Ding, L., Kahn, J. A.<br>Level: 1, State: Excluded	Not about HPV vaccination/vax attitudes (eg HPV infection/serology/prevalence; cervical cancer; HPV vax safety)
774	RefID: 774, Human Papillomavirus Antibody Levels and Quadrivalent Vaccine Clinical Effectiveness in Perinatally Human Immunodeficiency Virus–infected and Exposed, Uninfected Youth	Wrong/no population (eg parents, providers, children, policy makers,

	Moscicki, Anna-Barbara, Karalius, Brad, Tassiopoulos, Katherine, Yao, Tzy-Jyun, Jacobson, Denise L., Patel, Kunjal, Purswani, Murli, SeAge - Young adults 18-26yrs not included /outside age range, George R., Study, Pediatric H. I. V. Aids Cohort<br>Level: 1, State: Excluded	does not include 18-26yrs olds; social media posts)
776	RefID: 776, Opportunities for increasing human papillomavirus vaccine provision in school health centers<br>Moss, J. L., Feld, A. L., O'Malley, B., Entzel, P., Smith, J. S., Gilkey, M. B., Brewer, N. T.<br>Level: 1, State: Excluded	Not about HPV vaccination/vax attitudes (eg HPV infection/serology/prevalence; cervical cancer; HPV vax safety)
777	RefID: 777, Application of the Carolina Framework for Cervical Cancer Prevention<br>Moss, J. L., McCarthy, S. H., Gilkey, M. B., Brewer, N. T.<br>Level: 1, State: Excluded	Study Design - Not qualitative (methods or analysis) OR Qualitative survey data not analyzed qualitatively (only numeric stats)
778	RefID: 778, Cervical cancer and HPV: Awareness and vaccine acceptability among parents in Morocco<br>Mouallif, M., Bowyer, H. L., Festali, S., Albert, A., Filali-Zegzouti, Y., Guenin, S., Delvenne, P., Waller, J., Ennaji, M. M.<br>Level: 1, State: Excluded	Wrong/no population (eg parents, providers, children, policy makers, does not include 18-26yrs olds; social media posts)
779	RefID: 779, WAITING ROOM PROJECT: IMPROVING HEALTHCARE of SYSTEMIC LUPUS ERYTHEMATOSUS PATIENTS<br>Moura, F., Brito, S., Amaral Mota, D., Diniz, L., MonÇão, J., Nunes, V., GonÇalves, V., Lacerda De Oliveira Campos, J., Fradico, P., Baldini, M., Hasparyk, U., Calderaro, D., Lanna, C. C., Weiss Teles, R.<br>Level: 1, State: Excluded	Not about HPV vaccination/vax attitudes (eg HPV infection/serology/prevalence; cervical cancer; HPV vax safety)
780	RefID: 780, Barriers, supports, and effective interventions for uptake of human papillomavirus- and other vaccines within global and Canadian Indigenous peoples: a systematic review protocol<br>Mrklas, K. J., MacDonald, S., Shea-Budgell, M. A., Bedingfield, N., Ganshorn, H., Glaze, S., Bill, L., Healy, B., Healy, C., Guichon, J., Colquhoun, A., Bell, C., Richardson, R., Henderson, R., Kellner, J., Barnabe, C., Bednarczyk, R. A., Letendre, A., Nelson, G. S.<br>Level: 1, State: Excluded	Wrong/no population (eg parents, providers, children, policy makers, does not include 18-26yrs olds; social media posts)
781	RefID: 781, Information channels associated with awareness of human papillomavirus infections and vaccination among Latino immigrants from safety net clinics<br>Mueller, Noel T., Noone, Anne-Michelle, Luta, Gheorghe, Wallington, Sherrie Flynt, Huerta, Elmer E., Mandelblatt, Jeanne S.<br>Level: 1, State: Excluded	Not about HPV vaccination/vax attitudes (eg HPV infection/serology/prevalence; cervical cancer; HPV vax safety)
783	RefID: 783, Impact of human papillomavirus (HPV)-6/11/16/18 vaccine on all HPV-associated genital diseases in young women<br>Muñoz, N., Kjaer, S. K., Sigurdsson, K., Iversen, O. E., Hernandez-Avila, M., Wheeler, C. M., Perez, G., Brown, D. R., Koutsky, L. A., Tay, E. H., et al.<br>Level: 1, State: Excluded	Wrong publication type (eg reviews, opinions, conference proceedings)
784	RefID: 784, Knowledge, attitudes, and demographic factors influencing cervical cancer screening behavior of Zimbabwean women<br>Mupepi, S. C., Sampselle, C. M., Johnson, T. R.<br>Level: 1, State: Excluded	Study Design - Not qualitative (methods or analysis) OR Qualitative survey data not

		analyzed qualitatively (only numeric stats)
786	RefID: 786, Acquisition, prevalence and clearance of type-specific human papillomavirus infections in young sexually active Indian women: A community-based multicentric cohort study<br>Muwonge, R., Basu, P., Gheit, T., Anantharaman, D., Verma, Y., Bhatla, N., Joshi, S., Esmy, P. O., Poli, U. R. R., Shah, A., Zomawia, E., Shastri, S. S., Pimple, S., Prabhu, P. R., Hingmire, S., Chiwate, A., SauvAge - Young adults 18-26yrs not included /outside age ranget, C., Lucas, E., Malvi, S. G., Siddiqi, M., Sankaran, S., Kannan, Tpra, Varghese, R., Divate, U., Vashist, S., Mishra, G., Jadhav, R., Tommasino, M., Pillai, M. R., Sankaranarayanan, R., Jayant, K.<br>Level: 1, State: Excluded	Not about HPV vaccination/vax attitudes (eg HPV infection/serology/prevalence; cervical cancer; HPV vax safety)
789	RefID: 789, Immunogenicity to the bivalent HPV-16/18 vaccine among adolescent african students exposed to helminths and malaria<br>Nakalembe, M., Banura, C., Namujju, P. B., Mirembe, F. M.<br>Level: 1, State: Excluded	Not about HPV vaccination/vax attitudes (eg HPV infection/serology/prevalence; cervical cancer; HPV vax safety)
790	RefID: 790, Acceptability of study procedures (self-collected introital swabs, blood draws and stool sample collection) by students 10-16 years for an HPV vaccine effectiveness study: a pilot study<br>Nakalembe, M., Mutyaba, T., Mirembe, F.<br>Level: 1, State: Excluded	Study Design - Not qualitative (methods or analysis) OR Qualitative survey data not analyzed qualitatively (only numeric stats)
791	RefID: 791, Acceptance or Rejection of the COVID-19 Vaccine: A Study on Iranian People's Opinions toward the COVID-19 Vaccine<br>Nakhostin-Ansari, A., Zimet, G. D., Khonji, M. S., Aghajani, F., Teymourzadeh, A., Rastegar Kazerooni, A. A., Pirayandeh, P., Aghajani, R., Safari, S., Khalaj, K., Memari, A. H.<br>Level: 1, State: Excluded	Not about HPV vaccination/vax attitudes (eg HPV infection/serology/prevalence; cervical cancer; HPV vax safety)
792	RefID: 792, Reported adverse events in young women following quadrivalent human papillomavirus vaccination<br>Naleway, A. L., Gold, R., Drew, L., Riedlinger, K., Henninger, M. L., Gee, J.<br>Level: 2, State: Excluded	Age - Young adults 18-26yrs not included /outside age range
793	RefID: 793, Influence of evidence type and narrative type on HPV risk perception and intention to obtain the HPV vaccine<br>Nan, X., Dahlstrom, M. F., Richards, A., Rangarajan, S.<br>Level: 2, State: Excluded	Not about HPV vaccination/vax attitudes (eg HPV infection/serology/prevalence; cervical cancer; HPV vax safety)
794	RefID: 794, Role of Narrative Perspective and Modality in the Persuasiveness of Public Service Advertisements Promoting HPV Vaccination<br>Nan, X., Futerfas, M., Ma, Z.<br>Level: 2, State: Excluded	Study Design - Not qualitative (methods or analysis) OR Qualitative survey data not analyzed qualitatively (only numeric stats)
795	RefID: 795, Awareness and Attitude towards Human Papilloma Virus Vaccine among Medical Students of a Premier Medical College, Mysuru<br>Narendran, M., Renuka, M.<br>Level: 1, State: Excluded	Not about HPV vaccination/vax attitudes (eg HPV infection/serology/prevalence; cervical cancer; HPV vax safety)
796	RefID: 796, Factors predicting intermediate endpoints of cervical cancer and exposure to human papillomavirus (HPV) infections in young women screened as potential targets for prophylactic HPV vaccination	Not about HPV vaccination/vax attitudes (eg HPV

	in south of Brazil<br>Naud, P., Matos, J., Hammes, L., Stuckzynski, J., Brouwers, K., Magno, V., Dias, E., Crusius, P., d'Avila, A., Campos, C., Costa, M., Höblik, M., Marc, C., Marroni, R., Syrjänen, K.<br>Level: 1, State: Excluded	infection/serology/prevalence; cervical cancer; HPV vax safety)
797	RefID: 797, Educational Intervention to Minimize Disparities in Humanpapillomavirus Vaccination<br>Nct<br>Level: 2, State: Excluded	Study Design - Not qualitative (methods or analysis) OR Qualitative survey data not analyzed qualitatively (only numeric stats)
798	RefID: 798, mHealth Intervention in Increasing HPV Vaccinations in College Students<br>Nct<br>Level: 1, State: Excluded	Not about HPV vaccination/vax attitudes (eg HPV infection/serology/prevalence; cervical cancer; HPV vax safety)
799	RefID: 799, Engaging Patients and Providers in Collaborative Communication on HPV Vaccination (EPICC-HPV)<br>Nct<br>Level: 1, State: Excluded	studytype
800	RefID: 800, Scientific Evaluation of One or Two Doses of the Bivalent or Nonavalent Prophylactic HPV Vaccines<br>Nct<br>Level: 1, State: Excluded	Wrong publication type (eg reviews, opinions, conference proceedings)
801	RefID: 801, PreTeenVax Evaluation<br>Nct<br>Level: 1, State: Excluded	Wrong/no population (eg parents, providers, children, policy makers, does not include 18-26yrs olds; social media posts)
802	RefID: 802, Marketability of a Technology-based Intervention to Increase HPV Vaccination<br>Nct<br>Level: 1, State: Excluded	Not about HPV vaccination/vax attitudes (eg HPV infection/serology/prevalence; cervical cancer; HPV vax safety)
803	RefID: 803, Integrated Care Delivery of HIV Prevention and Treatment in AGYW in Zambia<br>Nct<br>Level: 1, State: Excluded	Wrong/no population (eg parents, providers, children, policy makers, does not include 18-26yrs olds; social media posts)
804	RefID: 804, Promoting HPV Vaccination Among Young Adults in Texas<br>Nct<br>Level: 1, State: Excluded	Wrong publication type (eg reviews, opinions, conference proceedings)
805	RefID: 805, Evaluation of a Narrative Communication Intervention to Increase Human Papillomavirus Vaccination Intentions and Uptake<br>Nct<br>Level: 1, State: Excluded	Not about HPV vaccination/vax attitudes (eg HPV infection/serology/prevalence; cervical cancer; HPV vax safety)
806	RefID: 806, Testing MessAge - Young adults 18-26yrs not included /outside age ranges to Promote HPV Vaccination<br>Nct<br>Level: 1, State: Excluded	Not about HPV vaccination/vax attitudes (eg HPV infection/serology/prevalence; cervical cancer; HPV vax safety)
807	RefID: 807, The epidemiology of HIV and other sexually transmitted infections in African, Caribbean and Black men in Toronto, Canada<br>Nelson, L. E., Tharao, W., Husbands, W., Sa, T., Zhang, N., Kushwaha, S.,	Wrong publication type (eg reviews, opinions, conference proceedings)

	Absalom, D., Kaul, R.<br>Level: 1, State: Excluded	
808	RefID: 808, Human papillomavirus vaccine introduction in Vietnam: formative research findings<br>Nghi, N. Q., Lamontagne, D. S., Bingham, A., Rafiq, M., Mai le, T. P., Lien, N. T., Khanh, N. C., Hong, D. T., Huyen, D. T., Tho, N. T., Hien, N. T.<br>Level: 1, State: Excluded	Study Design - Not qualitative (methods or analysis) OR Qualitative survey data not analyzed qualitatively (only numeric stats)
811	RefID: 811, Parents' Recall and Reflections on Experiences Related to HPV Vaccination for Their Children<br>Niccolai, L. M., Hansen, C. E., Credle, M., Shapiro, E. D.<br>Level: 1, State: Excluded	Not about HPV vaccination/vax attitudes (eg HPV infection/serology/prevalence; cervical cancer; HPV vax safety)
812	RefID: 812, Messaging of Different Disease Outcomes for Human Papillomavirus Vaccination: A Systematic Review<br>Niccolai, L. M., Johnson, N. P., Torres, A., Sullivan, E. L., Hansen, C. E.<br>Level: 1, State: Excluded	Wrong/no population (eg parents, providers, children, policy makers, does not include 18-26yrs olds; social media posts)
813	RefID: 813, Sources of information for assessing human papillomavirus vaccination history among young women<br>Niccolai, L. M., McBride, V., Julian, P. R.<br>Level: 1, State: Excluded	Wrong/no population (eg parents, providers, children, policy makers, does not include 18-26yrs olds; social media posts)
815	RefID: 815, Individual and geographic disparities in human papillomavirus types 16/18 in high-grade cervical lesions: Associations with race, ethnicity, and poverty<br>Niccolai, L. M., Russ, C., Julian, P. J., Hariri, S., Sinard, J., Meek, J. I., McBride, V., Markowitz, L. E., Unger, E. R., Hadler, J. L., Sosa, L. E.<br>Level: 1, State: Excluded	Wrong publication type (eg reviews, opinions, conference proceedings)
816	RefID: 816, Factors Influencing the Decision to Vaccinate against HPV amongst a Wrong/no population (eg parents, providers, children, policy makers, does not include 18-26yrs olds; social media posts) of Female Health Students<br>Nicolet, L., Viviano, M., Dickson, C., Jeannot, E.<br>Level: 1, State: Excluded	Study Design - Not qualitative (methods or analysis) OR Qualitative survey data not analyzed qualitatively (only numeric stats)
817	RefID: 817, Hpv-specific systemic antibody responses and memory b cells are independently maintained up to 6 years and in a vaccine-specific manner following immunization with cervarix and gardasil in adolescent and young adult women in vaccination programs in Italy<br>Nicoli, F., Mantelli, B., Gallerani, E., Telatin, V., Bonazzi, I., Marconi, P., Gavioli, R., Gabrielli, L., Lazzarotto, T., Barzon, L., Palù, G., Caputo, A.<br>Level: 1, State: Excluded	Not about HPV vaccination/vax attitudes (eg HPV infection/serology/prevalence; cervical cancer; HPV vax safety)
822	RefID: 822, Cytology role in healthy sexual life promotion of adolescents: Users of centro de atendimento a jovens de Coimbra<br>Nobre, S., Tomé, M. T.<br>Level: 1, State: Excluded	Not about HPV vaccination/vax attitudes (eg HPV infection/serology/prevalence; cervical cancer; HPV vax safety)
823	RefID: 823, Challenges to implementing an HPV education and vaccination program in new Mexico<br>Nodulman, J. A., Kong, A. S., Starling, R., Bryan, A. D., Romero, J., Wheeler, C. M., Buller, D. B., Woodall, W. G.<br>Level: 1, State: Excluded	Age - Young adults 18-26yrs not included /outside age range
824	RefID: 824, Letter to the editor: Human papillomavirus vaccine uptake among Asian American women Age - Young adults 18-26yrs not	Study Design - Not qualitative (methods or analysis) OR

	included /outside age ranged 18 to 26 years: Behavioral risk factor surveillance system (BRFSS) study, 2008-2010<br>Nomura, K., Rahman, M.<br>Level: 1, State: Excluded	Qualitative survey data not analyzed qualitatively (only numeric stats)
825	RefID: 825, Prevalence and factors associated with coinfection of human papillomavirus and Chlamydia trachomatis in adolescents and young women<br>Nonato, D. R., Alves, R. R., Ribeiro, A. A., Saddi, V. A., Segati, K. D., Almeida, K. P., de Lima, Y. A., D'Alessandro, W. B., Rabelo-Santos, S. H.<br>Level: 1, State: Excluded	Not about HPV vaccination/vax attitudes (eg HPV infection/serology/prevalence; cervical cancer; HPV vax safety)
828	RefID: 828, Primary and secondary prevention of cervical cancer among ethnically diverse and low-income Wrong/no population (eg parents, providers, children, policy makers, does not include 18-26yrs olds; social media posts)s<br>Nonzee, Narissa Jennifer<br>Level: 1, State: Excluded	Wrong publication type (eg reviews, opinions, conference proceedings)
829	RefID: 829, Sexual and reproductive health needs for young women living with perinatally acquired HIV through the pandemic<br>Nott, V., Hazell, G., Ayres, S., Kirkhope, N., Fidler, S., Foster, C.<br>Level: 1, State: Excluded	Not about HPV vaccination/vax attitudes (eg HPV infection/serology/prevalence; cervical cancer; HPV vax safety)
830	RefID: 830, Knowledge, attitudes and practices of cervical cancer prevention among Zambian women and men<br>Nyambe, A., Kampen, J. K., Baboo, S. K., Van Hal, G.<br>Level: 1, State: Excluded	Wrong publication type (eg reviews, opinions, conference proceedings)
831	RefID: 831, The uptake of adolescent vaccinations through the School Immunisation Program in specialist schools in Victoria, Australia<br>O'Neill, J., Newall, F., Antolovich, G., Lima, S., Danchin, M.<br>Level: 1, State: Excluded	Not about HPV vaccination/vax attitudes (eg HPV infection/serology/prevalence; cervical cancer; HPV vax safety)
832	RefID: 832, Estimation of utility weights for human papilloma virus-related health states according to disease severity<br>Ock, M., Park, J. Y., Son, W. S., Lee, H. J., Kim, S. H., Jo, M. W.<br>Level: 1, State: Excluded	Not about HPV vaccination/vax attitudes (eg HPV infection/serology/prevalence; cervical cancer; HPV vax safety)
833	RefID: 833, Gender, sex and equal health: school nurses' strategies and experiences of including boys in the HPV vaccination programme in Swedish primary schools<br>Odenbring, Y., Lindén, L.<br>Level: 1, State: Excluded	Not about HPV vaccination/vax attitudes (eg HPV infection/serology/prevalence; cervical cancer; HPV vax safety)
834	RefID: 834, Knowledge and Attitude of Female Students of Tertiary Institutions in Imo State, Nigeria Towards Cervical Cancer and Its Screening<br>Ogwunga, C. C., Anyadoh-Nwadike, S. O., Ahumibe, N. C., Nwakwasi, E. U.<br>Level: 1, State: Excluded	Not about HPV vaccination/vax attitudes (eg HPV infection/serology/prevalence; cervical cancer; HPV vax safety)
835	RefID: 835, Awareness of and attitude towards human papillomavirus infection and vaccination for cervical cancer prevention among adult males and females in Korea: a nationwide interview survey<br>Oh, J. K., Lim, M. K., Yun, E. H., Lee, E. H., Shin, H. R.<br>Level: 1, State: Excluded	Wrong/no population (eg parents, providers, children, policy makers, does not include 18-26yrs olds; social media posts)

836	RefID: 836, Awareness of and practice toward cancer prevention recommendations: results of the Korean National Cancer Prevention Awareness and Practice Survey in 2021<br>Oh, J. K., Park, E., Kim, B., Choi, Y. J., Yun, E. H., Lim, M. K., Im, J. S., Park, E. Y.<br>Level: 1, State: Excluded	Not about HPV vaccination/vax attitudes (eg HPV infection/serology/prevalence; cervical cancer; HPV vax safety)
837	RefID: 837, Racial Disparities in HPV-related Knowledge, Attitudes, and Beliefs Among African American and White Women in the USA<br>Ojeaga, A., Alema-Mensah, E., Rivers, D., Azonobi, I., Rivers, B.<br>Level: 1, State: Excluded	Study Design - Not qualitative (methods or analysis) OR Qualitative survey data not analyzed qualitatively (only numeric stats)
838	RefID: 838, Prevalence and distribution of cervical high-risk human papillomavirus infection in a rural community of Edo State, Nigeria<br>Okoeguale, J., Samuel, S. O., Amadi, S. C., Njoku, A., Okome, G. B. O.<br>Level: 1, State: Excluded	Not about HPV vaccination/vax attitudes (eg HPV infection/serology/prevalence; cervical cancer; HPV vax safety)
839	RefID: 839, Intervention studies to encourAge - Young adults 18-26yrs not included /outside age range HPV vaccination using narrative: A scoping review<br>Okuhara, T., Kagawa, Y., Okada, H., Tsunezumi, A., Kiuchi, T.<br>Level: 1, State: Excluded	Study Design - Not qualitative (methods or analysis) OR Qualitative survey data not analyzed qualitatively (only numeric stats)
840	RefID: 840, Cervical cancer risk factors in eight west African countries: cross-sectional analysis of the demographic and health survey 2017-20<br>Olajide, N., Robb, K., Niedzwiedz, C., Jani, B.<br>Level: 1, State: Excluded	Not about HPV vaccination/vax attitudes (eg HPV infection/serology/prevalence; cervical cancer; HPV vax safety)
841	RefID: 841, Knowledge and acceptability of HPV vaccine among HPV-vaccinated and unvaccinated adolescents at Western Amazon<br>Oliveira, M. S. F., Sorpreso, I. C. E., Zuchelo, L. T. S., Silva, Atmd, Gomes, J. M., Silva, B. K. R., Abreu, L. C., Wajnsztejn, R.<br>Level: 1, State: Excluded	Wrong publication type (eg reviews, opinions, conference proceedings)
842	RefID: 842, Knowledge, attitude and practice of cervical cancer prevention, among women residing in an urban slum in Lagos, South West, Nigeria<br>Olubodun, T., Odukoya, O. O., Balogun, M. R.<br>Level: 1, State: Excluded	Not about HPV vaccination/vax attitudes (eg HPV infection/serology/prevalence; cervical cancer; HPV vax safety)
843	RefID: 843, Application of the theoretical domains framework to identify factors influencing catch-up HPV vaccinations among male college students in the United States: A review of evidence and recommendations<br>Olusanya, O. A., Tomar, A., Thomas, J., Alonge, K., Wigfall, L. T.<br>Level: 1, State: Excluded	Age - Young adults 18-26yrs not included /outside age range
844	RefID: 844, Healthcare professionals' perceptions and recommendations regarding adolescent vaccinations in Georgia and Tennessee during the COVID-19 pandemic: A qualitative research<br>Olusanya, O. A., White, B., Malik, F., Hester, K. A., Davis, R. L., Bednarczyk, R. A., Shaban-Nejad, A.<br>Level: 1, State: Excluded	Study Design - Not qualitative (methods or analysis) OR Qualitative survey data not analyzed qualitatively (only numeric stats)
845	RefID: 845, EPH78 Beliefs Regarding HPV Vaccination Among Young Adult College Students: A Qualitative Study<br>Orji, C., Brown, C. M., Barner, J. C., Moczygemba, L., Morales-Campos,	Wrong publication type (eg reviews, opinions, conference proceedings)

	D., Nghiem, C.<br>Level: 2, State: Excluded	
846	RefID: 846, Knowledge, attitude and practice of school nurses in the United Arab Emirates about HPV infection and vaccine<br>Ortashi, O., Shallal, M., Osman, N., Raheel, H.<br>Level: 1, State: Excluded	Wrong/no population (eg parents, providers, children, policy makers, does not include 18-26yrs olds; social media posts)
847	RefID: 847, Prevalence, genotyping, and correlates of anogenital HPV infection in a Wrong/no population (eg parents, providers, children, policy makers, does not include 18-26yrs olds; social media posts)-based sample of women in Puerto Rico<br>Ortiz, A. P., Romaguera, J., Pérez, C. M., González, D., Muñoz, C., González, L., Marrero, E., Tortolero-Luna, G., Suárez, E., Palefsky, J.<br>Level: 1, State: Excluded	studytype
848	RefID: 848, Seroprevalence of HPV 6, 11, 16 and 18 and correlates of exposure in unvaccinated women Age - Young adults 18-26yrs not included /outside age ranged 16-64 years in Puerto Rico<br>Ortiz, A. P., Tortolero-Luna, G., Romaguera, J., Pérez, C. M., González, D., Muñoz, C., González, L., Marrero, E., Suárez, E., Palefsky, J. M., Panicker, G., Unger, E. R.<br>Level: 1, State: Excluded	Study Design - Not qualitative (methods or analysis) OR Qualitative survey data not analyzed qualitatively (only numeric stats)
850	RefID: 850, Assessing feasibility and strategies for clinicians to communicate via social media with adolescent patients about HPV vaccination<br>Ortiz, R., Shafer, A., Cates, J., Coyne-Beasley, T.<br>Level: 1, State: Excluded	Not about HPV vaccination/vax attitudes (eg HPV infection/serology/prevalence; cervical cancer; HPV vax safety)
851	RefID: 851, Development and Evaluation of a Social Media Health Intervention to Improve Adolescents' Knowledge About and Vaccination Against the Human Papillomavirus<br>Ortiz, R. R., Shafer, A., Cates, J., Coyne-Beasley, T.<br>Level: 1, State: Excluded	Age - Young adults 18-26yrs not included /outside age range
852	RefID: 852, No change in physician discussions with patients about the human papillomavirus vaccine between 2007 and 2013<br>Osazuwa-Peters, N., López, J., Rice, S., Tutlam, N., Tokarz, S., Varvares, M. A.<br>Level: 1, State: Excluded	Not about HPV vaccination/vax attitudes (eg HPV infection/serology/prevalence; cervical cancer; HPV vax safety)
853	RefID: 853, Sociodemographic predictors of HPV and HPV vaccine knowledge and awareness among Americans who use the internet as their primary source of health information<br>Osazuwa-Peters, N., Tobo, B. B., Gordon, R. M., Boakye, E. A.<br>Level: 1, State: Excluded	Not about HPV vaccination/vax attitudes (eg HPV infection/serology/prevalence; cervical cancer; HPV vax safety)
854	RefID: 854, Midwives at youth clinics attitude to HPV vaccination and their role in cervical cancer prevention<br>Oscarsson, M. G., Dahlberg, A., Tydén, T.<br>Level: 1, State: Excluded	Not about HPV vaccination/vax attitudes (eg HPV infection/serology/prevalence; cervical cancer; HPV vax safety)
855	RefID: 855, Young women’s decision-making process for HPV vaccination<br>Oscarsson, Marie G., Hannerfors, Anna-Karin, Tydén, Tanja<br>Level: 2, State: Excluded	Study Design - Not qualitative (methods or analysis) OR Qualitative survey data not analyzed qualitatively (only numeric stats)

857	RefID: 857, [SUS users' knowledge of and attitude to HPV virus and vaccines available in Brazil]<br>Osis, M. J., Duarte, G. A., Sousa, M. H.<br>Level: 1, State: Excluded	Wrong/no population (eg parents, providers, children, policy makers, does not include 18-26yrs olds; social media posts)
859	RefID: 859, Prevalence of anal dysplasia and HPV genotypes in gynecology patients: The ANGY cross-sectional prospective clinical study protocol<br>Pache, B., Balaya, V., Mathis, J., Hübner, M., Sahli, R., Cavassini, M., Sempoux, C., Mathevet, P., Jacot-Guillarmod, M.<br>Level: 1, State: Excluded	Multiple Wrong/no population (eg parents, providers, children, policy makers, does not include 18-26yrs olds; social media posts)s - unable to extract data specific to young adults (18-26 yrs)
860	RefID: 860, College graduation reduces vulnerability to STIs/HIV among African-American young adult women<br>Painter, J. E., Wingood, G. M., DiClemente, R. J., Depadilla, L. M., Simpson-Robinson, L.<br>Level: 1, State: Excluded	Study Design - Not qualitative (methods or analysis) OR Qualitative survey data not analyzed qualitatively (only numeric stats)
861	RefID: 861, [Social considerations affecting acceptance of HPV vaccination in Colombia. A systematic review]<br>Palencia-Sánchez, F., Echeverry-Coral, S. J.<br>Level: 1, State: Excluded	Not about HPV vaccination/vax attitudes (eg HPV infection/serology/prevalence; cervical cancer; HPV vax safety)
862	RefID: 862, HPV immunisation and increased uptake of cervical screening in Scottish women; observational study of routinely collected national data<br>Palmer, T. J., McFadden, M., Pollock, K. G., Kavanagh, K., Cuschieri, K., Cruickshank, M., Nicoll, S., Robertson, C.<br>Level: 1, State: Excluded	Not about HPV vaccination/vax attitudes (eg HPV infection/serology/prevalence; cervical cancer; HPV vax safety)
864	RefID: 864, Short-Term Efficacy of CBD-Enriched Hemp Oil in Girls with Dysautonomic Syndrome after Human Papillomavirus Vaccination<br>Palmieri, B., Laurino, C., Vadalà, M.<br>Level: 1, State: Excluded	Wrong publication type (eg reviews, opinions, conference proceedings)
865	RefID: 865, Effect of bivalent human papillomavirus vaccination on pregnancy outcomes: long term observational follow-up in the Costa Rica HPV Vaccine Trial<br>Panagiotou, O. A., Befano, B. L., Gonzalez, P., Rodríguez, A. C., Herrero, R., Schiller, J. T., Kreimer, A. R., Schiffman, M., Hildesheim, A., Wilcox, A. J., et al.<br>Level: 1, State: Excluded	Study Design - Not qualitative (methods or analysis) OR Qualitative survey data not analyzed qualitatively (only numeric stats)
869	RefID: 869, Vaccination against human papillomavirus among 865 female students from the health professions in central Greece: a questionnaire-based cross-sectional study<br>Papagiannis, D., Rachiotis, G., Symvoulakis, E. K., Daponte, A., Grivea, I. N., Syrogiannopoulos, G. A., Hadjichristodoulou, C.<br>Level: 2, State: Excluded	Not about HPV vaccination/vax attitudes (eg HPV infection/serology/prevalence; cervical cancer; HPV vax safety)
870	RefID: 870, Knowledge and awareness regarding cervical cancer with respect to risk factors, screening and vaccination<br>Paranjpe, M., Wani, R. J.<br>Level: 1, State: Excluded	Study Design - Not qualitative (methods or analysis) OR Qualitative survey data not analyzed qualitatively (only numeric stats)
871	RefID: 871, Increases in Human Papillomavirus Vaccination among Adolescent and Young Adult Males in the United States, 2011-2016	Study Design - Not qualitative (methods or analysis) OR

	Patel, E. U., Grabowski, M. K., Eisenberg, A. L., Packman, Z. R., Gravitt, P. E., Tobian, A. A. R.<br>Level: 1, State: Excluded	Qualitative survey data not analyzed qualitatively (only numeric stats)
873	RefID: 873, Pilot Study: A Novel Method for Cervical Health Monitoring in African American Women with Systemic Lupus Erythematosus (SLE) Using a Self-Sampling Brush to Assess Cervical HPV Infection and Cervical Cytology<br>Patricia Dhar, J., Walline, H., Fathallah, L., Szpunar, S., Saravolatz, L., Mor, G., Carey, T.<br>Level: 1, State: Excluded	Study Design - Not qualitative (methods or analysis) OR Qualitative survey data not analyzed qualitatively (only numeric stats)
874	RefID: 874, "Saving lives": Adapting and adopting Human Papilloma Virus (HPV) vaccination in Austria<br>Paul, K. T.<br>Level: 2, State: Excluded	Not about HPV vaccination/vax attitudes (eg HPV infection/serology/prevalence; cervical cancer; HPV vax safety)
876	RefID: 876, Acceptability of HPV vaccine implementation among parents in India<br>Paul, P., Tanner, A. E., Gravitt, P. E., Vijayaraghavan, K., Shah, K. V., Zimet, G. D., Study Group, C.<br>Level: 1, State: Excluded	Not about HPV vaccination/vax attitudes (eg HPV infection/serology/prevalence; cervical cancer; HPV vax safety)
877	RefID: 877, Conversational replies to oral queries in gynecologic oncology by Google, Alexa and Siri<br>Pavlik, E. J., Burgess, B. T., Quick, K., McDowell, A. B., Gorski, J. W., Riggs, M. B., Baldwin, L. A., Miller, R. W., Desimone, C. P., Dietrich, C. S., Gallion, H. H., Ueland, F. R., VannAge - Young adults 18-26yrs not included /outside age rangell, J. R.<br>Level: 1, State: Excluded	Not about HPV vaccination/vax attitudes (eg HPV infection/serology/prevalence; cervical cancer; HPV vax safety)
878	RefID: 878, Receipt of Selected Preventive Health Services for Women and Men of Reproductive Age - Young adults 18-26yrs not included /outside age range - United States, 2011-2013<br>Pazol, K., Robbins, C. L., Black, L. I., Ahrens, K. A., Daniels, K., Chandra, A., Vahratian, A., Gavin, L. E.<br>Level: 1, State: Excluded	Wrong/no population (eg parents, providers, children, policy makers, does not include 18-26yrs olds; social media posts)
879	RefID: 879, Human papillomavirus infection and seroprevalence among female university students in Mexico<br>Pedroza-Gonzalez, A., Reyes-Reali, J., Campos-Solorzano, M., Blancas-Diaz, E. M., Tomas-Morales, J. A., Hernandez-Aparicio, A. A., Montes de Oca-Samperio, D., Garrido, E., Garcia-Romo, G. S., Mendez-Catala, C. F., Alvarez Ortiz, P., Sánchez Ramos, J., Mendoza-Ramos, M. I., Saucedo-Campos, A. D., Pozo-Molina, G.<br>Level: 1, State: Excluded	Not about HPV vaccination/vax attitudes (eg HPV infection/serology/prevalence; cervical cancer; HPV vax safety)
880	RefID: 880, Understanding How Adolescents Think about the HPV Vaccine<br>Pennella, R. A., Ayers, K. A., Brandt, H. M.<br>Level: 1, State: Excluded	Not about HPV vaccination/vax attitudes (eg HPV infection/serology/prevalence; cervical cancer; HPV vax safety)
881	RefID: 881, Factors associated with HPV vaccination coverAge - Young adults 18-26yrs not included /outside age range at school level during HPV vaccine introduction in Thailand<br>Pensuk, P., Wihantong, N., Klinsupa, W., Wechakul, L., Pankhun, S., Jiamsiri, S.<br>Level: 1, State: Excluded	Not about HPV vaccination/vax attitudes (eg HPV infection/serology/prevalence; cervical cancer; HPV vax safety)

882	RefID: 882, Nativity Disparities in Human Papillomavirus Vaccination Among U.S. Adults<br>Pérez, A. E., Agénor, M., Gamarel, K. E., Operario, D.<br>Level: 1, State: Excluded	Wrong/no population (eg parents, providers, children, policy makers, does not include 18-26yrs olds; social media posts)
883	RefID: 883, Giving Boys a Shot: The HPV Vaccine's Portrayal in Canadian Newspapers<br>Perez, S., Fedoruk, C., Shapiro, G. K., Rosberger, Z.<br>Level: 1, State: Excluded	Not about HPV vaccination/vax attitudes (eg HPV infection/serology/prevalence; cervical cancer; HPV vax safety)
884	RefID: 884, What affects human papillomavirus vaccination rates? A qualitative analysis of providers' perceptions<br>Perkins, R. B., Clark, J. A.<br>Level: 1, State: Excluded	Age - Young adults 18-26yrs not included /outside age range
885	RefID: 885, Providers' attitudes toward human papillomavirus vaccination in young men: challenges for implementation of 2011 recommendations<br>Perkins, R. B., Clark, J. A.<br>Level: 1, State: Excluded	Wrong/no population (eg parents, providers, children, policy makers, does not include 18-26yrs olds; social media posts)
886	RefID: 886, Effectiveness of a provider-focused intervention to improve HPV vaccination rates in boys and girls<br>Perkins, R. B., Zisblatt, L., Legler, A., Trucks, E., Hanchate, A., Gorin, S. S.<br>Level: 1, State: Excluded	Wrong/no population (eg parents, providers, children, policy makers, does not include 18-26yrs olds; social media posts)
887	RefID: 887, Barriers and facilitators to HPV vaccine uptake among US rural Wrong/no population (eg parents, providers, children, policy makers, does not include 18-26yrs olds; social media posts)s: a scoping review<br>Peterson, C. E., Silva, A., Holt, H. K., Balanean, A., Goben, A. H., Dykens, J. A.<br>Level: 1, State: Excluded	Wrong/no population (eg parents, providers, children, policy makers, does not include 18-26yrs olds; social media posts)
888	RefID: 888, An investigation of three injections techniques in reducing local injection pain with a human papillomavirus vaccine: a randomized trial<br>Petousis-Harris, H., Poole, T., Stewart, J., Turner, N., Goodyear-Smith, F., Coster, G., Lennon, D.<br>Level: 1, State: Excluded	Study Design - Not qualitative (methods or analysis) OR Qualitative survey data not analyzed qualitatively (only numeric stats)
889	RefID: 889, Cervical cancer and HPV: knowledge, attitudes, beliefs, and behaviors among women living in Guatemala<br>Petrocy, A., Katz, M. L.<br>Level: 1, State: Excluded	Wrong publication type (eg reviews, opinions, conference proceedings)
892	RefID: 892, Incidence and Persistence of High-risk Anogenital Human Papillomavirus Infection Among Female Youth With and Without Perinatally Acquired Human Immunodefiency Virus Infection: A 3-year Observational Cohort Study<br>Phanuphak, N., Teeraananchai, S., Hansudewechakul, R., Gatechompol, S., Chokephaibulkit, K., Dang, H. L. D., Tran, D. N. H., Achalapong, J., Teeratakulpisarn, N., Chalermchockcharoenkit, A., Thamkhantho, M., Pankam, T., Singtoroj, T., Termrungruanglert, W., Chaithongwongwatthana, S., Kerr, S. J., Sohn, A. H.<br>Level: 1, State: Excluded	Not about HPV vaccination/vax attitudes (eg HPV infection/serology/prevalence; cervical cancer; HPV vax safety)

894	RefID: 894, Nephrologist or primary care physician? Immunization of patients receiving maintenance hemodialysis<br>Phen, S., Sattari, M., Ozrazgat-Baslanti, T., Bozorgmehri, S., Kazory, A.<br>Level: 1, State: Excluded	Study Design - Not qualitative (methods or analysis) OR Qualitative survey data not analyzed qualitatively (only numeric stats)
895	RefID: 895, Sexual and reproductive health education: contrasting teachers', health partners' and former students' perspectives<br>Phillips, K. P., Martinez, A.<br>Level: 1, State: Excluded	Age - Young adults 18-26yrs not included /outside age range
897	RefID: 897, Knowledge of Cervical Cancer and Human Papillomavirus Vaccines among Child-Bearing Age - Young adults 18-26yrs not included /outside age ranged Women in Hanoi, Vietnam<br>Phuong, N. T. N., Xuan, L. T. T., Huong, L. T., Toan, D. T. T., Oh, J. K., Won, Y. J., Choi, K. S.<br>Level: 1, State: Excluded	Not about HPV vaccination/vax attitudes (eg HPV infection/serology/prevalence; cervical cancer; HPV vax safety)
898	RefID: 898, [Prevalence of Chlamydia trachomatis and Neisseria gonorrhoea infections in sexual actives young women at a southern Brazilian city]<br>Piazzetta, R. C., de Carvalho, N. S., de Andrade, R. P., Piazzetta, G., Piazzetta, S. R., Carneiro, R.<br>Level: 1, State: Excluded	Not about HPV vaccination/vax attitudes (eg HPV infection/serology/prevalence; cervical cancer; HPV vax safety)
902	RefID: 902, Role of child's gender on acceptance of the human papillomavirus (HPV) vaccine among a high-risk sample of Haitian and African-American parents of adolescent sons and daughters<br>Pierre-Joseph, N., Walsh, J., Porte, C., Belizaire, M., Perkins, R.<br>Level: 1, State: Excluded	Study Design - Not qualitative (methods or analysis) OR Qualitative survey data not analyzed qualitatively (only numeric stats)
903	RefID: 903, Conversations about sexual activity within Haitian families: implications for HPV vaccine uptake<br>Pierre-Victor, D., Stephens, D., Gabbidon, K., Jean-Baptiste, N., Clarke, R., Madhivanan, P.<br>Level: 2, State: Excluded	Not about HPV vaccination/vax attitudes (eg HPV infection/serology/prevalence; cervical cancer; HPV vax safety)
904	RefID: 904, Health care providers' role in HPV vaccine uptake among young haitian women<br>Pierre-Victor, D., Stephens, D., Gabbidon, K., Madhivanan, P., Clarke, R.<br>Level: 2, State: Excluded	Wrong/no population (eg parents, providers, children, policy makers, does not include 18-26yrs olds; social media posts)
906	RefID: 906, Human papillomavirus type distribution in cervical intraepithelial neoplasia grade 2/3 and cervical cancer in Portugal: a CLEOPATRE II Study<br>Pista, A., de Oliveira, C. F., Lopes, C., Cunha, M. J.<br>Level: 1, State: Excluded	Multiple Wrong/no population (eg parents, providers, children, policy makers, does not include 18-26yrs olds; social media posts)s - unable to extract data specific to young adults (18-26 yrs)
907	RefID: 907, Singaporean women's knowledge of human papillomavirus (HPV) and attitudes toward HPV vaccination<br>Pitts, M., Smith, A., Croy, S., Lyons, A., Ryall, R., Garland, S., Wong, M. L., Tay, E. H.<br>Level: 2, State: Excluded	studytype
908	RefID: 908, College Males' Enduring and Novel Health Beliefs about the HPV Vaccine	Not about HPV vaccination/vax attitudes (eg HPV

	Pitts, M. J., Stanley, S. J., Kim, S.<br>Level: 2, State: Excluded	infection/serology/prevalence; cervical cancer; HPV vax safety)
909	RefID: 909, Knowledge of human papillomavirus (HPV) and the HPV vaccine in a national sample of Australian men and women<br>Pitts, M. K., Heywood, W., Ryall, R., Smith, A. M., Shelley, J. M., Richters, J., Simpson, J. M.<br>Level: 1, State: Excluded	Study Design - Not qualitative (methods or analysis) OR Qualitative survey data not analyzed qualitatively (only numeric stats)
910	RefID: 910, Lesbian women and knowledge about human papillomavirus<br>Polek, C., Hardie, T.<br>Level: 1, State: Excluded	Multiple Wrong/no population (eg parents, providers, children, policy makers, does not include 18-26yrs olds; social media posts)s - unable to extract data specific to young adults (18-26 yrs)
911	RefID: 911, A cross-sectional study to assess HPV knowledge and HPV vaccine acceptability in Mali<br>Poole, D. N., Tracy, J. K., Levitz, L., Rochas, M., Sangare, K., Yekta, S., Tounkara, K., Aboubacar, B., Koita, O., Lurie, M., De Groot, A. S.<br>Level: 1, State: Excluded	Study Design - Not qualitative (methods or analysis) OR Qualitative survey data not analyzed qualitatively (only numeric stats)
912	RefID: 912, Locating Purity within Corruption Rumors: Narratives of HPV Vaccination Refusal in a Peri-urban Community of Southern Romania<br>Pop, C. A.<br>Level: 1, State: Excluded	Study Design - Not qualitative (methods or analysis) OR Qualitative survey data not analyzed qualitatively (only numeric stats)
913	RefID: 913, Adolescents, knowledge of, beliefs about and attitudes to the human papilloma virus vaccine in the Valencian Community<br>Portero-Alonso, A., Alguacil-Ramos, A. M., Martín-Ivorra, R., Pastor-Villalba, E., Lluch-Rodrigo, J. A.<br>Level: 1, State: Excluded	Age - Young adults 18-26yrs not included /outside age range
914	RefID: 914, Barriers and facilitators to HPV vaccination: perspectives from Malawian women<br>Ports, K. A., Reddy, D. M., Rameshbabu, A.<br>Level: 1, State: Excluded	Wrong/no population (eg parents, providers, children, policy makers, does not include 18-26yrs olds; social media posts)
915	RefID: 915, Mothers' preferences and willingness to pay for HPV vaccines in Vinh Long Province, Vietnam<br>Poulos, Christine, Yang, Jui-Chen, Levin, Carol, Van Minh, Hoang, Giang, Kim Bao, Nguyen, Diep<br>Level: 1, State: Excluded	Study Design - Not qualitative (methods or analysis) OR Qualitative survey data not analyzed qualitatively (only numeric stats)
917	RefID: 917, Role of insurance, income, and affordability in human papillomavirus vaccination<br>Pourat, N., Jones, J. M.<br>Level: 1, State: Excluded	Wrong/no population (eg parents, providers, children, policy makers, does not include 18-26yrs olds; social media posts)
918	RefID: 918, Cervical cancers associated with human papillomavirus types 16, 18 and 45 are diagnosed in younger women than cancers associated with other types: a cross-sectional observational study in Wales and Scotland (UK)<br>Powell, N., Cuschieri, K., Cubie, H., Hibbitts, S., Rosillon, D., De Souza, S. C., Molijn, A., Quint, W., Holl, K., Fiander, A.<br>Level: 1, State: Excluded	Wrong/no population (eg parents, providers, children, policy makers, does not include 18-26yrs olds; social media posts)

920	RefID: 920, HPV vaccination uptake and administration from 2006 to 2016 in a commercially insured Wrong/no population (eg parents, providers, children, policy makers, does not include 18-26yrs olds; social media posts) of the United States<br>Prabhu, V. S., Bansal, N., Liu, Z., Finalle, R., Sénécal, M., Kothari, S., Trowers, K., Myers, E.<br>Level: 1, State: Excluded	Study Design - Not qualitative (methods or analysis) OR Qualitative survey data not analyzed qualitatively (only numeric stats)
924	RefID: 924, Knowledge, attitude and practice (KAP) of cervical cancer and hpv vaccine among pharmacy and paramedical female students at a private university (South India)<br>Priya, S., Ashok Kumar, M.<br>Level: 1, State: Excluded	Not about HPV vaccination/vax attitudes (eg HPV infection/serology/prevalence; cervical cancer; HPV vax safety)
928	RefID: 928, Investigate the sexual habits of young people: a cross-sectional study among nursing students of the University of Palermo<br>Provenzano, S., Santangelo, O. E., Terranova, A., D'Anna, G., Grigis, D., Firenze, A.<br>Level: 1, State: Excluded	Not about HPV vaccination/vax attitudes (eg HPV infection/serology/prevalence; cervical cancer; HPV vax safety)
930	RefID: 930, Exploring views regarding targeted HPV vaccination in young men who have sex with men and their healthcare professionals<br>Prue, G., Flannagan, C., Kesten, J., Merriel, S., Shapiro, G., Rosberger, Z.<br>Level: 2, State: Excluded	Not about HPV vaccination/vax attitudes (eg HPV infection/serology/prevalence; cervical cancer; HPV vax safety)
931	RefID: 931, The effect of public information about human papillomavirus (HPV) vaccine on knowledge, attitude and vaccination decision among women in Thailand<br>Putchong, C., Sirisamutr, T., Ktitanan, W., Udomsook, K., Tantivess, S., Teerawattananon, Y.<br>Level: 1, State: Excluded	Study Design - Not qualitative (methods or analysis) OR Qualitative survey data not analyzed qualitatively (only numeric stats)
932	RefID: 932, Influence of an educational video to improve Vaccination against human papillomavirus<br>qm3x, R. B. R.<br>Level: 1, State: Excluded	studytype
933	RefID: 933, A national survey about human papillomavirus vaccination: what we didn't ask, but physicians wanted us to know<br>Quinn, G. P., Murphy, D., Malo, T. L., Christie, J., Vadaparampil, S. T.<br>Level: 1, State: Excluded	Study Design - Not qualitative (methods or analysis) OR Qualitative survey data not analyzed qualitatively (only numeric stats)
934	RefID: 934, HPV.edu study protocol: a cluster randomised controlled evaluation of education, decisional support and logistical strategies in school-based human papillomavirus (HPV) vaccination of adolescents<br>Rachel Skinner, S., Davies, Cristyn, Cooper, Spring, Stoney, Tanya, Marshall, Helen, Jones, Jane, Collins, Joanne, Hutton, Heidi, Parrella, Adriana, Zimet, Gregory, Regan, David G., Whyte, Patti, Brotherton, Julia M. L., Richmond, Peter, McCaffrey, Kirsten, Garland, Suzanne M., Leask, Julie, Kang, Melissa, Braunack-Mayer, Annette, Kaldor, John<br>Level: 1, State: Excluded	Wrong/no population (eg parents, providers, children, policy makers, does not include 18-26yrs olds; social media posts)
935	RefID: 935, 'HPV? Never heard of it': students and the HPV vaccine<br>Racktoo, S., Coverdale, G.<br>Level: 1, State: Excluded	Wrong/no population (eg parents, providers, children, policy makers, does not include 18-26yrs olds; social media posts)

936	RefID: 936, Missed opportunities for catch-up human papillomavirus vaccination among university undergraduates: Identifying health decision-making behaviors and uptake barriers<br>Ragan, K. R., Bednarczyk, R. A., Butler, S. M., Omer, S. B.<br>Level: 1, State: Excluded	Wrong publication type (eg reviews, opinions, conference proceedings)
937	RefID: 937, HPV vaccination discourses and the construction of "at-risk" girls<br>Rail, G., Molino, L., Fusco, C., Norman, M. E., Petherick, L., Polzer, J., Moola, F., Bryson, M.<br>Level: 2, State: Excluded	Age - Young adults 18-26yrs not included /outside age range
938	RefID: 938, Awareness and knowledge of HPV, cervical cancer, and vaccines in young women after first delivery in São Paulo, Brazil--a cross-sectional study<br>Rama, C. H., Villa, L. L., Pagliusi, S., Andreoli, M. A., Costa, M. C., Aoki, A. L., Longatto-Filho, A., Eluf-Neto, J.<br>Level: 1, State: Excluded	Study Design - Not qualitative (methods or analysis) OR Qualitative survey data not analyzed qualitatively (only numeric stats)
940	RefID: 940, Self-reported barriers and facilitators to preventive human papillomavirus vaccination among adolescent girls and young women: A systematic review<br>Rambout, Lisa, Tashkandi, Mariam, Hopkins, Laura, Tricco, Andrea C.<br>Level: 1, State: Excluded	Multiple Wrong/no population (eg parents, providers, children, policy makers, does not include 18-26yrs olds; social media posts)s - unable to extract data specific to young adults (18-26 yrs)
941	RefID: 941, Human papillomavirus prevalence in male and female university students in Gaborone, Botswana<br>Ramogola-Masire, D., McClung, N., Mathoma, A., Gargano, J. W., Nyepetsi, N. G., Querec, T. D., Onyekwuluje, J., Mine, M., Morroni, C., Luckett, R., Markowitz, L. E.<br>Level: 1, State: Excluded	Not about HPV vaccination/vax attitudes (eg HPV infection/serology/prevalence; cervical cancer; HPV vax safety)
942	RefID: 942, Examining cervical cancer preventive behaviors for latinx transmasculine individuals among medical students<br>Ramos-Pibernus, A., Carminelli-Corretjer, P., Bermonti-Pérez, M., Tollinchi-Natali, N., Jiménez-Ricaurte, C., Mejías-Serrano, D., Silva-Reteguis, J., Moreta-ávila, F., Blanco, M., Justiz, L., Febo, M., Rivera-Segarra, E.<br>Level: 1, State: Excluded	Wrong publication type (eg reviews, opinions, conference proceedings)
943	RefID: 943, Patient-provider communication and human papillomavirus vaccine acceptance<br>Rand, C. M., Schaffer, S. J., Humiston, S. G., Albertin, C. S., Shone, L. P., Heintz, E. V., Blumkin, A. K., Stokley, S., Szilagyi, P. G.<br>Level: 1, State: Excluded	Not about HPV vaccination/vax attitudes (eg HPV infection/serology/prevalence; cervical cancer; HPV vax safety)
945	RefID: 945, Pioneers in Dermatology and Venereology: An Interview with Professor Annamari Ranki<br>Ranki, A.<br>Level: 1, State: Excluded	Not about HPV vaccination/vax attitudes (eg HPV infection/serology/prevalence; cervical cancer; HPV vax safety)
946	RefID: 946, Awareness of Cancer Cervix and its prevention with HPV vaccine in internees, final MBBS students, Nursing staff and paramedical staff in a teaching institute<br>Rao, V. R., Revathi, P., Karuna, V., Jose, J.<br>Level: 1, State: Excluded	Wrong/no population (eg parents, providers, children, policy makers, does not include 18-26yrs olds; social media posts)

947	RefID: 947, Human Papillomavirus Vaccine Increases High-Risk Sexual Behaviors: A Myth or Valid Concern<br>Ratanasiripong, Nop T.<br>Level: 1, State: Excluded	Not about HPV vaccination/vax attitudes (eg HPV infection/serology/prevalence; cervical cancer; HPV vax safety)
948	RefID: 948, HPV vaccination and factors influencing vaccine uptake among people of Indian ancestry living in the United States<br>Ratnasamy, P., Chagpar, A. B.<br>Level: 1, State: Excluded	Study Design - Not qualitative (methods or analysis) OR Qualitative survey data not analyzed qualitatively (only numeric stats)
949	RefID: 949, The effect of the Affordable Care Act dependent coverAge - Young adults 18-26yrs not included /outside age range provision on HPV vaccine uptake in young adult women, National Health and Nutrition Examination Survey 2007–2016<br>Raymond, S., Li, L., Taioli, E., Nash, D., Liu, B.<br>Level: 1, State: Excluded	Not about HPV vaccination/vax attitudes (eg HPV infection/serology/prevalence; cervical cancer; HPV vax safety)
950	RefID: 950, Adolescent Perceptions of Technology-Based Sexual and Reproductive Health Services: A Systematic Review<br>Rea, S., Zynda, A., Allison, B., Tolleson-Rinehart, S.<br>Level: 1, State: Excluded	Study Design - Not qualitative (methods or analysis) OR Qualitative survey data not analyzed qualitatively (only numeric stats)
951	RefID: 951, Usability Evaluation of the Novel Smartphone Application, HPV Vaccine: Same Way, Same Day, Among Pediatric Residents<br>Real, F. J., Rosen, B. L., Bishop, J. M., McDonald, S., DeBlasio, D., Kreps, G. L., Klein, M., Kahn, J. A.<br>Level: 1, State: Excluded	Study Design - Not qualitative (methods or analysis) OR Qualitative survey data not analyzed qualitatively (only numeric stats)
952	RefID: 952, Age - Young adults 18-26yrs not included /outside age range-specific outcomes from the first round of HPV screening in unvaccinated women: Observational study from the English cervical screening pilot<br>Rebolj, M., Mathews, C. S., Pesola, F., Cuschieri, K., Denton, K., Kitchener, H.<br>Level: 1, State: Excluded	Wrong publication type (eg reviews, opinions, conference proceedings)
954	RefID: 954, Test performance and acceptability of self- versus provider-collected swabs for high-risk HPV DNA testing in female-to-male trans masculine patients<br>Reisner, S. L., Deutsch, M. B., Peitzmeier, S. M., White Hughto, J. M., Cavanaugh, T. P., Pardee, D. J., McLean, S. A., Panther, L. A., Gelman, M., Mimiaga, M. J., et al.<br>Level: 1, State: Excluded	Wrong/no population (eg parents, providers, children, policy makers, does not include 18-26yrs olds; social media posts)
956	RefID: 956, Human papillomavirus knowledge and vaccine acceptability among a national sample of heterosexual men<br>Reiter, P. L., Brewer, N. T., Smith, J. S.<br>Level: 1, State: Excluded	Not about HPV vaccination/vax attitudes (eg HPV infection/serology/prevalence; cervical cancer; HPV vax safety)
957	RefID: 957, Effects of a web-based HPV vaccination intervention on cognitive outcomes among young gay, bisexual, and other men who have sex with men<br>Reiter, P. L., Gower, A. L., Kiss, D. E., Shoben, A. B., Katz, M. L., Bauermeister, J. A., Paskett, E. D., McRee, A. L.<br>Level: 1, State: Excluded	Not about HPV vaccination/vax attitudes (eg HPV infection/serology/prevalence; cervical cancer; HPV vax safety)

959	RefID: 959, A qualitative study of HPV vaccine acceptability among health workers, teachers, parents, female pupils, and religious leaders in northwest Tanzania<br>Remes, P., Selestine, V., Changalucha, J., Ross, D. A., Wight, D., de Sanjosé, S., Kapiga, S., Hayes, R. J., Watson-Jones, D.<br>Level: 2, State: Excluded	Study Design - Not qualitative (methods or analysis) OR Qualitative survey data not analyzed qualitatively (only numeric stats)
960	RefID: 960, The epidemiology of sexually transmitted co-infections in HIV-positive and HIV-negative African-Caribbean women in Toronto<br>Remis, R. S., Liu, J., Loutfy, M., Tharao, W., Rebbapragada, A., Perusini, S. J., Chieza, L., Saunders, M., Green-Walker, L., Kaul, R.<br>Level: 1, State: Excluded	Study Design - Not qualitative (methods or analysis) OR Qualitative survey data not analyzed qualitatively (only numeric stats)
961	RefID: 961, Factors associated with HPV vaccine refusal among young adult women after ten years of vaccine implementation<br>Restivo, V., Costantino, C., Fazio, T. F., Casuccio, N., D'Angelo, C., Vitale, F., Casuccio, A.<br>Level: 1, State: Excluded	Wrong/no population (eg parents, providers, children, policy makers, does not include 18-26yrs olds; social media posts)
962	RefID: 962, Healthcare resource utilization and costs in 23-25-year-old women with human papillomavirus (HPV) associated anogenital diseases in Germany - a retrospective analysis of statutory health insurance claims data<br>Reuschenbach, M., Mihm, S., Wölle, R., Schneider, K. M., Jacob, C., Greiner, W., Hampl, M., Goodman, E.<br>Level: 1, State: Excluded	Not about HPV vaccination/vax attitudes (eg HPV infection/serology/prevalence; cervical cancer; HPV vax safety)
964	RefID: 964, Prevalence of human papilloma virus (HPV) genotypes between outpatients males and females referred to seven laboratories in Tehran, Iran<br>Rezaee Azhar, I., Yaghoobi, M., Mossalaeie, M. M., Kollaee Darabi, A., Nejadeh, A. H., Jamshidi, M., Ahani, A., Karkhane Mahmoodi, M., Ghalichi, L., Shabanzadeh, A., Ataei-Pirkooh, A., Marjani, A., Khamseh, A., Shafiei, M., Hosseini, P., Soltani, S., Zandi, M., Ghafari, P., Aboofazeli, A., Ghaziasadi, A., Jazayeri, S. M.<br>Level: 1, State: Excluded	Study Design - Not qualitative (methods or analysis) OR Qualitative survey data not analyzed qualitatively (only numeric stats)
965	RefID: 965, Understanding views against the HPV vaccine school-entry requirement in Puerto Rico<br>Rivas, G., Soto-Abreu, R., Arroyo-Morales, G. O., Medina-Laabes, D. T., Diaz-Miranda, O. L., Vazquez-Otero, C., Colon-Lopez, V.<br>Level: 1, State: Excluded	Not about HPV vaccination/vax attitudes (eg HPV infection/serology/prevalence; cervical cancer; HPV vax safety)
966	RefID: 966, Content analysis of online media coverAge - Young adults 18-26yrs not included /outside age range of the human papillomavirus vaccine as a school-entry policy in Puerto Rico<br>Rivera-Figueroa, V., Arroyo-Morales, G. O., Soto-Abreu, R., Rivera-Encarnacion, M. E., Diaz-Miranda, O. L., Medina-Laabes, D. T., Ortiz-Martinez, A. P., Suarez-Perez, E. L., Fernandez, M. E., Hull, P. C., Colon-Lopez, V.<br>Level: 1, State: Excluded	Not about HPV vaccination/vax attitudes (eg HPV infection/serology/prevalence; cervical cancer; HPV vax safety)
967	RefID: 967, Knowledge, Attitudes and Perceptions about Cervical Cancer Risk, Prevention and Human Papilloma Virus (HPV) in Vulnerable Women in Greece<br>Riza, E., Karakosta, A., Tsiampalis, T., Lazarou, D., Karachaliou, A., Ntelis, S., KarAge - Young adults 18-26yrs not included /outside age	Wrong/no population (eg parents, providers, children, policy makers, does not include 18-26yrs olds; social media posts)

	rangeorgiou, V., Psaltopoulou, T.<br>Level: 1, State: Excluded	
968	RefID: 968, Cervical cancer prevention: Asian-American women's knowledge and participation in screening practices<br>Robison, K., Clark, L., Eng, W., Wu, L., Raker, C., Clark, M., Tejada-Berges, T., Dizon, D. S.<br>Level: 1, State: Excluded	Wrong publication type (eg reviews, opinions, conference proceedings)
969	RefID: 969, The influence of deductible health plans on receipt of the human papillomavirus vaccine series<br>Roblin, D. W., Ritzwoller, D. P., Rees, D. I., Carroll, N. M., Chang, A., Daley, M. F.<br>Level: 1, State: Excluded	Age - Young adults 18-26yrs not included /outside age range
970	RefID: 970, Factors Affecting Delivery of the HPV Vaccination: A Focus Group Study With NHS School-Age - Young adults 18-26yrs not included /outside age ranged Vaccination Teams in London<br>Rockliffe, Lauren, McBride, Emily, Heffernan, Catherine, Forster, Alice S.<br>Level: 1, State: Excluded	Study Design - Not qualitative (methods or analysis) OR Qualitative survey data not analyzed qualitatively (only numeric stats)
971	RefID: 971, A qualitative exploration of using financial incentives to improve vaccination uptake via consent form return in female adolescents in London<br>Rockliffe, L., Stearns, S., Forster, A. S.<br>Level: 1, State: Excluded	Not about HPV vaccination/vax attitudes (eg HPV infection/serology/prevalence; cervical cancer; HPV vax safety)
972	RefID: 972, [Prevalence of cytological atypia and high-risk human papillomavirus infection in Panará indigenous women in Central Brazil]<br>Rodrigues, D. A., Pereira É, R., Oliveira, L. S., Speck, N. M., Gimeno, S. G.<br>Level: 1, State: Excluded	Wrong/no population (eg parents, providers, children, policy makers, does not include 18-26yrs olds; social media posts)
974	RefID: 974, Barriers to HPV vaccine access<br>Rogg, K., Goodman, E., Grimes, C., Naresh, A., Robinson, W.<br>Level: 2, State: Excluded	Not about HPV vaccination/vax attitudes (eg HPV infection/serology/prevalence; cervical cancer; HPV vax safety)
975	RefID: 975, Tactics of strengthening for the prevention of uterine cervical cancer through the vaccination against the virus of the human papiloma, august 2017 to march 2018<br>Rojas, E. A., Rojas, W. A. A., Flores, R. S., Flores, O. S.<br>Level: 2, State: Excluded	Not about HPV vaccination/vax attitudes (eg HPV infection/serology/prevalence; cervical cancer; HPV vax safety)
976	RefID: 976, Cervical cancer screening among young adult women in the United States<br>Roland, K. B., Benard, V. B., Soman, A., Breen, N., Kepka, D., Saraiya, M.<br>Level: 1, State: Excluded	Study Design - Not qualitative (methods or analysis) OR Qualitative survey data not analyzed qualitatively (only numeric stats)
977	RefID: 977, Immune response to the HPV-16/18 AS04-adjuvanted vaccine administered as a 2-dose or 3-dose schedule up to 4 years after vaccination: results from a randomized study<br>Romanowski, B., Schwarz, T. F., Ferguson, L. M., Ferguson, M., Peters, K., Dionne, M., Schulze, K., Ramjattan, B., Hillemanns, P., Behre, U., et al.<br>Level: 1, State: Excluded	Study Design - Not qualitative (methods or analysis) OR Qualitative survey data not analyzed qualitatively (only numeric stats)
978	RefID: 978, A content analysis of HPV vaccine online continuing medical education purpose statements and learning objectives	Not about HPV vaccination/vax attitudes (eg HPV

	Rosen, B. L., Bishop, J. M., Anderson, R., Real, F. J., Klein, M. D., Kreps, G. L.<br>Level: 1, State: Excluded	infection/serology/prevalence; cervical cancer; HPV vax safety)
979	RefID: 979, Feasibility and sustainability of a school-based platform for integrated delivery of HPV vaccination with adolescent health services in Tanzania: qualitative insights from stakeholders<br>Rosen, J. G., Guillaume, D., Mlunde, L. B., Njiro, B. J., Munishi, C., Mlay, D., Gerste, A., Holroyd, T. A., Giattas, M. R., Morgan, C., Kyesi, F., Tinuga, F., Ishengoma, J., Sunguya, B. F., Limaye, R. J.<br>Level: 1, State: Excluded	Not about HPV vaccination/vax attitudes (eg HPV infection/serology/prevalence; cervical cancer; HPV vax safety)
980	RefID: 980, What Are They Thinking? Findings for Educators and Practitioners on Youths' Experience and Knowledge of the HPV Vaccine<br>Rosenbloom, S. R., Killian, C.<br>Level: 2, State: Excluded	Not about HPV vaccination/vax attitudes (eg HPV infection/serology/prevalence; cervical cancer; HPV vax safety)
981	RefID: 981, Design and feasibility of a study using the clinical practice research datalink general practice online database (CPRD gold) to assess the risk of new onset of auto-immune diseases (NOAD) following administration of the human papillomavirus (HPV)-16/18 AS04-adjuvanted vaccine<br>Rosillon, D., Willame, C., Pladevall, M., Zima, J., Van Den Bosch, J. H., Bunge, E., Van Staa, T., Boggon, R., Baril, L.<br>Level: 1, State: Excluded	Wrong/no population (eg parents, providers, children, policy makers, does not include 18-26yrs olds; social media posts)
983	RefID: 983, Communication method preference for patient reminders<br>Roston, A., Stern, L., Debevec, E., Davis, M., Todd, G., Morfesis, J., Patel, A.<br>Level: 1, State: Excluded	Multiple Wrong/no population (eg parents, providers, children, policy makers, does not include 18-26yrs olds; social media posts)s - unable to extract data specific to young adults (18-26 yrs)
984	RefID: 984, Safety and immunogenicity of the quadrivalent human papillomavirus vaccine in patients with childhood systemic lupus erythematosus: a real-world interventional multi-centre study<br>Rotstein Grein, I. H., Pinto, N. F., Lobo, A., Groot, N., Sztajnbok, F., da Silva, C. A. A., Paim Marques, L. B., Appenzeller, S., Islabão, A. G., Magalhães, C. S., de Almeida, R. G., Bica, B., Fraga, M., da Fraga, A. C. M., dos Santos, M. C., Robazzi, T., Terreri, M. T. R. A., Bandeira, M., Pasmans, H., Schepp, R., van der Klis, F., de Roock, S., Wulffraat, N., Pileggi, G.<br>Level: 1, State: Excluded	Not about HPV vaccination/vax attitudes (eg HPV infection/serology/prevalence; cervical cancer; HPV vax safety)
985	RefID: 985, Cytological Anal Squamous Intraepithelial Lesions Associated with Anal High-Risk Human Papillomavirus Infections among Men Who Have Sex with Men in Northern Thailand<br>Ruanpeng, D., Chariyalertsak, S., Kaewpoowat, Q., Supindham, T., Settakorn, J., Sukpan, K., Utaipat, U., Miura, T., Kosashunhanan, N., Saokhieo, P., Songsupa, R., Wongthanee, A.<br>Level: 1, State: Excluded	Study Design - Not qualitative (methods or analysis) OR Qualitative survey data not analyzed qualitatively (only numeric stats)
986	RefID: 986, Healthcare provider perspectives on the uptake of the human papillomavirus vaccine among newcomers to Canada: a qualitative study<br>Rubens-Augustson, T., Wilson, L. A., Murphy, M. S., Jardine, C., Pottie,	Not about HPV vaccination/vax attitudes (eg HPV infection/serology/prevalence; cervical cancer; HPV vax safety)

	K., Hui, C., Stafström, M., Wilson, K.<br>Level: 1, State: Excluded	
990	RefID: 990, Human papillomavirus vaccine knowledge and hypothetical acceptance among women in Appalachia Ohio<br>Ruffin, M. T. th, Hade, E. M., Gorsline, M. R., DeGraffinreid, C. R., Katz, M. L., Kobrin, S. C., Paskett, E. D.<br>Level: 1, State: Excluded	Not about HPV vaccination/vax attitudes (eg HPV infection/serology/prevalence; cervical cancer; HPV vax safety)
991	RefID: 991, Impact of an Electronic Health Record (EHR) Reminder on Human Papillomavirus (HPV) Vaccine Initiation and Timely Completion<br>Ruffin, M. T. th, Plegue, M. A., Rockwell, P. G., Young, A. P., Patel, D. A., Yeazel, M. W.<br>Level: 1, State: Excluded	Wrong/no population (eg parents, providers, children, policy makers, does not include 18-26yrs olds; social media posts)
992	RefID: 992, Immunizing against vaccine hesitancy: An assessment of online communication and social network factors impacting vaccine adoption<br>Ruiz, Jeanette B.<br>Level: 1, State: Excluded	Study Design - Not qualitative (methods or analysis) OR Qualitative survey data not analyzed qualitatively (only numeric stats)
993	RefID: 993, Why don't adolescent girls in a rural Uganda district initiate or complete routine 2-dose HPV vaccine series: Perspectives of adolescent girls, their caregivers, healthcare workers, community health workers and teachers<br>Rujumba, J., Akugizibwe, M., Basta, N. E., Banura, C.<br>Level: 2, State: Excluded	Study Design - Not qualitative (methods or analysis) OR Qualitative survey data not analyzed qualitatively (only numeric stats)
994	RefID: 994, Evaluation of the knowledge of students concerning sexually transmitted infections in Bavaria/Germany (a cross-sectional study)<br>Rummel, M., Clanner-Engelshofen, B. M., Nellessen, T., Zippel, S., Schuster, B., French, L. E., Reinholz, M.<br>Level: 1, State: Excluded	Study Design - Not qualitative (methods or analysis) OR Qualitative survey data not analyzed qualitatively (only numeric stats)
995	RefID: 995, Missed Opportunities for HPV Vaccination Among Vaccine-Eligible Women with High Grade Cervical Lesions<br>Russ, S. M., Brackney, M., Meek, J., Niccolai, L. M.<br>Level: 1, State: Excluded	Multiple Wrong/no population (eg parents, providers, children, policy makers, does not include 18-26yrs olds; social media posts)s - unable to extract data specific to young adults (18-26 yrs)
996	RefID: 996, Disparity in HPV vaccine use 2009-2015 among young adults in the US interview survey<br>Ruyi, T.<br>Level: 1, State: Excluded	Age - Young adults 18-26yrs not included /outside age range
997	RefID: 997, Knowledge, Attitude and Practice Concerning Human Papilloma Virus Infection and its Health Effects among Rural Women, Karnataka, South India<br>Sabeena, S., Bhat, P. V., Kamath, V., Aswathyraj, S., Arunkumar, G.<br>Level: 1, State: Excluded	Study Design - Not qualitative (methods or analysis) OR Qualitative survey data not analyzed qualitatively (only numeric stats)
998	RefID: 998, The comparison of human papillomavirus knowledge by general characteristics in vaccinated Thai women<br>Saeloo, S., Taepisitpong, C., Kantathavorn, N., Rawangban, S., Kwangkaew, S., Krongthong, W.<br>Level: 1, State: Excluded	Study Design - Not qualitative (methods or analysis) OR Qualitative survey data not analyzed qualitatively (only numeric stats)

999	RefID: 999, Epidemiological study of anti-HPV16/18 seropositivity and subsequent risk of HPV16 and -18 infections<br>Safaeian, M., Porras, C., Schiffman, M., Rodriguez, A. C., Wacholder, S., Gonzalez, P., Quint, W., van Doorn, L. J., Sherman, M. E., Xhenseval, V., Herrero, R., Hildesheim, A.<br>Level: 1, State: Excluded	Study Design - Not qualitative (methods or analysis) OR Qualitative survey data not analyzed qualitatively (only numeric stats)
1002	RefID: 1002, Recommendations for Structure and Content for a School-Based Adolescent Immunization Curriculum<br>Salazar, K. R., Seib, K. G., Underwood, N. L., Gargano, L. M., Sales, J. M., Morfaw, C., Murray, D., Diclemente, R. J., Hughes, J. M.<br>Level: 1, State: Excluded	Age - Young adults 18-26yrs not included /outside age range
1003	RefID: 1003, Predictor factors for conservative manAge - Young adults 18-26yrs not included /outside age rangement of cervical intraepithelial neoplasia grade 2: Cytology and HPV genotyping<br>Salvadó, A., Miralpeix, E., Solé-Sedeno, J. M., Kanjou, N., Lloveras, B., Duran, X., Mancebo, G.<br>Level: 1, State: Excluded	Study Design - Not qualitative (methods or analysis) OR Qualitative survey data not analyzed qualitatively (only numeric stats)
1004	RefID: 1004, Ethical issues related to human papillomavirus vaccination programs: an example from Bangladesh<br>Salwa, M., Abdullah Al-Munim, T.<br>Level: 1, State: Excluded	Age - Young adults 18-26yrs not included /outside age range
1005	RefID: 1005, Sexual and reproductive health care: adolescent and adult men's willingness to talk and preferred approach<br>Same, R. V., Bell, D. L., Rosenthal, S. L., Marcell, A. V.<br>Level: 1, State: Excluded	Not about HPV vaccination/vax attitudes (eg HPV infection/serology/prevalence; cervical cancer; HPV vax safety)
1006	RefID: 1006, Design and statistical considerations for studies evaluating the efficacy of a single dose of the human papillomavirus (HPV) vaccine<br>Sampson, J. N., Hildesheim, A., Herrero, R., Gonzalez, P., Kreimer, A. R., Gail, M. H.<br>Level: 1, State: Excluded	Age - Young adults 18-26yrs not included /outside age range
1007	RefID: 1007, [Knowledge and acceptance of vaccine against human papillomavirus among mothers of students from Durango city, Mexico]<br>Sánchez Anguiano, L. F., Lechuga Quiñones, A. M., Milla Villeda, R. H., Lares Bayona, E. F.<br>Level: 1, State: Excluded	Study Design - Not qualitative (methods or analysis) OR Qualitative survey data not analyzed qualitatively (only numeric stats)
1009	RefID: 1009, HPV vaccine acceptance among Latina mothers by HPV status<br>Sanderson, M., Coker, A. L., Eggleston, K. S., Fernandez, M. E., Arrastia, C. D., Fadden, M. K.<br>Level: 1, State: Excluded	Not about HPV vaccination/vax attitudes (eg HPV infection/serology/prevalence; cervical cancer; HPV vax safety)
1010	RefID: 1010, Medical students' knowledge and attitudes regarding vaccination against measles, influenza and HPV. An international multicenter study<br>Sanftenberg, L., Roggendorf, H., Babucke, M., Breckwoldt, J., Gaertner, B., Hetzer, B., Lendeckel, A., Riemenschneider, H., Voigt, K., Keplinger, A., Wiedermann, U., Berberat, P. O., Schelling, J.<br>Level: 1, State: Excluded	Wrong/no population (eg parents, providers, children, policy makers, does not include 18-26yrs olds; social media posts)
1011	RefID: 1011, Development of Human Papillomavirus (HPV) Vaccines: A Review of Literature and Clinical Update	Wrong/no population (eg parents, providers, children, policy makers,

	Sangar, V. C., Ghongane, B., Mathur, G.<br>Level: 1, State: Excluded	does not include 18-26yrs olds; social media posts)
1012	RefID: 1012, Human papillomavirus infection and cervical cancer prevention in India, Bangladesh, Sri Lanka and Nepal<br>Sankaranarayanan, R., Bhatla, N., Gravitt, P. E., Basu, P., Esmy, P. O., Ashrafunnessa, K. S., Ariyaratne, Y., Shah, A., Nene, B. M.<br>Level: 1, State: Excluded	Study Design - Not qualitative (methods or analysis) OR Qualitative survey data not analyzed qualitatively (only numeric stats)
1013	RefID: 1013, Knowledge, Attitude, and Practice Towards Cervical Cancer Among Primary School Female Teachers in Phnom Penh<br>Sann, C., Koum, K., Kruy, L., Kim, L., Chhit, M., Uy, K., Krouch, R., Korn, A., Shikino, K., Tomoko, K., Nozomu, Y., Aiko, O.<br>Level: 1, State: Excluded	Wrong publication type (eg reviews, opinions, conference proceedings)
1014	RefID: 1014, Contraceptive methods and knowledge of sexually transmitted diseases in nursing students. Results from a survey conducted at the University of Palermo<br>Santangelo, O. E., Provenzano, S., Alagna, E., Terranova, A., D'Anna, G., Grigis, D., Cedrone, F., Firenze, A.<br>Level: 1, State: Excluded	Not about HPV vaccination/vax attitudes (eg HPV infection/serology/prevalence; cervical cancer; HPV vax safety)
1015	RefID: 1015, Factors involved in human papillomavirus (HPV) vaccine hesitancy among women in the South-East Asian Region (SEAR) and Western Pacific Region (WPR): A scoping review<br>Santhanes, D., Wong, C. P., Yap, Y. Y., San, S. P., Chaiyakunapruk, N., Khan, T. M.<br>Level: 1, State: Excluded	Not about HPV vaccination/vax attitudes (eg HPV infection/serology/prevalence; cervical cancer; HPV vax safety)
1016	RefID: 1016, Oncogenic human papilloma virus and cervical pre-cancerous lesions in brothel-based sex workers in India<br>Sarkar, K., Bhattacharya, S., Bhattacharyya, S., Chatterjee, S., Mallick, A. H., Chakraborti, S., Chatterjee, D., Bal, B.<br>Level: 1, State: Excluded	Study Design - Not qualitative (methods or analysis) OR Qualitative survey data not analyzed qualitatively (only numeric stats)
1017	RefID: 1017, Differential uptake of recent Papanicolaou testing by HPV vaccination status among young women in the United States, 2008-2013<br>Sauer, A. G., Jemal, A., Simard, E. P., Fedewa, S. A.<br>Level: 1, State: Excluded	Wrong publication type (eg reviews, opinions, conference proceedings)
1018	RefID: 1018, Reducing Cancer and Cancer Disparities: Lessons From a Youth-Generated Diabetes Prevention Campaign<br>Schillinger, D., Ling, P. M., Fine, S., Boyer, C. B., Rogers, E., Vargas, R. A., Bibbins-Domingo, K., Chou, W. Y. S.<br>Level: 1, State: Excluded	Not about HPV vaccination/vax attitudes (eg HPV infection/serology/prevalence; cervical cancer; HPV vax safety)
1019	RefID: 1019, Incidence and Types of Human Papillomavirus Infections in Adolescent Girls and Young Women Immunized With the Human Papillomavirus Vaccine<br>Schlecht, Nicolas F., Diaz, Angela, Nucci-Sack, Anne, Shyhalla, Kathleen, Shankar, Viswanathan, Guillot, Mary, Hollman, Dominic, Strickler, Howard D., Burk, Robert D.<br>Level: 1, State: Excluded	Study Design - Not qualitative (methods or analysis) OR Qualitative survey data not analyzed qualitatively (only numeric stats)
1020	RefID: 1020, Differences in patterns of high-risk human papillomavirus infection between urban and rural low-resource settings: cross-sectional findings from Mali	Age - Young adults 18-26yrs not included /outside age range

	Schluterman, N. H., Sow, S. O., Traore, C. B., Bakarou, K., Dembelé, R., Sacko, F., Gravitt, P. E., Tracy, J. K.<br>Level: 1, State: Excluded	
1021	RefID: 1021, Young adults awareness of HPV and vaccine acceptance after introduction of the HPV vaccine in the Dutch national vaccination program<br>Schmeink, C. E., Gosens, K. C., Melchers, W. J., Massuger, L. F., Bekkers, R. L.<br>Level: 2, State: Excluded	Not about HPV vaccination/vax attitudes (eg HPV infection/serology/prevalence; cervical cancer; HPV vax safety)
1023	RefID: 1023, Vaccination interest and trends in human papillomavirus vaccine uptake in young adult women Age - Young adults 18-26yrs not included /outside age ranged 18 to 26 years in the United States: an analysis using the 2008-2012 National Health Interview Survey<br>Schmidt, S., Parsons, H. M.<br>Level: 1, State: Excluded	Not about HPV vaccination/vax attitudes (eg HPV infection/serology/prevalence; cervical cancer; HPV vax safety)
1028	RefID: 1028, Using an Implementation Research Framework to Identify Potential Facilitators and Barriers of an Intervention to Increase HPV Vaccine Uptake<br>Selove, R., Foster, M., Mack, R., Sanderson, M., Hull, P. C.<br>Level: 1, State: Excluded	Study Design - Not qualitative (methods or analysis) OR Qualitative survey data not analyzed qualitatively (only numeric stats)
1029	RefID: 1029, Potential process improvements to increase coverAge - Young adults 18-26yrs not included /outside age range of human papillomavirus vaccine in schools - A focus on schools with low vaccine uptake<br>Selvey, L. A., Roux, F., Burns, S.<br>Level: 2, State: Excluded	Study Design - Not qualitative (methods or analysis) OR Qualitative survey data not analyzed qualitatively (only numeric stats)
1031	RefID: 1031, Differences between African-American adolescent females with and without human papillomavirus infection<br>Seth, P., Wingood, G. M., Diclemente, R. J., Crosby, R. A., Salazar, L. F., Rose, E. S., Sales, J. M.<br>Level: 1, State: Excluded	Wrong/no population (eg parents, providers, children, policy makers, does not include 18-26yrs olds; social media posts)
1032	RefID: 1032, Exposure to high-risk genital human papillomavirus and its association with risky sexual practices and laboratory-confirmed chlamydia among African-American women<br>Seth, P., Wingood, G. M., Robinson, L. S., Diclemente, R. J.<br>Level: 1, State: Excluded	Wrong/no population (eg parents, providers, children, policy makers, does not include 18-26yrs olds; social media posts)
1033	RefID: 1033, Challenges to Human Papillomavirus Vaccine Acceptability among Women in South India: An Exploratory Study<br>Shah, P., Shetty, V., Ganesh, M., Shetty, A. K.<br>Level: 2, State: Excluded	Study Design - Not qualitative (methods or analysis) OR Qualitative survey data not analyzed qualitatively (only numeric stats)
1034	RefID: 1034, Digital public health surveillance: a systematic scoping review<br>Shakeri Hossein Abad, Z., Kline, A., Sultana, M., Noaeen, M., Nurmambetova, E., Lucini, F., Al-Jefri, M., Lee, J.<br>Level: 1, State: Excluded	Not about HPV vaccination/vax attitudes (eg HPV infection/serology/prevalence; cervical cancer; HPV vax safety)
1035	RefID: 1035, Prevalence of human papillomavirus infection among women in rural Nepal<br>Shakya, S., Syversen, U., Åsvold, B. O., Bofin, A. M., Aune, G., Nordbø, S.	Multiple Wrong/no population (eg parents, providers, children, policy makers, does not include

	A., Vaidya, K. M., Karmacharya, B. M., Afset, J. E., Tingulstad, S.<br>Level: 1, State: Excluded	18-26yrs olds; social media posts)s - unable to extract data specific to young adults (18-26 yrs)
1036	RefID: 1036, Prevalence and type distribution of human papillomavirus among women older than 18 years in Egypt: a multicenter, observational study<br>Shaltout, M. F., Sallam, H. N., AbouSeeda, M., Moiety, F., Hemeda, H., Ibrahim, A., Sherbini, M. E., Rady, H., Gopala, K., DeAntonio, R.<br>Level: 1, State: Excluded	Wrong publication type (eg reviews, opinions, conference proceedings)
1037	RefID: 1037, Objective quantification of spontaneous retinal venous pulsations using a novel tablet-based ophthalmoscope<br>Shariflou, S., Agar, A., Rose, K., Bowd, C., Golzan, S. M.<br>Level: 1, State: Excluded	Age - Young adults 18-26yrs not included /outside age range
1038	RefID: 1038, Development of culturally tailored educational brochures on HPV and pap tests for American Indian women<br>Sharpe, P. A., Brandt, H. M., McCree, D. H., Owl-Myers, E., Taylor, B., Mullins, G.<br>Level: 1, State: Excluded	Wrong/no population (eg parents, providers, children, policy makers, does not include 18-26yrs olds; social media posts)
1039	RefID: 1039, AVPCancerFree: impact of a digital behavior change intervention on parental HPV vaccine –related perceptions and behaviors<br>Shegog, R., Savas, L. S., Healy, C. M., Frost, E. L., Coan, S. P., Gabay, E. K., Preston, S. M., Spinner, S. W., Wilbur, M., Becker, E., et al.<br>Level: 1, State: Excluded	Not about HPV vaccination/vax attitudes (eg HPV infection/serology/prevalence; cervical cancer; HPV vax safety)
1042	RefID: 1042, The factors associated with maternal consent to human papillomavirus vaccination among adolescents in Israel<br>Shibli, R., Rishpon, S.<br>Level: 1, State: Excluded	Wrong/no population (eg parents, providers, children, policy makers, does not include 18-26yrs olds; social media posts)
1043	RefID: 1043, An Internet-Based Education Program for Human Papillomavirus Vaccination Among Female College Students in Mainland China: application of the Information-Motivation-Behavioral Skills Model in a Cluster Randomized Trial<br>Si, M., Su, X., Jiang, Y., Wang, W., Zhang, X., Gu, X., Ma, L., Li, J., Zhang, S., Ren, Z., et al.<br>Level: 1, State: Excluded	Wrong/no population (eg parents, providers, children, policy makers, does not include 18-26yrs olds; social media posts)
1045	RefID: 1045, Distribution of human papillomavirus genotypes in invasive cervical cancer in Italy: a representative, single institution case series<br>Sideri, M., Cristoforoni, P., Casadio, C., Boveri, S., Igidbashian, S., Schmitt, M., Gheit, T., Tommasino, M.<br>Level: 1, State: Excluded	Wrong/no population (eg parents, providers, children, policy makers, does not include 18-26yrs olds; social media posts)
1046	RefID: 1046, Age - Young adults 18-26yrs not included /outside age range distribution of HPV genotypes in cervical intraepithelial neoplasia<br>Sideri, M., Igidbashian, S., Boveri, S., Radice, D., Casadio, C., Spolti, N., Sandri, M. T.<br>Level: 1, State: Excluded	Study Design - Not qualitative (methods or analysis) OR Qualitative survey data not analyzed qualitatively (only numeric stats)
1047	RefID: 1047, Perceptions, Knowledge and Attitudes among Young Adults about Prevention of HPV Infection and Immunization<br>Sidiropoulou, Maria, Gerogianni, Georgia, Kourti, Freideriki Eleni, Pappa, Despoina, Zartaloudi, Afroditi, Koutelekos, Ioannis, Dousis,	Not about HPV vaccination/vax attitudes (eg HPV infection/serology/prevalence; cervical cancer; HPV vax safety)

	Evangelos, Margari, Nikoletta, Mangoulia, Polyxeni, Ferentinou, Eftychia, Giga, Anna, Zografakis-Sfakianakis, Michail, Dafogianni, Chrysoula<br>Level: 1, State: Excluded	
1048	RefID: 1048, Risk Factors for Non-Human Papillomavirus (HPV) Type 16/18 Cervical Infections and Associated Lesions Among HPV DNA-Negative Women Vaccinated Against HPV-16/18 in the Costa Rica Vaccine Trial<br>Sierra, Mónica S., Tsang, Sabrina H., Hu, Shangying, Porras, Carolina, Herrero, Rolando, Kreimer, Aimée R., Schussler, John, Boland, Joseph, Wagner, Sarah, Cortes, Bernal, Rodríguez, Ana C., Quint, Wim, Doorn, Leen-Jan van, Schiffman, Mark, Sampson, Joshua N., Hildesheim, Allan, Group, Costa Rica Human Papillomavirus Vaccine Trial, van Doorn, Leen-Jan<br>Level: 1, State: Excluded	Not about HPV vaccination/vax attitudes (eg HPV infection/serology/prevalence; cervical cancer; HPV vax safety)
1051	RefID: 1051, Knowledge of Human Papillomavirus and Cervical Cancer Among Low-Income Women in New Jersey<br>Silvera, S. A. N., Kaplan, A. M., Laforet, P.<br>Level: 1, State: Excluded	Study Design - Not qualitative (methods or analysis) OR Qualitative survey data not analyzed qualitatively (only numeric stats)
1052	RefID: 1052, Effectiveness of “catch-up” HPV vaccination on incident cervical neoplasia in a U.S. healthcare setting<br>Silverberg, M., Leyden, W., Gregorich, S., Huchko, M., Kulasingam, S., Kuppermann, M., Smith-McCune, K., Sawaya, G.<br>Level: 1, State: Excluded	Study Design - Not qualitative (methods or analysis) OR Qualitative survey data not analyzed qualitatively (only numeric stats)
1053	RefID: 1053, Effectiveness of catch-up human papillomavirus vaccination on incident cervical neoplasia in a US health-care setting: a Wrong/no population (eg parents, providers, children, policy makers, does not include 18-26yrs olds; social media posts)-based case-control study<br>Silverberg, M. J., Leyden, W. A., Lam, J. O., Gregorich, S. E., Huchko, M. J., Kulasingam, S., Kuppermann, M., Smith-McCune, K. K., Sawaya, G. F.<br>Level: 1, State: Excluded	Not about HPV vaccination/vax attitudes (eg HPV infection/serology/prevalence; cervical cancer; HPV vax safety)
1054	RefID: 1054, A survey of Wrong/no population (eg parents, providers, children, policy makers, does not include 18-26yrs olds; social media posts)-based utility scores for cervical cancer prevention<br>Simonella, L., Howard, K., Canfell, K.<br>Level: 1, State: Excluded	Age - Young adults 18-26yrs not included /outside age range
1055	RefID: 1055, Disclosure of Sexual Behavior Is Significantly Associated With Receiving a Panel of Health Care Services Recommended for Men Who Have Sex With Men<br>Singh, V., Crosby, R. A., Gratzer, B., Gorbach, P. M., Markowitz, L. E., Meites, E.<br>Level: 1, State: Excluded	Study Design - Not qualitative (methods or analysis) OR Qualitative survey data not analyzed qualitatively (only numeric stats)
1057	RefID: 1057, Acceptability and response to a postal survey using self-taken samples for HPV vaccine impact monitoring<br>Sinka, K., Lacey, M., Robertson, C., Kavanagh, K., Cuschieri, K., Nicholson, D., Donaghy, M.<br>Level: 1, State: Excluded	Not about HPV vaccination/vax attitudes (eg HPV infection/serology/prevalence; cervical cancer; HPV vax safety)

1058	RefID: 1058, An Integrative Review of the Influences on Decision-Making of Young People About Human Papillomavirus Vaccine<br>Sisson, Helen, Wilkinson, Yvonne<br>Level: 1, State: Excluded	Not about HPV vaccination/vax attitudes (eg HPV infection/serology/prevalence; cervical cancer; HPV vax safety)
1060	RefID: 1060, Social and cultural construction processes involved in HPV vaccine hesitancy among Chinese women: a qualitative study<br>Siu, J. Y., Fung, T. K. F., Leung, L. H.<br>Level: 2, State: Excluded	Study Design - Not qualitative (methods or analysis) OR Qualitative survey data not analyzed qualitatively (only numeric stats)
1061	RefID: 1061, Schoolteachers' experiences of implementing school-based vaccination programs against human papillomavirus in a Chinese community: a qualitative study<br>Siu, J. Y., Lee, A., Chan, P. K. S.<br>Level: 1, State: Excluded	Wrong publication type (eg reviews, opinions, conference proceedings)
1071	RefID: 1071, Seroprevalence and genital DNA prevalence of HPV types 6, 11, 16 and 18 in a cohort of young Norwegian women: study design and cohort characteristics<br>Skjeldestad, F. E., Mehta, V., Sings, H. L., Øvreness, T., Turpin, J., Su, Ling, Boerckel, P., Roberts, C., Bryan, J., Jansen, K. U., Esser, M. T., Liaw, K. L.<br>Level: 1, State: Excluded	Multiple Wrong/no population (eg parents, providers, children, policy makers, does not include 18-26yrs olds; social media posts)s - unable to extract data specific to young adults (18-26 yrs)
1072	RefID: 1072, Risk of human papillomavirus-related cancers among kidney transplant recipients and patients receiving chronic dialysis--an observational cohort study<br>Skov Dalgaard, L., Fassel, U., Østergaard, L. J., Jespersen, B., Schmeltz Søgaard, O., Jensen-Fangel, S.<br>Level: 1, State: Excluded	Wrong/no population (eg parents, providers, children, policy makers, does not include 18-26yrs olds; social media posts)
1075	RefID: 1075, The Male Voice: A Qualitative Assessment of Young Men's Communication Preferences About HPV and 9vHPV<br>Sledge, J. A., Jensen, C. E., Cibulka, N. J., Hoffman, M.<br>Level: 2, State: Excluded	Not about HPV vaccination/vax attitudes (eg HPV infection/serology/prevalence; cervical cancer; HPV vax safety)
1076	RefID: 1076, Community-Based Assessment to Inform a Chlamydia Screening Program for Women in a Rural American Indian Community<br>Smartlowit-Briggs, L., Pearson, C., Whitefoot, P., Altamirano, B. N., Womack, M., Bastin, M., Dombrowski, J. C.<br>Level: 1, State: Excluded	Not about HPV vaccination/vax attitudes (eg HPV infection/serology/prevalence; cervical cancer; HPV vax safety)
1077	RefID: 1077, Assessing knowledge of human papillomavirus and collecting data on sexual behavior: computer assisted telephone versus face to face interviews<br>Smith, A., Lyons, A., Pitts, M., Croy, S., Ryall, R., Garland, S., Wong, M. L., Tay, E. H.<br>Level: 1, State: Excluded	Not about HPV vaccination/vax attitudes (eg HPV infection/serology/prevalence; cervical cancer; HPV vax safety)
1079	RefID: 1079, The impact of publicly funded human papillomavirus (HPV) vaccination on cervical dysplasia: The ontario grade 8 HPV vaccine cohort study<br>Smith, L. M., Kaufman, J. S., Strumpf, E. C., Lévesque, L. E.<br>Level: 1, State: Excluded	Not about HPV vaccination/vax attitudes (eg HPV infection/serology/prevalence; cervical cancer; HPV vax safety)
1080	RefID: 1080, Cancer screening in the United States, 2015: A review of current American Cancer Society guidelines and current issues in cancer	Study Design - Not qualitative (methods or analysis) OR

	screening<br>Smith, R. A., Manassaram-Baptiste, D., Brooks, D., Doroshenk, M., Fedewa, S., Saslow, D., Brawley, O. W., Wender, R.<br>Level: 1, State: Excluded	Qualitative survey data not analyzed qualitatively (only numeric stats)
1081	RefID: 1081, Mental representations of HPV in Appalachia: Gender, semantic network analysis, and knowledge gaps<br>Smith, R. A., Parrott, R. L.<br>Level: 1, State: Excluded	Not about HPV vaccination/vax attitudes (eg HPV infection/serology/prevalence; cervical cancer; HPV vax safety)
1082	RefID: 1082, Parents' Knowledge and Attitude towards HPV and HPV Vaccination in Poland<br>Smolarczyk, K., Duszewska, A., Drozd, S., Majewski, S.<br>Level: 1, State: Excluded	Wrong publication type (eg reviews, opinions, conference proceedings)
1083	RefID: 1083, Adolescents' knowledge of HPV and sexually transmitted infections at public high schools in São Paulo: A cross-sectional study<br>Soares Junior, J. M., de Oliveira, H. M. C., Luquetti, C. M., Zuchelo, L. T. S., de Arruda Veiga, E. C., Raimundo, J. Z., Dos Santos Figueiredo, F. W., Alves, M. S., Sorpreso, I. C. E., Baracat, E. C.<br>Level: 1, State: Excluded	Study Design - Not qualitative (methods or analysis) OR Qualitative survey data not analyzed qualitatively (only numeric stats)
1084	RefID: 1084, Risk factors for human papillomavirus infection and abnormal cervical cytology among perinatally human immunodeficiency virus-infected and uninfected asian youth<br>Sohn, A. H., Kerr, S. J., Hansudewechakul, R., Gatechompol, S., Chokephaibulkit, K., Dang, H. L. D., Tran, D. N. H., Achalapong, J., Teeratakulpisarn, N., Chalermchockcharoenkit, A., Thamkhantho, M., Pankam, T., Singtoroj, T., Termrungruanglert, W., Chaithongwongwatthana, S., Phanuphak, N.<br>Level: 1, State: Excluded	Wrong/no population (eg parents, providers, children, policy makers, does not include 18-26yrs olds; social media posts)
1085	RefID: 1085, Decade of research into the acceptability of interventions aimed at improving adolescent and youth health and social outcomes in Africa: a systematic review and evidence map<br>Somefun, O. D., Casale, M., Haupt Ronnie, G., Desmond, C., Cluver, L., Sherr, L.<br>Level: 1, State: Excluded	Study Design - Not qualitative (methods or analysis) OR Qualitative survey data not analyzed qualitatively (only numeric stats)
1086	RefID: 1086, Epidemiology of genital warts in the British Wrong/no population (eg parents, providers, children, policy makers, does not include 18-26yrs olds; social media posts): implications for HPV vaccination programmes<br>Sonnenberg, P., Tanton, C., Mesher, D., King, E., Beddows, S., Field, N., Mercer, C. H., Soldan, K., Johnson, A. M.<br>Level: 1, State: Excluded	Not about HPV vaccination/vax attitudes (eg HPV infection/serology/prevalence; cervical cancer; HPV vax safety)
1087	RefID: 1087, Human papillomavirus school-entry vaccination mandate in Puerto Rico: Barriers and facilitators from the perspective of key informants<br>Soto-Abreu, R., Rivera-Encarnacion, M. E., Rivera-Figueroa, V., Arroyo-Morales, G. O., Medina-Laabes, D. T., Diaz-Miranda, O. L., Hull, P. C., Ortiz-Martinez, A. P., Suarez-Perez, E. L., Fernandez, M. E., Colon-Lopez, V.<br>Level: 1, State: Excluded	Wrong publication type (eg reviews, opinions, conference proceedings)
1088	RefID: 1088, A Qualitative Study of Parental Knowledge and Perceptions of Human Papillomavirus and Cervical Cancer Prevention in Rural	Not about HPV vaccination/vax attitudes (eg HPV

	Central Java, Indonesia: Understanding Community Readiness for Prevention Interventions<br>Spagnoletti, B. R. M., Bennett, L. R., Wahdi, A. E., Wilopo, S. A., Keenan, C. A.<br>Level: 2, State: Excluded	infection/serology/prevalence; cervical cancer; HPV vax safety)
1090	RefID: 1090, A qualitative study to assess the potential of the human papillomavirus vaccination programme to encourAge - Young adults 18-26yrs not included /outside age range under-screened mothers to attend for cervical screening<br>Spencer, A. M., Brabin, L., Roberts, S. A., Patnick, J., Elton, P., Verma, A.<br>Level: 1, State: Excluded	Wrong/no population (eg parents, providers, children, policy makers, does not include 18-26yrs olds; social media posts)
1091	RefID: 1091, An increase in HPV-related knowledge and vaccination intent among parental and non-parental caregivers of adolescent girls, Age - Young adults 18-26yrs not included /outside age range 9-17 years, in Appalachian Pennsylvania<br>Spleen, Angela, Kluhsman, Brenda, Clark, Allison, Dignan, Mark, Lengerich, Eugene, Spleen, Angela M., Kluhsman, Brenda C., Clark, Allison D., Dignan, Mark B., Lengerich, Eugene J.<br>Level: 1, State: Excluded	Wrong/no population (eg parents, providers, children, policy makers, does not include 18-26yrs olds; social media posts)
1094	RefID: 1094, Prevalence of High-risk Nonavalent Vaccine-type Human Papillomavirus Infection Among Unvaccinated, Sexually Active Asian Female Adolescents With and Without Perinatally Acquired HIV Infection<br>Sricharoenchai, S., Kerr, S. J., Gatechompol, S., Hansudewechakul, R., Dang, H. L. D., Tran, D. N. H., Teeratakulpisarn, N., Chalermchockcharoenkit, A., Achalapong, J., Teeraananchai, S., Singtoroj, T., Phanuphak, N., Sohn, A. H., Chokephaibulkit, K.<br>Level: 1, State: Excluded	Wrong/no population (eg parents, providers, children, policy makers, does not include 18-26yrs olds; social media posts)
1096	RefID: 1096, Vaccination differences among U.S. adults by their self-identified sexual orientation, National Health Interview Survey, 2013-2015<br>Srivastav, A., O'Halloran, A., Lu, P. J., Williams, W. W., Hutchins, S. S.<br>Level: 1, State: Excluded	Wrong/no population (eg parents, providers, children, policy makers, does not include 18-26yrs olds; social media posts)
1098	RefID: 1098, Differences in stakeholder-reported barriers and implementation strategies between counties with high, middle, and low HPV vaccine initiation rates: a mixed methods study<br>Staras, S. A. S., Kastrinos, A. L., Wollney, E. N., Desai, S., O'Neal, T. J., Johnson-Mallard, V., Bylund, C. L.<br>Level: 1, State: Excluded	Not about HPV vaccination/vax attitudes (eg HPV infection/serology/prevalence; cervical cancer; HPV vax safety)
1099	RefID: 1099, Ill-informed consent? A content analysis of physical risk disclosure in school-based HPV vaccine programs<br>Steenbeek, Audrey, MacDonald, Noni, Downie, Jocelyn, Appleton, Mary, Baylis, Françoise<br>Level: 1, State: Excluded	Study Design - Not qualitative (methods or analysis) OR Qualitative survey data not analyzed qualitatively (only numeric stats)
1101	RefID: 1101, Cervical cancer-related knowledge, attitudes, and practices of health professionals working in brazil's network of primary care units<br>Stormo, A. R., de Moura, L., Saraiya, M.<br>Level: 1, State: Excluded	Wrong/no population (eg parents, providers, children, policy makers, does not include 18-26yrs olds; social media posts)
1102	RefID: 1102, Self-efficacy and HPV Vaccine Attitudes Mediate the Relationship Between Social Norms and Intentions to Receive the HPV	Wrong/no population (eg parents, providers, children, policy makers,

	Vaccine Among College Students<br>Stout, M. E., Christy, S. M., Winger, J. G., Vadaparampil, S. T., Mosher, C. E.<br>Level: 1, State: Excluded	does not include 18-26yrs olds; social media posts)
1103	RefID: 1103, Utilization of Preventive Health Care in Adults and Children With Eczema<br>Strom, M. A., Silverberg, J. I.<br>Level: 1, State: Excluded	Wrong/no population (eg parents, providers, children, policy makers, does not include 18-26yrs olds; social media posts)
1104	RefID: 1104, Examining the Role of HPV Communication Training in the Knowledge, Attitudes, Comfort, and Confidence of Dental Hygiene Students<br>Stull, C. L., Matthews, E., Evans, M., Arnett, M. C.<br>Level: 1, State: Excluded	Study Design - Not qualitative (methods or analysis) OR Qualitative survey data not analyzed qualitatively (only numeric stats)
1105	RefID: 1105, Young adults' preferences for influenza vaccination campaign messAge - Young adults 18-26yrs not included /outside age ranges: Implications for COVID-19 vaccine intervention design and development<br>Su, Z., McDonnell, D., Wen, J., Cheshmehzangi, A., Ahmad, J., Goh, E., Li, X., Šegalo, S., Mackert, M., Xiang, Y. T., Wang, P.<br>Level: 1, State: Excluded	Not about HPV vaccination/vax attitudes (eg HPV infection/serology/prevalence; cervical cancer; HPV vax safety)
1106	RefID: 1106, Differences Regarding Knowledge of Sexually Transmitted Infections, Sexual Habits, and Behavior Between University Students of Medical and Nonmedical Professions in Serbia<br>Subotic, S., Vukomanovic, V., Djukic, S., Radevic, S., Radovanovic, S., Radulovic, D., Boricic, K., Andjelkovic, J., Tosic Pajic, J., Simic Vukomanovic, I.<br>Level: 1, State: Excluded	Study Design - Not qualitative (methods or analysis) OR Qualitative survey data not analyzed qualitatively (only numeric stats)
1108	RefID: 1108, Assessment of US Preventive Services Task Force Guideline-Concordant Cervical Cancer Screening Rates and Reasons for Underscreening by Age - Young adults 18-26yrs not included /outside age range, Race and Ethnicity, Sexual Orientation, Rurality, and Insurance, 2005 to 2019<br>Suk, R., Hong, Y. R., Rajan, S. S., Xie, Z., Zhu, Y., Spencer, J. C.<br>Level: 1, State: Excluded	Not about HPV vaccination/vax attitudes (eg HPV infection/serology/prevalence; cervical cancer; HPV vax safety)
1109	RefID: 1109, Three-year questionnaire survey on human papillomavirus vaccination targeting new female college students<br>Sukegawa, A., Ohshige, K., Arai, S., Sakanashi, K., Usui, M., Hirahara, F., Miyagi, E.<br>Level: 1, State: Excluded	Not about HPV vaccination/vax attitudes (eg HPV infection/serology/prevalence; cervical cancer; HPV vax safety)
1111	RefID: 1111, Ten-year questionnaire study on human papillomavirus vaccination targeting new female medical school students: Follow-up to the 2015 report<br>Sukegawa, A., Ohshige, K., Suzuki, Y., Mizushima, T., Ueda, Y., Sekine, M., Enomoto, T., Miyagi, E.<br>Level: 1, State: Excluded	Not about HPV vaccination/vax attitudes (eg HPV infection/serology/prevalence; cervical cancer; HPV vax safety)
1113	RefID: 1113, Rationale and design of the iPap trial: A randomized controlled trial of home-based HPV self-sampling for improving participation in cervical screening by never- and under-screened women in Australia<br>Sultana, F., English, D. R., Simpson, J. A., Brotherton, J. M. L., Drennan,	Study Design - Not qualitative (methods or analysis) OR Qualitative survey data not analyzed qualitatively (only numeric stats)

	K., Mullins, R., Heley, S., Wrede, C. D., Saville, M., Gertig, D. M.<br>Level: 1, State: Excluded	
1114	RefID: 1114, Extended surveillance to assess safety of 9-valent human papillomavirus vaccine<br>Sundaram, M. E., Kieke, B. A., Hanson, K. E., Belongia, E. A., Weintraub, E. S., Daley, M. F., Hechter, R. C., Klein, N. P., Lewis, E. M., Naleway, A. L., Nelson, J. C., Donahue, J. G.<br>Level: 1, State: Excluded	Not about HPV vaccination/vax attitudes (eg HPV infection/serology/prevalence; cervical cancer; HPV vax safety)
1115	RefID: 1115, Beyond the birds and the bees: a qualitative content analysis of online HPV vaccination communication<br>Sundstrom, B., Aylor, E., Cartmell, K. B., Brandt, H. M., Bryant, D. C., Halbert, C. H., Pierce, J. Y.<br>Level: 1, State: Excluded	Not about HPV vaccination/vax attitudes (eg HPV infection/serology/prevalence; cervical cancer; HPV vax safety)
1117	RefID: 1117, A reproductive justice approach to understanding women's experiences with HPV and cervical cancer prevention<br>Sundstrom, Beth, Smith, Ellie, Delay, Cara, Luque, John S., Davila, Caroline, Feder, Bailey, Paddock, Vincenza, Poudrier, Jessie, Pierce, Jennifer Young, Brandt, Heather M.<br>Level: 2, State: Excluded	Not about HPV vaccination/vax attitudes (eg HPV infection/serology/prevalence; cervical cancer; HPV vax safety)
1118	RefID: 1118, High Prevalence and Genotype Diversity of Anal HPV Infection among MSM in Northern Thailand<br>Supindham, T., Chariyalertsak, S., Utaipat, U., Miura, T., Ruanpeng, D., Chotirosniramit, N., Kosashunhanan, N., Sugandhavesa, P., Saokhieo, P., Songsupa, R., Siriaunkgul, S., Wongthanee, A.<br>Level: 1, State: Excluded	Wrong/no population (eg parents, providers, children, policy makers, does not include 18-26yrs olds; social media posts)
1119	RefID: 1119, A Modern Perspective on Vaccinating Healthcare Service Providers in India: A Narrative Review<br>Surendranath, M., Wankhedkar, R., Lele, J., Cintra, O., Kolhapure, S., Agrawal, A., Dewda, P.<br>Level: 1, State: Excluded	Multiple Wrong/no population (eg parents, providers, children, policy makers, does not include 18-26yrs olds; social media posts)s - unable to extract data specific to young adults (18-26 yrs)
1120	RefID: 1120, Stakeholder Perspectives of Australia's National HPV Vaccination Program<br>Swift, C., Dey, A., Rashid, H., Clark, K., Manocha, R., Brotherton, J., Beard, F.<br>Level: 1, State: Excluded	Not about HPV vaccination/vax attitudes (eg HPV infection/serology/prevalence; cervical cancer; HPV vax safety)
1121	RefID: 1121, Prevalence of Anal Human Papillomavirus Infection in Hungarian Men Who Have Sex with Men<br>Szabó, E., Kósa, C., Babarczi, E., Sulyok, M., Ujhelyi, E., Bánhegyi, D., Vályi-Nagy, I.<br>Level: 1, State: Excluded	Wrong publication type (eg reviews, opinions, conference proceedings)
1122	RefID: 1122, Efficacy of the HPV-16/18 AS04-adjuvanted vaccine against low-risk HPV types (PATRICIA randomized trial): an unexpected observation<br>Szarewski, A., Skinner, S. R., Garland, S. M., Romanowski, B., Schwarz, T. F., Apter, D., Chow, SongNan, Paavonen, J., Rosario-Raymundo, M. R. del, Teixeira, J. C., Carvalho, N. S. de, Castro-Sanchez, M., Castellsagué, X., Poppe, W. A. J., Sutter, P. de, Huh, W., Chatterjee, A., Tjalma, W. A., Ackerman, R. T., Martens, M., Papp, K. A., Bajo-Arenas, J., Harper, D.	Wrong/no population (eg parents, providers, children, policy makers, does not include 18-26yrs olds; social media posts)

	M., Torné, A., David, M. P., Struyf, F.<br>Level: 1, State: Excluded	
1127	RefID: 1127, Knowledge and Attitude Toward Human Papillomavirus and HPV Vaccination in Iranian Wrong/no population (eg parents, providers, children, policy makers, does not include 18-26yrs olds; social media posts): A Systematic Review<br>Taebi, M., Riazi, H., Keshavarz, Z., Afrakhteh, M.<br>Level: 1, State: Excluded	Not about HPV vaccination/vax attitudes (eg HPV infection/serology/prevalence; cervical cancer; HPV vax safety)
1128	RefID: 1128, Communication skills in HPV prevention: An audit among Italian healthcare workers<br>Tafuri, S., Martinelli, D., Vece, M. M., Quarto, M., Germinario, C., Prato, R.<br>Level: 1, State: Excluded	Not about HPV vaccination/vax attitudes (eg HPV infection/serology/prevalence; cervical cancer; HPV vax safety)
1129	RefID: 1129, Distribution of Human Papillomavirus Genotypes among Women in Mashhad, Iran<br>Taghizadeh, E., Taheri, F., Abdolkarimi, H., Ghorbani Renani, P., Gheibi Hayat, S. M.<br>Level: 1, State: Excluded	Wrong publication type (eg reviews, opinions, conference proceedings)
1130	RefID: 1130, Peri-conceptional or pregnancy exposure of HPV vaccination and the risk of spontaneous abortion: a systematic review and meta-analysis<br>Tan, J., Xiong, Y. Q., He, Q., Liu, Y. M., Wang, W., Chen, M., Zou, K., Liu, X. H., Sun, X.<br>Level: 1, State: Excluded	Wrong/no population (eg parents, providers, children, policy makers, does not include 18-26yrs olds; social media posts)
1131	RefID: 1131, Disparity of cervical cancer risk in young japanese women: Bipolarized status of hpv vaccination and cancer screening<br>Taniguchi, M., Ueda, Y., Yagi, A., Miyoshi, A., Tanaka, Y., Minekawa, R., Endo, M., Tomimatsu, T., Hirai, K., Nakayama, T., Kimura, T.<br>Level: 1, State: Excluded	Not about HPV vaccination/vax attitudes (eg HPV infection/serology/prevalence; cervical cancer; HPV vax safety)
1132	RefID: 1132, California State University Fullerton and the University of California Irvine Chao family comprehensive cancer center partnership for cancer health disparities research<br>Tanjasiri, S. P., Allan Hubbell, F.<br>Level: 1, State: Excluded	Wrong publication type (eg reviews, opinions, conference proceedings)
1133	RefID: 1133, High-Risk Human Papillomavirus (HPV) Infection and Cervical Cancer Prevention in Britain: Evidence of Differential Uptake of Interventions from a Probability Survey<br>Tanton, C., Soldan, K., Beddows, S., Mercer, C. H., Waller, J., Field, N., Clifton, S., Copas, A. J., Panwar, K., Manyenga, P., da Silva, F., Wellings, K., Ison, C. A., Johnson, A. M., Sonnenberg, P.<br>Level: 1, State: Excluded	Not about HPV vaccination/vax attitudes (eg HPV infection/serology/prevalence; cervical cancer; HPV vax safety)
1134	RefID: 1134, The clinical and economic benefits of school-based quadrivalent HPV vaccination in Singapore<br>Tay, S. K., Hsu, T. Y., Shcheprov, A., Walia, A., Kulkarni, A. S.<br>Level: 1, State: Excluded	Not about HPV vaccination/vax attitudes (eg HPV infection/serology/prevalence; cervical cancer; HPV vax safety)
1135	RefID: 1135, HPV vaccination uptake among Cambodian mothers<br>Taylor, Victoria, Burke, Nancy, Do, Hoai, Liu, Qi, Yasui, Yutaka, Bastani, Roshan, Taylor, Victoria M.<br>Level: 1, State: Excluded	Age - Young adults 18-26yrs not included /outside age range

1136	RefID: 1136, Immunizations within the context of juvenile detention<br>Teck, J., Beyda, R. M., Eissa, M., Benjamins, L.<br>Level: 1, State: Excluded	Age - Young adults 18-26yrs not included /outside age range
1138	RefID: 1138, Social cognitive and clinical factors associated with HPV vaccine initiation among urban, economically disadvantAge - Young adults 18-26yrs not included /outside age ranged women<br>Teitelman, A. M., Stringer, M., Nguyen, G. T., Hanlon, A. L., Averbuch, T., Stimpfel, A. W.<br>Level: 2, State: Excluded	Wrong/no population (eg parents, providers, children, policy makers, does not include 18-26yrs olds; social media posts)
1139	RefID: 1139, The epidemiologic and economic impact of a quadrivalent human papillomavirus vaccine in Thailand<br>Termrungruanglert, W., Khemapech, N., Vasuratna, A., Havanond, P., Deebukkham, P., Kulkarni, A. S., Pavelyev, A.<br>Level: 1, State: Excluded	Not about HPV vaccination/vax attitudes (eg HPV infection/serology/prevalence; cervical cancer; HPV vax safety)
1140	RefID: 1140, Impact of HPV vaccination on outcome of cervical cytology screening in Denmark-A register-based cohort study<br>Thamsborg, L. H., Napolitano, G., Larsen, L. G., Lynge, E.<br>Level: 1, State: Excluded	
1142	RefID: 1142, Factors influencing parental decision making for the human papillomavirus (HPV) vaccine: A literature review<br>Thavarajah, Nemica, Chow, Edward, Arocha, Jose<br>Level: 1, State: Excluded	Multiple Wrong/no population (eg parents, providers, children, policy makers, does not include 18-26yrs olds; social media posts)s - unable to extract data specific to young adults (18-26 yrs)
1143	RefID: 1143, The Influence of Religiosity and Spirituality on Rural Parents' Health Decision Making and Human Papillomavirus Vaccine Choices<br>Thomas, T., Blumling, A., Delaney, A.<br>Level: 1, State: Excluded	Age - Young adults 18-26yrs not included /outside age range
1144	RefID: 1144, Educating Latinas about cervical cancer and HPV: a pilot randomized study<br>Thompson, B., Barrington, W. E., Briant, K. J., Kupay, E., Carosso, E., Gonzalez, N. E., Gonzalez, V. J.<br>Level: 1, State: Excluded	Age - Young adults 18-26yrs not included /outside age range
1145	RefID: 1145, The influence of relationship status on HPV vaccine decision-making among young adult women<br>Thompson, Erika L.<br>Level: 2, State: Excluded	Wrong publication type (eg reviews, opinions, conference proceedings)
1146	RefID: 1146, "My mom said it wasn't important": A case for catch-up human papillomavirus vaccination among young adult women in the United States<br>Thompson, E. L., Best, A. L., Vamos, C. A., Daley, E. M.<br>Level: 1, State: Excluded	Wrong/no population (eg parents, providers, children, policy makers, does not include 18-26yrs olds; social media posts)
1147	RefID: 1147, Social Determinants of Health and Human Papillomavirus Vaccination Among Young Adults, National Health Interview Survey 2016<br>Thompson, E. L., Rosen, B. L., Maness, S. B.<br>Level: 1, State: Excluded	Not about HPV vaccination/vax attitudes (eg HPV infection/serology/prevalence; cervical cancer; HPV vax safety)

1149	RefID: 1149, Relationship status impacts primary reasons for interest in the HPV vaccine among young adult women<br>Thompson, E. L., Vamos, C. A., Sappenfield, W. M., Straub, D. M., Daley, E. M.<br>Level: 1, State: Excluded	studytype
1152	RefID: 1152, Prevalence and distribution of cervical high-risk human papillomavirus and cytological abnormalities in women living with HIV in Denmark - the SHADE<br>Thorsteinsson, K., Storgaard, M., Katzenstein, T. L., Ladelund, S., Rønsholt, F. F., Johansen, I. S., Pedersen, G., Hashemi, L., Nielsen, L. N., Nilas, L., Obel, N., Bonde, J., Lebech, A. M.<br>Level: 1, State: Excluded	Wrong/no population (eg parents, providers, children, policy makers, does not include 18-26yrs olds; social media posts)
1154	RefID: 1154, Challenges of health promotion and education strategies to prevent cervical cancer in India: A systematic review<br>Thulaseedharan, J. V., Frie, K. G., Sankaranarayanan, R.<br>Level: 1, State: Excluded	Study Design - Not qualitative (methods or analysis) OR Qualitative survey data not analyzed qualitatively (only numeric stats)
1155	RefID: 1155, Prepared: Implementation of a pre-exposure prophylaxis (PrEP) program in a hospital-based HIV clinic<br>Tiberio, P. J., Williams, K., Barakat, L. A., Edelman, E. J., Virata, M., Ogbuagu, O.<br>Level: 1, State: Excluded	Study Design - Not qualitative (methods or analysis) OR Qualitative survey data not analyzed qualitatively (only numeric stats)
1156	RefID: 1156, Human papillomavirus vaccine use among adolescent girls and young adult women: an analysis of the 2007 California Health Interview Survey<br>Tiro, J. A., Tsui, J., Bauer, H. M., Yamada, E., Kobrin, S., Breen, N.<br>Level: 1, State: Excluded	Not about HPV vaccination/vax attitudes (eg HPV infection/serology/prevalence; cervical cancer; HPV vax safety)
1157	RefID: 1157, Immunological response to quadrivalent HPV vaccine in treatment of recurrent respiratory papillomatosis<br>Tjon Pian Gi, R. E. A., San Giorgi, M. R. M., Pawlita, M., Michel, A., van Hemel, B. M., Schuuring, E. M. D., van den Heuvel, E. R., van der Laan, Bfam, Dikkers, F. G.<br>Level: 1, State: Excluded	Wrong publication type (eg reviews, opinions, conference proceedings)
1159	RefID: 1159, Parental knowledge, views, and perceptions of human papilloma virus infection and vaccination-cross-sectional descriptive study<br>Tobaiqy, M. A., Mehdar, S. A., Altayeb, T. I., Saad, T. M., Alqutub, S. T.<br>Level: 1, State: Excluded	Not about HPV vaccination/vax attitudes (eg HPV infection/serology/prevalence; cervical cancer; HPV vax safety)
1160	RefID: 1160, Alaska Native parental attitudes on cervical cancer, HPV and the HPV vaccine<br>Toffolon-Weiss, M., Hagan, K., Leston, J., Peterson, L., Provost, E., Hennessy, T.<br>Level: 1, State: Excluded	Study Design - Not qualitative (methods or analysis) OR Qualitative survey data not analyzed qualitatively (only numeric stats)
1161	RefID: 1161, Investigation on spontaneous abortion and human papillomavirus infection<br>Tognon, M., Tagliapietra, A., Magagnoli, F., Mazziotta, C., Oton-Gonzalez, L., Lanzillotti, C., Vesce, F., Contini, C., Rotondo, J. C., Martini, F.<br>Level: 1, State: Excluded	Not about HPV vaccination/vax attitudes (eg HPV infection/serology/prevalence; cervical cancer; HPV vax safety)

1162	RefID: 1162, Mmr vaccine attitude and uptake research in the united kingdom: A critical review<br>Torracinta, L., Tanner, R., Vanderslott, S.<br>Level: 1, State: Excluded	Study Design - Not qualitative (methods or analysis) OR Qualitative survey data not analyzed qualitatively (only numeric stats)
1163	RefID: 1163, Evaluating medical and dental students'human papillomavirus-related cancer knowledge andperceived self-efficacy in HPV vaccine communicationand recommendation practices<br>Torres, E., Richman, A., Wright, W., Eldridge, D., Lawson, L.<br>Level: 1, State: Excluded	Wrong/no population (eg parents, providers, children, policy makers, does not include 18-26yrs olds; social media posts)
1164	RefID: 1164, Evaluation of Type Replacement Following HPV16/18 Vaccination: pooled Analysis of Two Randomized Trials<br>Tota, J. E., Struyf, F., Merikukka, M., Gonzalez, P., Kreimer, A. R., Bi, D., Castellsagué, X., de Carvalho, N. S., Garland, S. M., Harper, D. M., et al.<br>Level: 1, State: Excluded	Study Design - Not qualitative (methods or analysis) OR Qualitative survey data not analyzed qualitatively (only numeric stats)
1168	RefID: 1168, Knowledge, attitudes, and practices toward cervical cancer prevention among women in Kampong Speu Province, Cambodia<br>Touch, Sothy, Oh, Jin-Kyoung<br>Level: 1, State: Excluded	Wrong publication type (eg reviews, opinions, conference proceedings)
1170	RefID: 1170, New quadrivalent HPV vaccine developments<br>Tovar, J. M., Bazaldua, O. V.<br>Level: 1, State: Excluded	Study Design - Not qualitative (methods or analysis) OR Qualitative survey data not analyzed qualitatively (only numeric stats)
1171	RefID: 1171, Comparison of Quality of Internet PAge - Young adults 18-26yrs not included /outside age ranges on Human Papillomavirus Immunization in Italian and in English<br>Tozzi, A. E., Buonuomo, P. S., Ciofi degli Atti, M. L., Carloni, E., Meloni, M., Gamba, F.<br>Level: 1, State: Excluded	Not about HPV vaccination/vax attitudes (eg HPV infection/serology/prevalence; cervical cancer; HPV vax safety)
1172	RefID: 1172, HPV vaccination hesitancy in Reunion Island<br>Tran, P. L., Bruneteaux, A., Lazaro, G., Antoine, B., Malik, B.<br>Level: 1, State: Excluded	Not about HPV vaccination/vax attitudes (eg HPV infection/serology/prevalence; cervical cancer; HPV vax safety)
1173	RefID: 1173, Understanding barriers and motivations to papillomavirus vaccination in a middle school in Reunion Island<br>Tran, P. L., Leruste, S., Sitthisone, J., Humbert, M., Gilhard, X., Lazaro, G., Chirpaz, E., Boukerrou, M., Bertolotti, A.<br>Level: 1, State: Excluded	Wrong publication type (eg reviews, opinions, conference proceedings)
1174	RefID: 1174, Response to vaccination against Mumps in medical students: Two doses are needed<br>Trevisan, A., Moretto, A., Bertoncello, C., Nicolli, A., Maso, S., Scapellato, M. L., Mason, P.<br>Level: 1, State: Excluded	Wrong/no population (eg parents, providers, children, policy makers, does not include 18-26yrs olds; social media posts)
1175	RefID: 1175, Safety, efficacy, and immunogenicity of VGX-3100, a therapeutic synthetic DNA vaccine targeting human papillomavirus 16 and 18 E6 and E7 proteins for cervical intraepithelial neoplasia 2/3: a randomised, double-blind, placebo-controlled phase 2b trial<br>Trimble, C. L., Morrow, M. P., Kraynyak, K. A., Shen, X., Dallas, M., Yan,	Study Design - Not qualitative (methods or analysis) OR Qualitative survey data not analyzed qualitatively (only numeric stats)

	J., Edwards, L., Parker, R. L., Denny, L., Giffear, M., et al.<br>Level: 1, State: Excluded	
1176	RefID: 1176, Understanding primary care physician perspectives on recommending HPV vaccination and addressing vaccine hesitancy<br>Tsui, J., Vincent, A., Anuforo, B., Btoush, R., Crabtree, B. F.<br>Level: 1, State: Excluded	Wrong/no population (eg parents, providers, children, policy makers, does not include 18-26yrs olds; social media posts)
1178	RefID: 1178, Perceptions of human papillomavirus vaccination of adolescent schoolgirls in western Uganda and their implications for acceptability of HPV vaccination: a qualitative study<br>Turiho, A. K., Okello, E. S., Muhwezi, W. W., Katahoire, A. R.<br>Level: 2, State: Excluded	Not about HPV vaccination/vax attitudes (eg HPV infection/serology/prevalence; cervical cancer; HPV vax safety)
1179	RefID: 1179, Prevalence of anal, oral, penile and urethral Human Papillomavirus in HIV infected and HIV uninfected men who have sex with men<br>Ucciferri, C., Tamburro, M., Falasca, K., Sammarco, M. L., Ripabelli, G., Vecchiet, J.<br>Level: 1, State: Excluded	Study Design - Not qualitative (methods or analysis) OR Qualitative survey data not analyzed qualitatively (only numeric stats)
1180	RefID: 1180, Human papillomavirus vaccination among adolescents in Georgia<br>Underwood, N. L., Weiss, P., Gargano, L. M., Seib, K., Rask, K. J., Morfaw, C., Murray, D., DiClemente, R. J., Hughes, J. M., Sales, J. M.<br>Level: 1, State: Excluded	Wrong/no population (eg parents, providers, children, policy makers, does not include 18-26yrs olds; social media posts)
1182	RefID: 1182, Knowdlege and preventive behaviors related to cervical cancer and human papiloma virus in a group of Chilean adolescents<br>Urrutia, M. T., Concha, X., Riquelme, G., Padilla, O.<br>Level: 1, State: Excluded	Wrong/no population (eg parents, providers, children, policy makers, does not include 18-26yrs olds; social media posts)
1183	RefID: 1183, Knowledge, Attitude, and Practice Toward Cervical Cancer Screening Among Female University Students in Ishaka Western Uganda<br>Usman, I. M., Chama, N., Aigbogun, E. O., Jr., Kabanyoro, A., Kasozi, K. I., Usman, C. O., Fernandez Diaz, M. E., Ndyamuhakyi, E., Archibong, V. B., Onongha, C., Ochieng, J. J., Kanee, R. B., Ssebuufu, R.<br>Level: 1, State: Excluded	Not about HPV vaccination/vax attitudes (eg HPV infection/serology/prevalence; cervical cancer; HPV vax safety)
1184	RefID: 1184, High prevalence of human papillomavirus infection in the female Wrong/no population (eg parents, providers, children, policy makers, does not include 18-26yrs olds; social media posts) of Guatemala<br>Vallès, X., Murga, G. B., Hernández, G., Sabidó, M., Chuy, A., Lloveras, B., Alameda, F., de San José, S., Bosch, F. X., Pedroza, I., Castellsagué, X., Casabona, J.<br>Level: 1, State: Excluded	Study Design - Not qualitative (methods or analysis) OR Qualitative survey data not analyzed qualitatively (only numeric stats)
1185	RefID: 1185, LINDA - a solution-focused low-intensity intervention aimed at improving health behaviors of young females: a cluster-randomized controlled trial<br>Valve, P., Lehtinen-Jacks, S., Eriksson, T., Lehtinen, M., Lindfors, P., Saha, M. T., Rimpelä, A., Anglé, S.<br>Level: 1, State: Excluded	Study Design - Not qualitative (methods or analysis) OR Qualitative survey data not analyzed qualitatively (only numeric stats)
1186	RefID: 1186, Health literacy needs and preferences for a technology-based intervention to improve college students' sexual and	Study Design - Not qualitative (methods or analysis) OR

	reproductive health<br>Vamos, C. A., Puccio, J. A., Griner, S. B., Logan, R. G., Piepenbrink, R., Richardson Cayama, M., Lovett, S. M., Mahony, H., Daley, E. M.<br>Level: 2, State: Excluded	Qualitative survey data not analyzed qualitatively (only numeric stats)
1187	RefID: 1187, HPV-related risk perceptions and HPV vaccine uptake among a sample of young rural women<br>Vanderpool, R. C., Casey, B. R., Crosby, R. A.<br>Level: 1, State: Excluded	Study Design - Not qualitative (methods or analysis) OR Qualitative survey data not analyzed qualitatively (only numeric stats)
1188	RefID: 1188, Correlation of HPV vaccination status and sexual behavior: a study of adolescent and young women in Greece<br>Vatopoulou, A., Theodoridise, T., Athanasiadis, L., Papanikolaou, A., Tarlatzis, B., Agorastos, T.<br>Level: 1, State: Excluded	Not about HPV vaccination/vax attitudes (eg HPV infection/serology/prevalence; cervical cancer; HPV vax safety)
1189	RefID: 1189, Individualism, acceptance and differentiation as attitude traits in the public's response to vaccination<br>Velan, B., Boyko, V., Lerner-Geva, L., Ziv, A., Yagar, Y., Kaplan, G.<br>Level: 1, State: Excluded	Not about HPV vaccination/vax attitudes (eg HPV infection/serology/prevalence; cervical cancer; HPV vax safety)
1190	RefID: 1190, Identification of HPV genotypes causing cervical precancer using tissue-based genotyping<br>Venetianer, R., Clarke, M. A., van der Marel, J., Tota, J., Schiffman, M., Dunn, S. T., Walker, J., Zuna, R., Quint, W., Wentzensen, N.<br>Level: 1, State: Excluded	Study Design - Not qualitative (methods or analysis) OR Qualitative survey data not analyzed qualitatively (only numeric stats)
1191	RefID: 1191, Parents' and providers' attitudes toward school-located provision and school-entry requirements for HPV vaccines<br>Vercruysse, J., Chigurupati, N. L., Fung, L., Apte, G., Pierre-Joseph, N., Perkins, R. B.<br>Level: 1, State: Excluded	Study Design - Not qualitative (methods or analysis) OR Qualitative survey data not analyzed qualitatively (only numeric stats)
1192	RefID: 1192, Dose-related Effectiveness of Quadrivalent Human Papillomavirus Vaccine against Cervical Intraepithelial Neoplasia: A Danish Nationwide Cohort Study<br>Verdoodt, F., Dehlendorff, C., Kjaer, S. K.<br>Level: 1, State: Excluded	Wrong/no population (eg parents, providers, children, policy makers, does not include 18-26yrs olds; social media posts)
1193	RefID: 1193, Implementation of an HPV vaccination program in Eldoret, Kenya: results from a qualitative assessment by key stakeholders<br>Vermandere, H., Naanyu, V., Degomme, O., Michielsen, K.<br>Level: 1, State: Excluded	Not about HPV vaccination/vax attitudes (eg HPV infection/serology/prevalence; cervical cancer; HPV vax safety)
1195	RefID: 1195, Human papillomavirus infections: influence of perceptions of disease and vaccine on immunization status<br>Verrier, F., Gautier, A., Quelet, S., Bonmarin, I.<br>Level: 1, State: Excluded	Wrong/no population (eg parents, providers, children, policy makers, does not include 18-26yrs olds; social media posts)
1196	RefID: 1196, Papillomavirus Infection and Prevention: How Much Does the Sicilian Wrong/no population (eg parents, providers, children, policy makers, does not include 18-26yrs olds; social media posts) Know? An Observational Study<br>Verro, B., Gallina, S., Saraniti, C.<br>Level: 1, State: Excluded	Not about HPV vaccination/vax attitudes (eg HPV infection/serology/prevalence; cervical cancer; HPV vax safety)
1197	RefID: 1197, Parental knowledge gaps and barriers for children receiving human papillomavirus vaccine in the Rio Grande Valley of	Wrong/no population (eg parents, providers, children, policy makers,

	Texas<br>Victory, M., Do, T. Q. N., Kuo, Y. F., Rodriguez, A. M.<br>Level: 1, State: Excluded	does not include 18-26yrs olds; social media posts)
1199	RefID: 1199, Genital Tract Infections in an Isolated Community: 100 Women of the Príncipe Island<br>Vieira-Baptista, P., Grinceviciene, S., Bellen, G., Sousa, C., Saldanha, C., Broeck, D. V., Bogers, J. P., Donders, G.<br>Level: 1, State: Excluded	Study Design - Not qualitative (methods or analysis) OR Qualitative survey data not analyzed qualitatively (only numeric stats)
1200	RefID: 1200, Summary of the evidence on the safety, efficacy, and effectiveness of human papillomavirus vaccines: Umbrella review of systematic reviews<br>Villa, Alessandro, Patton, Lauren L., Giuliano, Anna R., Estrich, Cameron G., Pahlke, Sarah C., O'Brien, Kelly K., Lipman, Ruth D., Araujo, Marcelo W. B.<br>Level: 1, State: Excluded	Not about HPV vaccination/vax attitudes (eg HPV infection/serology/prevalence; cervical cancer; HPV vax safety)
1201	RefID: 1201, Estimating seroprevalence of human papillomavirus type 16 using a mixture model with smoothed Age - Young adults 18-26yrs not included /outside age range-dependent mixing proportions<br>Vink, M. A., van de Kassteele, J., Wallinga, J., Teunis, P. F., Bogaards, J. A.<br>Level: 1, State: Excluded	Study Design - Not qualitative (methods or analysis) OR Qualitative survey data not analyzed qualitatively (only numeric stats)
1203	RefID: 1203, Knowledge, attitudes and practices amongst female university students in Vietnam: A cross-sectional study on human papillomavirus vaccination<br>Vo, T. Q.<br>Level: 1, State: Excluded	Not about HPV vaccination/vax attitudes (eg HPV infection/serology/prevalence; cervical cancer; HPV vax safety)
1204	RefID: 1204, Prevalence of cervical infection with HPV type 16 and 18 in Vietnam: implications for vaccine campaign<br>Vu, L. T., Bui, D., Le, H. T.<br>Level: 1, State: Excluded	Wrong publication type (eg reviews, opinions, conference proceedings)
1207	RefID: 1207, A systematic review of practice-, provider-, and patient-level determinants impacting Asian-Americans' human papillomavirus vaccine intention and uptake<br>Vu, M., Berg, C. J., Escoffery, C., Jang, H. M., Nguyen, T. T., Travis, L., Bednarczyk, R. A.<br>Level: 1, State: Excluded	Not about HPV vaccination/vax attitudes (eg HPV infection/serology/prevalence; cervical cancer; HPV vax safety)
1208	RefID: 1208, [Evolution of the awareness of Human Papillomavirus (HPV) in the French Wrong/no population (eg parents, providers, children, policy makers, does not include 18-26yrs olds; social media posts): Results of a telephonic inquiry]<br>Wafo, E., Ivorra-Deleuze, D., Thuillier, C., Rouzier, R.<br>Level: 1, State: Excluded	Wrong publication type (eg reviews, opinions, conference proceedings)
1209	RefID: 1209, Parents, adolescents, children and the human papillomavirus vaccine: a review<br>Walhart, T.<br>Level: 1, State: Excluded	Not about HPV vaccination/vax attitudes (eg HPV infection/serology/prevalence; cervical cancer; HPV vax safety)
1210	RefID: 1210, Attitudes, practices, and beliefs about human papillomavirus vaccine among young adult African-American women: Implications for effective implementation	Wrong publication type (eg reviews, opinions, conference proceedings)

	Walker, Chastity L.<br>Level: 2, State: Excluded	
1211	RefID: 1211, "We fear the unknown": Emergence, route and transfer of hesitancy and misinformation among HPV vaccine accepting mothers<br>Walker, K. K., Owens, H., Zimet, G.<br>Level: 1, State: Excluded	Not about HPV vaccination/vax attitudes (eg HPV infection/serology/prevalence; cervical cancer; HPV vax safety)
1212	RefID: 1212, Public knowledge and attitudes towards Human Papilloma Virus (HPV) vaccination<br>Walsh, C. D., Gera, A., Shah, M., Sharma, A., Powell, J. E., Wilson, S.<br>Level: 2, State: Excluded	Wrong publication type (eg reviews, opinions, conference proceedings)
1213	RefID: 1213, The use of Web-based interactive technology to promote HPV vaccine uptake among young females: a randomized controlled trial<br>Wang, Q., Zhang, W.<br>Level: 1, State: Excluded	studytype
1214	RefID: 1214, Hospital-based prevalence of high-risk cervical HPV types infecting the general Wrong/no population (eg parents, providers, children, policy makers, does not include 18-26yrs olds; social media posts) and female sex workers in Huzhou, China<br>Wang, X., Gu, D., Lou, B., Xu, B., Qian, F., Chen, Y.<br>Level: 1, State: Excluded	Wrong/no population (eg parents, providers, children, policy makers, does not include 18-26yrs olds; social media posts)
1216	RefID: 1216, Effectiveness of a community-based organization—private clinic service model in promoting human papillomavirus vaccination among chinese men who have sex with men<br>Wang, Z., Fang, Y., Chan, P. S. F., Chidgey, A., Fong, F., Ip, M., Lau, J. T. F.<br>Level: 1, State: Excluded	Study Design - Not qualitative (methods or analysis) OR Qualitative survey data not analyzed qualitatively (only numeric stats)
1217	RefID: 1217, Facilitators and barriers to take up clinician-collected and self-collected hpv tests among chinese men who have sex with men<br>Wang, Z., Fang, Y., Wong, N. S., Ip, M., Guo, X., Wong, S. Y. S.<br>Level: 1, State: Excluded	Not about HPV vaccination/vax attitudes (eg HPV infection/serology/prevalence; cervical cancer; HPV vax safety)
1218	RefID: 1218, Two Web-Based and Theory-Based Interventions With and Without Brief Motivational Interviewing in the Promotion of Human Papillomavirus Vaccination Among Chinese Men Who Have Sex With Men: Randomized Controlled Trial<br>Wang, Z., Lau, J. T. F., Ip, T. K. M., Yu, Y., Fong, F., Fang, Y., Mo, P. K. H.<br>Level: 1, State: Excluded	Not about HPV vaccination/vax attitudes (eg HPV infection/serology/prevalence; cervical cancer; HPV vax safety)
1220	RefID: 1220, Acceptability of HPV vaccines and perceptions related to genital warts and penile/anal cancers among men who have sex with men in Hong Kong<br>Wang, Z., Mo, P. K., Lau, J. T., Lau, M., Lai, C. H.<br>Level: 1, State: Excluded	Study Design - Not qualitative (methods or analysis) OR Qualitative survey data not analyzed qualitatively (only numeric stats)
1222	RefID: 1222, Uptake and correlates of cervical cancer screening among HIV-infected women attending HIV care in Uganda<br>Wanyenze, R. K., Bwanika, J. B., Beyeza-Kashesya, J., Mugerwa, S., Arinaitwe, J., Matovu, J. K. B., Gwokyalya, V., Kasozi, D., Bukenya, J., Makumbi, F.<br>Level: 1, State: Excluded	Study Design - Not qualitative (methods or analysis) OR Qualitative survey data not analyzed qualitatively (only numeric stats)
1223	RefID: 1223, A cluster analysis of serious adverse event reports after human papillomavirus (HPV) vaccination in Danish girls and young	Study Design - Not qualitative (methods or analysis) OR

	women, September 2009 to August 2017<br>Ward, D., Thorsen, N. M., Frisch, M., Valentiner-Branth, P., Mølbak, K., Hviid, A.<br>Level: 1, State: Excluded	Qualitative survey data not analyzed qualitatively (only numeric stats)
1224	RefID: 1224, Latino Parents' Perceptions of the HPV Vaccine for Sons and Daughters<br>Warner, E. L., Lai, D., Carbajal-Salisbury, S., Garza, L., Bodson, J., Mooney, K., Kepka, D.<br>Level: 1, State: Excluded	Study Design - Not qualitative (methods or analysis) OR Qualitative survey data not analyzed qualitatively (only numeric stats)
1225	RefID: 1225, Awareness and Knowledge of HPV, HPV Vaccination, and Cervical Cancer among an Indigenous Caribbean Community<br>Warner, Z. C., Reid, B., Auguste, P., Joseph, W., Kepka, D., Warner, E. L.<br>Level: 1, State: Excluded	Not about HPV vaccination/vax attitudes (eg HPV infection/serology/prevalence; cervical cancer; HPV vax safety)
1227	RefID: 1227, Exploring HPV awareness and understanding before and after health education<br>Watson, Claudia, Serrant-Green, Laura<br>Level: 1, State: Excluded	Not about HPV vaccination/vax attitudes (eg HPV infection/serology/prevalence; cervical cancer; HPV vax safety)
1228	RefID: 1228, Access and Attitudes to HPV Vaccination amongst Hard-To-Reach Wrong/no population (eg parents, providers, children, policy makers, does not include 18-26yrs olds; social media posts)s in Kenya<br>Watson-Jones, D., Mugo, N., Lees, S., Mathai, M., Vusha, S., Ndirangu, G., Ross, D. A.<br>Level: 1, State: Excluded	Wrong/no population (eg parents, providers, children, policy makers, does not include 18-26yrs olds; social media posts)
1229	RefID: 1229, Sex Differences in the Incidence and Clearance of Anogenital Human Papillomavirus Infection in Liuzhou, China: An Observational Cohort Study<br>Wei, F., Guo, M., Huang, S., Li, M., Cui, X., Su, Y., Wu, X., Ma, X., Zheng, Y., Huang, Y., Wang, L., Pan, L., Wu, T., Zhang, J., Xia, N.<br>Level: 1, State: Excluded	Study Design - Not qualitative (methods or analysis) OR Qualitative survey data not analyzed qualitatively (only numeric stats)
1230	RefID: 1230, Geographic variability in human papillomavirus vaccination among U.S. young women<br>Wei, F., Moore, P. C., Green, A. L.<br>Level: 1, State: Excluded	
1232	RefID: 1232, Sex differences in the incidence and clearance of anal human papillomavirus infection among heterosexual men and women in Liuzhou, China: An observational cohort study<br>Wei, F., Su, Y., Yao, X., Cui, X., Bian, L., Yin, K., Yu, X., Zhuang, C., Bi, Z., Huang, S., Li, M., Wu, T., Xia, N., Zhang, J.<br>Level: 1, State: Excluded	Age - Young adults 18-26yrs not included /outside age range
1234	RefID: 1234, Age - Young adults 18-26yrs not included /outside age range distribution of human papillomavirus infection and neutralizing antibodies in healthy Chinese women Age - Young adults 18-26yrs not included /outside age ranged 18-45 years enrolled in a clinical trial<br>Wei, L. H., Su, Y. Y., Hu, Y. M., Li, R. C., Chen, W., Pan, Q. J., Zhang, X., Zhao, F. H., Zhao, Y. Q., Li, Q., et al.<br>Level: 1, State: Excluded	Wrong/no population (eg parents, providers, children, policy makers, does not include 18-26yrs olds; social media posts)
1235	RefID: 1235, POP-Brazil study protocol: a nationwide cross-sectional evaluation of the prevalence and genotype distribution of human papillomavirus (HPV) in Brazil	Not about HPV vaccination/vax attitudes (eg HPV

	Wendland, E. M., Caierão, J., Domingues, C., Maranhão, A. G. K., de Souza, F. M. A., Hammes, L. S., Falavigna, M., Hilgert, J. B., Hugo, F. N., Bessel, M., Villa, L. L., Benzaken, A. S.<br>Level: 1, State: Excluded	infection/serology/prevalence; cervical cancer; HPV vax safety)
1236	RefID: 1236, Effectiveness of a universal vaccination program with an HPV quadrivalent vaccine in young Brazilian women<br>Wendland, E. M., Kops, N. L., Bessel, M., Comerlato, J., Maranhão, A. G. K., Souza, F. M. A., Villa, L. L., Pereira, G. F. M.<br>Level: 1, State: Excluded	Study Design - Not qualitative (methods or analysis) OR Qualitative survey data not analyzed qualitatively (only numeric stats)
1239	RefID: 1239, Health care provider use of motivational interviewing to address vaccine hesitancy in college students<br>Wermers, R., Ostroski, T., Hagler, D.<br>Level: 1, State: Excluded	Not about HPV vaccination/vax attitudes (eg HPV infection/serology/prevalence; cervical cancer; HPV vax safety)
1240	RefID: 1240, A Technology-Mediated Behavioral Weight Gain Prevention Intervention for College Students: controlled, Quasi-Experimental Study<br>West, D. S., Monroe, C. M., Turner-McGrievy, G., Sundstrom, B., Larsen, C., Magradey, K., Wilcox, S., Brandt, H. M.<br>Level: 1, State: Excluded	Not about HPV vaccination/vax attitudes (eg HPV infection/serology/prevalence; cervical cancer; HPV vax safety)
1241	RefID: 1241, Engaging parents and schools improves uptake of the human papillomavirus (HPV) vaccine: Examining the role of the public health nurse<br>Whelan, N. W., Steenbeek, A., Martin-Misener, R., Scott, J., Smith, B., D'Angelo-Scott, H.<br>Level: 1, State: Excluded	Not about HPV vaccination/vax attitudes (eg HPV infection/serology/prevalence; cervical cancer; HPV vax safety)
1243	RefID: 1243, Prevalence of Modifiable Cancer Risk Factors Among U.S. Adults Age - Young adults 18-26yrs not included /outside age ranged 18-44 Years<br>White, M. C., Shoemaker, M. L., Park, S., Neff, L. J., Carlson, S. A., Brown, D. R., Kanny, D.<br>Level: 1, State: Excluded	Age - Young adults 18-26yrs not included /outside age range
1245	RefID: 1245, Study protocol: Yarning about HPV Vaccination: a qualitative study of factors influencing HPV vaccination among Aboriginal and Torres Strait Islander adolescents in Australia<br>Whop, L. J., Butler, T. L., Brotherton, J. M. L., Anderson, K., Cunningham, J., Tong, A., Garvey, G.<br>Level: 1, State: Excluded	Wrong/no population (eg parents, providers, children, policy makers, does not include 18-26yrs olds; social media posts)
1246	RefID: 1246, Knowledge, attitude and practice of HPV vaccination in undergraduate medical and nursing students<br>Wibisono, P., Widjaja, A. G., Vatvani, A. D., Velies, D. S.<br>Level: 1, State: Excluded	Study Design - Not qualitative (methods or analysis) OR Qualitative survey data not analyzed qualitatively (only numeric stats)
1247	RefID: 1247, Factors affecting HPV vaccination status among medical students in Indonesia<br>Widjaja, A. G., Wibisono, P., Velies, D. S.<br>Level: 1, State: Excluded	Wrong/no population (eg parents, providers, children, policy makers, does not include 18-26yrs olds; social media posts)
1248	RefID: 1248, Clinician and Parent Perspectives on Educational Needs for Increasing Adolescent HPV Vaccination<br>Widman, C. A., Rodriguez, E. M., Saad-Harfouche, F., Twarozek, A. M.,	Not about HPV vaccination/vax attitudes (eg HPV

	Erwin, D. O., Mahoney, M. C.<br>Level: 1, State: Excluded	infection/serology/prevalence; cervical cancer; HPV vax safety)
1249	RefID: 1249, Parents' attitudes, beliefs and uptake of the school-based human papillomavirus (HPV) vaccination program in Jakarta, Indonesia - A quantitative study<br>Wijayanti, K. E., Schütze, H., MacPhail, C.<br>Level: 1, State: Excluded	Wrong/no population (eg parents, providers, children, policy makers, does not include 18-26yrs olds; social media posts)
1251	RefID: 1251, Risk of new onset autoimmune disease in 9- to 25-year-old women exposed to human papillomavirus-16/18 AS04-adjuvanted vaccine in the United Kingdom<br>Willame, C., Rosillon, D., Zima, J., Angelo, M. G., Stuurman, A. L., Vroling, H., Boggon, R., Bunge, E. M., Pladevall-Vila, M., Baril, L.<br>Level: 1, State: Excluded	Study Design - Not qualitative (methods or analysis) OR Qualitative survey data not analyzed qualitatively (only numeric stats)
1252	RefID: 1252, Louisiana Physicians Are Increasing HPV Vaccination Rates<br>Williams, D. L., Wheeler, C. S., Lawrence, M., Hall, S. S., HAge - Young adults 18-26yrs not included /outside age rangensee, M.<br>Level: 1, State: Excluded	Age - Young adults 18-26yrs not included /outside age range
1253	RefID: 1253, 2. Availability of Youth STI and Reproductive Services in Urgent Care Centers<br>Williams, S. P., Carry, M. G.<br>Level: 1, State: Excluded	Wrong/no population (eg parents, providers, children, policy makers, does not include 18-26yrs olds; social media posts)
1254	RefID: 1254, Vaccination coverAge - Young adults 18-26yrs not included /outside age range among adults, excluding influenza vaccination - United States, 2013<br>Williams, Walter W., Lu, Peng-Jun, O'Halloran, Alissa, Bridges, Carolyn B., Kim, David K., Pilishvili, Tamara, Hales, Craig M., Markowitz, Lauri E.<br>Level: 1, State: Excluded	Wrong/no population (eg parents, providers, children, policy makers, does not include 18-26yrs olds; social media posts)
1255	RefID: 1255, Noninfluenza vaccination coverAge - Young adults 18-26yrs not included /outside age range among adults - United States, 2012<br>Williams, Walter W., Lu, Peng-Jun, O'Halloran, Alissa, Bridges, Carolyn B., Pilishvili, Tamara, Hales, Craig M., Markowitz, Lauri E.<br>Level: 1, State: Excluded	Not about HPV vaccination/vax attitudes (eg HPV infection/serology/prevalence; cervical cancer; HPV vax safety)
1258	RefID: 1258, Surveillance of Vaccination CoverAge - Young adults 18-26yrs not included /outside age range among Adult Wrong/no population (eg parents, providers, children, policy makers, does not include 18-26yrs olds; social media posts)s - United States, 2015<br>Williams, W. W., Lu, P. J., O'Halloran, A., Kim, D. K., Grohskopf, L. A., Pilishvili, T., Skoff, T. H., Nelson, N. P., Harpaz, R., Markowitz, L. E., Rodriguez-Lainz, A., Fiebelkorn, A. P.<br>Level: 1, State: Excluded	Wrong/no population (eg parents, providers, children, policy makers, does not include 18-26yrs olds; social media posts)
1259	RefID: 1259, Factors associated with human papillomavirus vaccination among young adult women in the United States<br>Williams, W. W., Lu, P. J., Saraiya, M., Yankey, D., Dorell, C., Rodriguez, J. L., Kepka, D., Markowitz, L. E.<br>Level: 1, State: Excluded	Not about HPV vaccination/vax attitudes (eg HPV infection/serology/prevalence; cervical cancer; HPV vax safety)
1261	RefID: 1261, Factors related to HPV vaccine uptake and 3-dose completion among women in a low vaccination region of the USA: an observational study<br>Wilson, A. R., Hashibe, M., Bodson, J., Gren, L. H., Taylor, B. A.,	Not about HPV vaccination/vax attitudes (eg HPV infection/serology/prevalence; cervical cancer; HPV vax safety)

	Greenwood, J., Jackson, B. R., She, R., Egger, M. J., Kepka, D.<br>Level: 1, State: Excluded	
1262	RefID: 1262, Barriers to immunization among newcomers: A systematic review<br>Wilson, L., Rubens-Augustson, T., Murphy, M., Jardine, C., Crowcroft, N., Hui, C., Wilson, K.<br>Level: 1, State: Excluded	Not about HPV vaccination/vax attitudes (eg HPV infection/serology/prevalence; cervical cancer; HPV vax safety)
1263	RefID: 1263, Newcomer knowledge, attitudes, and beliefs about human papillomavirus (HPV) vaccination<br>Wilson, L. A., Quan, A. M. L., Bota, A. B., Mithani, S. S., Paradis, M., Jardine, C., Hui, C., Pottie, K., Crowcroft, N., Wilson, K.<br>Level: 2, State: Excluded	Not about HPV vaccination/vax attitudes (eg HPV infection/serology/prevalence; cervical cancer; HPV vax safety)
1264	RefID: 1264, Ontario's school-based HPV vaccination program: School board assent and parental consent<br>Wilson, S. E., Deeks, S. L., Karas, E., Simpson, M., Henning, B., Bontovics, E., Crowcroft, N. S.<br>Level: 1, State: Excluded	Study Design - Not qualitative (methods or analysis) OR Qualitative survey data not analyzed qualitatively (only numeric stats)
1265	RefID: 1265, Anal human papillomavirus and HIV: A cross-sectional study among men who have sex with men in Moscow, Russia, 2012–2013<br>Wirtz, A. L., Zelaya, C. E., Peryshkina, A., McGowan, I., Cranston, R. D., Latkin, C., Galai, N., Mogilniy, V., Dzhigun, P., Kostetskaya, I., Beyrer, C.<br>Level: 1, State: Excluded	Study Design - Not qualitative (methods or analysis) OR Qualitative survey data not analyzed qualitatively (only numeric stats)
1266	RefID: 1266, Human papillomavirus infection among male adolescents and young adults with perinatally-acquired HIV and without HIV in Thailand<br>Wittawatmongkol, O., Gatechompol, S., Kerr, S. J., Chalermchockcharoenkit, A., Teeratakulpisarn, N., Lermankul, W., Thamkhantho, M., Phanuphak, N., Sohn, A. H., Chokephaibulkit, K.<br>Level: 1, State: Excluded	Wrong publication type (eg reviews, opinions, conference proceedings)
1267	RefID: 1267, Bivalent Vaccine Effectiveness Against Type-Specific HPV Positivity: Evidence for Cross-Protection Against Oncogenic Types Among Dutch STI Clinic Visitors<br>Woestenberg, P. J., King, A. J., van Benthem, B. H. B., Donken, R., Leussink, S., van der Klis, F. R. M., de Melker, H. E., van der Sande, M. A. B., Hoebe, Cjpa, Bogaards, J. A.<br>Level: 1, State: Excluded	Multiple Wrong/no population (eg parents, providers, children, policy makers, does not include 18-26yrs olds; social media posts)s - unable to extract data specific to young adults (18-26 yrs)
1270	RefID: 1270, Partial Protective Effect of Bivalent Human Papillomavirus 16/18 Vaccination Against Anogenital Warts in a Large Cohort of Dutch Primary Care Patients<br>Woestenberg, Petra J., Morel, Alejandra E. Guevara, Bogaards, Johannes A., Hooiveld, Mariëtte, Klooster, Tessa M. Schurink-van 't, Hoebe, Christian J. P. A., Sande, Marianne A. B. van der, Benthem, Birgit H. B. van<br>Level: 1, State: Excluded	Not about HPV vaccination/vax attitudes (eg HPV infection/serology/prevalence; cervical cancer; HPV vax safety)
1271	RefID: 1271, Knowledge and Attitudes About HPV Infection, HPV Vaccination, and Cervical Cancer Among Rural Southeast Asian Women<br>Wong, Li<br>Level: 1, State: Excluded	Study Design - Not qualitative (methods or analysis) OR Qualitative survey data not analyzed qualitatively (only numeric stats)

1273	RefID: 1273, Preventing cervical cancer through human papillomavirus vaccination: perspective from focus groups<br>Wong, L. P.<br>Level: 1, State: Excluded	Not about HPV vaccination/vax attitudes (eg HPV infection/serology/prevalence; cervical cancer; HPV vax safety)
1275	RefID: 1275, Role of men in promoting the uptake of HPV vaccinations: focus groups' finding from a developing country<br>Wong, L. P.<br>Level: 2, State: Excluded	Not about HPV vaccination/vax attitudes (eg HPV infection/serology/prevalence; cervical cancer; HPV vax safety)
1276	RefID: 1276, Ethnically diverse female university students' knowledge and attitudes toward human papillomavirus (HPV), HPV vaccination and cervical cancer<br>Wong, L. P., Sam, I. C.<br>Level: 1, State: Excluded	Not about HPV vaccination/vax attitudes (eg HPV infection/serology/prevalence; cervical cancer; HPV vax safety)
1278	RefID: 1278, What does Medicaid expansion mean for cancer screening and prevention? Results from a randomized trial on the impacts of acquiring Medicaid coverAge - Young adults 18-26yrs not included /outside age range<br>Wright, B. J., Conlin, A. K., Allen, H. L., Tsui, J., Carlson, M. J., Li, H. F.<br>Level: 1, State: Excluded	Study Design - Not qualitative (methods or analysis) OR Qualitative survey data not analyzed qualitatively (only numeric stats)
1280	RefID: 1280, The effect of perceived psychological stress on the immunogenicity of the quadrivalent human papillomavirus vaccine in males<br>Wu, R. F., Zimmerman, R. K., Lin, C. J.<br>Level: 1, State: Excluded	Wrong/no population (eg parents, providers, children, policy makers, does not include 18-26yrs olds; social media posts)
1282	RefID: 1282, Effects of messAge - Young adults 18-26yrs not included /outside age range framing and evidence type on health information behavior: the case of promoting HPV vaccination<br>Xu, X., Yang, M., Zhao, Y. C., Zhu, Q.<br>Level: 1, State: Excluded	Multiple Wrong/no population (eg parents, providers, children, policy makers, does not include 18-26yrs olds; social media posts)s - unable to extract data specific to young adults (18-26 yrs)
1283	RefID: 1283, Awareness, attitudes and acceptability of the HPV vaccine among female university students in Morocco<br>Yacouti, A., Elkhoudri, N., El Got, A., Benider, A., Hadrya, F., Baddou, R., Forster, A., Mouallif, M.<br>Level: 1, State: Excluded	Study Design - Not qualitative (methods or analysis) OR Qualitative survey data not analyzed qualitatively (only numeric stats)
1284	RefID: 1284, A nationwide birth year-by-year analysis of effectiveness of HPV vaccine in Japan<br>Yagi, A., Ueda, Y., Nakagawa, S., Masuda, T., Miyatake, T., Ikeda, S., Abe, H., Hirai, K., Sekine, M., Miyagi, E., Enomoto, T., Nakayama, T., Kimura, T.<br>Level: 1, State: Excluded	Not about HPV vaccination/vax attitudes (eg HPV infection/serology/prevalence; cervical cancer; HPV vax safety)
1286	RefID: 1286, Designing and psychometric assessment of the scale of factors influencing HPV vaccine uptake behaviors in young adults<br>Yarmohammadi, S., Ghaffari, M., Mehrabi, Y., Mousavi, S., Ramezankhani, A.<br>Level: 1, State: Excluded	Not about HPV vaccination/vax attitudes (eg HPV infection/serology/prevalence; cervical cancer; HPV vax safety)
1287	RefID: 1287, Human papillomavirus knowledge and awareness among Vietnamese mothers	Not about HPV vaccination/vax attitudes (eg HPV

	Yi, J. K., Lackey, S. C., Zahn, M. P., Castaneda, J., Hwang, J. P.<br>Level: 1, State: Excluded	infection/serology/prevalence; cervical cancer; HPV vax safety)
1289	RefID: 1289, Epidemiology of human papillomavirus on condyloma acuminatum in Shandong Province，China<br>Yuan, H., Li, R., Lv, J., Yi, G., Sun, X., Zhao, N., Zhao, F., Xu, A., Kou, Z., Wen, H.<br>Level: 1, State: Excluded	Study Design - Not qualitative (methods or analysis) OR Qualitative survey data not analyzed qualitatively (only numeric stats)
1290	RefID: 1290, Barriers to receiving human papillomavirus (HPV) vaccination among female university students in hong kong<br>Yuen-Man, J., Lam, D. C.<br>Level: 2, State: Excluded	Not about HPV vaccination/vax attitudes (eg HPV infection/serology/prevalence; cervical cancer; HPV vax safety)
1292	RefID: 1292, Knowledge, attitude and practices of cervical cancer and screening among women of reproductive Age - Young adults 18-26yrs not included /outside age range attending gynaecology clinic of tertiary care hospitals in islamic republic of pakistan: A cross sectional survey<br>Zahid Ali Khan, F., Osoba, T., Batool Mazhar, S.<br>Level: 1, State: Excluded	Study Design - Not qualitative (methods or analysis) OR Qualitative survey data not analyzed qualitatively (only numeric stats)
1293	RefID: 1293, HPV and its high-risk genotypes in Middle Eastern countries: a meta-analysis<br>Zare, E., Roozbeh, N., Akbari, P. A., Teshnizi, S. H., Ghazanfarpour, M., Abdi, F.<br>Level: 1, State: Excluded	Wrong/no population (eg parents, providers, children, policy makers, does not include 18-26yrs olds; social media posts)
1294	RefID: 1294, What impact does information about the HPV vaccination have on the decision of young women to be vaccinated?<br>Zeyer, A., Sidler, T.<br>Level: 1, State: Excluded	Not about HPV vaccination/vax attitudes (eg HPV infection/serology/prevalence; cervical cancer; HPV vax safety)
1295	RefID: 1295, How does information provided on HPV vaccination influence young women?<br>Zeyer, A., Sidler, T.<br>Level: 1, State: Excluded	studytype
1296	RefID: 1296, A retrospective study of 152 women with vaginal intraepithelial neoplasia<br>Zhang, J., Chang, X., Qi, Y., Zhang, Y., Zhang, S.<br>Level: 1, State: Excluded	Age - Young adults 18-26yrs not included /outside age range
1297	RefID: 1297, Assessment of the knowledge, attitude and practices about human papilloma virus vaccine among the nurses working in a tertiary hospital in China: A cross-sectional descriptive study<br>Zhang, J. M., Zhao, Q. M., Zhang, L. M.<br>Level: 1, State: Excluded	Not about HPV vaccination/vax attitudes (eg HPV infection/serology/prevalence; cervical cancer; HPV vax safety)
1298	RefID: 1298, Facebook for Health Promotion: Female College Students' Perspectives on Sharing HPV Vaccine Information Through Facebook<br>Zhang, N., Tsark, J., Campo, S., Teti, M.<br>Level: 2, State: Excluded	Not about HPV vaccination/vax attitudes (eg HPV infection/serology/prevalence; cervical cancer; HPV vax safety)
1299	RefID: 1299, The failure of news coverAge - Young adults 18-26yrs not included /outside age range supportive of human papillomavirus vaccination: The investigation of the effects of online comments on female college students' vaccination intention<br>Zhang, W., Wang, Q.<br>Level: 1, State: Excluded	Wrong publication type (eg reviews, opinions, conference proceedings)

1300	RefID: 1300, A multi-center survey of Age - Young adults 18-26yrs not included /outside age range of sexual debut and sexual behavior in Chinese women: suggestions for optimal Age - Young adults 18-26yrs not included /outside age range of human papillomavirus vaccination in China<br>Zhao, F. H., Tiggelaar, S. M., Hu, S. Y., Xu, L. N., Hong, Y., Niyazi, M., Gao, X. H., Ju, L. R., Zhang, L. Q., Feng, X. X., Duan, X. Z., Song, X. L., Wang, J., Yang, Y., Li, C. Q., Liu, J. H., Liu, J. H., Lu, Y. B., Li, L., Zhou, Q., Liu, J. F., Zhao, N., Schmidt, J. E., Qiao, Y. L.<br>Level: 1, State: Excluded	Not about HPV vaccination/vax attitudes (eg HPV infection/serology/prevalence; cervical cancer; HPV vax safety)
1303	RefID: 1303, Factors Related to Chinese Parents' HPV Vaccination Intention for Children<br>Zhu, L., Zhai, S., Siu, P. T., Xia, H. Y., Lai, S., Zambrano, C. N., Ma, G. X.<br>Level: 1, State: Excluded	Study Design - Not qualitative (methods or analysis) OR Qualitative survey data not analyzed qualitatively (only numeric stats)
1304	RefID: 1304, Physicians' sexual health discussions with adolescent males and attitudes about HPV vaccination<br>Zimet, G., Weiss, T., Rosenthal, S., Brenneman, S., Klein, J.<br>Level: 1, State: Excluded	Not about HPV vaccination/vax attitudes (eg HPV infection/serology/prevalence; cervical cancer; HPV vax safety)
1305	RefID: 1305, Site-specific human papillomavirus infection in adolescent men who have sex with men (HYPER): an observational cohort study<br>Zou, H., Tabrizi, S. N., Grulich, A. E., Hocking, J. S., Bradshaw, C. S., Cornall, A. M., Morrow, A., PrestAge - Young adults 18-26yrs not included /outside age range, G., Law, M. G., Garland, S. M., Chen, M. Y., Fairley, C. K.<br>Level: 1, State: Excluded	Study Design - Not qualitative (methods or analysis) OR Qualitative survey data not analyzed qualitatively (only numeric stats)
1306	RefID: 1306, HPV vaccines: what remains to be done? Interview by Lauren Constable<br>Zur Hausen, H.<br>Level: 1, State: Excluded	Study Design - Not qualitative (methods or analysis) OR Qualitative survey data not analyzed qualitatively (only numeric stats)
1307	RefID: 1307, Special Issue: Focus groups<br><br>Level: 1, State: Excluded	Wrong/no population (eg parents, providers, children, policy makers, does not include 18-26yrs olds; social media posts)
1308	RefID: 1308, Special Issue: Advancing the science of qualitative research to promote health equity<br><br>Level: 1, State: Excluded	Study Design - Not qualitative (methods or analysis) OR Qualitative survey data not analyzed qualitatively (only numeric stats)
1309	RefID: 1309, Special Issue: Latin American health sociology<br><br>Level: 1, State: Excluded	Not about HPV vaccination/vax attitudes (eg HPV infection/serology/prevalence; cervical cancer; HPV vax safety)
1310	RefID: 1310, Abstracts of the RCOG World Congress 2022<br><br>Level: 1, State: Excluded	Wrong publication type (eg reviews, opinions, conference proceedings)
1311	RefID: 1311, Corrigendum to Human papillomavirus vaccine communication materials for young people in English-speaking	Wrong/no population (eg parents, providers, children, policy makers,

	countries: A content analysis (Health Education Journal, (2022), 10.1177/00178969221092135.)<br><br>Level: 2, State: Excluded	does not include 18-26yrs olds; social media posts)
1312	RefID: 1312, Corrigendum to Human papillomavirus vaccine communication materials for young people in English-speaking countries: A content analysis...Fisher H, Chantler T, Mounier-Jack S, et al. Human papillomavirus vaccine communication materials for young people in English-speaking countries: A content analysis. Health Education Journal. 2022;81(5):513-528<br><br>Level: 1, State: Excluded	Wrong publication type (eg reviews, opinions, conference proceedings)
1313	RefID: 1313, How can we improve the acceptability of vaccination against Human Papillomavirus (HPV) in France? An original qualitative study with focus groups comprising parents and school staff, interviewed separately<br>Ailloud, J., Branchereau, M., Fall, E., Juneau, C., Partouche, H., Bonnay, S., Oudin-Doglioni, D., Michel, M., Gagneux-Brunon, A., Bruel, S., Thilly, N., Gauchet, A.<br>Level: 1, State: Excluded	Wrong publication type (eg reviews, opinions, conference proceedings)
1314	RefID: 1314, Knowledge, attitude, and utilization of human papillomavirus vaccination among female undergraduates in Ekiti State, Nigeria<br>Akpor, O. A., Adeyiolu, A. T., Alade, M. I., Adebiyi, S., Adeola, R. S., Adeyanju, B. T.<br>Level: 1, State: Excluded	Wrong publication type (eg reviews, opinions, conference proceedings)
1315	RefID: 1315, StAge - Young adults 18-26yrs not included /outside age range of adoption of the human papillomavirus vaccine among college women<br>Allen, J. D., Mohllajee, A. P., Shelton, R. C., Othus, M. K. D., Fontenot, H. B., Hanna, R.<br>Level: 1, State: Excluded	Not about HPV vaccination/vax attitudes (eg HPV infection/serology/prevalence; cervical cancer; HPV vax safety)
1317	RefID: 1317, School-based interventions on human papillomavirus in Africa: a systematic scoping review<br>Amzat, J., Kanmodi, K. K., Aminu, K., Egbedina, E. A.<br>Level: 1, State: Excluded	Wrong publication type (eg reviews, opinions, conference proceedings)
1318	RefID: 1318, Genital warts, persistent infection, and disclosure after genital human papillomavirus infection in young men<br>Arima, Yuzo<br>Level: 1, State: Excluded	Wrong/no population (eg parents, providers, children, policy makers, does not include 18-26yrs olds; social media posts)
1319	RefID: 1319, Correlates of Human papillomavirus (HPV) vaccine acceptance in Appalachian Tennessee<br>Ariyo, Oluwatosin<br>Level: 1, State: Excluded	Study Design - Not qualitative (methods or analysis) OR Qualitative survey data not analyzed qualitatively (only numeric stats)
1320	RefID: 1320, Impact and acceptability of self-consent procedures for the school-based human papillomavirus vaccine: a mixed-methods study protocol<br>Audrey, S., Batista Ferrer, H., Ferrie, J., Evans, K., Bell, M., Yates, J.,	Study Design - Not qualitative (methods or analysis) OR Qualitative survey data not analyzed qualitatively (only numeric stats)

	Roderick, M., MacLeod, J., Hickman, M.<br>Level: 2, State: Excluded	
1321	RefID: 1321, Implementing new consent procedures for schools-based human papillomavirus vaccination: a qualitative study<br>Audrey, Suzanne, Evans, Karen, Farr, Michelle, Ferrie, Joanne, Yates, Julie, Roderick, Marion, Fisher, Harriet<br>Level: 1, State: Excluded	Wrong publication type (eg reviews, opinions, conference proceedings)
1322	RefID: 1322, How acceptable is adolescent self-consent for the HPV vaccination: Findings from a qualitative study in south-west England<br>Audrey, S., Farr, M., Roderick, M., Evans, K., Fisher, H.<br>Level: 1, State: Excluded	Study Design - Not qualitative (methods or analysis) OR Qualitative survey data not analyzed qualitatively (only numeric stats)
1326	RefID: 1326, Pharmacovigilance Reports Received from Children and Young People, and Development of Information to Aid Future Reporting from this Age - Young adults 18-26yrs not included /outside age range Group<br>Bhoombla, N., Preston, J., Ainsworth, J., Bird, H., Jadeja, M., King, C., Hawcutt, D. B.<br>Level: 1, State: Excluded	Study Design - Not qualitative (methods or analysis) OR Qualitative survey data not analyzed qualitatively (only numeric stats)
1327	RefID: 1327, Knowledge of young Polish women of human papillomavirus (HPV) infection and cervical cancer prevention<br>Biała, M., Inglot, M., Knysz, B.<br>Level: 1, State: Excluded	studytype
1328	RefID: 1328, Epidemiology and cost of cervical cancer care and prevention in Apulia (Italy), 2007/2016<br>Bianchi, F. P., Gallone, M. S., Fortunato, F., Boccalini, S., Martinelli, D., Prato, R., Tafuri, S.<br>Level: 1, State: Excluded	Not about HPV vaccination/vax attitudes (eg HPV infection/serology/prevalence; cervical cancer; HPV vax safety)
1329	RefID: 1329, From invitation to follow-up: women's experiences of participation or non-participation in cervical cancer screening<br>Blomberg, K.<br>Level: 1, State: Excluded	Wrong/no population (eg parents, providers, children, policy makers, does not include 18-26yrs olds; social media posts)
1331	RefID: 1331, Co-development of a school-based and primary care-based multicomponent intervention to improve HPV vaccine coverAge - Young adults 18-26yrs not included /outside age range amongst French adolescents (the PrevHPV Study)<br>Bocquier, Aurélie, Bruel, Sébastien, Michel, Morgane, Le Duc-Banaszuk, Anne-Sophie, Bonnay, Stéphanie, Branchereau, Marion, Chevreul, Karine, Chyderiotis, Sandra, Gauchet, Aurélie, Giraudeau, Bruno, Hagiu, Dragos-Paul, Mueller, Judith E., Gagneux-Brunon, Amandine, Thilly, Nathalie<br>Level: 2, State: Excluded	Age - Young adults 18-26yrs not included /outside age range
1334	RefID: 1334, Knowledge and attitudes about human papillomavirus and vaccination<br>Carneiro da Silva, Priscila Mendonça, Barbosa Silva, Izabele Maria, da Conceição Souza, Iris Nayara, Interaminense, Pereira Linhares, Francisca Márcia, Serrano, Solange Queiroga, Pontes, Cleide Maria<br>Level: 2, State: Excluded	Study Design - Not qualitative (methods or analysis) OR Qualitative survey data not analyzed qualitatively (only numeric stats)

1335	RefID: 1335, Prevalence and determinants of human Papillomavirus infection in young women from guanacasteand puntarenas, Costa Rica, 2004-2005<br>Carvajal, L. J., Herrero, R., Angulo, M. M., Schussler, J., Porras, C., Ocampo, R., Cortés, B., Loría, V., Castrillo, H., Romero, B., Barrientos, G., Coronado, K., Ávila, C., Hildesheim, A., Rodríguez, A. C., Jiménez, S. E., Kreimer, A. R., Sierra, M. S.<br>Level: 1, State: Excluded	Study Design - Not qualitative (methods or analysis) OR Qualitative survey data not analyzed qualitatively (only numeric stats)
1337	RefID: 1337, High-risk human papillomavirus genotype distribution among women living with and at risk for HIV in Africa<br>ChachAge - Young adults 18-26yrs not included /outside age range, M., Parikh, A. P., Mahenge, A., Bahemana, E., Mnkai, J., Mbuya, W., McHaro, R., Maganga, L., Mwamwaja, J., Gervas, R., Kibuuka, H., Maswai, J., Singoei, V., Iroezindu, M., Fasina, A., Esber, A., Dear, N., Imbach, M., Crowell, T. A., Hern, J., Song, X., Hoelscher, M., Polyak, C. S., Ake, J. A., Geldmacher, C.<br>Level: 1, State: Excluded	Not about HPV vaccination/vax attitudes (eg HPV infection/serology/prevalence; cervical cancer; HPV vax safety)
1338	RefID: 1338, A randomized controlled trial of HPV vaccine hesitancy text intervention based on health behavior theory (Students and parents)<br>ChiCtr<br>Level: 1, State: Excluded	Not about HPV vaccination/vax attitudes (eg HPV infection/serology/prevalence; cervical cancer; HPV vax safety)
1340	RefID: 1340, Optimising HPV vaccination communication to adolescents: A discrete choice experiment<br>Chyderiotis, S., Sicsic, J., Raude, J., Bonmarin, I., Jeanleboeuf, F., Le Duc Banaszuk, A. S., Gauchet, A., Bruel, S., Michel, M., Giraudeau, B., Thilly, N., Mueller, J. E.<br>Level: 1, State: Excluded	Wrong/no population (eg parents, providers, children, policy makers, does not include 18-26yrs olds; social media posts)
1342	RefID: 1342, Impact of preparing nursing students to deliver a parent-based sexual health intervention on attitudes and intentions for sexual health education and parent communication counseling: a mixed methods study<br>Coleman, D. C., Frederick, A., Cron, S., Markham, C., Guilamo-Ramos, V., Santa Maria, D.<br>Level: 1, State: Excluded	Study Design - Not qualitative (methods or analysis) OR Qualitative survey data not analyzed qualitatively (only numeric stats)
1343	RefID: 1343, HPV vaccination mandates - lawmaking amid political and scientific controversy<br>Colgrove, J., Abiola, S., Mello, M. M.<br>Level: 1, State: Excluded	Study Design - Not qualitative (methods or analysis) OR Qualitative survey data not analyzed qualitatively (only numeric stats)
1344	RefID: 1344, Development of a human papillomavirus vaccination intervention for Australian adolescents<br>Cooper, S. C., Davies, C., McBride, K., Blades, J., Stoney, T., Marshall, H., Skinner, S. R.<br>Level: 1, State: Excluded	Study Design - Not qualitative (methods or analysis) OR Qualitative survey data not analyzed qualitatively (only numeric stats)
1346	RefID: 1346, NURSING STUDENTS AND KNOWLEDGE ABOUT HUMAN PAPILLOMAVIRUS AND ITS IMMUNIZING: A CROSS-SECTIONAL STUDY<br>da Silva Júnior, J. A., dos Santos, S. M. P., Bezerra, L. L. O., da Silva Freitas, J. L. G., Neta, M. L. B.<br>Level: 1, State: Excluded	Age - Young adults 18-26yrs not included /outside age range

1348	RefID: 1348, Knowledge and attitudes of parents of children/adolescents about human papillomavirus: cross-sectional study<br>de Matos, L. F. S. F., Campelo, G. S., da Silva, A. S., de Paula Andrade, R. L., dos Santos, E. M., Mendez, R. D. R., dos Santos, M. A., Wysocki, A. D.<br>Level: 1, State: Excluded	Wrong/no population (eg parents, providers, children, policy makers, does not include 18-26yrs olds; social media posts)
1349	RefID: 1349, An Application of the Integrative Model to Women's Intention to Be Vaccinated Against HPV: Implications for MessAge - Young adults 18-26yrs not included /outside age range Design<br>Dillard, James Price<br>Level: 1, State: Excluded	Wrong/no population (eg parents, providers, children, policy makers, does not include 18-26yrs olds; social media posts)
1350	RefID: 1350, Real-world effectiveness of HPV vaccination against cervical neoplasia among birth cohorts ineligible for routine vaccination<br>Dong, L., Nygård, M., Støer, N. C., Klungsøyr, O., Hansen, B. T.<br>Level: 1, State: Excluded	Wrong/no population (eg parents, providers, children, policy makers, does not include 18-26yrs olds; social media posts)
1351	RefID: 1351, The Effect of University Students' Levels of Knowledge about HPV Infection and the HPV Vaccine on Their Health Beliefs: Health Sciences Students<br>Ergün, S.<br>Level: 1, State: Excluded	Study Design - Not qualitative (methods or analysis) OR Qualitative survey data not analyzed qualitatively (only numeric stats)
1353	RefID: 1353, YOUNG PEOPLE IN WEB RADIO: SOCIAL REPRESENTATIONS ABOUT HUMAN PAPILLOMAVIRUS<br>Feitosa de Araújo, Aretha, Ribeiro de Castro Júnior, André, de Freitas, Maria Célia, Duarte Pereira, Maria Lúcia, Paiva Rodrigues, Dafne, Martins Torres, Raimundo Augusto, Ferreira da Silva, Maria Rocineide, de Sá Barreto Marinho, Mirna Neyara Alexandre<br>Level: 1, State: Excluded	Study Design - Not qualitative (methods or analysis) OR Qualitative survey data not analyzed qualitatively (only numeric stats)
1355	RefID: 1355, Development of a multicomponent intervention to increase parental vaccine confidence and young people's access to the universal HPV vaccination programme in England: protocol for a co-design study<br>Fisher, H., Chantler, T., Denford, S., Finn, A., Hickman, M., Mounier-Jack, S., Roderick, M., Tucker, L., Yates, J., Audrey, S.<br>Level: 1, State: Excluded	Study Design - Not qualitative (methods or analysis) OR Qualitative survey data not analyzed qualitatively (only numeric stats)
1356	RefID: 1356, Adolescent self-consent for vaccinations: Protocol for a mixed methods systematic review<br>Fisher, H., Hickman, M., Macleod, J., Audrey, S.<br>Level: 1, State: Excluded	Study Design - Not qualitative (methods or analysis) OR Qualitative survey data not analyzed qualitatively (only numeric stats)
1357	RefID: 1357, Development and piloting of a randomized controlled trial of a narrative communication intervention to increase human papillomavirus vaccination intentions and uptake in a college Wrong/no population (eg parents, providers, children, policy makers, does not include 18-26yrs olds; social media posts)<br>Fleszar-Pavlović, Sara E.<br>Level: 1, State: Excluded	Study Design - Not qualitative (methods or analysis) OR Qualitative survey data not analyzed qualitatively (only numeric stats)
1358	RefID: 1358, Applying the COM-B behaviour model to understand factors which impact school immunisation nurses' attitudes towards designing and delivering a HPV educational intervention in post-primary schools for 15-17 year old students in Northern Ireland, UK	Study Design - Not qualitative (methods or analysis) OR Qualitative survey data not

	Flood, T., McLaughlin, M., Hughes, C. M., Wilson, I. M.<br>Level: 1, State: Excluded	analyzed qualitatively (only numeric stats)
1360	RefID: 1360, Parent and healthcare provider beliefs about a chlamydia adolescent vaccine<br>Footman, Alison P.<br>Level: 1, State: Excluded	Wrong publication type (eg reviews, opinions, conference proceedings)
1361	RefID: 1361, Exploring knowledge and beliefs of human papillomavirus (HPV) infection and HPV vaccination among US Chinese international students<br>Gao, Haijuan<br>Level: 2, State: Excluded	Wrong publication type (eg reviews, opinions, conference proceedings)
1362	RefID: 1362, Examining the Barriers and Opportunities for Human Papillomavirus Vaccine Delivery in Cancer Care Settings: A Mixed-Methods Study<br>Garcia, M. A., Schlecht, N. F., Rokitka, D. A., Attwood, K. M., Rodriguez, E. M.<br>Level: 1, State: Excluded	Not about HPV vaccination/vax attitudes (eg HPV infection/serology/prevalence; cervical cancer; HPV vax safety)
1364	RefID: 1364, Knowledge and willingness of parents towards child girl HPV vaccination in Debre Tabor town, Ethiopia: a community-based cross-sectional study<br>Gedefaye Nibret, Mihretie, Tewachew Muche, Liyeh, Alemu Degu, Ayele, Habtamu Gebrehana, Belay, Tigist Seid, Yimer, Age - Young adults 18-26yrs not included /outside age rangernesh Dereje, Miskr<br>Level: 1, State: Excluded	Age - Young adults 18-26yrs not included /outside age range
1365	RefID: 1365, Development and Appraisal of a Web-Based Decision Aid for HPV Vaccination for Young Adults and Parents of Children in Israel-A Quasi-Experimental Study<br>Gendler, Y.<br>Level: 2, State: Excluded	Not about HPV vaccination/vax attitudes (eg HPV infection/serology/prevalence; cervical cancer; HPV vax safety)
1367	RefID: 1367, Playful strategies to increase knowledge about the human papillomavirus in a group of adolescents<br>Gómez-Marín, S. M., Agudelo-Ramírez, A., Pradilla-Serrano, Á S., García-Hincapié, J.<br>Level: 2, State: Excluded	studytype
1368	RefID: 1368, Informed consent for HPV vaccination: a relational approach<br>Gottvall, M., Tydén, T., Larsson, M., Stenhammar, C., Höglund, A. T.<br>Level: 1, State: Excluded	Wrong/no population (eg parents, providers, children, policy makers, does not include 18-26yrs olds; social media posts)
1369	RefID: 1369, Awareness and knowledge of cervical cancer in medical and paramedical staff-an observational study<br>Gupta, P., Kaveeshwar, M., Patil, A.<br>Level: 1, State: Excluded	Study Design - Not qualitative (methods or analysis) OR Qualitative survey data not analyzed qualitatively (only numeric stats)
1371	RefID: 1371, Newsprint media representations of the introduction of the HPV vaccination programme for cervical cancer prevention in the UK (2005 -- 2008)<br>Hilton, S., Hunt, K., Langan, M., Bedford, H., Petticrew, M.<br>Level: 1, State: Excluded	Study Design - Not qualitative (methods or analysis) OR Qualitative survey data not analyzed qualitatively (only numeric stats)

1373	RefID: 1373, Communication Pathways: HPV Information and MessAge - Young adults 18-26yrs not included /outside age range Barriers Reported among American Indian College Students<br>Hodge, Felicia Schanche, Line-Itty, Tracy, Ellenwood, Cheryl<br>Level: 1, State: Excluded	Wrong/no population (eg parents, providers, children, policy makers, does not include 18-26yrs olds; social media posts)
1374	RefID: 1374, Cervical Cancer and Human Papillomavirus Awareness among Women in Antigua and Barbuda<br>Honnavar, P., Mansoor, E., Tulloch, C., Udayan, U., Cosmello, I., Patel, P., Bersma, A.<br>Level: 1, State: Excluded	Not about HPV vaccination/vax attitudes (eg HPV infection/serology/prevalence; cervical cancer; HPV vax safety)
1375	RefID: 1375, Culture-centric narratives as health messAge - Young adults 18-26yrs not included /outside age range design strategy: Developing an HPV vaccine intervention for college-Age - Young adults 18-26yrs not included /outside age ranged women<br>Hopfer, Suellen<br>Level: 1, State: Excluded	Study Design - Not qualitative (methods or analysis) OR Qualitative survey data not analyzed qualitatively (only numeric stats)
1379	RefID: 1379, Disparities in utilization of preventive health services among Asian young adults in the United States<br>Kang, Y., Kang, S., Gibson, D., Rodriguez, A. M., Prochaska, J., Kaul, S.<br>Level: 1, State: Excluded	Wrong publication type (eg reviews, opinions, conference proceedings)
1380	RefID: 1380, Acceptability of HPV vaccine among young adolescent girls in Uganda: Young people's perspectives count<br>Katahoire, Anne R., Wani, John Arube, Murokora, Daniel, Mugisha, Emmanuel, LaMontague, D. Scott<br>Level: 1, State: Excluded	Not about HPV vaccination/vax attitudes (eg HPV infection/serology/prevalence; cervical cancer; HPV vax safety)
1381	RefID: 1381, 'Just that little bit of doubt': Scottish parents', teenAge - Young adults 18-26yrs not included /outside age range girls' and health professionals' views of the MMR, H1N1 and HPV vaccines<br>Kennedy, C., Gray Brunton, C., Hogg, R.<br>Level: 1, State: Excluded	Study Design - Not qualitative (methods or analysis) OR Qualitative survey data not analyzed qualitatively (only numeric stats)
1384	RefID: 1384, Spontaneous reporting of suspected adverse drug reactions by children and young people: Past, present and future<br>King, C., Bhoombla, N., Clarke, J., Morgan, V., Preston, J., Ainsworth, J., Hanson, A., Hawcutt, D.<br>Level: 1, State: Excluded	Wrong publication type (eg reviews, opinions, conference proceedings)
1385	RefID: 1385, Acceptability, access, and uptake of human papillomavirus vaccination in mental health Wrong/no population (eg parents, providers, children, policy makers, does not include 18-26yrs olds; social media posts)s: a scoping review<br>King, K. D., Fernandez-Sanchez, H., MacDonald, S. E.<br>Level: 1, State: Excluded	Not about HPV vaccination/vax attitudes (eg HPV infection/serology/prevalence; cervical cancer; HPV vax safety)
1387	RefID: 1387, The impact of health education interventions on HPV vaccination uptake, awareness, and acceptance among people under 30 years old in India: a literature review with systematic search<br>Krokidi, E., Rao, A. P., Ambrosino, E., Thomas, P. P. M.<br>Level: 1, State: Excluded	Age - Young adults 18-26yrs not included /outside age range
1388	RefID: 1388, Qualitative study of the feasibility of HPV vaccine delivery to young adolescent girls in Vietnam: evidence from a government-implemented demonstration program	Wrong/no population (eg parents, providers, children, policy makers,

	LaMontagne, D. S., Nguyen Quy, Nghi, Le Thi, Nga, Janmohamed, A., Dang Thi, Huyen, Nguyen Tran, Hien, Tsu, V. D.<br>Level: 1, State: Excluded	does not include 18-26yrs olds; social media posts)
1390	RefID: 1390, Implementation of a provider-focused intervention for maximizing human papillomavirus (HPV) vaccine uptake in young cancer survivors receiving follow-up care in pediatric oncology practices: protocol for a cluster-randomized trial of the HPV PROTECT intervention<br>Landier, W., Bhatia, S., Richman, J. S., Campos Gonzalez, P. D., Cherven, B., Chollette, V., Aye, J., Castellino, S. M., Gramatges, M. M., Lindemulder, S., Russell, T. B., Turcotte, L. M., Colditz, G. A., Gilkey, M. B., Klosky, J. L.<br>Level: 1, State: Excluded	Study Design - Not qualitative (methods or analysis) OR Qualitative survey data not analyzed qualitatively (only numeric stats)
1391	RefID: 1391, Knowledge about cervical cancer, health beliefs and human papillomavirus vaccination rate in female university students<br>Lee, Eun-Joo, Pakr, Jeong-Sook<br>Level: 2, State: Excluded	Wrong publication type (eg reviews, opinions, conference proceedings)
1392	RefID: 1392, Barriers to Cervical Cancer Screening and Prevention in Young Korean Immigrant Women: Implications for Intervention Development<br>Lee, Hee Yun, Lee, Mi Hwa<br>Level: 2, State: Excluded	Wrong publication type (eg reviews, opinions, conference proceedings)
1396	RefID: 1396, Romanian adolescents' knowledge and attitudes towards human papillomavirus infection and prophylactic vaccination<br>Maier, C., Maier, T., Neagu, C. E., Vlădăreanu, R.<br>Level: 1, State: Excluded	Not about HPV vaccination/vax attitudes (eg HPV infection/serology/prevalence; cervical cancer; HPV vax safety)
1397	RefID: 1397, Papillomavirus vaccination and Guillain-Barre Syndrome among girls: A cohort study in Spain<br>Martín-Merino, E., Castillo-Cano, B., Martín-Perez, M., Llorente-García, A., Montero-Corominas, D.<br>Level: 1, State: Excluded	Wrong/no population (eg parents, providers, children, policy makers, does not include 18-26yrs olds; social media posts)
1398	RefID: 1398, Association between Human Papilloma Virus (HPV) vaccination and risk of Multiple Sclerosis: A systematic review<br>Meggiolaro, A., Migliara, G., La Torre, G.<br>Level: 1, State: Excluded	Study Design - Not qualitative (methods or analysis) OR Qualitative survey data not analyzed qualitatively (only numeric stats)
1399	RefID: 1399, Pharmaceutical Companies' Role in State Vaccination Policymaking: The Case of Human Papillomavirus Vaccination<br>Mello, Michelle M., Abiola, Sara, Colgrove, James<br>Level: 1, State: Excluded	Multiple Wrong/no population (eg parents, providers, children, policy makers, does not include 18-26yrs olds; social media posts)s - unable to extract data specific to young adults (18-26 yrs)
1400	RefID: 1400, Human papillomavirus vaccine hesitancy highly evident among caregivers of girls attending South African private schools<br>Milondzo, T., Meyer, J. C., Dochez, C., Burnett, R. J.<br>Level: 1, State: Excluded	Age - Young adults 18-26yrs not included /outside age range
1404	RefID: 1404, Concomitant adolescent vaccination: The influence of seasonal variation, school requirements, and patient-provider communication	Study Design - Not qualitative (methods or analysis) OR Qualitative survey data not

	Moss, Jennifer L.<br>Level: 1, State: Excluded	analyzed qualitatively (only numeric stats)
1405	RefID: 1405, HPV vaccine uptake in survivors of childhood and adolescent and young adult (AYA) cancer<br>Nair, M., Dagli, C. S., Tobo, B. B., Tam, S. H., Al-Antary, N., Osazuwa-Peters, N., Boakye, E. A.<br>Level: 1, State: Excluded	Wrong publication type (eg reviews, opinions, conference proceedings)
1406	RefID: 1406, Perceived facilitators and barriers to the uptake of the human papillomavirus (HPV) vaccine among adolescents of Arabic-speaking mothers in NSW, Australia: A qualitative study<br>Netfa, F., King, C., Davies, C., Rashid, H., Tashani, M., Booy, R., Rachel Skinner, S.<br>Level: 1, State: Excluded	Wrong/no population (eg parents, providers, children, policy makers, does not include 18-26yrs olds; social media posts)
1407	RefID: 1407, The patient perspective on vaccine uptake in adults with psoriasis and eczema<br>Noe, M. H., Archila, M., Barbieri, J. S., Goldman, N., Lopez, C. G., Mostaghimi, A., Scherer, A. M., Tan, A. J., Perez-Chada, L. M., Asgari, M. M., Gelfand, J. M.<br>Level: 1, State: Excluded	Wrong/no population (eg parents, providers, children, policy makers, does not include 18-26yrs olds; social media posts)
1409	RefID: 1409, Rapidly increasing trends in oropharyngeal carcinoma assessed by worldwide epidemiologic analysis<br>Ofo, E., Yokoyama, J.<br>Level: 1, State: Excluded	Age - Young adults 18-26yrs not included /outside age range
1410	RefID: 1410, Factors associated with knowledge, attitude, and practice of schoolgirls about vaccination against Human Papillomavirus<br>Oliveira Catunda Ferreira, Hellen Lívia, Siqueira, Cícero Mendes, da Costa, Nicolau, Pereira, Edienovi da Costa, Fiúza, Adine de Andrade, Ribeiro, Samila Gomes, Bezerra Pinheiro, Ana Karina<br>Level: 1, State: Excluded	Age - Young adults 18-26yrs not included /outside age range
1411	RefID: 1411, Walking the (argumentative) talk using citizen science: Involving young people in a critical policy analysis of vaccination policy in Austria<br>Paul, K. T., Palfinger, T.<br>Level: 1, State: Excluded	Wrong/no population (eg parents, providers, children, policy makers, does not include 18-26yrs olds; social media posts)
1412	RefID: 1412, HPV infection among HIV-positive women in some countries of Eastern Europe and Central Asia<br>Popova, A., Shipulina, O., Deulina, M., Almamedova, E., Rzayeva, A., Kadyrova, A., Grigoryan, S., Asmaryan, A., Pepanyan, A., Davidyan, A., Ermolenko, L., Nevmerzhitskaya, T., Tavtyn, I., Kadyrbekov, U., Abylgazieva, N., Zhaanbaeva, Z., Spirin, A., Agafonova, O., Chesnokov, M., Kalenik, L., Karimov, S., Rahimova, R., Rustamova, M., Nurlyaminova, Z., Dmitryukova, M., Pokrovsky, V.<br>Level: 1, State: Excluded	Not about HPV vaccination/vax attitudes (eg HPV infection/serology/prevalence; cervical cancer; HPV vax safety)
1413	RefID: 1413, Development and validation of a theory of planned behavior-based instrument to predict Human Papillomavirus vaccination intentions of college males at a southeastern university<br>Priest, Hannah Marie<br>Level: 1, State: Excluded	Not about HPV vaccination/vax attitudes (eg HPV infection/serology/prevalence; cervical cancer; HPV vax safety)

1414	RefID: 1414, Attitude and family support influenced human papilloma virus vaccination in female midwifery students<br>Putri, I. A., Handayani, S., Tjokroprawiro, B. A.<br>Level: 1, State: Excluded	Study Design - Not qualitative (methods or analysis) OR Qualitative survey data not analyzed qualitatively (only numeric stats)
1417	RefID: 1417, Integration of the program health in the school for means of actions of promotion and prevention during the supervised curse stAge - Young adults 18-26yrs not included /outside age range of nursing: experience report<br>Rocha Júnior, E. F., Peixoto, H. M. C., Lopes, V. C., Silva, V. F., Alves, C. R., Silva, P. L. N. da<br>Level: 1, State: Excluded	Not about HPV vaccination/vax attitudes (eg HPV infection/serology/prevalence; cervical cancer; HPV vax safety)
1418	RefID: 1418, Balancing between being proactive and neutral: School nurses' experiences of offering human papilloma virus vaccination to girls<br>Runngren, Eva, Eriksson, Mats, Blomberg, Karin<br>Level: 1, State: Excluded	Age - Young adults 18-26yrs not included /outside age range
1419	RefID: 1419, Awareness regarding human papilloma virus and its vaccine among final year MBBS students<br>Sanghavi, M. M.<br>Level: 2, State: Excluded	Study Design - Not qualitative (methods or analysis) OR Qualitative survey data not analyzed qualitatively (only numeric stats)
1420	RefID: 1420, The Relationship Between Receiving Care Within a Medical Home and HPV Vaccine Receipt for Adolescent Girls: Results of the 2007 National Survey of Children's Health<br>Sara Test, F., Caskey, Rachel, Rankin, Kristin<br>Level: 1, State: Excluded	Study Design - Not qualitative (methods or analysis) OR Qualitative survey data not analyzed qualitatively (only numeric stats)
1421	RefID: 1421, Adaptation and Formative Evaluation of Online Decision Support to Implement Evidence-Based Strategies to Increase HPV Vaccination Rates in Pediatric Clinics<br>Shegog, R., Savas, L. S., Frost, E. L., Thormaehlen, L. C., Teague, T., Steffy, J., Healy, C. M., Shay, L. A., Preston, S., Vernon, S. W.<br>Level: 1, State: Excluded	Wrong/no population (eg parents, providers, children, policy makers, does not include 18-26yrs olds; social media posts)
1422	RefID: 1422, Public awareness of the HPV vaccine: A national Wrong/no population (eg parents, providers, children, policy makers, does not include 18-26yrs olds; social media posts)-based study<br>Shrader, A., Niccolai, L., Mayne, S. T., DiMaio, D., Chagpar, A. B.<br>Level: 1, State: Excluded	Wrong/no population (eg parents, providers, children, policy makers, does not include 18-26yrs olds; social media posts)
1424	RefID: 1424, Perceptions of and barriers to vaccinating daughters against human papillomavirus (HPV) among mothers in Hong Kong<br>Siu, J. Y.<br>Level: 1, State: Excluded	Study Design - Not qualitative (methods or analysis) OR Qualitative survey data not analyzed qualitatively (only numeric stats)
1431	RefID: 1431, Exploring HPV vaccination policy and payer strategies for opportunities to improve uptake in safety-net settings<br>Sloan, K., Shin, M., Palinkas, L. A., Hudson, S. V., Crabtree, B. F., Cantor, J. C., Tsui, J.<br>Level: 1, State: Excluded	Study Design - Not qualitative (methods or analysis) OR Qualitative survey data not analyzed qualitatively (only numeric stats)

1432	RefID: 1432, What do South African adolescents want in a sexual health service? Evidence from the South African Studies on HIV in Adolescents (SASHA) project<br>Smith, P., Marcus, R., Bennie, T., Nkala, B., Nchabeleng, M., Latka, M. H., Gray, G., Wallace, M., Bekker, L. G.<br>Level: 1, State: Excluded	Study Design - Not qualitative (methods or analysis) OR Qualitative survey data not analyzed qualitatively (only numeric stats)
1435	RefID: 1435, Analysis of the Conversations on Twitter regarding HPV Vaccine<br>Suzuki, Daisuke, Nishimura, Shoji, Jin, Qun, Ogihara, Atsushi<br>Level: 1, State: Excluded	Study Design - Not qualitative (methods or analysis) OR Qualitative survey data not analyzed qualitatively (only numeric stats)
1436	RefID: 1436, Details of Cancer Education Programs for Adolescents and Young Adults and Their Effectiveness: A Scoping Review<br>Suzuki, K., Yamanaka, M., Minamiguchi, Y., Hayashi, N., Yamauchi, E., Fukawa, A., Tsuda, Y., Fujisaka, Y., Doi, T., Shiino, I., Tomari, Y.<br>Level: 1, State: Excluded	Wrong/no population (eg parents, providers, children, policy makers, does not include 18-26yrs olds; social media posts)
1437	RefID: 1437, Awareness and attitude about carcinoma cervix and human papillomavirus vaccine: a cross-sectional study among undergraduate female medical students<br>Tripathy, R. M., Nivedita, Karmee, Kabita, Behera, Sahoo, S. K.<br>Level: 2, State: Excluded	Wrong/no population (eg parents, providers, children, policy makers, does not include 18-26yrs olds; social media posts)
1438	RefID: 1438, Awareness and Compliance with the Recommendations of Primary and Secondary Prevention of Cancer in Patients with Inflammatory Bowel Disease<br>Tulewicz-Marti, E., Stępień-Wrochna, B., Maciejewska, K., Łodyga, M., Karłowicz, K., Lewandowski, K., Rydzewska, G.<br>Level: 1, State: Excluded	Not about HPV vaccination/vax attitudes (eg HPV infection/serology/prevalence; cervical cancer; HPV vax safety)
1439	RefID: 1439, Effect of school-based human papillomavirus (HPV) vaccination on adolescent girls' knowledge and acceptability of the HPV vaccine in Ibanda District in Uganda<br>Turiho, A. K., Okello, E. S., Muhwezi, W. W., Harvey, S., Byakika-Kibwika, P., Meya, D., Katahoire, A. R.<br>Level: 2, State: Excluded	Study Design - Not qualitative (methods or analysis) OR Qualitative survey data not analyzed qualitatively (only numeric stats)
1441	RefID: 1441, Arguments in favor of and against the HPV vaccine school-entry requirement in Puerto Rico: a content analysis of newspaper media<br>Vázquez-Otero, C., Tyson, D. M., Vamos, C. A., Romero-Daza, N., Beckstead, J., Daley, E. M.<br>Level: 1, State: Excluded	Wrong publication type (eg reviews, opinions, conference proceedings)
1442	RefID: 1442, Natural history of human papillomavirus infection among college students in Liuzhou, Guangxi of China: a prospective study<br>Wei, FeiXue, Cui, XueLian, Guo, Meng, Yin, Kai, Li, MingQiang, Huang, ShouJie, Su, YingYing, Wu, Ting<br>Level: 1, State: Excluded	Study Design - Not qualitative (methods or analysis) OR Qualitative survey data not analyzed qualitatively (only numeric stats)
1443	RefID: 1443, A mixed methods study of health literacy and its role in HPV vaccine uptake among college students<br>Williams, Michelle S.<br>Level: 2, State: Excluded	Not about HPV vaccination/vax attitudes (eg HPV infection/serology/prevalence; cervical cancer; HPV vax safety)

1445	RefID: 1445, Perception about human papillomavirus vaccination among middle adolescent school girls in Addis Ababa, Ethiopia 2023: qualitative study<br>Wubu, A., Balta, B., Cherie, A., Bizuwork, K.<br>Level: 2, State: Excluded	Wrong/no population (eg parents, providers, children, policy makers, does not include 18-26yrs olds; social media posts)
1448	RefID: 1448, Incidence, persistence, and clearance of anogenital human papillomavirus among men who have sex with men in Taiwan: a community cohort study<br>Zhou, X., Tian, T., Lu, Z., Yu, Y. F., Li, Y., Zhou, Y., Lin, Y. F., Strong, C., Zou, H.<br>Level: 1, State: Excluded	Wrong publication type (eg reviews, opinions, conference proceedings)
