# Supplementary material for: Perspectives of HPV vaccine decision-making among young adults: A qualitative systematic review and evidence synthesis
Source: PLoS One. 2025 May 5;20(5):e0321448. doi: 10.1371/journal.pone.0321448 (PMC12052141; doi:10.1371/journal.pone.0321448)
Supplement: Attachment — Submitted filename: Mantina_Plos_Response to Reviewers.docx [file pone.0321448.s007.docx]

Dear Dr. Khudyakov

Thank you for the opportunity to revise our manuscript “Perspectives of HPV Vaccine Decision Making Among Young Adults: A Qualitative Systematic Review and Evidence Synthesis " (Submission ID: PONE-D-24-44380). We appreciate the careful review and constructive suggestions. We believe the manuscript is much improved after making the further suggested edits.

Following this letter are the comments from the reviewer with our responses, including how and where the text was modified. Changes made in the manuscript are indicated using tracking changes. The revision has been developed in consultation with all coauthors, and each author has approved the final form of this revision.

Thank you for your consideration.

Sincerely,

Namoonga M. Mantina

| **ACADEMIC EDITOR /JOURNAL REQUIREMENTS** AUTHOR RESPONSE | **LOCATION OF CHANGE** |
| --- | --- |
| **Please ensure that your manuscript meets PLOS ONE's style requirements, including those for file naming.**  Style guidelines were reviewed. No changes were made to the follow as they were already done in accordance with the guidelines:   - Level headings were used - Figures are appropriately cited and referenced throughout the document with captions listed directly after the paragraph they are first cited and in bold typeface - Tables are also included and listed as required. - Citation references are in number brackets - Supplementary materials are labeled and captioned as guided. |  |
| **As required by our policy on Data Availability, please ensure your manuscript or supplementary information includes the following:**   - **A numbered table of all studies identified in the literature search, including those that were excluded from the analyses.**   This information is provided in Table 1   - **For every excluded study, the table should list the reason(s) for exclusion.**   S5 Appendix is now included detailing excluded studies and the reason for exclusion   - **If any of the included studies are unpublished, include a link (URL) to the primary source or detailed information about how the content can be accessed.**   All included studies were published empirical studies in accordance with the review protocol   - **A table of all data extracted from the primary research sources for the systematic review and/or meta-analysis. The table must include the following information for each study:**   - **Name of data extractors and date of data extraction**   - **Confirmation that the study was eligible to be included in the review.**   - **All data extracted from each study for the reported systematic review and/or meta-analysis that would be needed to replicate your analyses.**   Given the total volume of qualitative data extracted, it would not be feasible to include all the data extracted in a table – the extracted data is not solely numerical. The manuscript details that data was independently extracted by 2 reviewers and discrepancies were reconciled by a third reviewer. All studies extracted were eligible for inclusion in accordance with the review protocol. All data is available in the data repository.   - **If data or supporting information were obtained from another source (e.g. correspondence with the author of the original research article), please provide the source of data and dates on which the data/information were obtained by your research group.**   Not applicable for this study.   - **If applicable for your analysis, a table showing the completed risk of bias and quality/certainty assessments for each study or outcome.  Please ensure this is provided for each domain or parameter assessed. For example, if you used the Cochrane risk-of-bias tool for randomized trials, provide answers to each of the signalling questions for each study. If you used GRADE to assess certainty of evidence, provide judgements about each of the quality of evidence factor. This should be provided for each outcome.**   Formal quantitative analyses was not done since this was a systematic review of qualitative data. GRADE CERQUAL evidence tool was used and judgements about each quality of evidence factor is detailed in S3 Appendix   - **An explanation of how missing data were handled.**   Given this review is an evaluation of qualitative data, the authors believe “missing data” is not applicable in this context. | S5 Appendix |
| **Please provide a complete Data Availability Statement in the submission form, ensuring you include all necessary access information or a reason for why you are unable to make your data freely accessible.**  The submission form has been updated to include the following: Data file are available via ReDATA, which is the University of Arizona's institutional research data repository and is managed by the Libraries. Accession numbers and/or DOIs will be made available after acceptance. |  |

| **REVIEWER 1 COMMENTS** AUTHOR RESPONSE | **LOCATION OF CHANGE** |
| --- | --- |
| **Background: The paragraph discussing the need for qualitative research could be streamlined to enhance cohesion.**  Thank you for this feedback. The authors are unclear on what the specific concerns for cohesion are that were raised by the reviewer. The paragraph on qualitative research has been reviewed and lightly edited given the collective feedback received from all reviewers. | Page 14-15 |
| **Background: To improve readability, authors should consider combining points on the CDC and WHO vaccination recommendations. Then proceed to the discussion on disparities in vaccination rates and their implications on public health.**  Thank you for the suggestion. The authors believe that combining the points on the CDC and WHO recommendation would negatively impact readability given differences in ages, dosing and timeline of their respective recommendations that are detailed. |  |
| **Background: Authors should add a brief concluding sentence summarizing the gaps in research and the importance of targeted interventions for HPV vaccination in young adults. This will provide a stronger ending.**  This comment is taken into consideration with reviewer 2’s comment on the research objectives section being a part of the introduction and not a separate section. The last paragraph of the introduction has been revised. | Page 5 |
| **Methods: The authors should consider introducing subheadings for each subsection (e.g., "Eligibility Criteria," "Information Sources and Search Strategy," etc.) to improve navigation for readers.**  Subheadings are included in manuscript according to the journal’s style guidelines. |  |
| **Methods: Authors should explain briefly why the review focuses on young adults aged 18-26 specifically. Adding a sentence to contextualize this age range would enhance the reader's understanding of the focus.**  The authors believe that this information has been provided and is justified in the introduction section. This information is proved in the last paragraph of page 3 through page 5. |  |
| **Methods:  In the sentence, “In alignment with the approaches taken by other qualitative review authors [30], we purposively sampled a subset of the studies for analysis…,” briefly clarify why purposive sampling was used (e.g., to enhance the depth of thematic analysis) as this adds rationale to your methods.**  The authors believe the requested information is provided in the preceding sentence to the one aforementioned:   - “*In QES, managing the volume of data examined is crucial to uphold analysis quality [28]. Maximizing the heterogeneity of identified themes and concepts to foster a more profound understanding of the research subject is prioritized over exhaustively assessing every study [29].”*   An additional preceding sentence has been added for more clarity/reinforcement : *“Consequently, purposively sampling methods were used to aid the management and analysis of the qualitative data to be extracted”* | Page 9 |
| **Methods: In Data Synthesis, this section could be expanded slightly to explain the coding process more clearly, particularly how codes were created, discussed, and agreed upon. The authors should explicitly mention how discrepancies in coding were handled to enhance transparency.**  The authors detail the data synthesis process according to the methodological process followed. We detail (on page 11) how codes were created –notes from reviewing the extracted data, inductively coding a subset of studies to identify initial codes, meeting to discuss the initial codebook followed by another round of preliminary coding to test the codebook. We also detail that discrepancies in coding for each study were handling by a meeting of the review authors who coded the data who would determine the finalized coding of the data. |  |
| **Methods: The authors should add a sentence to explain why GRADE-CERQual was chosen (does it assess confidence in qualitative evidence rigorously?). This would give this section more depth.**  The authors have added a statement this tool was used as recommended by the Cochrane handbook on QES to evaluate the confidence in review findings of qualitative research. Cochrane is generally accepted as the gold standard for reviewed among the scientific community and this review follows their methodology/handbook in conducting qualitative reviews (a statement provided at the beginning of the methods section – 5). The reference is cited. The manuscript details the criteria by which the confidence is assessed. | Page 11 |
| **Methods: Was there ethical considerations? If yes, consider including a statement on ethical considerations or any limitations of the methodology, particularly around sampling and data extraction.**  Ethical considerations were assessed as part of the assessment on methodological limitations for each included study. The findings from this process are reported on page 30 |  |
| **Methods: Ensure consistent formatting for tool names and software (e.g., "DistillerSR," "EndNote," "REDCap").**  This information has been reviewed. Tools are listed according to the citation preference given by each tool. |  |
| **Methods: Use italics for journal names, e.g., BMJ Open.**  No journal names are listed in the methods section. The citation format of the manuscript followed the journals guidelines, utilizing their specific citation style file for endnote. |  |
| **Methods: Ensure that all acronyms, like "QES" and "CASP," are spelled out on the first mention to improve readability.**  All acronyms are spelled out the first time they are used. |  |
| **Discussion: When discussing barriers like “the cost of and accessibility to the HPV vaccine,” please provide more context or examples to illustrate these barriers. For instance, “limited financial support? etc”**  Thank you for this comment. Additional information has been added for context. | Page 57 |
| **Discussion: While the authors discuss parental and physician influence, they should consider addressing why these factors are relevant for young adults specifically. A sentence explaining why young adults may still be influenced by their parents or need strong physician support would add depth.**  Additional information has been added. | Page 58 |
| **Discussion: The authors should expand on the limitations section, particularly regarding the geographic and age restrictions. They could clarify how these limitations might affect the generalizability of their findings**.  Additional information has been provided detailing implications on the mentioned limitations. | Page 59 |
| **Discussion: The authors should also consider suggesting future research directions based on these limitations, such as studying younger adolescents' transition into adulthood or assessing the impact of parental influence on older adolescents.**  Thank you for this feedback. This comment has been taken into consideration along with a comment from reviewer 2 regarding clearer emphasis on points of intervention. Additional information has been added to the discussion section | Page 59-60 |

| **REVIEWER 2 COMMENTS** AUTHOR RESPONSE | **LOCATION OF CHANGE** |
| --- | --- |
| **The ABSTRACT section is succinct and sufficient.**  Thank you for this feedback. |  |
| **The research objectives can be incorporated into the INTRODUCTION section.**  We have removed the section title (research objectives) | Page 5 |
| **Table 2 [page 27] is a very good summary of the 71 articles representing 68 studies that met the eligibility criteria and the 45 articles representing 42 studies that were included and synthesized for the review.**  Thank you for this feedback! We wanted to find a helpful way to detail this information between the eligible studies and the included studies. |  |
| **The study is very thorough and detailed. While this is essential to the comprehensiveness of the manuscript, it may impact on the length of the paper.**  We have tried to be mindful of the length of the manuscript while also providing sufficient details regarding the research process. Overall, we are compliant with the journal guidelines which does not enforce restriction on word count. The length of the manuscript is also greatly impacted by the tables, one of which spans as many as 12 pages but is a required table to report in the text of systematic reviews (table 1 – characteristics of studies) |  |
| **There are 10 thematic categories identified [page 35]. Perhaps a one-line definition of each theme may be necessary given that not all of the themes are self-explanatory. Further, looking at the specific findings per theme, there seems to be some overlap. For instance, the findings of theme 4 (sex and romantic relationships) overlap with or may be related to the findings of theme 1 (individual factors).**  Thank you for this observation. We also encountered this during the analysis and discussion of how we would present the findings, acknowledging that these elements may not truly be distinct and may be related to other themes. We have added a sentence to acknowledge the overlap. Overall, we endeavored to present the data/findings according to the prevailing thematic group we thought most helpful/relevant.  One-line definitions of the themes have been added | Page 36  Page 36-37 |
| **If the manuscript needs to be shortened or made more concise, perhaps the themes can be combined to lessen their number and to highlight possible points of intervention, which may also enrich the discussion. For example, themes 1, 3, 4, and 9 can be placed under one theme relating to proper information; themes 5 and 6 can be under the theme regarding influences to decision-making; and themes 2, 7, 8, and 10 relate to the vaccine itself, including alternatives, logistics, and policy. The number of findings will not change, only the themes that cover them. This will make the manuscript more focused and enable it to posit more focused interventions.**  Thank you for the suggestion. While collapsing the themes is possible, given the number of findings is maintained, there is unlikely to be substantial change to the length of the manuscript. However, this information has been well received to inform enrichments to the discussion section on points of interventions. | Page 59-50 |
| **The DISCUSSION section mentions that the findings “highlight several constraints that young adults experience in getting vaccinated” [page 56]. However, there is no discussion, no matter how short, on possible action areas. Some recommendation may be helpful, especially in the context of the relevance underscored in the INTRODUCTION section stated as “(T)his presents a crucial opportunity to intervene and promote catch-up HPV vaccination” [page 3].**  Thank you for this feedback. This comment has been taken into consideration along with a comment from reviewer 1. Suggestions for future research has been added to the section referenced. Additionally, more information has been added to the discussion on action areas/future interventions. | Page 58  Page 58 - 60 |
| **A brief CONCLUSION section may help.**  A conclusion section has been added. | Page 60 |
